# A data-driven evaluation of optimization techniques in cell culture media

**DOI:** 10.1093/bioinformatics/btag607

**Published:** 2026-08-19

**Authors:** Ali Parsaee, Miranda Stahn, Arian Amirvaresi, Reza Ovissipour, David Staszak

**Affiliations:** Alberta Machine Intelligence Institute (AMII), Edmonton, AB T6E 4S6, Canada; New Harvest, Edmonton, AB T6E 1M4, Canada; Department of Food Science and Technology, Texas A&M University, College Station, TX 77843-2256, United States; Department of Food Science and Technology, Texas A&M University, College Station, TX 77843-2256, United States; Alberta Machine Intelligence Institute (AMII), Edmonton, AB T6E 4S6, Canada

## Abstract

**Motivation:**

Optimizing cell-culture media for cultivated meat and cellular agriculture is both experimentally demanding and expensive. Traditional approaches use Design of Experiments (DOE) to iterate through candidate formulations until a satisfactory medium emerges. Data-driven methods, and in particular Bayesian optimization (BO), can reach higher objective function values (here, titer) with fewer evaluations than classical DOE. Prior comparisons of optimizers on cell-media data have, however, relied largely on computational simulations rather than measured formulations, leaving researchers without evidence-based guidance on which strategy to adopt.

**Results:**

We introduce two complementary benchmark protocols and apply them to six published cell-culture datasets. In the “Hide-the-Label” protocol each optimizer begins with only a partial view of the data and sequentially reveals hidden formulations, singly or in batches, reproducing the stepwise acquisition of costly laboratory measurements; the “Open Race” instead measures search efficiency of a method under a fixed evaluation budget. Both protocols were run across four generative surrogate classes [Gaussian process (GP), random forest, neural network, and Bayesian neural network], two difficulty regimes, and batch sizes of one to 20. GP-based BO was consistently the most sample-efficient strategy, locating hidden targets in substantially fewer evaluations than classical DOE designs or random search; its advantage narrowed but remained under injected noise and multi-modality, and optimizers built on more complex surrogates (deeper networks or adaptive ensembles) performed worse rather than better on these harder landscapes. We detail the mathematical basis of the benchmark, criteria for selecting suitable datasets, and how to obtain statistically robust conclusions in the presence of measurement bias and heterogeneity, and we provide a roadmap for selecting an optimization strategy in practice.

**Availability and implementation:**

Source code and the benchmark datasets are freely available at https://github.com/Amii-Applied-AI/amii-cell-ag-tools/tree/main/active-learning-for-cell-media and archived at https://doi.org/10.5281/zenodo.21501659.

## 1 Introduction

Optimizing cell culture media is a critical and fundamental bottleneck in modern biotechnology, with profound implications for bio-manufacturing and the growing field of cellular agriculture (Todhunter *et al.* 2024). The objective is to formulate a medium that provides the precise balance of dozens of nutrients, growth factors, and supplements necessary to maximize cellular proliferation and productivity. However, the design space of potential formulations is combinatorially vast; even with a modest number of components varied across a few concentrations, the number of possible combinations can easily run into the billions, making exhaustive searches impossible. This challenge is compounded by the resource-intensive nature of experimental validation. Each candidate formulation requires significant investment in terms of costly reagents, sterile consumables, and specialized labor, as well as long incubation periods, severely limiting experimental throughput. For cellular agriculture, where achieving price parity with conventionally farmed meat is a primary goal, the high cost of media represents a particularly formidable economic barrier, often accounting for the majority of production expenses (Post *et al.* 2020, Specht 2020, Swartz *et al.* 2021).

Over the past decades, formulations have progressed from ill-defined serum-based mixtures to chemically defined media (Galbraith *et al.* 2018), yet a typical medium still contains 20–50 components whose effects are non-linear, interactive, and incompletely understood (Galbraith *et al.* 2018, Hashizume and Ying 2024). In CHO-based bio-pharmaceutical manufacturing, individual nutrients exhibit concentration-dependent, non-monotonic effects on growth and product quality (Galbraith *et al.* 2018), and comparative analyses—e.g. data-driven regressors versus genome-scale metabolic models—show that integrating ML with systems biology yields better predictions than either alone (Kavoni *et al.* 2025). Analogous challenges arise for cultivated-meat cell lines, where serum-free media must maximize proliferation while meeting cost targets (Cosenza *et al.* 2021). Comprehensive reviews consistently find that no single optimization strategy dominates across all cell types and objectives (Zhou *et al.* 2023, Hashizume and Ying 2024, Kavoni *et al.* 2025).

Historically, this complex optimization challenge has been addressed using classical methodologies, most notably Design of Experiments (DOE). Approaches such as fractional factorial designs and Response Surface Methodology (RSM) represent a significant statistical improvement over rudimentary one-factor-at-a-time testing by allowing for the study of interacting variables (Montgomery 2017). These methods typically involve fitting local, low-order polynomial models to experimental data to map out a response surface. However, a key limitation of standard DOE is its non-adaptive nature; the full set of experiments is typically defined before the study begins. Consequently, in the high-dimensional, non-linear, and often multi-modal landscapes characteristic of media optimization, DOE can be inefficient, requiring a large number of experiments to locate a global optimum (Zhou *et al.* 2023). Efforts to extend DOE with nonlinear surrogate models have offered partial relief: Cosenza *et al.* (2021) showed that a hybrid coordinate-search genetic algorithm could identify an optimal serum-free formulation for rat muscle cells with substantially fewer experiments than conventional grid-based DOE, while also reducing medium cost—yet even this improvement remains constrained by the static, non-adaptive nature of the design.

In parallel, recent advancements in machine learning have introduced powerful, data-driven strategies that are exceptionally well-suited to this problem domain. Bayesian optimization (BO) has emerged as a particularly promising alternative, framing the optimization process as a sequential, adaptive decision problem (Frazier 2018). BO operates in a cycle: it first builds a probabilistic surrogate model (commonly a Gaussian Process) to create a statistical representation of the objective function from available data. It then uses an acquisition function to intelligently identify the next most informative experiment to perform, balancing exploration of uncertain regions with exploitation of promising ones. Despite its demonstrated sample efficiency and suitability for expensive black-box functions, BO remains underutilized in industrial and academic bioprocess development, where classical DOE continues to be the dominant paradigm (Hashizume and Ying 2024). The slow adoption of these advanced methods can be attributed to several factors: a lack of familiarity within the experimental community, a perception of BO as an uninterpretable “black box” compared to the linear models of DOE, and a historical scarcity of accessible case studies demonstrating its real-world success (Shahriari *et al.* 2016).

Beyond GP-based BO, complementary data-driven approaches have emerged: Ozawa *et al.* (2025) presented a data-driven workflow for cell culture medium optimization that systematically compared ML-guided reformulation strategies; Gangwar *et al.* (2024) applied explainable ML with SHAP to CHO cultures, reporting that ensemble tree models achieved $R^{2}\approx 0.92$–0.95 and identifying metal-ion concentrations, particularly iron and zinc, as key drivers of antibody quality; and Hashizume and Ying (2025) proposed a biology-aware ML framework incorporating metabolic constraints directly into the model architecture. These studies reflect a growing consensus that the frontier lies in integrating data-driven modeling with biological domain knowledge (Hashizume and Ying 2024, Kavoni *et al.* 2025).

A crucial barrier to wider BO adoption is the lack of head-to-head comparisons on realistic data; existing benchmarks often rely on synthetic functions that may not capture biological noise and batch effects (Zhou *et al.* 2023). This study introduces a “hide-the-label” protocol on publicly available cell-media datasets: each optimizer starts with a partial view and sequentially “purchases” hidden data points, faithfully mimicking laboratory resource constraints. The protocol is complemented by an “Open Race” competition on learned functional embeddings (Busetto *et al.* 2025) and by techniques to de-bias historical datasets. By casting media formulation as an active-learning problem, this work bridges ML research and bioprocess engineering, providing an evidence-based roadmap for selecting optimization strategies. A public GitHub code repository is provided at https://github.com/Amii-Applied-AI/amii-cell-ag-tools/tree/main/active-learning-for-cell-media, and an archived release is deposited at https://doi.org/10.5281/zenodo.21501659. Readers should refer to the README.md for usage details. A landing page can be found at https://amii-applied-ai.github.io/amii-cell-ag-tools/.

## 2 Related work

Cell culture media optimization has evolved from serum-supplemented formulations in the 1950s to chemically defined media developed with advanced statistical and computational techniques (American Pharmaceutical Review 2019, Jayme and Smith 2019) (https://www.americanpharmaceuticalreview.com/Featured-Articles/559489-CHO-Media-Development-for-Therapeutic-Protein-Production/). This work builds on three domains: classical experimental design, sequential model-based optimization, and benchmarking for bioprocesses.

### 2.1 Classical design of experiments in media formulation

The Design of Experiments (DOE) framework has been the standard for multi-factor optimization in biotechnology for decades (Mandenius and Brundin 2008, Montgomery 2017, O’Kennedy 2019, Kasemiire *et al.* 2021). However, classical DOE is inherently non-sequential: the full experiment set is designed upfront, which can be inefficient when the optimum lies outside the initial region (Parampalli *et al.* 2007, Puente-Massaguer *et al.* 2019). Efficacy also declines in high-dimensional spaces due to the curse of dimensionality (Myers *et al.* 2016). [Bayesian optimization faces its own dimensionality challenges—surrogate coverage degrades beyond ∼10 to 20 dimensions (Shahriari *et al.* 2016)—but the key distinction is that DOE suffers an *experimental design* curse whereas BO suffers a *search* curse; in the 7–30 dimensional settings typical of cell media, BO retains a practical advantage.] The core limitation is that sampling points are fixed from the outset, preventing adaptation as data accumulates (Seufert *et al.* 2021).

### 2.2 Sequential optimization and Bayesian methods

Sequential optimization strategies use an iterative “Design-Build-Test-Learn” cycle, updating the model after each experiment (Zhou *et al.* 2023). Bayesian optimization (BO) has emerged as particularly sample-efficient for black-box problems, coupling a GP surrogate with an acquisition function that balances exploration and exploitation (Shahriari *et al.* 2016, Frazier 2018). Studies in bioprocessing confirm that BO can identify superior formulations with significantly fewer experiments than DOE (Cosenza *et al.* 2022, Narayanan *et al.* 2025). This advantage stems from the surrogate model’s ability to generalize from sparse observations: rather than covering the design space uniformly, BO concentrates evaluations where the predicted improvement is largest, making each experiment maximally informative (Shahriari *et al.* 2016, Frazier 2018).

### 2.3 Benchmarking optimizers in biological systems

Despite the proliferation of optimization algorithms, direct head-to-head benchmarks in cell culture remain rare (Zhou *et al.* 2023). Existing suites like BBOB target synthetic functions and may not capture biological noise and resource constraints. Other fields are further ahead: Olympus (Hickman *et al.* 2023) (materials science) and GuacaMol (Brown *et al.* 2019) (molecular design) provide toolkits for comparing algorithms on real experimental data. The absence of analogous benchmarks for cell media may partly explain the slow adoption of advanced methods in this domain (Hashizume *et al.* 2023), motivating the present study.

## 3 Materials and methods

To compare the performance of optimization algorithms in a realistic bioprocessing context, a simulation framework was developed utilizing existing public cell media formulation datasets. The framework uses two distinct benchmarking protocols: a “Hide-the-Label” protocol on experimental data and a fixed-budget “Open Race” on synthetic functions. For all the following methods, surrogate models are initially trained on the data. Models are only accepted if their $R^{2}$ value exceeds a pre-specified acceptance threshold. “Accepted” here means the fitted surrogate is used as the generative model for synthetic dataset creation; if a fitted model fails to reach $R^{2}>0.8$ on leave-one-out cross-validation, the corresponding dataset is excluded from the benchmark rather than generating unreliable synthetic data from a poorly fitting surrogate. For the experiments the trained Gaussian process, random forest, neural network, and Bayesian neural network models all had $R^{2}>0.8$. This threshold was selected as an empirical criterion indicating that the surrogate model explains at least 80% of the variance in the observed labels, providing a reasonable assurance that the learned landscape faithfully represents the true objective surface; values in this range are widely used as acceptance criteria in applied regression and experimental design (Montgomery 2017). However, it is important to note that $R^{2}$ measures point-prediction accuracy alone and does not directly assess uncertainty calibration—a property that is particularly critical for Bayesian optimization methods, where the acquisition function relies on accurate posterior variance estimates in addition to the posterior mean. A surrogate with high $R^{2}$ may still produce overconfident or poorly calibrated uncertainty intervals, potentially biasing exploration toward regions that appear uncertain only because the model is miscalibrated rather than genuinely unexplored. While the precise cutoff is somewhat arbitrary, values in the range 0.75–0.90 are widely used in applied surrogate modeling and the 0.8 criterion is intentionally conservative enough to exclude surrogates with weak explanatory power. A formal sensitivity analysis of how the benchmark conclusions change as the acceptance threshold varies, together with complementary calibration diagnostics—such as leave-one-out cross-validation of predictive intervals or the expected calibration error—would provide a more complete picture of surrogate quality beyond predictive accuracy, and we flag this as a direction for future work.

### 3.1 Public datasets for simulation

Six publicly available experimental datasets from previous cell culture medium optimization studies were used as the foundation for the simulations.

1. **Human hematopoietic cells:** The T-Cell and TF-Cell datasets from Kim and Audet (2019), optimized for serum-free expansion of human T-cells and a myeloid progenitor line (TF-1).
2. **Rat myocytes:** The MOBO rat myocyte and DBO rat myocyte datasets from Cosenza *et al*. (2023), focused on designing low-cost, high-growth serum-free media for rat myoblasts.
3. **HeLa-S3 cells:** The Human HeLa regular mode and Human HeLa timesaving mode datasets from Hashizume *et al*. (2023), where 29 medium components were optimized for a human cell line using active learning.

While there are no publicly available datasets for food-relevant cell lines derived from animals commonly consumed by humans, these selected studies represent the closest available analogues. They involve mammalian muscle progenitor, immune, and cancer cell lines optimized with DOE or Bayesian optimization methods. The rat myocyte datasets, in particular, provide a relevant proxy for muscle cell proliferation. These resources are indispensable for developing and validating optimization methods until dedicated datasets for cellular agriculture become available.

### 3.2 The hide-the-label protocol

This protocol simulates a real-world experimental study where resources are limited. The benchmark begins with an experimental dataset where each input (media formulation) is associated with a measured output (titer). The dataset is partitioned into a small, initially *visible* set and a larger *hidden* set, which contains the best-performing formulation (the target).

An optimization algorithm is then tasked with sequentially selecting a batch (of size 1 or more) formulations from the hidden set to “reveal,” using the accumulating data from the visible set to inform its next choice. The primary performance metric is the number of iterations required to identify the hidden target. To mitigate sampling bias inherent in the original datasets, a generative approach was used. A Bayesian model was fitted to the entire experimental dataset and then used to generate multiple synthetic datasets. This provided a debiased and reproducible ground truth for robustly evaluating algorithm performance across many replicates. A formal outline of this method is detailed in Algorithms 1 and 2, available as supplementary data at *Bioinformatics* online. For surrogate model specification and synthetic data generation: In Regular Mode, a GP with a Matérn-$\frac{5}{2}$ kernel (length_scale = 0.3, WhiteKernelnoise_level = $10^{-6}$, hyperparameters optimized by marginal log-likelihood) is fitted to each experimental dataset. In Hard Mode, three alternative generative surrogates are used: a Random Forest (100 estimators, max_features=’sqrt’, unlimited depth, bootstrap sampling); a Neural Network (two hidden layers of width 100 and 50, ReLU, Adam with learning rate $10^{-3}$, batch size 32); and a Bayesian Neural Network via MC-Dropout (layers 128 and 64, dropout rate 0.2, 50 MC samples, Adam with learning rate $10^{-3}$, up to 300 epochs). All surrogates receive standardized inputs (zero mean, unit variance). Synthetic datasets are produced by drawing $N_{\text{pts}}=200$ points uniformly from the axis-aligned bounding box of each experimental dataset, labeling them by querying the fitted surrogate, and repeating with independent random seeds to produce 30 synthetic datasets per cell line. Exact implementation details and random seeds are available in the code repository. Some experimental datasets contain replicate measurements of the same formulation. Where replicates exist, the surrogate model is fitted to all available observations including replicates; the surrogate’s own regression smooths over replicate variability during fitting. Because the benchmark runs entirely on *synthetic* datasets generated by the fitted surrogate—and the original experimental data is never partitioned into train and test splits—there is no risk of information leakage between the visible and hidden sets. A final note: many DOE methods may propose points outside the existing dataset. In such cases, the closest available point in the dataset—based on L2 distance—is selected instead. This constraint does not apply in the Open Race protocol described below.

### 3.3 The open race protocol

The Open Race protocol provides a complementary benchmark that isolates an algorithm’s search efficiency from dataset noise and bias. In this setting, optimizers query a known, stationary ground-truth function—typically a surrogate model (e.g. Gaussian Process, Random Forest, Neural Network, or Bayesian Neural Network) trained on experimental data. Initially they start off knowing the function values of a few points, making this their *visible* set.

Each optimizer is given a fixed budget of 200 queries and may evaluate batches of one or more points while searching for the input that maximizes the function value. All algorithms begin with the same initial points and operate under identical conditions, ensuring that performance differences are attributable to the optimization strategy alone. The primary performance metric is the maximum function value achieved within the budget. This process, repeated across multiple competitions, provides a controlled and reproducible testbed for assessing algorithmic behavior. A formal outline of this method is detailed in Algorithms 3 and 4, available as supplementary data at *Bioinformatics* online.

### 3.4 Changing problem difficulty

To ensure fair evaluation, the benchmarking framework varies problem difficulty along two independent axes. First, alternative surrogate classes replace the GP as the generative model: Random Forests (RF) produce piecewise-constant landscapes that break GP smoothness assumptions, Neural Networks (NN) provide smooth but potentially non-stationary surfaces, and Bayesian Neural Networks (BNN) additionally provide calibrated uncertainty estimates—each tests optimizers in a structurally distinct setting. The *primary* purpose of this surrogate switching is debiasing: ensuring benchmark landscapes are not aligned with GP assumptions, producing a fairer comparison across all optimizer classes. In the Open Race protocol the alternative surrogate serves as the queryable ground-truth function; in Hide-the-Label it generates the synthetic datasets. Second, explicit difficulty factors are injected on top of any surrogate: (i) *homoskedastic noise* adds $\varepsilon ∼N(0,\sigma^{2})$ with σ=0.1 to each surrogate output; (ii) *heteroskedastic noise* scales the standard deviation with the local function value, σ(x)=0.1|f(x)|+0.01, so that high-response regions are noisier; (iii) *discontinuities* are introduced by placing $n_{\text{disc}}$ random hyperplanes in the input space—each defined by a unit normal vector and a random anchor point drawn from the data bounding box—and adding a fixed jump of magnitude discontinuity_strength to all outputs on the positive side of each plane; and (iv) *multi-modality* is induced by replacing the training labels with a weighted sum of $n_{\text{modes}}$ mode components, where each component shifts the inputs to a randomly placed center and weights the contribution by a Gaussian kernel $\text{exp}(-\|x-c_{i}{\|}^{2}/(2s^{2}))$ with separation *s*, creating localized optima that can trap greedy algorithms. Noise and multi-modal perturbations are applied to the training data before the surrogate is fitted. In Regular Mode the surrogate is queried without difficulty injection; in Hard Mode, the difficulty configuration combines heteroskedastic noise (σ(x)=0.1|f(x)|+0.01) with bi-modal perturbations ($n_{\text{modes}}=2$, separation s=2.0), applied on top of each surrogate class (GP, RF, NN, or BNN). Discontinuities and homoskedastic noise are implemented in the code repository as an extended testbed for future work. In Hide-the-Label these difficulties are built into the generated datasets. In the Open Race benchmark, the same factors are applied stochastically at query time: the underlying objective function is fixed throughout, but each evaluation returns a noisy or transformed observation (e.g. with additive noise $\varepsilon ∼N(0,\sigma^{2})$ applied at the moment of querying). The objective surface itself does not change during optimization; rather, optimizers receive stochastically corrupted feedback at each query, mirroring the irreducible measurement noise of real laboratory experiments. Further details are provided in the “Changing problem difficulty” section, available as supplementary data at *Bioinformatics* online.

### 3.5 Statistical analysis framework

To compare optimizers across both protocols, a unified non-parametric framework was used. In Hide-the-Label (HtL) the response variable was the number of queries to reveal the global maximum, $t^{*}(z,c,j)$, where *z* indexes the synthetic dataset, *c* the competition, and *j* the optimizer. In Open Race (OR), the response was b(z,c,j), the best (“b”) function value achieved within a fixed budget of $J_{q}$ queries:


$$b(z,c,j)=\text{max}\{f(x_{1}^{\left(0\right)}),\ldots ,f(x_{n}^{\left(0\right)}),f(x^{\left(1\right)}),\ldots ,f(x^{\left(J_{q}\right)})\}.$$

Each instance i=(z,c) is observed across all optimizers, enabling paired comparisons.

The non-parametric Friedman test was applied to test $H_{0}$: all optimizers have equal performance distributions, treating instances as blocks and optimizers as treatments (α=0.05). When global differences were detected, pairwise comparisons were conducted: for each optimizer pair (a,b) the per-instance difference $\Delta_{i}^{\text{HtL}}=t^{*}(z,c,a)-t^{*}(z,c,b)$ (resp. $\Delta_{i}^{\text{OR}}$ for Open Race) was tested via a two-sided sign-flip permutation test with Holm correction. Effect sizes were reported as Hodges–Lehmann median paired differences with 95% bootstrap confidence intervals. As a conservative check, results were aggregated within datasets (median over competitions per optimizer) and the tests repeated at the dataset level. A Monte Carlo power analysis (1000 replicates per design configuration) confirmed that ≥30 competitions across 30 synthetic datasets with 200 points each yields power above 0.95. The synthetic function used for the power analysis was a linear-in-parameters model with eight input variables, of which a subset were assigned non-zero effect sizes (β=1) and the remainder set to zero; realistic complexities including multicollinearity among inputs, mild non-linearity, and heteroscedastic observation noise were added to match the statistical structure of the real cell-culture datasets. Full details of the power-analysis synthetic function and simulation protocol are provided in the “Statistical Analysis Framework” and “Power Analysis” sections, available as supplementary data at *Bioinformatics* online.

## 4 Bayesian optimization methodology

Bayesian optimization (BO) is a sequential, model-based framework for finding $x^{*}={\text{argmax}}_{x\in X}f(x)$ from noisy observations y=f(x)+ε, $\varepsilon ∼N(0,\sigma_{n}^{2})$, where each evaluation is expensive (Shahriari *et al.* 2016, Frazier 2018). BO couples a probabilistic surrogate model—typically a Gaussian Process (GP)—with an acquisition function that balances **exploration** of uncertain regions and **exploitation** of promising ones.

A GP places a non-parametric prior over functions, $f∼GP(m(x),k(x,x^{\prime }))$, where the kernel *k* encodes smoothness assumptions [e.g. Matérn-$\frac{5}{2}$ (Stein 1999, Rasmussen and Williams 2006)]. Given observations $D_{t}={\left\{(x_{i},y_{i})\right\}}_{i=1}^{t}$, the posterior $f(x_{*})|D_{t}∼N(\mu_{t}(x_{*}),\sigma_{t}^{2}(x_{*}))$ provides both a prediction and its uncertainty. The acquisition function $\alpha_{t}(x)$ converts these into a selection rule; the next point is $x_{t+1}={\text{argmax}}_{x\in X}\alpha_{t}(x)$. The two acquisition functions used in this work are Expected Improvement (EI),


(1) $$\begin{array}{cc} \text{EI}(x)=(\mu_{t}(x)-f(x^{+}))\Phi (z)+\sigma_{t}(x)\phi (z), & \\ & \text{where}\;z=\frac{\mu_{t}(x)-f(x^{+})}{\sigma_{t}(x)}, \end{array}$$

with Φ and ϕ the standard normal CDF and PDF and $f(x^{+})$ the best value observed so far, and Upper Confidence Bound $\text{UCB}(x)=\mu_{t}(x)+\kappa \sigma_{t}(x)$, which trades off the predicted mean against uncertainty via κ.

In practice, BO iterates: (i) fit the GP to $D_{t}$; (ii) maximize the acquisition function to select $x_{t+1}$; (iii) evaluate $f(x_{t+1})$; (iv) augment $D_{t+1}=D_{t}\cup \{(x_{t+1},y_{t+1})\}$ and repeat (Cosenza *et al.* 2022). A fuller treatment of GP regression, kernel selection, and the BO loop is provided in the “Bayesian Optimization Methodology” section, available as supplementary data at *Bioinformatics* online.

## 5 Design of experiments methods (non-Bayesian optimization)

**Figure 1:**
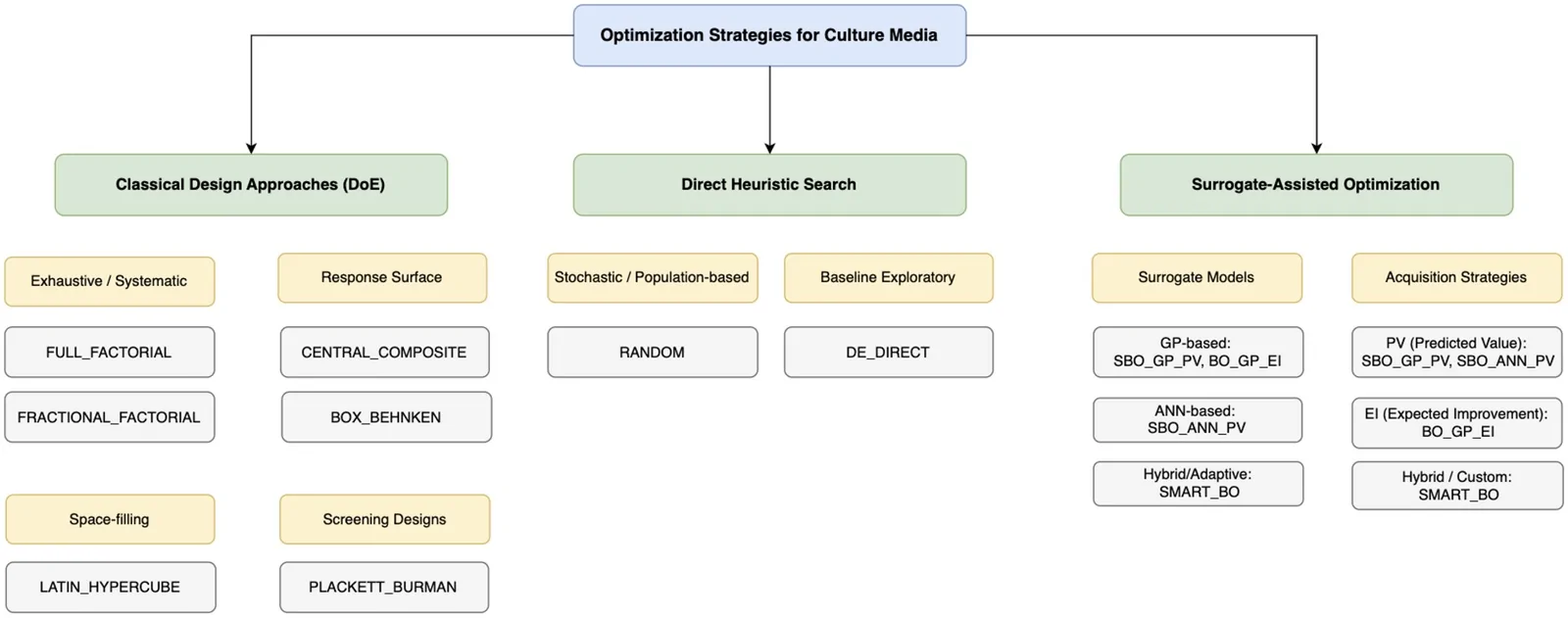
Optimization strategies for culture media: overview of classical DoE, direct heuristic search, and surrogate-assisted methods, mapping each to the specific optimizers used in this work.

To benchmark Bayesian strategies, a suite of non-Bayesian optimization methods drawn from classical and modern design of experiments (DOE) and direct search heuristics were implemented. The set of all optimization methods (Bayesian and other) discussed in this section are categorized in Fig. 1, the figure is inspired by Fig. 2 in Zhou *et al.* (2023). The non-Bayesian methods provide three complementary functions: (i) diverse exploration without strong priors through orthogonal, rotatable, or space-filling trial designs (e.g. factorial, CCD, LHS), (ii) unbiased baselines and optimistic upper bounds, since DOE points are chosen independent of outcomes while black-box optimizers query the true function directly; and (iii) robustness checks under non-ideal laboratory conditions, where Bayesian surrogates may fail due to noise, cliffs, or discreteness. Detailed descriptions of the implemented strategies—including Full Factorial, Fractional Factorial, Plackett–Burman, Central Composite, Box–Behnken, Latin Hypercube, Differential Evolution, and Random Sampling—are provided in the “Design of Experiments Methods (Non-Bayesian Optimization)” section, available as supplementary data at *Bioinformatics* online.

More detailed information for each optimizer, as well as how non-adaptive DOE methods are extended to multi-step/batch settings, are provided in the “Optimizer Definitions and Implementation Details” section, available as supplementary data at *Bioinformatics* online.

## 6 Results and discussion

All optimizers were evaluated in two distinct benchmarking protocols: the resource-constrained “Hide-the-Label” protocol and the fixed-budget “Open Race.” The results reveal a consistent performance hierarchy, with Gaussian Process-based Bayesian optimization methods demonstrating superior sample efficiency compared to classical and other model-based strategies. Throughout the Results, we use *robustness* to mean maintaining competitive performance across diverse datasets, noise levels, and batch sizes without problem-specific tuning. For further exploration results, additional figures and tables (Tables 3–8, available as supplementary data at *Bioinformatics* online report the summary statistics and pairwise comparisons) can be found in the “Additional Results” section, available as supplementary data at *Bioinformatics* online.

### 6.1 Hide-the-label competition

In the Hide-the-Label protocol, performance was measured by the mean number of steps required to identify the hidden optimal formulation; lower values indicate better performance. Figure 7, available as supplementary data at *Bioinformatics* online in the “Additional Results” section, available as supplementary data at *Bioinformatics* online shows the results for one of the settings, namely a hard version of the Hide-the-Label algorithm with a batch size of 10. More results can be found in the “Additional Results” section, available as supplementary data at *Bioinformatics* online and the pattern between optimizers remains the same.

Across all six datasets, a clear and consistent pattern emerged: Bayesian optimizers using a Gaussian Process (GP) surrogate, such as BO_GP_EI and SBO_GP_PV, were substantially more efficient than all other methods. These GP-based optimizers consistently located the target in approximately **2–6 steps** (representative configuration: HTL, Regular Mode, GP surrogate, hidden fraction = 95%, Batch size = 10; the exact threshold shifts with batch size and mode—see Tables 1 and 2, available as supplementary data at *Bioinformatics* online for full per-configuration statistics).

In stark contrast, classical Design of Experiments (DOE) strategies—including factorial, central composite, and Box-Behnken designs—along with random search, performed poorly. These non-adaptive methods required at least **6 steps** and often substantially more to find the target (same representative configuration: HTL, Regular Mode, GP surrogate, hidden fraction = 95%, Batch size = 10), indicating an inability to focus the search on promising regions of the design space. This pattern seems to hold true in both the regular and the harder modes of Hide-the-Label.

Notably, more complex Bayesian variants using artificial neural network surrogates (SBO_ANN_PV) did not seem to offer as much of a competitive advantage. In most cases, they performed worse than the simpler GP-based approaches, suggesting that their increased model complexity was not beneficial in these experimental regimes. The overall performance ranking remained stable across all datasets: GP-based Bayesian optimization was the most effective, followed by other Bayesian methods, with classical DOE and random search forming a distinct, lower-performing cluster. The summary statistics for the statistical analysis reinforce these results and can be found in Tables 1 and 2, available as supplementary data at *Bioinformatics* online in the “Changing number of initial points provided” section, available as supplementary data at *Bioinformatics* online.

### 6.2 Open race competition

**Figure 2:**
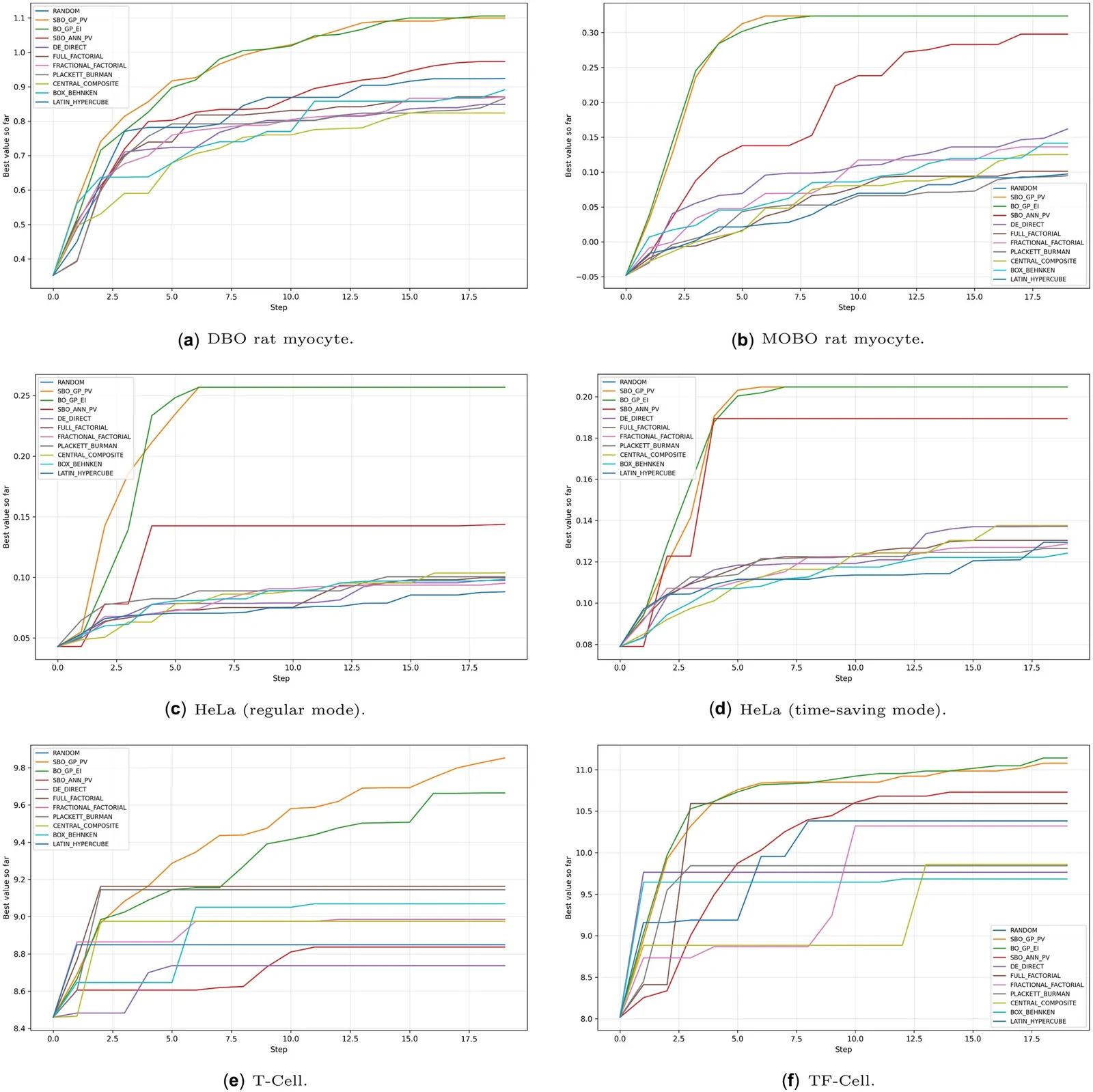
Open Race (OR) competition results. Each panel shows step-function line plots of the best value found so far over cumulative evaluation steps (higher is better). Configuration: Regular Mode (GP surrogate, low noise), hidden fraction = 95% (5% initially revealed), Batch size = 10. Datasets: DBO rat myocyte (a), MOBO rat myocyte (b), HeLa regular mode (c), HeLa time-saving mode (d), T-Cell (e), TF-Cell (f). Optimizers are ordered in the legend: Classical DOE → Direct Heuristic → Surrogate-Assisted. This exact configuration can be reproduced interactively in the online playground.

The Open Race competition measured the ability of each optimizer to find the highest-performing formulation within a fixed budget of evaluations. Figure 2 shows the best value found over time for each optimizer.

The results reinforce the findings from the Hide-the-Label protocol. GP-based Bayesian optimizers exhibited a rapid and steep learning curve, identifying near-optimal solutions within the first **10 evaluations** (in this configuration: Open Race, Regular Mode, GP surrogate, hidden fraction = 95%, Batch size = 10, averaged over six datasets; the convergence advantage persists under Hard Mode and other batch sizes, though the exact threshold varies—see the interactive playground for other configurations). Their performance quickly converged to a high plateau that other methods failed to reach.

Classical DOE methods showed minimal improvement after their initial space-covering evaluations, plateauing early at suboptimal values. This behavior highlights their inefficiency in black-box optimization tasks where sequential learning is critical. The more complex Bayesian models (SMART_BO and SBO_ANN_PV) demonstrated inconsistent performance, occasionally making progress but generally lagging behind the more robust GP-based methods.

In summary, the Open Race demonstrates that GP-based Bayesian optimization not only finds good solutions faster but also consistently converges to superior solutions compared to the other tested strategies.

### 6.3 Summary

Across both benchmarking protocols, a clear conclusion emerges: the choice of optimization strategy has a profound impact on experimental efficiency.

- **GP-based Bayesian optimization** consistently demonstrated the highest sample efficiency. Its ability to intelligently balance exploration and exploitation makes it exceptionally well-suited for resource-constrained experimental campaigns, such as cell media development.
- **Classical DOE methods**, being non-adaptive, proved inefficient for sequential optimization, consistently requiring two to three times more evaluations than GP-based methods to achieve similar goals (at batch size 1; see Tables 3–5, available as supplementary data at *Bioinformatics* online).
- **More complex Bayesian models** (SBO_ANN_PV, SBO_POLY_PV, and SMART_BO) did not outperform simpler GP surrogates in these test environments, suggesting that their added complexity may not be justified in low-to-moderate dimensional biological optimization problems.

These findings underscore the practical value of using simple, robust and sample-efficient methods like GP-based Bayesian optimization to accelerate discovery in experimental biology. The summary statistics for the statistical analysis reinforce these results and can be found in Table 3, available as supplementary data at *Bioinformatics* online in the “Additional Results” section, available as supplementary data at *Bioinformatics* online.

### 6.4 Changing problem difficulty results

Results from these harder settings show clear trends. In the Hide-the-Label competition with random forest, neural network, and Bayesian neural network surrogates (Hard Mode, hidden fraction = 95%), GP-based Bayesian optimizers (BO_GP_EI and SBO_GP_PV) continued to perform best, often finding the target in 16–70 steps (batch size 1; see Table 4, available as supplementary data at *Bioinformatics* online for the full Hard Mode Hide-the-Label summary statistics). Their advantage over other methods was reduced compared to the standard setting, but they remained the most data-efficient. By contrast, more complex Bayesian methods such as SBO_ANN_PV and SMART_BO performed poorly, typically requiring 50–90 steps or plateauing near 85–90 steps, close to the levels of classical design strategies. These results suggest that increasing surrogate model complexity via deeper architectures (SBO_ANN_PV) or adaptive ensemble mechanisms (SMART_BO) did not improve robustness when the assumptions of smoothness were violated. Classical DOE methods (factorial, composite, Box–Behnken) and heuristic search strategies performed even worse, generally needing 80–120 steps or more, while random search typically needed to reveal roughly half of the hidden pool before locating the target.

In the Open Race experiments, where optimizers had access to the full random forest, neural network, or Bayesian neural network objective, the same general pattern held. GP-based methods again pulled ahead early and maintained their advantage. Some DOE strategies performed slightly better in this setting: factorial designs occasionally found strong intermediate values thanks to their space-filling construction, but could not exploit those lucky early observations because subsequent batches follow the fixed pre-planned grid—a characteristic plateau signature of non-adaptive methods. Non-GP Bayesian methods again failed to adapt to the noise and discontinuity, and random or direct search methods remained ineffective.

These results reinforce two lessons: (i) the strong inductive biases of GP models remain effective even when smoothness assumptions are violated, and (ii) increasing surrogate *architectural* complexity (deeper networks, ensembles) does not by itself guarantee robustness in low-data regimes—added capacity helps only when it matches the data structure (e.g. heteroskedastic noise models or discontinuity-aware kernels), a direction we flag for future work. Overall, the harder benchmarks provide a more realistic testbed for comparing optimizers in biological contexts. Additional results for Hide-the-Label, Open Race, and their harder versions can be found in the “Additional Results” section, available as supplementary data at *Bioinformatics* online. The summary statistics for the statistical analysis reinforce these results and can be found in Tables 4 and 5, available as supplementary data at *Bioinformatics* online in the “Additional Results” section, available as supplementary data at *Bioinformatics* online.

## 7 Practical roadmap for researchers

The benchmarking framework developed in this study provides practical guidance for designing future media optimization campaigns. Despite the dominance of adaptive modeling approaches in these settings, it is important to emphasize the importance of combining methods such as Bayesian optimization with the careful use of controls traditionally emphasized in DOE. Although GP-based Bayesian optimization methods often achieve strong sample efficiency, they rarely incorporate explicit controls such as replicates, center points, or reference formulations that are critical for detecting plate effects, batch heterogeneity, or systematic experimental drift. In practice, DOE-style design elements remain essential for anchoring optimization runs against these sources of variability. By contrast, Bayesian optimization tends to repeatedly sample uncertain or nearby points as a hedge against noise, which improves model robustness but does not substitute for true controls. Researchers are therefore advised to embed controls within Bayesian optimization loops to ensure that model-driven exploration is interpretable and biologically reliable.

An analysis was conducted in the “Changing number of initial points provided” section, available as supplementary data at *Bioinformatics* online, showing that even when only 1% of the points are initially revealed and the remaining 99% are hidden, the same Bayesian methods such as BO_GP_EI and SBO_GP_PV end up outperforming the other optimizers. Often in machine learning, a rule of thumb is to have at least 10 times as many datapoints (rows in the data table) as covariates (columns) (so if you have 7 ingredients, you should have at least 70 rows to build a good model) (Babyak 2004). However, this rule of thumb is subject to many factors including the quality of the data, the columns, the rows, and noise, and the complexity of the function mapping covariates to the label. Despite this, Bayesian optimization methods often adjust to account for this: more uncertain regions are sampled more often, and if the function does not have enough points to build a strong surrogate model, the algorithm naturally places greater emphasis on exploration. Beyond optimizer selection, the benchmarking framework itself can be directly applied as a pre-experimental planning tool. The *Hide-the-Label* protocol provides a realistic simulation of resource-constrained discovery, forcing optimizers to succeed with limited and sequential feedback, while the *Open Race* protocol isolates pure search efficiency under fixed budgets. Together, these complementary views allow researchers to anticipate the strengths and weaknesses of competing algorithms under different laboratory conditions. To make these tools accessible, the accompanying code repository provides implementations of both protocols, as well as routines for debiasing a dataset to correct for sampling artifacts and heterogeneity. By leveraging these resources, practitioners can stress-test optimization strategies in silico before committing costly laboratory assays, systematically compare Bayesian and DOE approaches under controlled conditions, and design hybrid campaigns that combine adaptive exploration with robust experimental controls. Taken together, these practices encourage a balanced strategy: use Bayesian optimization for adaptive sampling, DOE designs for controls and baselines, and simulation-based benchmarking for stress-testing—all of which help ensure robust, reproducible, and cost-effective experimental discovery.

## 8 Conclusion

This study introduced a principled framework for benchmarking optimization algorithms, tailored to the practical constraints of experimental biology. The results demonstrate that in typical, label-limited environments simulated by the Hide-the-Label protocol, simple Gaussian Process-based Bayesian optimization (BO) methods offer superior sample efficiency. This suggests that the strong inductive biases of GPs, such as smoothness, align well with the response surfaces commonly encountered in cell media optimization. However, the findings also highlight that no single optimizer is universally dominant. In more data-rich settings like the Open Race, advanced optimizers showed competitive performance, and even classical Design of Experiments methods displayed robustness under certain conditions. The ideal choice of algorithm is therefore critically dependent on the task structure, data availability, and experimental budget.

The primary contribution of this work is a rigorous, data-driven methodology that enables practitioners to select an appropriate optimization strategy *in silico* before committing to costly and time-consuming laboratory experiments. The Hide-the-Label protocol provides a realistic simulation of the iterative, resource-constrained nature of scientific discovery, and the accompanying modular codebase allows for easy integration into existing research workflows. By using this framework as a pre-experimental simulation tool, researchers can make more informed, context-aware decisions that maximize the efficiency of their experimental campaigns.

Several limitations should be noted. No publicly available datasets exist for edible cell lines (e.g. beef myocytes), so biologically similar proxy lines were used; direct laboratory validation remains a valuable next step. The synthetic data generation step—fitting a GP, RF, NN, or BNN surrogate—may partially smooth fine-grained plate-to-plate variability, so the benchmark captures the aggregate biological signal but may underrepresent highly localized artifacts; incorporating explicit plate-effect or batch-effect models would enhance fidelity. Future directions include transfer learning—reusing a model trained on one cell line to accelerate optimization of another (Narayanan *et al.* 2025)—broader algorithm classes (reinforcement learning, trust-region methods), and application to other expensive-evaluation domains such as materials science and chemical synthesis. The research community is encouraged to contribute additional datasets to expand the benchmark.

## Supplementary Material

btag607_Supplementary_Data

## Data Availability

No primary data were generated by this study. The six benchmark datasets derive from previously published open-access studies (doi:10.1002/elsc.202300005; doi:10.1038/s41540-023-00284-7; doi:10.1038/s42003-019-0296-7) and, reformatted into a common schema with full provenance notes, are distributed with the accompanying code at https://doi.org/10.5281/zenodo.21501659. Benchmark results are regenerated deterministically by the scripts in that archive rather than deposited as static files.
