# Supplementary material for: A data-driven evaluation of optimization techniques in cell culture media
Source: Bioinformatics. 2026 Aug 19;42(9):btag607. doi: 10.1093/bioinformatics/btag607 (PMC13529365; doi:10.1093/bioinformatics/btag607)
Supplement: Supplementary file 1 [file btag607_supplementary_data.pdf]

Bayesian Optimization Methodology (Extended)

This section provides the full treatment of Bayesian optimization (BO) methodology summarized in the main text. BO seeks $x^* = \arg\max_{x\in\mathcal{X}} f(x)$, where $\mathcal{X} \subset \mathbb{R}^d$ is the input domain, from noisy observations $y = f(x) + \varepsilon$, $\varepsilon \sim \mathcal{N}(0, \sigma_n^2)$.

Surrogate Model: The Gaussian Process

The unknown objective function $f(x)$ is modelled using a **Gaussian Process (GP)**, a non-parametric prior over functions. A GP is fully specified by a mean function $m(x)$ and a covariance (kernel) function $k(x, x')$, written as $f \sim \mathcal{GP}(m(x), k(x, x'))$. The kernel encodes prior assumptions about the function's properties, such as smoothness. Common choices include the Radial Basis Function (RBF) kernel for infinitely smooth functions and the Matérn family of kernels, which can model functions with less smoothness—often a more realistic assumption for biological systems (Rasmussen and Williams, 2006; Stein, 1999).

Given a set of observations $\mathcal{D}_t = \{(x_i, y_i)\}_{i=1}^t$, the GP provides a posterior distribution for the function value at any new point x_*. This posterior is a normal distribution, $f(x_*) \mid \mathcal{D}_t \sim \mathcal{N}(\mu_t(x_*), \sigma_t^2(x_*))$, where $\mu_t(x_*)$ is the predicted mean and $\sigma_t^2(x_*)$ is the predictive variance, which quantifies the model's uncertainty at that point.

Acquisition Functions

The acquisition function, $\alpha_t(x)$, uses the posterior mean and variance from the GP to quantify the utility of evaluating a candidate point x. The next point to be evaluated, x_{t+1}, is selected by maximizing this function:

$$x_{t+1} = \arg\max_{x\in\mathcal{X}} \alpha_t(x).$$

This step translates the exploration–exploitation trade-off into a well-defined optimization problem. Prominent acquisition functions include:

- **Expected Improvement (EI):** Calculates the expected improvement over the best value observed so far, $f(x^+)$:

$$\begin{aligned} \mathrm{EI}(x) =& (\mu_t(x) - f(x^+))\Phi(z) + \sigma_t(x)\phi(z), \\ &\text{where } z = \frac{\mu_t(x) - f(x^+)}{\sigma_t(x)}. \end{aligned} \tag{1}$$

  with $\Phi(\cdot)$ and $\phi(\cdot)$ being the CDF and PDF of the standard normal distribution.
- **Upper Confidence Bound (UCB):** Selects points with a high upper confidence bound on their value, balancing the predicted mean with uncertainty via a trade-off parameter κ:

$$\mathrm{UCB}(x) = \mu_t(x) + \kappa\sigma_t(x).$$

The Bayesian Optimization Loop

The full BO process is an iterative loop that adaptively refines the search for the optimum:

1. **Initialization:** Begin with a small set of initial data points, often generated via random sampling or a space-filling design like Latin Hypercube Sampling (Cosenza et al., 2022).
2. **Model Fitting:** Fit a GP surrogate model to all currently available data $\mathcal{D}_t$.
3. **Acquisition Maximisation:** Find the next point to evaluate, x_{t+1}, by maximizing the chosen acquisition function over the domain $\mathcal{X}$.
4. **Function Evaluation:** Perform the physical experiment at x_{t+1} to obtain a new noisy observation, y_{t+1}.
5. **Update:** Augment the dataset with the new point, $\mathcal{D}_{t+1} = \mathcal{D}_t \cup \{(x_{t+1}, y_{t+1})\}$, and repeat from Step 2 until a stopping criterion is met.

This closed-loop process allows BO to efficiently navigate the search space and often locate near-optimal solutions in a fraction of the experiments required by traditional methods.

Design of Experiments Methods (Non-Bayesian Optimization)

To complement Bayesian optimization strategies, a suite of non-Bayesian optimization methods was implemented based on classical and modern design of experiments (DOE) principles to compare against the Bayesian optimization models.

The selected non-Bayesian strategies fulfill three complementary roles in the evaluation framework:

- **Diverse exploration without strong priors.** DOE designs generate *a priori* fixed sets of trials that are orthogonal (factorials), rotatable (CCD), or space-filling (LHS), whereas evolutionary/swarm optimizers adapt online via simple stochastic rules rather than a learned surrogate. Taken together, they span uniform, stratified methods that enable probing of the objective landscape with no distributional or smoothness assumptions.
- **Baselines and optimistic upper bounds for Bayesian methods.** Because DOE points are chosen independent of observed responses, they form an unbiased baseline for measuring the marginal benefit of closed-loop, acquisition-driven sampling. Conversely, black-box optimizers that are allowed to query the true synthetic function directly—yet remain free of surrogate-model error—approximate the *best possible* outcome reachable within the same evaluation budget. Bayesian optimizers should therefore fall between these two extremes if the modelling assumptions are reasonable.
- **Stress-testing under non-ideal conditions.** Real laboratory data often deviate from the Gaussian, homoscedastic, and smooth function assumptions built into Gaussian-process (GP) surrogates—e.g. abrupt viability cliffs, heteroskedastic measurement noise, or discrete formulation constraints. The non-Bayesian suite operates agnostically to these limitations, giving a fairness check: if a Bayesian method under-performs relative to these baselines, poor prior/specification rather than evaluation budget is the likely culprit.

Collectively, the DOE and evolutionary/swarm procedures thus serve as a *dual bracket*: they set a conservative lower bar (uninformed design) and an optimistic upper bar (oracle-guided black-box search) against which the incremental value of Bayesian active learning can be quantitatively judged.

Experimental Design Methods

Six classical design-of-experiments (DOE) strategies were implemented, spanning screening, response surface, and space-filling paradigms. In this work, no additional libraries beyond SciPy and scikit-learn were used in implementing the proposed

methods, primarily because custom implementations were required to accommodate the specific experimental settings. However, several established libraries exist for implementing classical design-of-experiments methods, such as pyDOE2, DOEpy, and OpenDOE, which provide standardized routines for generating factorial, fractional factorial, response surface, and other experimental designs. The DOE methods are as follows:

- **Full Factorial Design** Enumerates every possible combination of factor levels (e.g. 2^k for k two-level factors). *Parameters*: number of factors, levels per factor. *Strengths*: exhaustive coverage yields unbiased estimates of all main effects and interactions up to the full order represented. *Limitations*: run count grows exponentially ($\mathcal{O}(L^k)$) and becomes impractical beyond ~ 7 factors at three levels each. Consequently, it serves mainly as a gold-standard reference for low-dimension cases.
- **Fractional Factorial Design** Selects a resolution-controlled subset (e.g. 2^{k-p}) of the full factorial table. *Key concept*: defining relation that aliases higher-order interactions with each other while keeping targeted effects unconfounded. *Typical use*: early-stage screening where main effects dominate and two-factor interactions are assumed negligible. *Trade-off*: 65–90 % run-count reduction at the cost of partial aliasing, which must be checked against subject-matter knowledge.
- **Plackett–Burman Design** An orthogonal array of $N = 4m$ runs ($m \geq 2$) capable of estimating main effects for up to $N-1$ factors with each factor appearing at two levels. *Screening focus*: intentionally sacrifices interaction estimation to minimize runs. *Caveat*: If strong two-factor interactions exist, main-effect estimates are biased, so follow-up augmentation is recommended once significant factors are identified.
- **Central Composite Design (CCD)** Combines a two-level factorial (or fractional factorial), $2k$ axial "star" points at radius α, and n_c center replicates. *Goal*: fit a second-order (quadratic) response-surface model with rotatability and near-optimal prediction variance in the design region. *Variants*: circum-rotatable, face-centered ($\alpha = 1$), or inscribed depending on region-of-interest constraints. *Cost*: total runs $N = 2^k + 2k + n_c$; grows linearly with k after the initial factorial core.
- **Box–Behnken Design** Uses mid-points of edges and the design center; never requires all factors simultaneously at their extremes, making it safer for biological media experiments where extreme pH or osmolarity can kill cells. *Efficiency*: needs $\frac{k(k-1)}{2} \times 2 + n_c$ runs, typically 20–40 % fewer than CCD for $k \geq 4$. *Limitation*: cannot estimate pure cubic terms without augmentation.
- **Latin Hypercube Sampling (LHS)** A stratified, space-filling scheme in which each of the k factors is divided into N equiprobable strata and one sample is drawn from each stratum, then the k univariate samples are randomly paired. *Advantage*: provides nearly uniform marginal coverage with only $N = k$ runs in its minimal form, making it popular for surrogate modelling and emulator training. *Enhancements*: maximin and orthogonal LHS further spread points to maximize the minimum pairwise distance or balance correlation.

Direct Optimization Methods

To bound best-case performance, one direct black-box optimizer that iteratively evaluates the true synthetic objective was benchmarked without surrogate-model bias:

- **Differential Evolution (DE)** Maintains a population $\{\mathbf{x}_i\}_{i=1}^{P}$ and generates trial vectors via scaled differences, $\mathbf{v}_i = \mathbf{x}_{r_1} + F(\mathbf{x}_{r_2} - \mathbf{x}_{r_3})$, followed by binomial crossover and greedy selection. *Hyper-parameters*: mutation factor $F \in [0.4, 1]$, crossover rate C_r, population size $P \approx 10\,k$. *Notable feature*: self-adaptive exploration of non-convex, non-differentiable spaces with few control knobs.

Baseline Strategy

A **random sampling** method has been included to establish a performance baseline. It selects query points uniformly at random from the pool of candidates without considering any prior observations.

Power Analysis

Choosing the Number of Competitions and Synthetic Datasets

Statistical power was quantified via a two-factor Monte Carlo simulation. For each candidate configuration, the following are varied:

$n_{\mathbf{comp}}$	number of optimizer "competitions" per synthetic dataset,
$n_{\mathbf{syn}}$	number of independently sampled synthetic datasets.

Each repetition consisted of the following: (1) sampling a synthetic response surface; (2) simulating the outcome of a hide–the–label protocol with n_{comp} paired optimizer evaluations; and (3) recording whether a statistically significant winner emerged under the assumed ground-truth performance ranking. To reduce computational cost, the entire optimization process was not rerun in each simulation. Instead, outcome statistics consistent with a fixed performance gap were directly generated, modelling what the test would observe if one optimizer were consistently better than its competitors.

To simulate ground-truth superiority, one optimizer was explicitly designated as better (consistently exhibiting lower regret on synthetic surfaces) and measured how often this true superiority was detected using a one-sided performance test with tolerance threshold $\epsilon = 10^{-3}$. Thus, power in this context represents the likelihood that a given design configuration is sensitive enough to reliably identify the superior optimizer across variability in surfaces and sampling.

This was repeated for a grid of design sizes (see Figure 1), and for each grid point statistical power $\hat{\pi}$ was estimated along with a 95% confidence interval using Agresti–Coull adjusted Wald estimates.

Interpreting the results.

The "High" configuration ($n_{\text{comp}} = 25$, $n_{\text{syn}} = 15$) achieved power of 99.2% with a ±6.2% confidence interval, completing in ~ 3 minutes on the reference hardware. This met the target criteria while remaining computationally efficient. Larger designs yielded diminishing returns in precision relative to runtime.

Power Analysis Detailed Comparison
(Recommended configuration highlighted in gold)

Configuration	n_competitions	num_synthetic_data	Total	Power	CI Width	Runtime
Minimal (budget)	10	5	50	29.0%	±19.9%	0.4m
Low	15	8	120	67.6%	±11.6%	1.0m
Moderate	20	10	200	89.6%	±8.7%	1.7m
**High**	**25**	**15**	**375**	**99.2%**	**±6.2%**	**3.1m**
Very High	30	20	600	100.0%	±4.8%	5.0m
Maximum	40	25	1000	100.0%	±3.7%	8.3m

Excellent (≥90% power, ≤±5% CI) Poor (<80% power, >±7.5% CI)
Good (80-90% power, ±5-7.5% CI) Recommended Configuration

Fig. 1. Comparison of statistical power, confidence–interval width, and wall-clock runtime across candidate power analysis configurations. The gold-highlighted row indicates the minimal configuration that satisfies the criteria: $\hat{\pi} \geq 0.90$.

Final sample size and configuration.

A conservative setting of $n_{\text{comp}} = 30$ and $n_{\text{syn}} = 30$ (i.e. 900 competition runs) was adopted. All benchmarks were executed in Python 3.10 on a **MacBook Pro** (2.4 GHz Quad-Core Intel Core i5, 16 GB RAM), with total runtime of approximately **4 hours** for all 900 runs.

Choosing $n = 30$ aligns with common parametric assumptions (via the Central Limit Theorem) and provides sufficient resolution for statistical comparisons between optimization strategies. This configuration yields estimated power near 100% and a confidence interval width below 5%, while staying within practical runtime limits.

Sampling Density Within Each Synthetic Dataset

The prior subsection established how many independent optimization runs are required for statistical confidence. The analysis examined how many query points (n_{pts}) each synthetic dataset must contain in order to reliably detect influential input factors under realistic conditions.

To simulate this, synthetic response surfaces were constructed based on a linear-in-parameters model with eight input variables—some assigned non-zero effect sizes ($\beta = 1$), and others set to zero. Surrogate models were then fitted to noisy samples drawn from each synthetic function, and the empirical power was computed to reject the null hypothesis $H_0 : \beta = 0$ at significance level $\alpha = 0.05$.

This procedure was repeated for each candidate sampling density $n_{\text{pts}} \in \{20, 25, \ldots, 200\}$ using 1 000 Monte Carlo replicates per setting. Importantly, realistic complexities including multicollinearity (correlated inputs), non-linearity, and heteroscedasticity (input-dependent noise) were introduced to match the statistical structure of real laboratory data.

The resulting power curves (Figure 2) show that for effect sizes $\beta = 1$, the probability of correctly identifying active variables exceeds 90% once $n_{\text{pts}} \gtrsim 150$. Meanwhile, null factors maintain Type I error rates near the nominal 5% level, indicating good calibration.

Final sampling density choice.

To ensure robust detectability of signal and maintain parity with earlier design choices, $n_{\text{pts}} = 200$ was selected for each synthetic dataset. This provides power ≥ 0.95 to detect strong factors as shown in Figure 2, ensures correct rejection rates for nulls, and remains computationally feasible within the total budget.

With 200 points per dataset, 30 datasets, and 30 competitions per dataset, the final experimental allocation totals $200 \times 30 \times 30 = 180{,}000$ function evaluations. This design enables principled discrimination between strong and weak optimizers and supports generalization across variable response landscapes.

This two-tiered power analysis thus justifies both the number of evaluations *across* datasets and the density of sampling *within* each dataset used in the main experiments (Methods section).

Statistical Analysis Framework (Extended)

This section provides the full mathematical details of the statistical testing framework summarized in the main text.

Response Variables

In Hide-the-Label (HtL) the response variable was the number of queries to reveal the global maximum, denoted $t^*(z, c, j)$, where z indexes the synthetic dataset, c the competition (masking configuration), and j the optimizer. In Open Race (OR), the response was $b(z, c, j)$, the *best* function value found within a fixed budget of J_q queries ("b" abbreviates "best"; "HtL" and "OR" are used as superscripts to label protocol-specific pairwise differences):

$$b(z, c, j) = \max\{f(x_1^{(0)}), \ldots, f(x_n^{(0)}), f(x^{(1)}), \ldots, f(x^{(J_q)})\},$$

where $x_1^{(0)}, \ldots, x_n^{(0)}$ are the shared warm-start points for competition c. In both settings the fundamental unit of analysis is the *instance* $i = (z, c)$, observed across all optimizers.

Omnibus and Pairwise Tests

The non-parametric Friedman test was first applied to test

$$H_0 : \text{ All optimizers have equal performance distributions}$$

against the alternative of systematic differences, treating instances i as blocks and optimizers as treatments. The Friedman test operates on within-block ranks and is robust to skew, outliers, and monotone transformations.

When Friedman indicated global differences ($\alpha = 0.05$), pairwise comparisons were conducted. For each optimizer pair

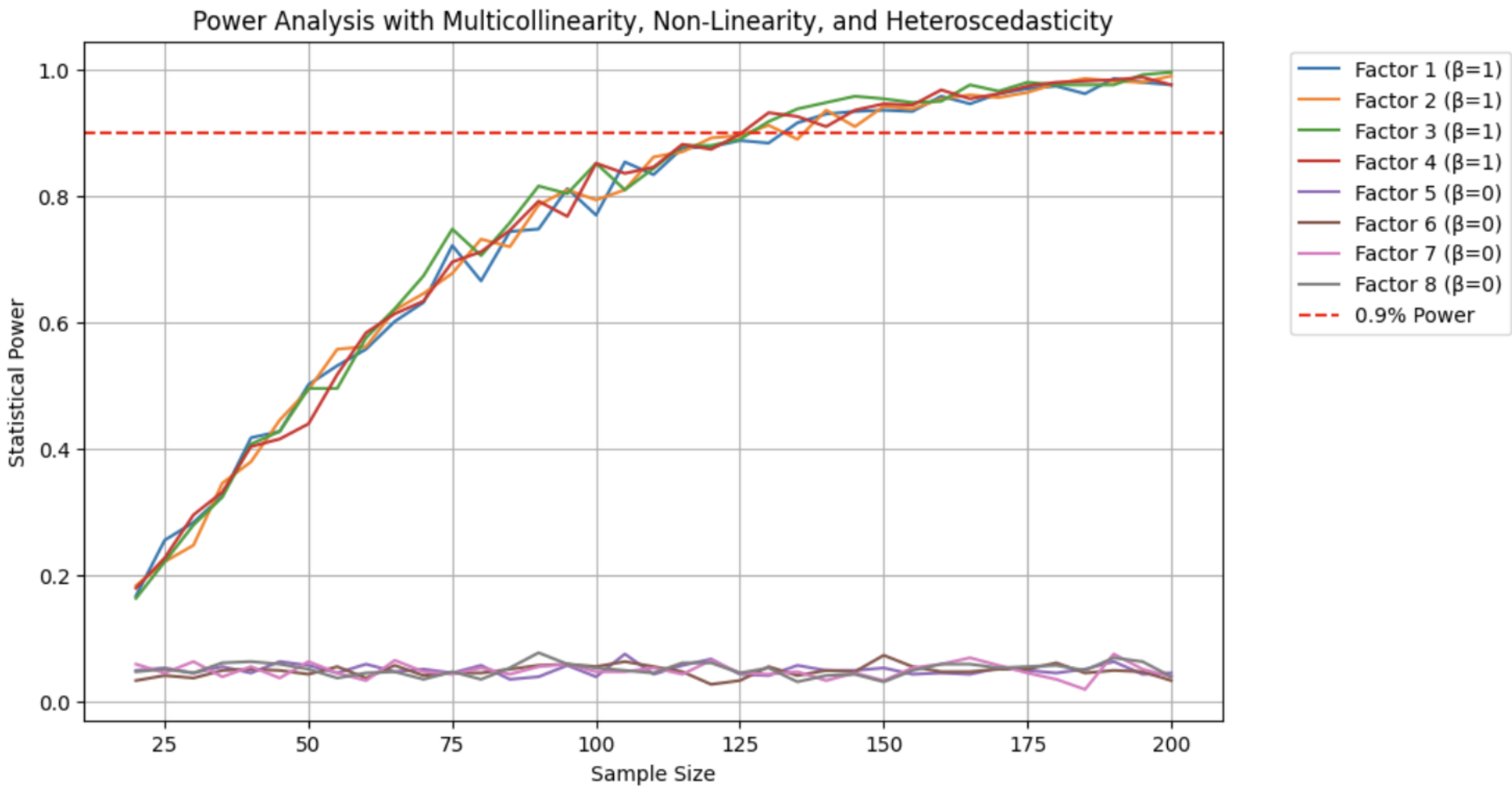

Fig. 2. Power curves for detecting active and null factors as a function of per-dataset sample size. Solid lines represent variables with true effect size $\beta = 1$, muted lines represent noise-only variables ($\beta = 0$). The horizontal dashed line indicates the power target of 0.9.

(a, b) the per-instance difference was formed

$$\Delta_i^{\mathrm{HtL}} = t^*(z, c, a) - t^*(z, c, b), \quad \Delta_i^{\mathrm{OR}} = b(z, c, a) - b(z, c, b).$$

The null $\mathbb{E}[\Delta_i] = 0$ was tested using a two-sided sign-flip permutation test, with signs permuted jointly within each dataset z to preserve correlation among competitions. Family-wise error was controlled by the Holm step-down procedure.

Effect Sizes

For each pair, the Hodges–Lehmann median paired difference $\hat{\theta} = \mathrm{median}\{\Delta_i\}$ was reported, interpreted as typical attempts saved (HtL) or typical value gain (OR), with 95% bootstrap confidence intervals obtained by resampling datasets z as the clustering unit.

Dataset-Level Sensitivity Check

As a conservative check, results were aggregated within datasets: for each optimizer j,

$$\tilde{t}^*_{zj} = \mathrm{median}_c\, t^*(z, c, j), \quad \tilde{b}_{zj} = \mathrm{median}_c\, b(z, c, j).$$

The Friedman and pairwise tests were then repeated on the resulting $Z \times J$ tables, treating datasets as independent units.

Power Analysis Synthetic Function

The synthetic function used for the power analysis was a linear-in-parameters model with eight input variables, of which a subset were assigned non-zero effect sizes ($\beta = 1$) and the remainder set to zero. Realistic complexities including multicollinearity among inputs, mild non-linearity, and heteroscedastic observation noise were added to match the statistical structure of real cell-culture datasets. Surrogate models were fitted to noisy samples drawn from this function and power was estimated as the empirical probability of correctly rejecting $H_0 : \beta = 0$ at $\alpha = 0.05$ over 1,000 Monte Carlo replicates per design configuration.

Changing problem difficulty

To ensure that the benchmarking framework fairly evaluates optimizers under realistic biological conditions, a comprehensive mechanism was implemented to systematically increase the difficulty of the optimization tasks. This approach addresses two key concerns: (1) the unfair advantage that Gaussian process (GP)-based optimizers may have in GP-generated environments, and (2) the need to model complexities present in real-world biological systems.

Removing the GP Advantage

Earlier benchmarks have often relied on Gaussian process (GP) surrogates to generate synthetic objective functions. While convenient, this design implicitly favours GP-based Bayesian optimizers by aligning the benchmark surface with their underlying assumptions of smoothness and stationarity. To address this limitation, the surrogate model class was expanded to include alternative function generators. Random forests were introduced to produce piecewise-constant landscapes that break differentiability, neural networks were used to capture complex nonlinear dependencies, and Bayesian neural networks (BNNs) were added to provide calibrated predictive uncertainty alongside non-stationary surfaces. Together, these alternatives ensure that the evaluation setting does not systematically privilege GP-based methods.

Difficulty Factors

Beyond the choice of surrogate, a set of configurable difficulty parameters was introduced to stress-test optimizers under more realistic and heterogeneous conditions. Noise was added

to function outputs, including both homoscedastic Gaussian noise and input-dependent heteroscedastic noise to simulate variable experimental error. Multi-modality was induced by embedding multiple local optima within the search space, forcing optimizers to navigate away from deceptively good regions. Finally, discontinuities were included to mimic threshold-like behaviour or sudden phase shifts, producing landscapes that violate standard smoothness assumptions. The reported Hard Mode experiments combine heteroskedastic noise with bimodal perturbations. Homoskedastic noise and discontinuities are also implemented in the codebase as optional difficulty factors for extended testing.

Implementation in Benchmarks

These difficulty factors were incorporated differently across benchmark settings. In the *Hide-the-Label* competition, the surrogate itself was augmented during dataset generation, meaning the optimizers encountered noisy, multi-modal, or discontinuous label structures. By contrast, in the *Open Race* benchmark, the same factors were applied stochastically at query time: the underlying objective function is fixed throughout, but each evaluation returns a noisy or transformed observation (e.g. with additive noise $\varepsilon \sim \mathcal{N}(0, \sigma^2)$ applied at the moment of querying). The objective surface itself does not change during optimization; rather, optimizers receive stochastically corrupted feedback at each query, mirroring the irreducible measurement noise of real laboratory experiments. This distinction ensures that both limited-feedback and fully transparent regimes reflect the same biological and experimental challenges, albeit in structurally different ways.

Biological Justification

The inclusion of these difficulty factors is motivated directly by biological experimentation. Laboratory measurements are rarely noise-free and often display heteroscedastic variance, as error rates depend on both reagent concentrations and assay conditions. Many biological systems also display multi-stability, with multiple distinct optima or stable states that cannot be captured by unimodal surrogates. Similarly, discontinuities are commonplace, arising from regulatory thresholds, phase transitions, or binary cellular switches. Embedding these properties into the benchmarks produces a testbed that more closely mirrors the realities of experimental biology, thereby increasing the generalizability and relevance of optimizer comparisons.

Algorithms

This section provides the algorithms mentioned in the paper. The Hide-the-Label and Open Race protocols described in the paper are divided into two sections. Algorithm 1 describes the hiding mechanism introduced, which is then used in Algorithm 2 for implementing the full Hide-the-Label protocol as an optimizer benchmark. Similarly, Algorithm 3 introduces the Open Race mechanism, which is then used in Algorithm 4 for implementing the full Open Race protocol as an optimizer benchmark.

Algorithm 1 Hide-the-Label protocol

Require: Dataset $\mathcal{D} = \{(x_i, y_i)\}_{i=1}^N$, hidden fraction $\alpha \in (0,1)$, optimizer $\mathcal{O}$, batch size B
$labels = \{y_i\}_{i=1}^N$, $covariates = \{x_i\}_{i=1}^N$
Identify target point $(\mathbf{x}^*, y^*) = \arg\max_{(\mathbf{x},y)\in\mathcal{D}} y$
Randomly Split $labels$ into visible set $labels_{vis}$ and hidden set $labels_{hid}$ such that $|labels_{hid}| = \alpha N$
Ensure $y^* \in labels_{hid}$
$t \leftarrow 0$
repeat
  $t \leftarrow t + 1$
  $batch = \mathcal{O}(covariates, labels_{vis}, B)$
  $labels_{vis} \leftarrow labels_{vis} \cup labels(batch)$
  $labels_{hid} \leftarrow labels_{hid} \setminus labels(batch)$
until $y^* \in labels_{vis}$
return $t^* = t$ (number of steps to find target)

Algorithm 2 Optimizers Race Benchmark - with Hide-the-Label protocol

Require: Dataset $\mathcal{D} = \{(x_i, y_i)\}_{i=1}^N$, hidden fraction $\alpha \in (0,1)$, batch size B, optimizers $\mathsf{Optimizers} = [\mathcal{O}_1, \ldots, \mathcal{O}_J]$, number of synthetic datasets Z, number of competitions per optimizer C, and the feature space (input domain) from which candidate formulations can be sampled $\mathcal{X}$
Let $\mathcal{T}$ be the chosen model type (e.g. Gaussian process model)
Train model $\mathcal{M} \leftarrow \textsc{TrainModel}(\mathcal{T}, \mathcal{D})$
Initialize Results $\leftarrow [\,]$
for $z = 1$ to Z **do**
  Randomly Sample candidate inputs $D' \subset \mathcal{X}$
  Generate synthetic labels $S_z \leftarrow \mathcal{M}(D')$
  Initialize OptimizerResults $\leftarrow [\,]$
  for $j = 1$ to J **do** ▷ Loop over optimizers
    Initialize Trials $\leftarrow [\,]$
    for $c = 1$ to C **do** ▷ Repeat competition C times
      output $\leftarrow \textsc{HideTheLabel}(D', S_z, \alpha, \mathcal{O}_j, B)$
      Add output to Trials
    end for
    Add Trials to OptimizerResults
  end for
  Add OptimizerResults to Results
end for
return Results

Changing number of initial points provided

To assess how the amount of initial labelled data influences active learning outcomes, we compared optimization methods under two initialization regimes: a sparse setting (`hidden_fraction = 0.99`, approximately 42 revealed points on average) and a denser one (`hidden_fraction = 0.95`, approximately 105 revealed points on average). This allowed us to test the robustness of each method when starting with limited versus moderately rich initial information. The reported revealed-point counts are averages taken across the six experimental datasets and 30 synthetic replicates per dataset. Because the candidate pools differ in size and structure across datasets, the ratio of revealed points between the two regimes is not exactly 5 : 1 but instead reflects this dataset-level averaging.

Across both evaluation protocols—*Hide-the-Label* and *Open Race*—Bayesian optimization methods consistently outperformed others, regardless of the hidden fraction. In the *Hide-the-Label* protocol, `BO_GP_EI` was especially efficient, requiring a mean of only 1.25 steps to reach the target in the 0.99 setting, and maintaining top-tier performance with more initial data (mean 7.95 steps in the 0.95 setting). Similarly, in

Algorithm 3 Open Race protocol

Require: Function f, batch size B, optimizers $Optimizers = [\mathcal{O}_1, \ldots, \mathcal{O}_J]$, total number of points sampled S, number of initial random points R

Sample R initial points $\{x_i\}_{i=1}^{R}$ uniformly within the domain of f
$D_{\text{init}} \leftarrow \{(x_i, f(x_i))\}_{i=1}^{R}$
ObservationList $\leftarrow [\,]$
for $j = 1$ to J **do** ▷ For each optimizer
  $D \leftarrow D_{\text{init}}$ ▷ Reset to initial observation
  for $s = 1$ to $\lfloor \frac{S}{B} \rfloor$ **do**
    $X_{\text{next}} \leftarrow \mathcal{O}_j(D, B)$ ▷ Optimizer selects next batch
    Evaluate $Y_{\text{next}} \leftarrow f(X_{\text{next}})$
    $D \leftarrow D \cup \{(X_{\text{next}}, Y_{\text{next}})\}$
  end for
  Append $\max_{(x,y)\in D} y$ to ObservationList ▷ Record best outcome
end for
return ObservationList

Algorithm 4 Optimizers Race Benchmark with Open Race protocol

Require: Dataset $\mathcal{D}$, batch size B, optimizer list Optimizers $= [\mathcal{O}_1, \ldots, \mathcal{O}_J]$, number of competitions C, total points per run S, number of initial random points R

Choose surrogate model type $\mathcal{T}$ (e.g. Random Forest, GP, Neural Network, or Bayesian Neural Network)
Train surrogate model: $\mathcal{M} \leftarrow \textsc{TrainModel}(\mathcal{T}, \mathcal{D})$
Define synthetic objective: $f(x) \leftarrow \mathcal{M}(x)$
Initialize CompetitionsList $\leftarrow [\,]$
for $c = 1$ to C **do**
  ObservationList $\leftarrow \textsc{OpenRaceProtocol}(f, B, \text{Optimizers}, S, R)$
  Append ObservationList to CompetitionsList
end for
return CompetitionsList

the *Open Race* protocol, Bayesian methods such as BO_GP_EI and SBO_GP_PV achieved the best function values under both hidden fraction scenarios, with their relative rankings largely unaffected by the size of the initial labelled set.

Table 1 presents a detailed comparison of the top three optimization methods across both initialization regimes and evaluation protocols. The results reveal a striking pattern: GP-based Bayesian optimization methods (BO_GP_EI, SBO_GP_PV, and SBO_GP_EI_TRUNCDE) consistently occupy the top positions regardless of the hidden fraction setting. Table 2 provides a comprehensive statistical comparison of all 14 optimization methods across both hidden fraction settings. The top 3 performers in each setting are highlighted in bold, revealing that GP-based Bayesian methods dominate with BO_GP_EI (1.25 steps), SBO_GP_PV (1.59 steps), and SBO_GP_EI_TRUNCDE (1.61 steps) in the sparse HF=0.99 setting, and SBO_GP_PV (7.93 steps), BO_GP_EI (7.95 steps), and SBO_GP_EI_TRUNCDE (8.06 steps) in the denser HF=0.95 setting. These results clearly demonstrate that GP-based Bayesian approaches outperform classical Design of Experiments and baseline methods regardless of initialization conditions.

Crucially, increasing the number of initial labelled points did not substantially shift the performance hierarchy. GP-based Bayesian methods remained dominant, showing a notable insensitivity to initialization conditions. For instance, in the *Hide-the-Label* protocol, the three GP-based Bayesian methods (BO_GP_EI, SBO_GP_PV, and SBO_GP_EI_TRUNCDE) occupied the top three positions among optimizers evaluated on the full configuration set with hidden fraction 0.99, with mean steps ranging from 1.25 to 1.61, and maintained the same top three positions with hidden fraction 0.95 (7.93–8.06 steps). Across all optimizers, GP-based Bayesian methods as a category demonstrated consistent superiority over classical Design of Experiments and baseline approaches. In the *Open Race* protocol, BO_GP_EI and SBO_GP_PV alternated between the top two positions across both settings, demonstrating exceptional consistency.

This robustness implies that practitioners can expect reliable performance from these methods even when starting with minimal data, reducing the need for extensive upfront labelling. The consistency of these results reinforces Bayesian optimization as a highly dependable strategy across diverse active learning setups. Moreover, the stability of the performance hierarchy across hidden-fraction settings suggests that the adaptive nature of Bayesian optimization effectively compensates for limited initial information through intelligent exploration guided by uncertainty quantification.

Additional results

Friedman Test Results:

- Instance-Level: $\chi^2 = 129.78$, $p < 0.001$ (highly significant)
- Dataset-Level: $\chi^2 = 35.57$, $p < 0.001$ (highly significant)

Table 1. Top-3 Optimization Methods Across Different Initial Labelled Points

Protocol	Hidden Fraction	Avg Init. Points	Rank	Optimizer	Mean	Median
Hide-the-Label	0.99	42	1	BO_GP_EI	1.25	1.0
			2	SBO_GP_PV	1.59	1.0
			3	SBO_GP_EI_TRUNCDE	1.61	1.0
Hide-the-Label	0.95	105	1	SBO_GP_PV	7.93	3.0
			2	BO_GP_EI	7.95	3.0
			3	SBO_GP_EI_TRUNCDE	8.06	3.0
Open Race	0.99	N/A	1	SBO_GP_PV	3.42	1.04
			2	BO_GP_EI	3.41	1.06
			3	SBO_GP_EI_TRUNCDE	3.38	1.04
Open Race	0.95	N/A	1	BO_GP_EI	9.26	9.37
			2	SBO_ANN_PV	8.90	8.75
			3	DE_DIRECT	8.85	8.64

Note: For Hide-the-Label, lower values indicate better performance (fewer steps to find target). For Open Race, higher values indicate better performance (better function value found). Rankings include only optimizers evaluated across all six datasets and all 30 synthetic replicates (Count = 906).

Table 2. Performance Comparison Across Hidden Fractions (Hide-the-Label Protocol)

Category	Optimizer	HF = 0.99 (∼42 initial)			HF = 0.95 (∼105 initial)		
		Mean	Median	Std	Mean	Median	Std
Bayesian	SBO_GP_PV	**1.59**	1.0	3.05	**7.93**	3.0	15.23
	BO_GP_EI	**1.25**	1.0	2.16	**7.95**	3.0	15.53
	SBO_GP_EI_TRUNCDE	**1.61**	1.0	3.42	**8.06**	3.0	17.10
	SMART_BO	18.39	1.0	39.82	24.54	7.0	39.07
	SBO_ANN_PV	20.29	5.6	30.90	28.98	8.0	44.14
	SBO_POLY_PV	20.34	5.6	31.86	29.05	8.0	45.52
DoE	BOX_BEHNKEN	28.30	7.4	47.31	26.95	7.0	45.06
	FULL_FACTORIAL	33.12	9.5	51.02	31.54	9.0	48.59
	FRACTIONAL_FACTORIAL	33.69	9.5	51.67	32.09	9.0	49.21
	LATIN_HYPERCUBE	35.57	9.5	54.57	33.88	9.0	51.97
	CENTRAL_COMPOSITE	37.78	10.5	55.64	35.98	10.0	52.99
	PLACKETT_BURMAN	39.09	9.5	59.34	37.23	9.0	56.51
Baseline	DE_DIRECT	46.62	15.4	64.56	42.38	14.0	58.69
	RANDOM	48.53	14.5	63.07	42.38	14.0	58.69

Note: Lower values indicate better performance (fewer steps to find target). Top 3 performers in each setting shown in bold. HF = Hidden Fraction; higher values mean fewer initially labelled data points. GP-based Bayesian optimization methods consistently achieve top-tier performance across both initialization regimes.

Table 3. Summary Statistics - Regular Mode Open Race

Rank	Optimizer	Mean	Median	Std	Min	Max	Count	Avg Rank
1	BO_GP_EI	4.20	1.11	4.86	0.20	11.14	160	1.69
2	SBO_GP_PV	4.21	1.11	4.87	0.20	11.14	160	1.69
3	SBO_ANN_PV	3.93	0.96	4.60	0.14	10.83	160	4.59
4	SMART_BO	3.85	0.89	4.57	0.05	10.83	160	6.12
5	FULL_FACTORIAL	3.85	0.82	4.63	0.03	10.59	160	7.30
6	CENTRAL_COMPOSITE	3.78	0.78	4.52	0.04	10.38	160	7.68
7	LATIN_HYPERCUBE	3.78	0.89	4.52	-0.04	10.38	160	7.71
8	PLACKETT_BURMAN	3.75	0.77	4.48	0.02	9.84	160	7.85
9	BOX_BEHNKEN	3.74	0.83	4.45	0.02	10.04	160	7.88
10	FRACTIONAL_FACTORIAL	3.76	0.81	4.49	0.02	10.32	160	8.05
11	DE_DIRECT	3.67	0.74	4.36	0.05	9.76	160	8.88
12	RANDOM	3.67	0.74	4.36	0.05	9.76	160	8.88

Table 4. Summary Statistics - Hard Mode Hide-the-Label

Rank	Optimizer	Mean	Median	Std	Min	Max	Count	Avg Rank
1	SBO_GP_EI_TRUNCDE	45.50	40.00	34.37	1.00	169.00	540	4.36
2	BO_GP_EI	51.67	47.00	37.68	1.00	181.00	540	5.13
3	SBO_GP_PV	52.44	46.00	38.29	1.00	177.00	540	5.85
4	SBO_POLY_PV	64.64	51.50	52.41	1.00	190.00	540	6.18
5	SMART_BO	65.38	50.50	50.82	1.00	189.00	540	6.39
6	SBO_ANN_PV	78.81	70.00	54.59	1.00	190.00	540	7.46
7	BOX_BEHNKEN	88.85	83.50	56.14	1.00	190.00	540	8.21
8	PLACKETT_BURMAN	100.15	103.50	54.02	1.00	190.00	540	8.23
9	RANDOM	101.20	103.00	54.34	1.00	190.00	540	8.29
10	FULL_FACTORIAL	98.67	99.00	56.64	1.00	190.00	540	8.72
11	FRACTIONAL_FACTORIAL	100.55	102.50	49.93	1.00	190.00	540	8.83
12	CENTRAL_COMPOSITE	100.91	104.00	53.37	1.00	189.00	540	8.85
13	LATIN_HYPERCUBE	96.51	96.00	55.44	1.00	190.00	540	9.13
14	DE_DIRECT	101.20	103.00	54.34	1.00	190.00	540	9.38

Table 5. Summary Statistics - Hard Mode Open Race

Rank	Optimizer	Mean	Median	Std	Min	Max	Count	Avg Rank
1	BO_GP_EI	1.55	0.93	2.31	0.56	10.55	39	4.31
2	SBO_GP_PV	1.56	0.92	2.37	0.55	11.04	39	4.56
3	SBO_GP_EI_TRUNCDE	0.86	0.81	0.21	0.60	1.24	36	4.78
4	SBO_POLY_PV	0.84	0.80	0.21	0.54	1.25	36	5.28
5	SBO_ANN_PV	1.41	0.87	1.90	0.58	8.31	39	5.46
6	CENTRAL_COMPOSITE	1.36	0.66	2.17	0.24	9.17	39	7.59
7	FULL_FACTORIAL	1.33	0.66	2.20	0.34	9.37	39	7.92
8	PLACKETT_BURMAN	1.26	0.67	1.96	0.42	8.55	39	8.23
9	SMART_BO	1.29	0.65	2.09	0.21	9.29	39	8.62
10	LATIN_HYPERCUBE	1.28	0.65	2.10	0.28	8.89	39	9.13
11	RANDOM	1.20	0.61	1.95	0.20	8.70	39	10.33
12	BOX_BEHNKEN	1.33	0.61	2.47	0.21	12.84	39	10.38
13	FRACTIONAL_FACTORIAL	1.29	0.62	2.20	0.34	10.00	39	10.72
14	DE_DIRECT	1.22	0.60	2.11	0.25	10.00	39	11.82

Friedman Test Results:

- Instance-Level: $\chi^2 = 264.22$, $p < 0.001$ (highly significant)
- Dataset-Level: $\chi^2 = 36.17$, $p = 0.001$ (significant)

Friedman Test Results:

- Instance-Level: $\chi^2 = 166.56$, $p < 0.001$ (highly significant)
- Dataset-Level: $\chi^2 = 30.28$, $p = 0.007$ (significant)

Table 6. Top Pairwise Comparisons - Hard Mode Hide-the-Label

Optimizer A	Optimizer B	HL Median	P-Value	Adj. P-Value	Significant
RANDOM	SBO_GP_PV	8.00	< 0.001	< 0.001	✓
CENTRAL_COMPOSITE	BOX_BEHNKEN	4.00	< 0.001	< 0.001	✓
CENTRAL_COMPOSITE	SBO_GP_EI_TRUNCDE	5.00	< 0.001	< 0.001	✓
SBO_ANN_PV	CENTRAL_COMPOSITE	-2.00	< 0.001	< 0.001	✓
SBO_ANN_PV	PLACKETT_BURMAN	-2.00	< 0.001	< 0.001	✓
SBO_ANN_PV	DE_DIRECT	-4.00	< 0.001	< 0.001	✓
SMART_BO	SBO_GP_EI_TRUNCDE	6.00	< 0.001	< 0.001	✓

Table 7. Top Pairwise Comparisons - Hard Mode Open Race

Optimizer A	Optimizer B	HL Median	P-Value	Adj. P-Value	Significant
RANDOM	BO_GP_EI	-0.30	< 0.001	< 0.001	✓
SMART_BO	SBO_GP_PV	-0.19	< 0.001	< 0.001	✓
SBO_GP_EI_TRUNCDE	FULL_FACTORIAL	0.11	< 0.001	< 0.001	✓
SBO_GP_EI_TRUNCDE	DE_DIRECT	0.16	< 0.001	< 0.001	✓
SMART_BO	SBO_POLY_PV	-0.10	< 0.001	< 0.001	✓
SMART_BO	SBO_GP_EI_TRUNCDE	-0.08	< 0.001	< 0.001	✓
SBO_POLY_PV	LATIN_HYPERCUBE	0.16	< 0.001	< 0.001	✓
SBO_POLY_PV	BOX_BEHNKEN	0.13	< 0.001	< 0.001	✓

Optimizer Definitions and Implementation Details

All optimizers evaluated in this benchmark are summarized below. Acronyms follow the convention `TYPE_SURROGATE_ACQUISITION`, where applicable. Hyperparameters not listed here default to scikit-learn defaults; exact values and version pins are in the code repository.

Surrogate-Assisted Bayesian Optimizers

- `BO_GP_EI`: Standard Bayesian Optimization with a Gaussian Process surrogate (squared-exponential *RBF* kernel with `length_scale` = 1.0 and an additive `WhiteKernel` noise term with `noise_level` = 0.1; in the fast configuration used in this benchmark both hyperparameters are held fixed, with $\alpha = 10^{-6}$ and standardized inputs) and Expected Improvement (EI) acquisition:

$$\mathrm{EI}(x) = (\mu_t(x) - f(x^+))\Phi(z) + \sigma_t(x)\phi(z), \quad \text{where } z = \frac{\mu_t(x) - f(x^+)}{\sigma_t(x)}. \tag{2}$$

  with $\Phi(\cdot)$ and $\phi(\cdot)$ the CDF and PDF of the standard normal distribution, and $f(x^+)$ the best value observed so far.
- `SBO_GP_PV`: Surrogate-Based Optimization with the same GP surrogate as `BO_GP_EI` but with an *Upper Confidence Bound* (UCB) acquisition, $a_t(x) = \mu_t(x) + \kappa\,\sigma_t(x)$ with $\kappa = 1.0$, which trades off the GP posterior mean against its uncertainty. (The legacy "`_PV`" suffix is retained for backwards compatibility with earlier result files.)
- `SBO_GP_EI_TRUNCDE`: Same GP and EI acquisition as `BO_GP_EI`, but instead of running a continuous acquisition-function optimizer, EI is evaluated directly on the discrete pool of unrevealed candidate points and the candidate with the largest EI is selected. The legacy "`TRUNCDE`" suffix reflects an earlier design that used a truncated Differential Evolution (DE) optimizer; in the shipped implementation the DE refinement step is skipped because evaluating EI on all hidden candidates already yields the discrete maximum directly.
- `SBO_ANN_PV`: Surrogate-Based Optimization with a two-layer Artificial Neural Network (ANN) surrogate (`MLPRegressor` with hidden layers of widths 50 and 20, `max_iter` = 1000, and `early_stopping=True`); the candidate with the highest predicted value is selected at each step. The "`_PV`" suffix denotes the predicted-value selection rule.
- `SBO_POLY_PV`: Surrogate-Based Optimization with a degree-2 polynomial regression surrogate; the candidate with the highest predicted value is selected at each step. Serves as a simple parametric baseline for the surrogate-assisted category.
- `SMART_BO`: A custom adaptive BO variant designed for higher-dimensional problems. Inputs are projected into a low-dimensional random embedding (embedding dimension $\min(\max(3, \lfloor n_{\mathrm{obs}}/3 \rfloor), \lfloor d/2 \rfloor, 8))$ whose rows are importance-weighted by a correlation-and-variance score, so informative input dimensions receive larger projection weight. A GP is fitted in the embedded space, and an Expected Improvement acquisition is used with a $0.1\,\sigma(x)$ exploration bonus, $a(x) = \mathrm{EI}(x) + 0.1\,\sigma(x)$, evaluated only on candidates that lie within an adaptive trust region (initial radius 0.3, expanded or contracted based on recent improvement). This was developed to investigate whether added complexity beyond standard GP-EI would yield gains; results show it did not.

Direct Heuristic Optimizers

- `DE_DIRECT`: Runs scipy.optimize.differential_evolution (maxiter=10, popsize=5) directly on the true (synthetic) objective function with no surrogate model. Because the optimizer returns a continuous solution but the benchmark requires picking from a fixed candidate pool, the proposed point is snapped to the nearest unrevealed candidate (by Euclidean distance) before being submitted. Serves as an optimistic upper bound for heuristic search.

Classical Design-of-Experiments Methods

`FULL_FACTORIAL`, `FRACTIONAL_FACTORIAL`, `PLACKETT_BURMAN`, `CENTRAL_COMPOSITE` (CCD), `BOX_BEHNKEN`, and `LATIN_HYPERCUBE` (LHS): standard DOE strategies described in detail in

Table 8. Top Pairwise Comparisons - Regular Mode Open Race

Optimizer A	Optimizer B	HL Median	P-Value	Adj. P-Value	Significant
RANDOM	SBO_GP_PV	-0.21	< 0.001	< 0.001	✓
BO_GP_EI	BOX_BEHNKEN	0.23	< 0.001	< 0.001	✓
BO_GP_EI	LATIN_HYPERCUBE	0.21	< 0.001	< 0.001	✓
SMART_BO	SBO_ANN_PV	-0.05	< 0.001	< 0.001	✓
SMART_BO	DE_DIRECT	0.06	< 0.001	< 0.001	✓
FULL_FACTORIAL	PLACKETT_BURMAN	0.02	< 0.001	< 0.001	✓
SMART_BO	FRACTIONAL_FACTORIAL	0.04	< 0.001	< 0.001	✓
SMART_BO	PLACKETT_BURMAN	0.04	< 0.001	< 0.001	✓
FULL_FACTORIAL	FRACTIONAL_FACTORIAL	0.02	< 0.001	< 0.001	✓

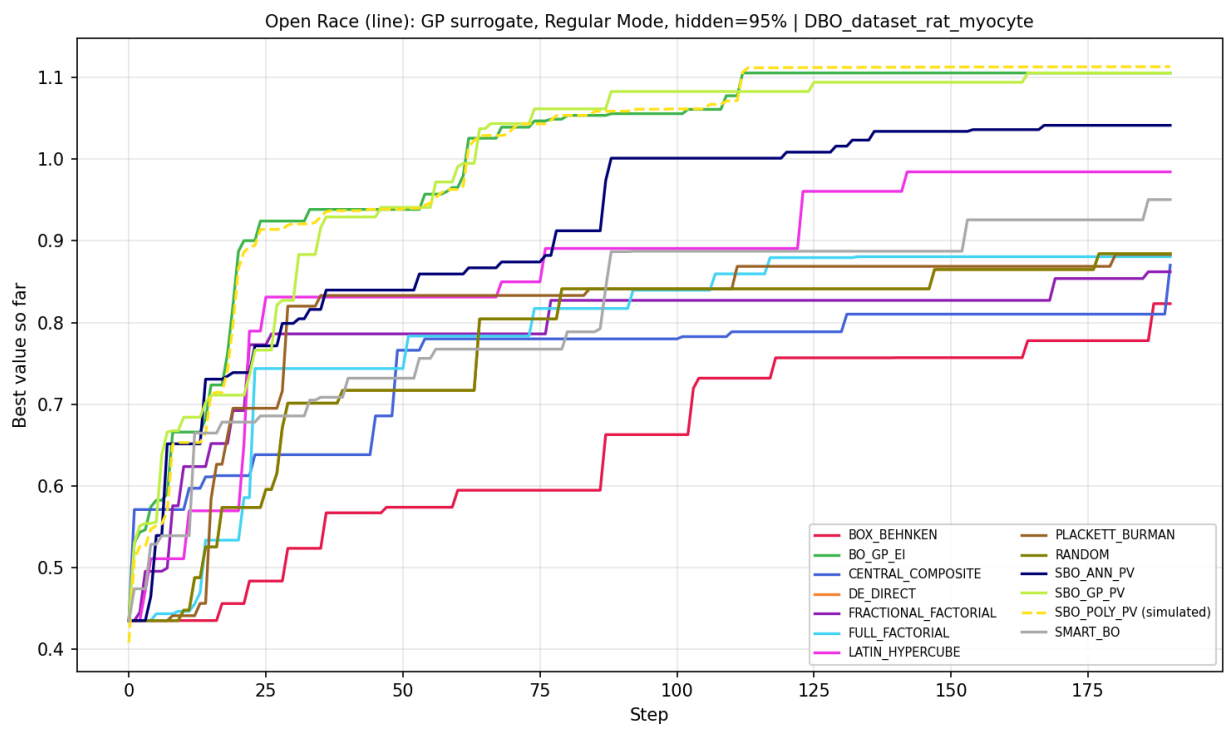

(a) DBO rat myocyte.

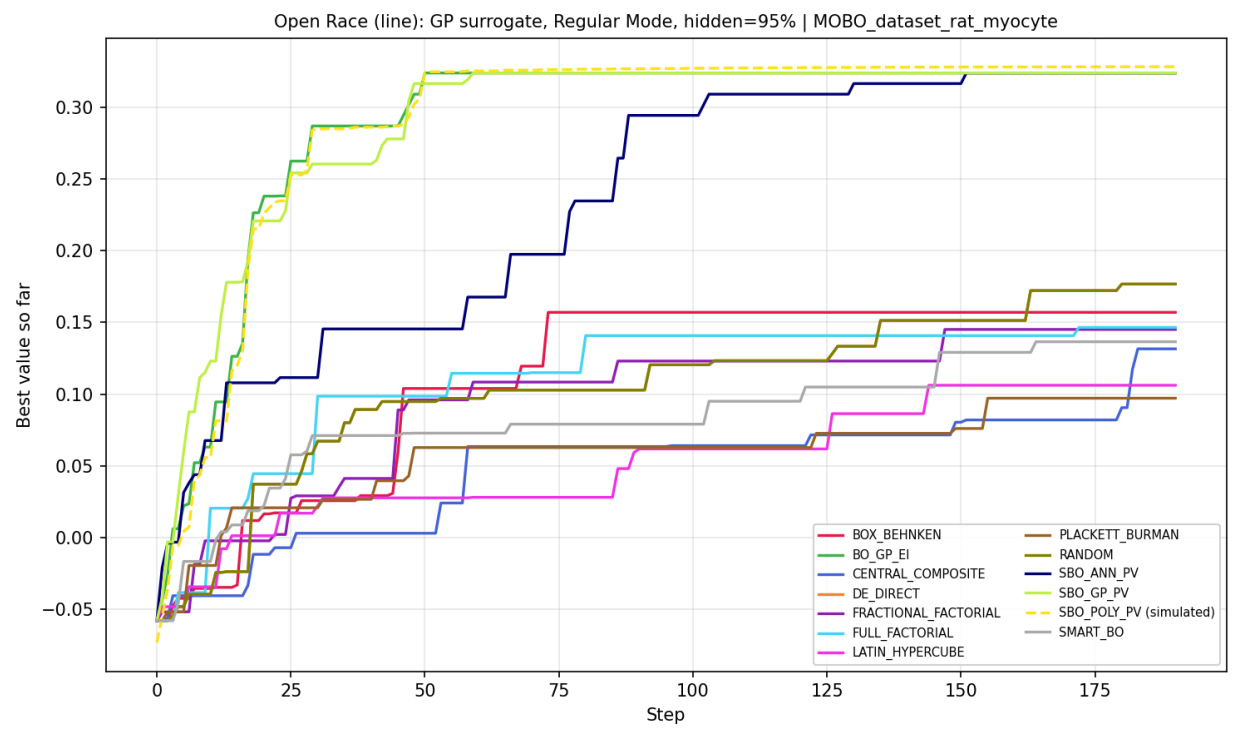

(b) MOBO rat myocyte.

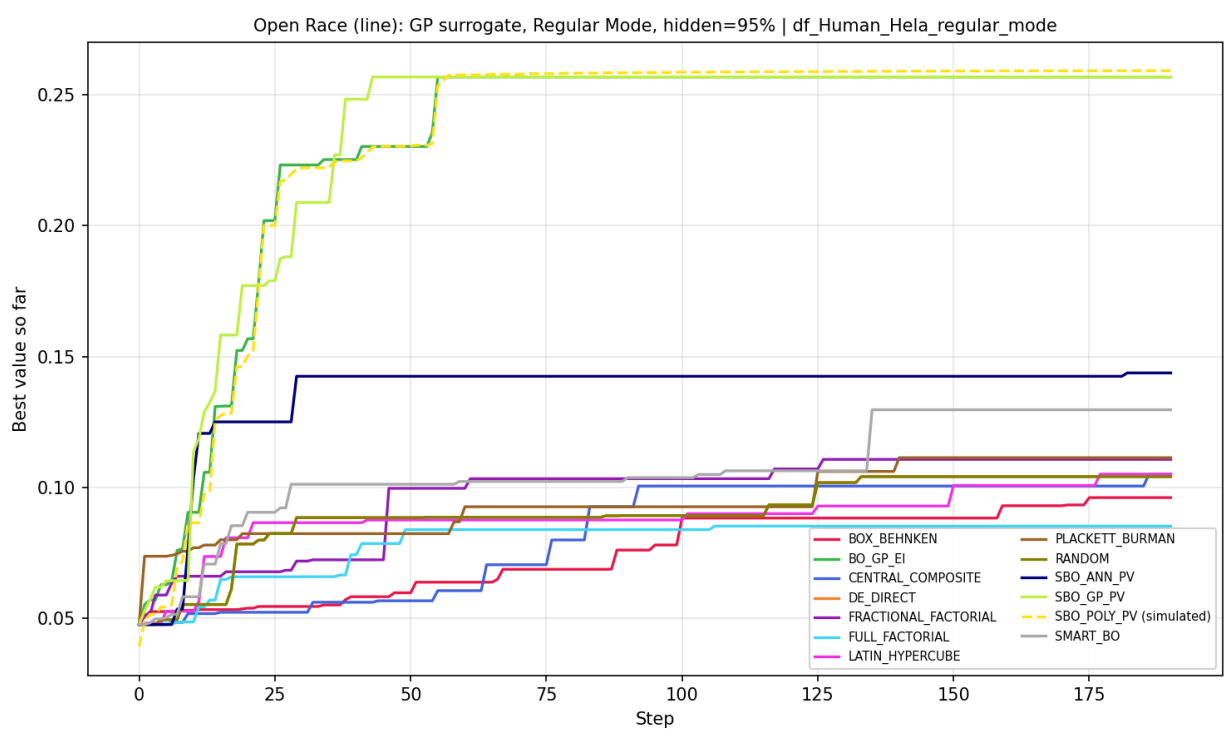

(c) HeLa (regular mode).

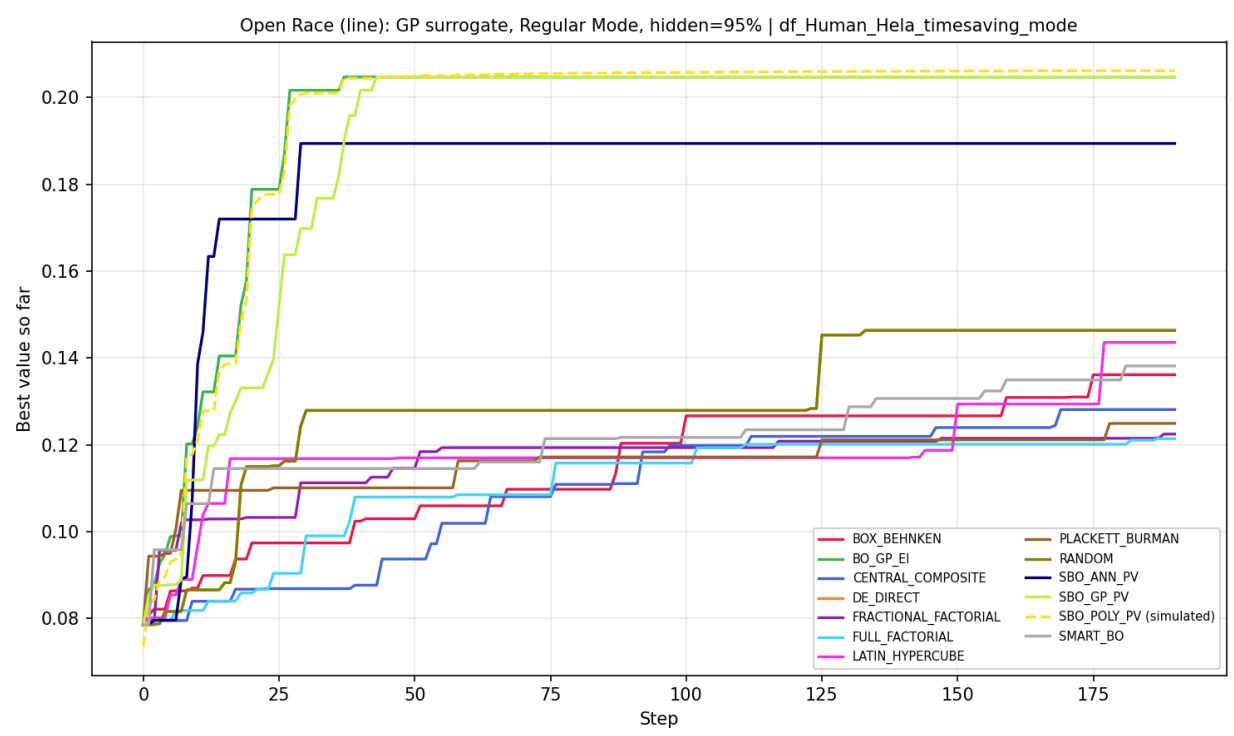

(d) HeLa (time-saving mode).

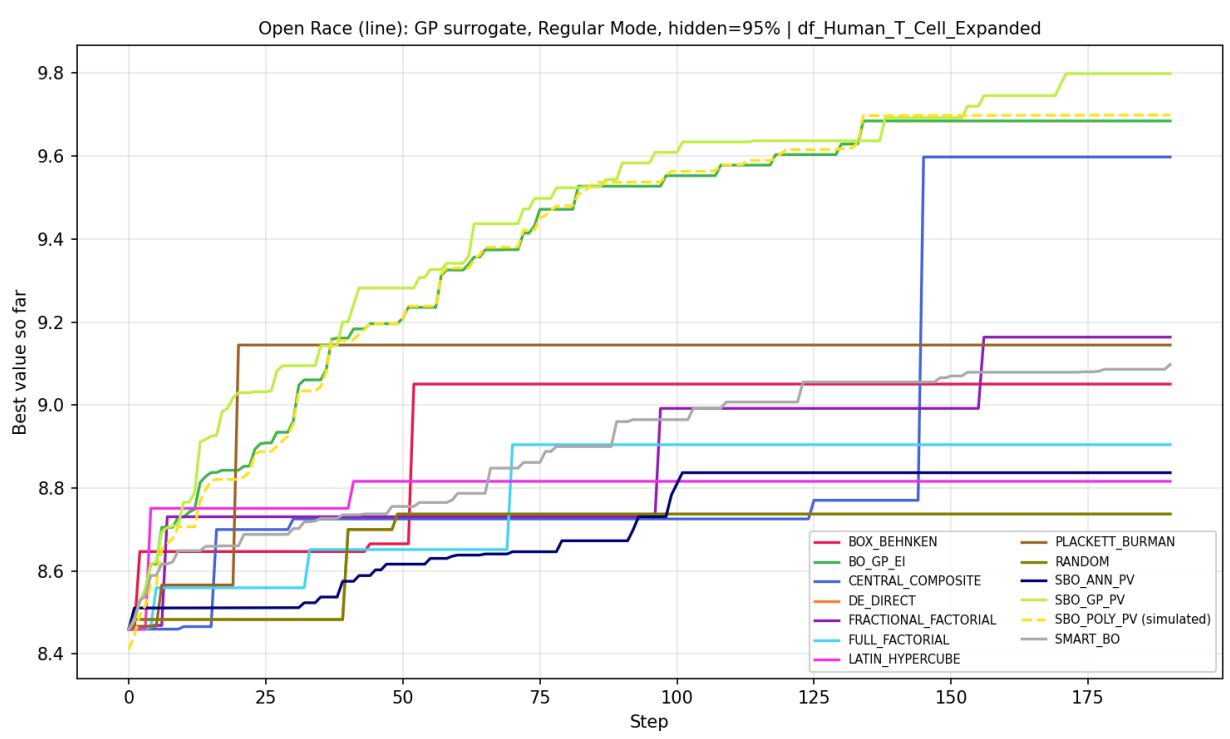

(e) T-Cell.

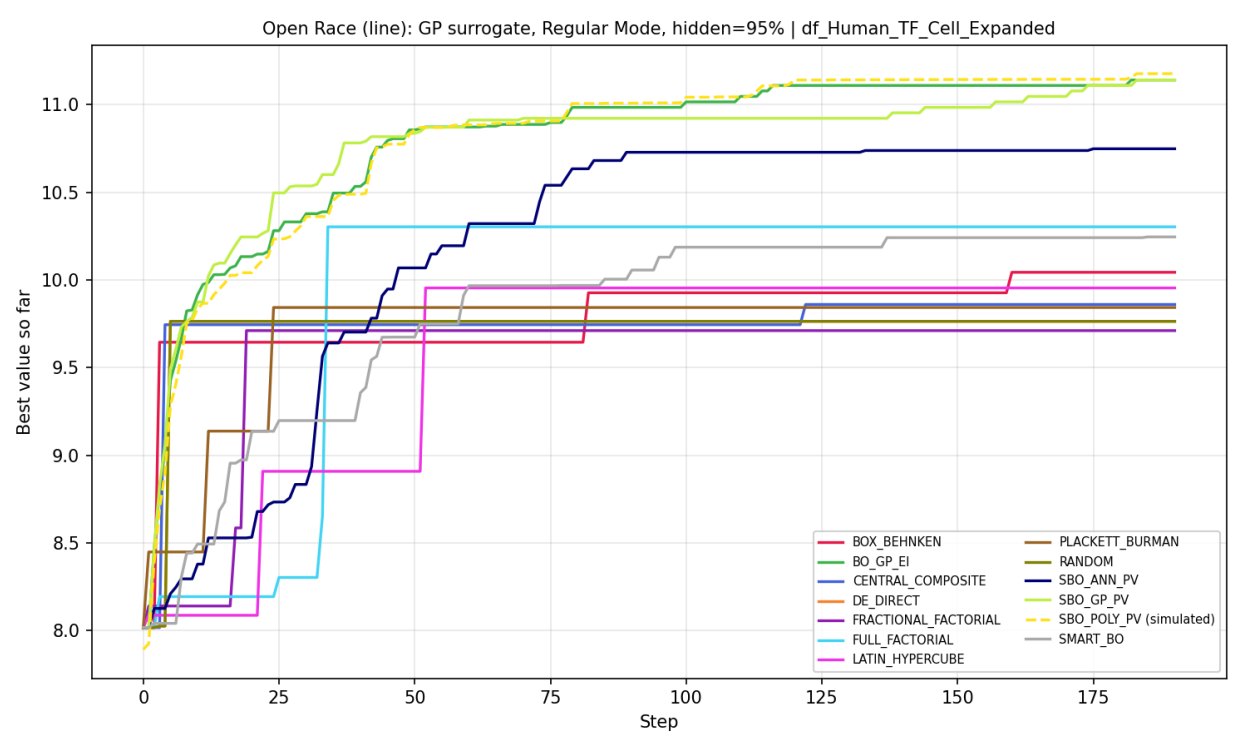

(f) TF-Cell.

Fig. 3. Open Race (OR) competition results. **Configuration:** Regular Mode (GP surrogate, low noise), hidden fraction = 95% (5% initially revealed). **Datasets:** DBO rat myocyte (a), MOBO rat myocyte (b), HeLa regular mode (c), HeLa time-saving mode (d), T-Cell (e), TF-Cell (f). **Metric:** best value found so far over cumulative evaluation steps (higher is better).

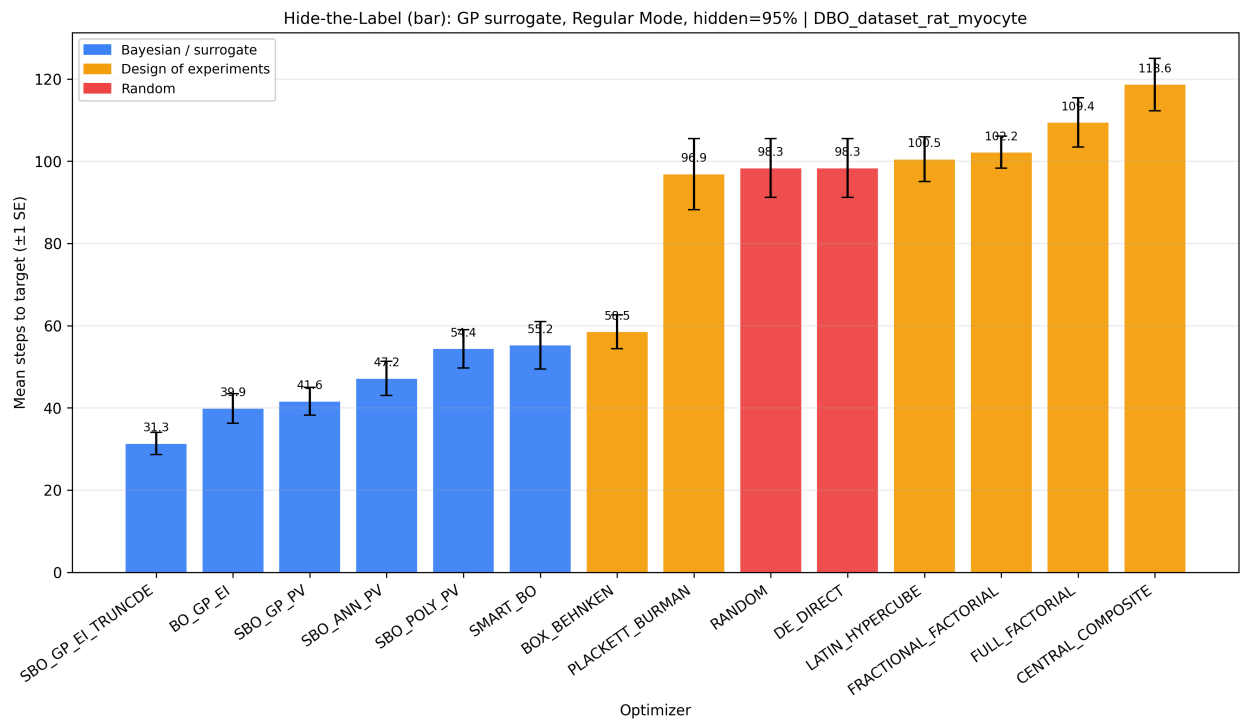

(a) DBO rat myocyte.

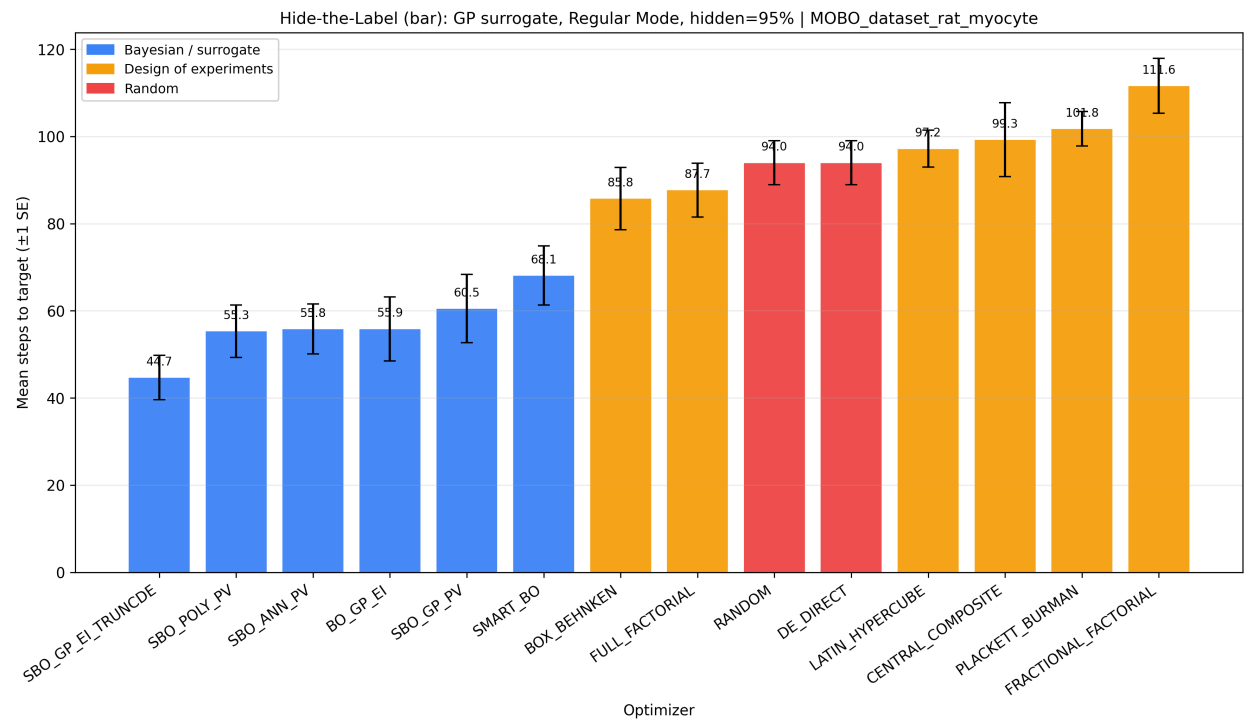

(b) MOBO rat myocyte.

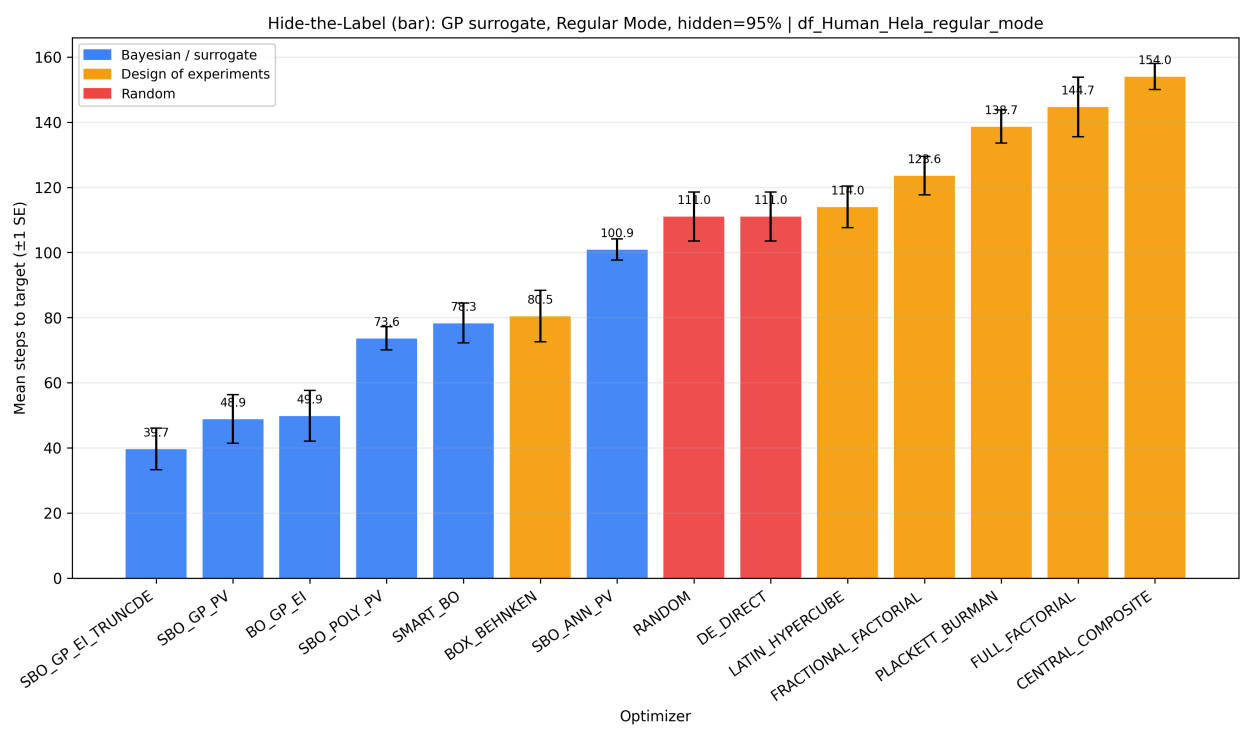

(c) HeLa (regular mode).

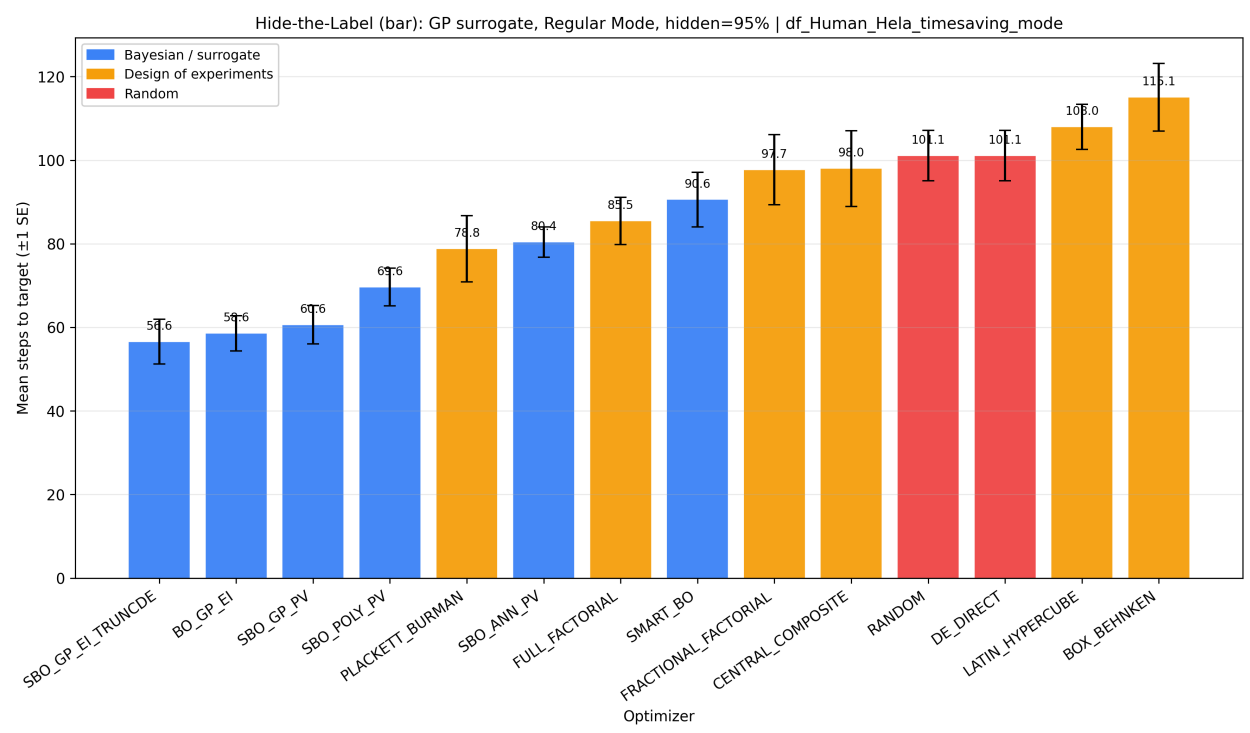

(d) HeLa (time-saving mode).

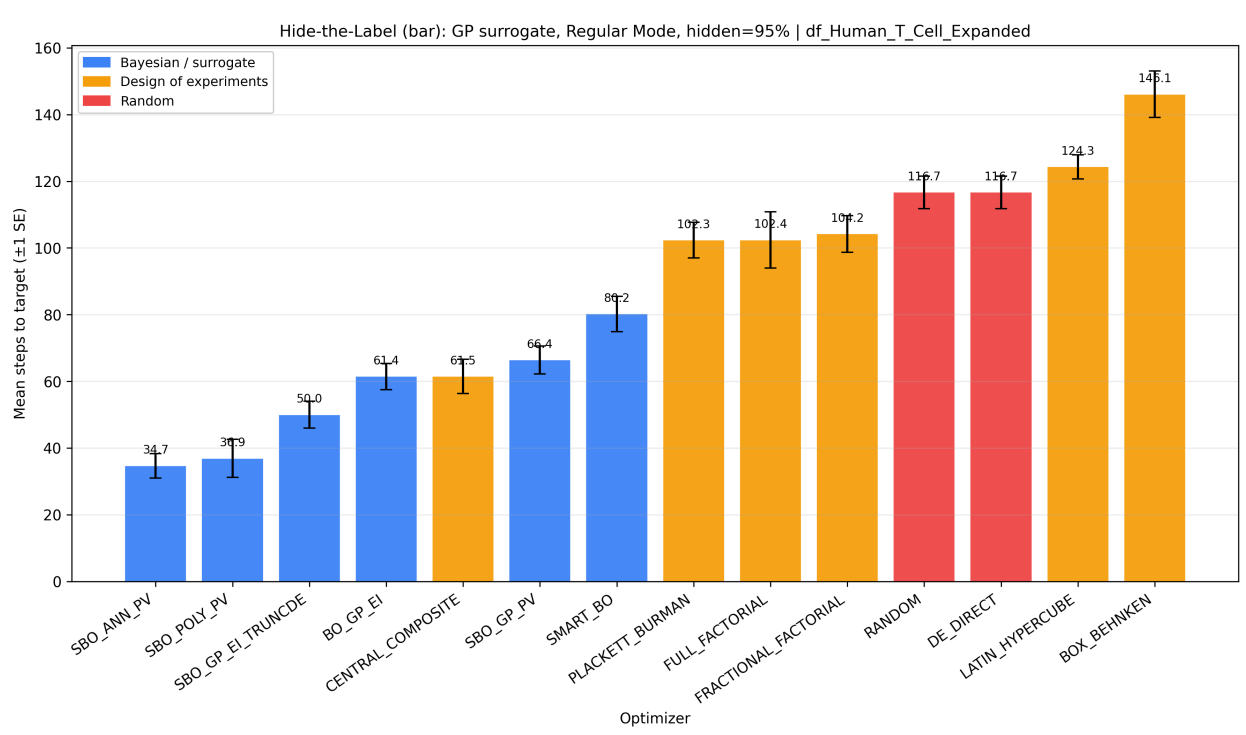

(e) T-Cell.

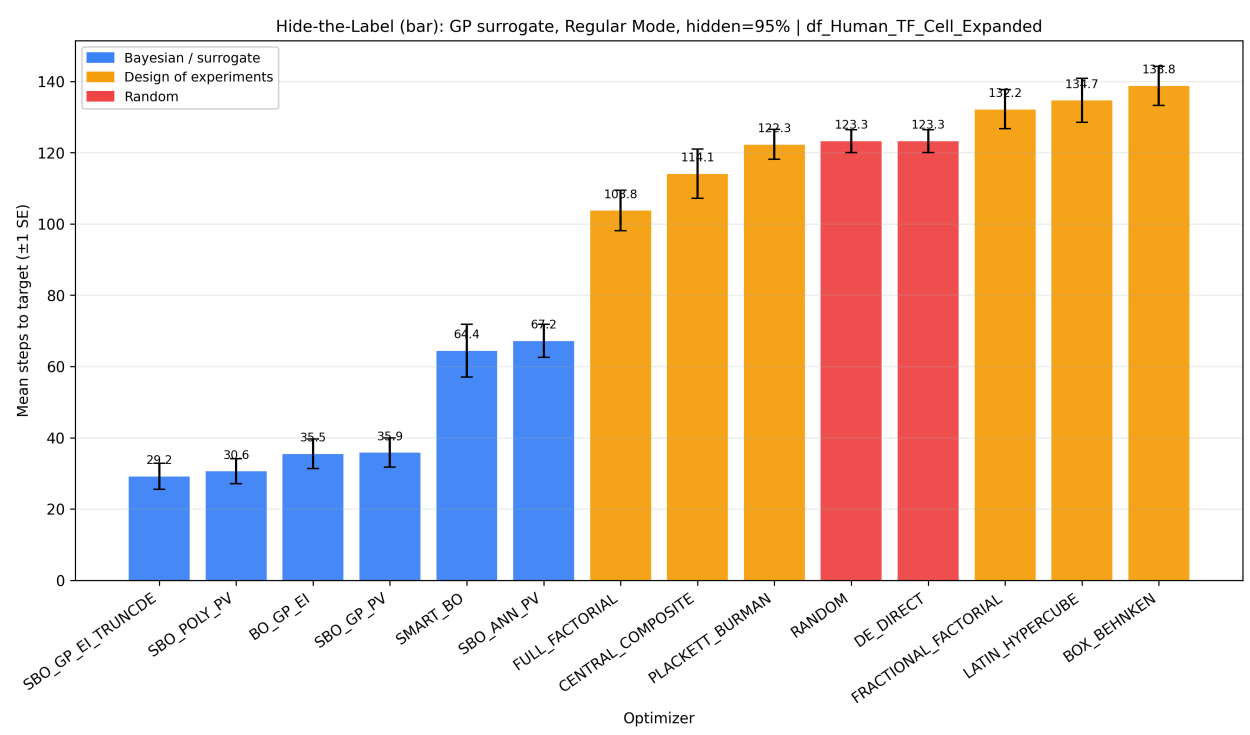

(f) TF-Cell.

Fig. 4. Hide-the-Label (HTL) competition results. **Configuration:** Regular Mode (GP surrogate, low noise), hidden fraction = 95% (5% initially revealed), Batch size = 1. **Datasets:** DBO rat myocyte (a), MOBO rat myocyte (b), HeLa regular mode (c), HeLa time-saving mode (d), T-Cell (e), TF-Cell (f). **Metric:** steps to find target label (lower is better).

the "Design of Experiment methods" section above and in (Montgomery, 2017). Because DOE methods generate their full set of candidate points upfront (they do not adapt based on observations), "multiple steps" in the benchmark are implemented by splitting the pre-planned design into batches of size B: the first B points are revealed at step 1, the next B at step 2, and so on. No response surface model is fitted between steps—the entire design is fixed before any observations are collected. If the pre-planned design is exhausted before the target is found, further batches are drawn uniformly at random from the remaining unqueried candidates. This fixed-design behaviour is precisely what distinguishes DOE from adaptive methods and is the source of their lower efficiency in sequential benchmarks. In the Hide-the-Label protocol, DOE methods may propose points outside the existing dataset; in such cases the nearest available point by Euclidean (L2) distance is selected instead.

Baseline

`RANDOM`: Uniform random sampling from the candidate pool; provides the floor of expected performance.

References

Z. Cosenza et al. Multi-information source bayesian optimization of culture media for cellular agriculture. *Biotechnology and Bioengineering*, 119(9):2447–2458, 2022. doi: 10.1002/bit.28

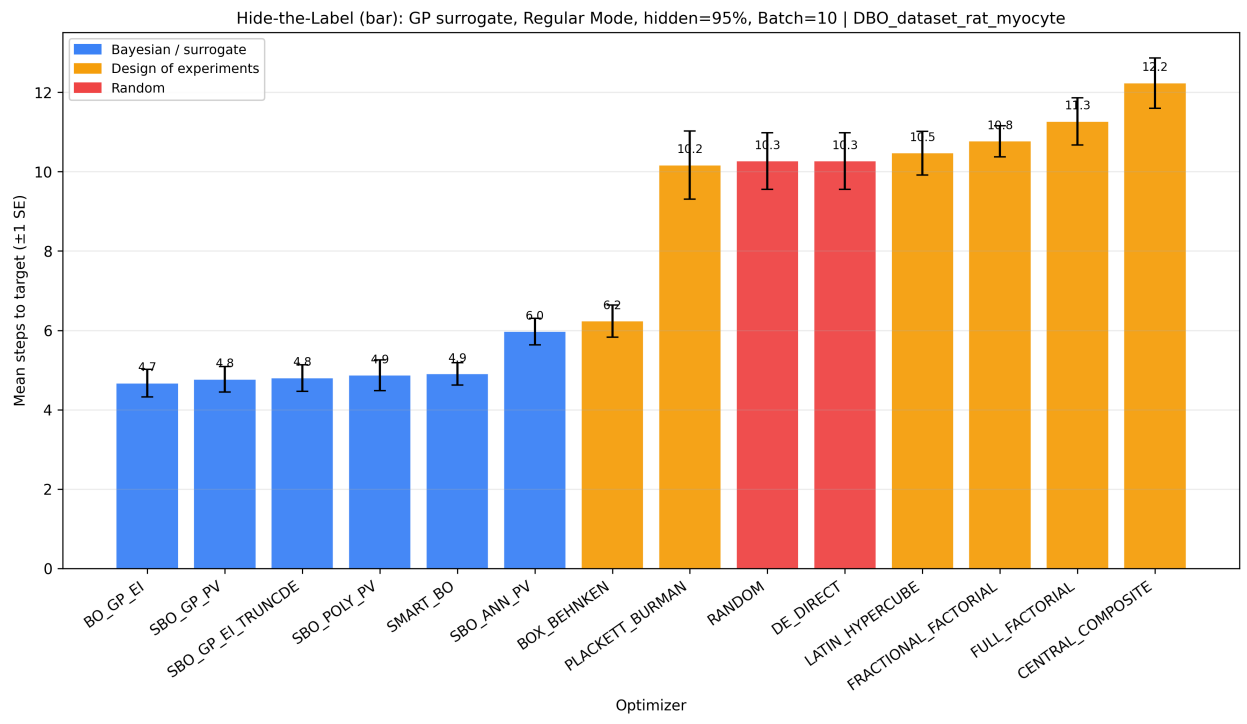

(a) DBO rat myocyte.

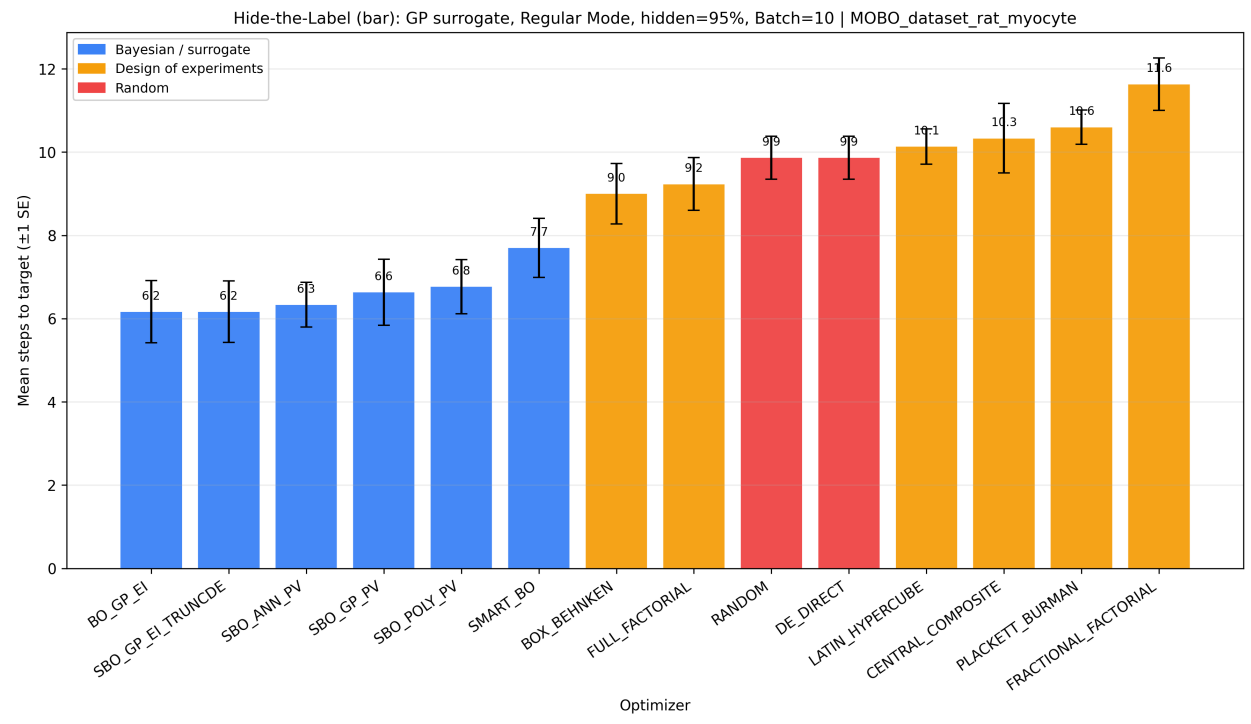

(b) MOBO rat myocyte.

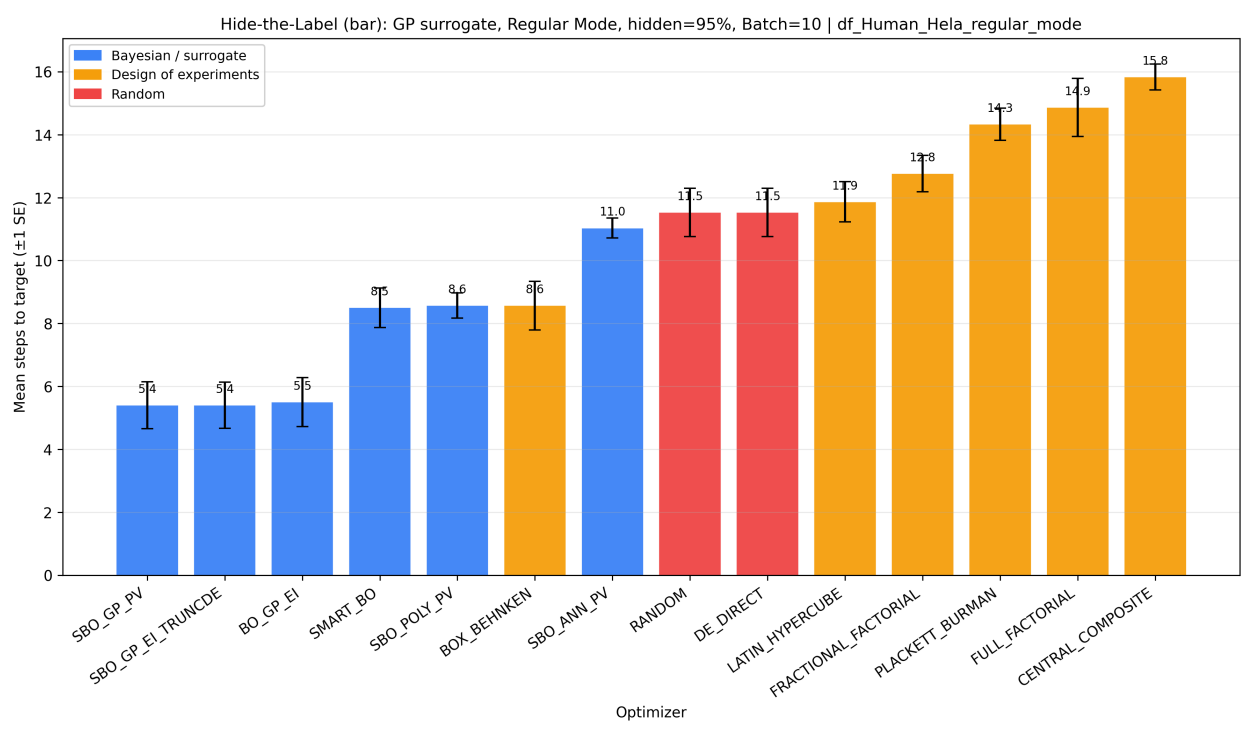

(c) HeLa (regular mode).

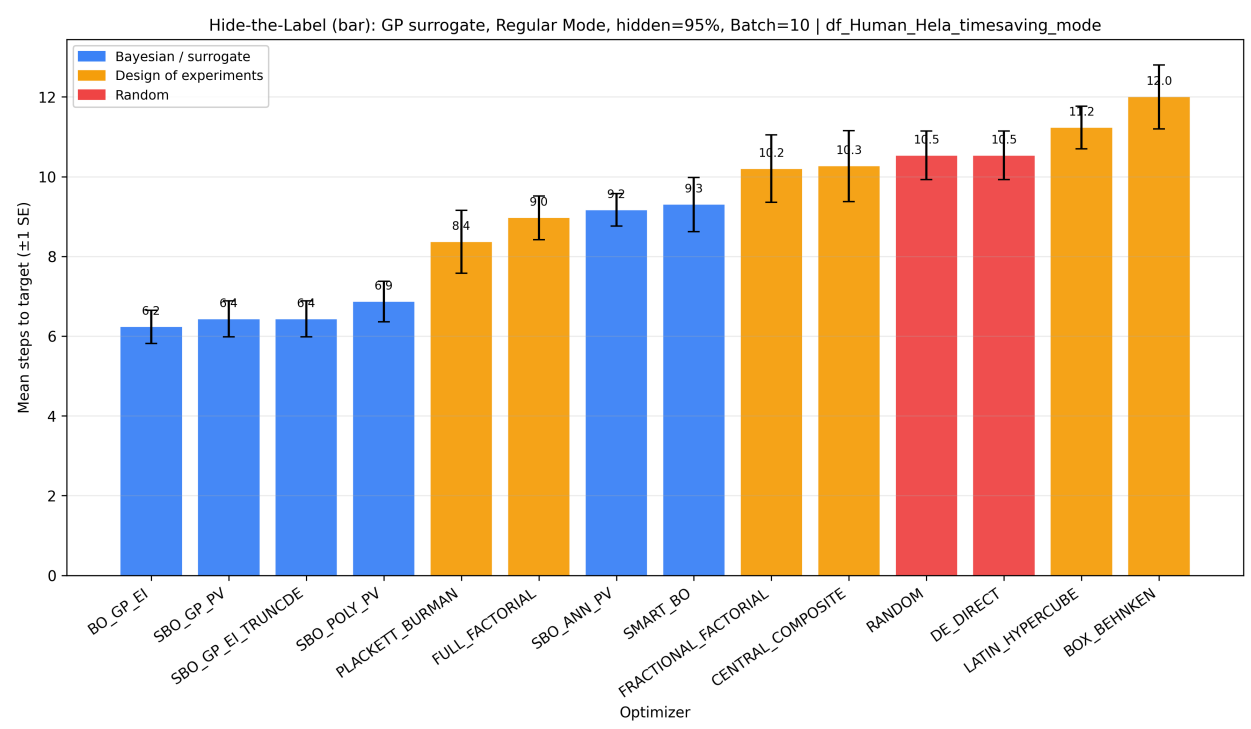

(d) HeLa (time-saving mode).

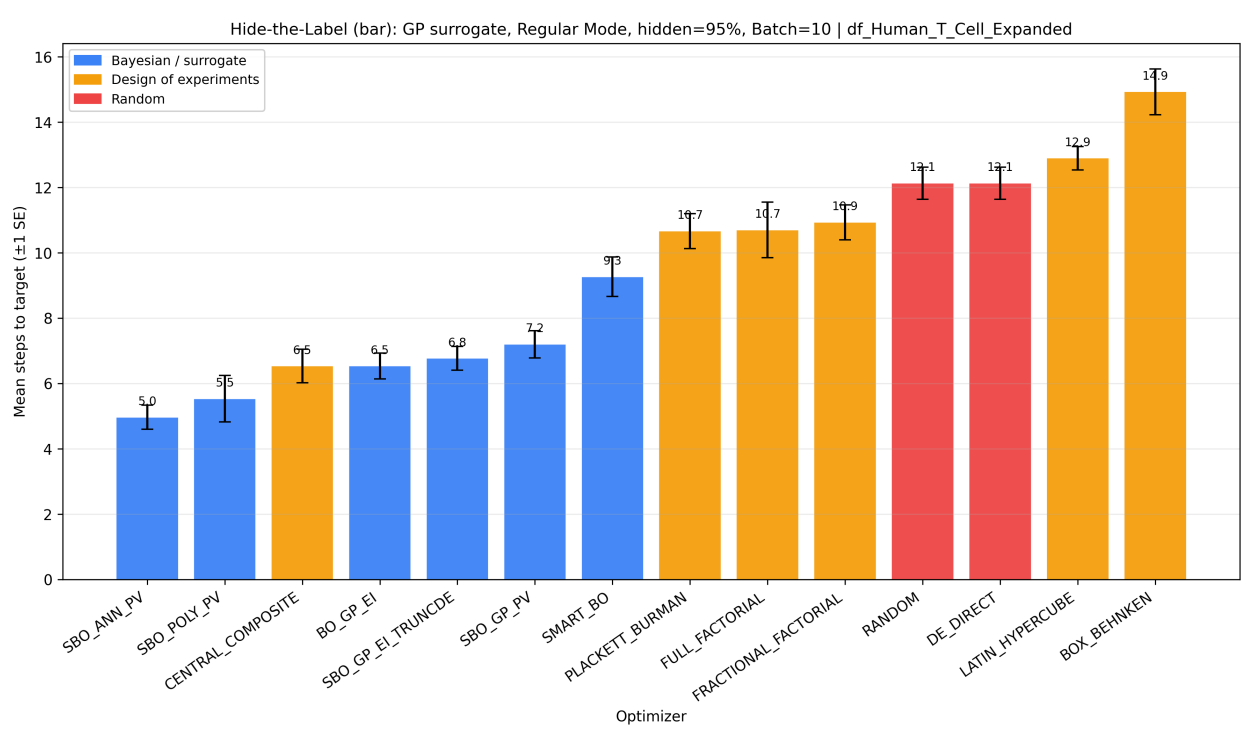

(e) T-Cell.

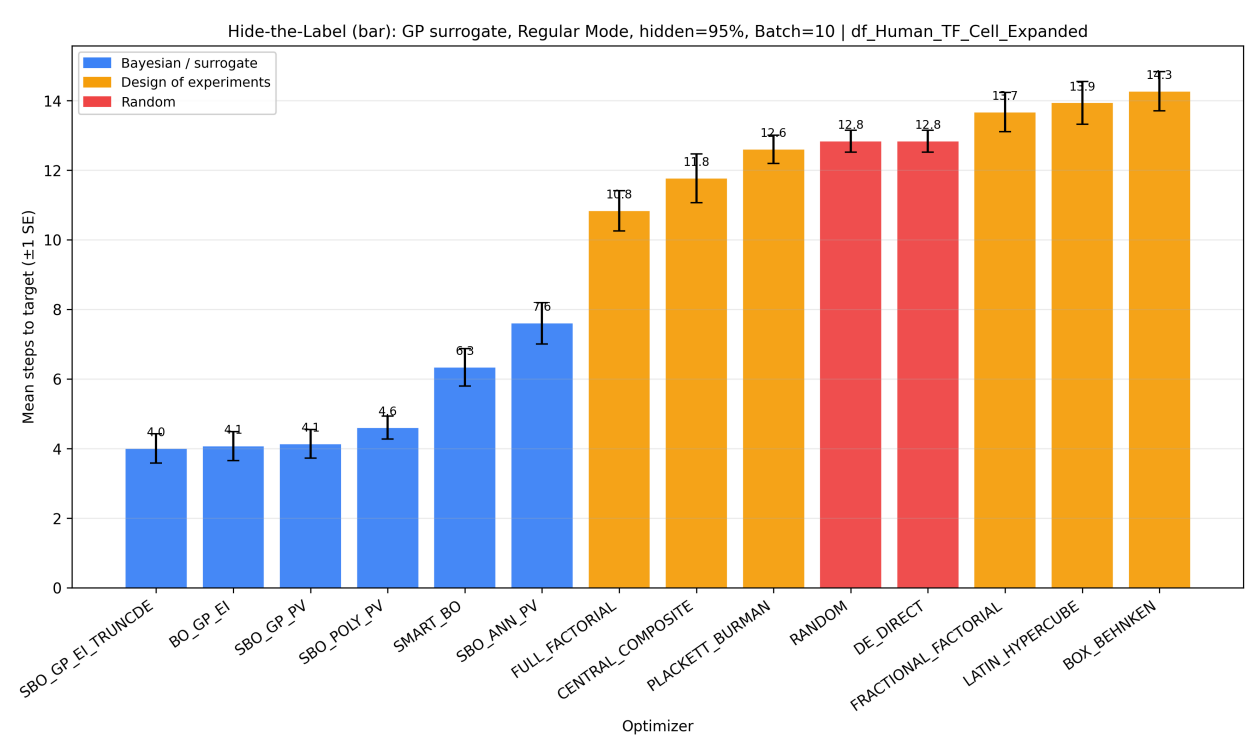

(f) TF-Cell.

Fig. 5. Hide-the-Label (HTL) competition results. **Configuration:** Regular Mode (GP surrogate, low noise), hidden fraction = 95% (5% initially revealed), Batch size = 10. **Datasets:** DBO rat myocyte (a), MOBO rat myocyte (b), HeLa regular mode (c), HeLa time-saving mode (d), T-Cell (e), TF-Cell (f). **Metric:** steps to find target label (lower is better).

132.

D. C. Montgomery. *Design and analysis of experiments*. John wiley & sons, 2017.

C. E. Rasmussen and C. K. I. Williams. *Gaussian Processes for Machine Learning*. MIT Press, Cambridge, MA, 2006.

M. L. Stein. *Interpolation of Spatial Data: Some Theory for Kriging*. Springer, New York, 1999. doi: 10.1007/978-1-461 2-1494-6.

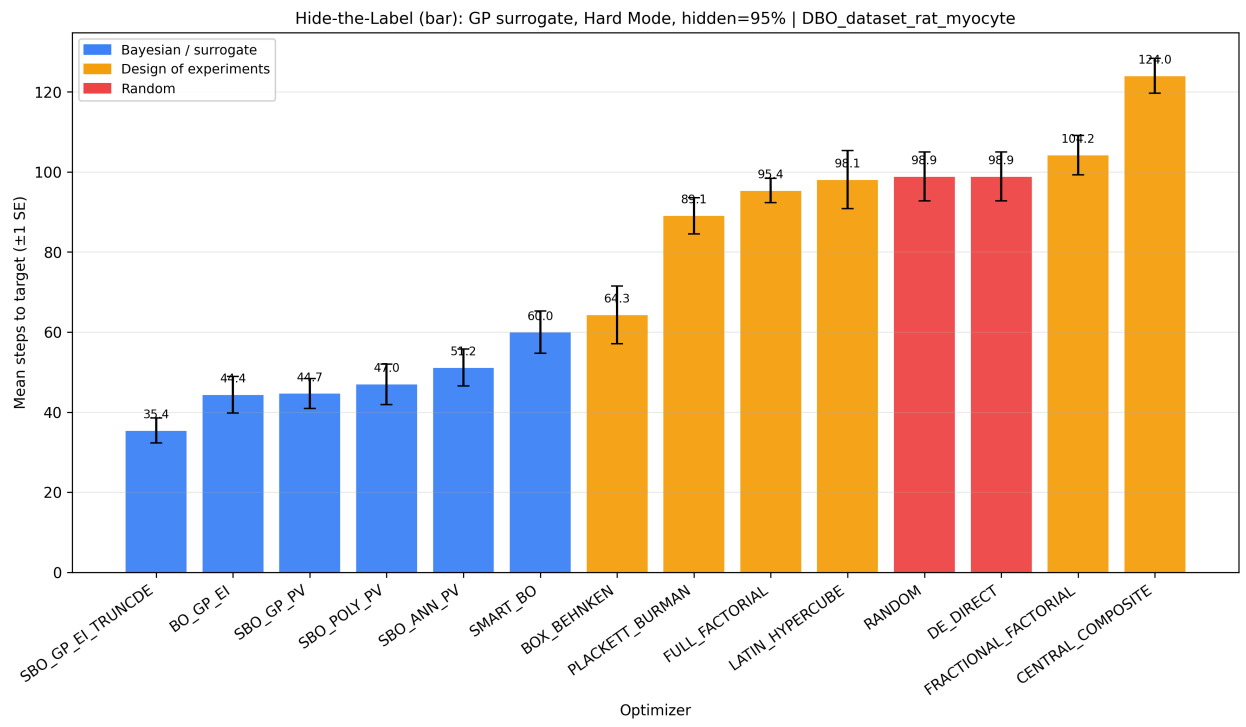

(a) DBO rat myocyte.

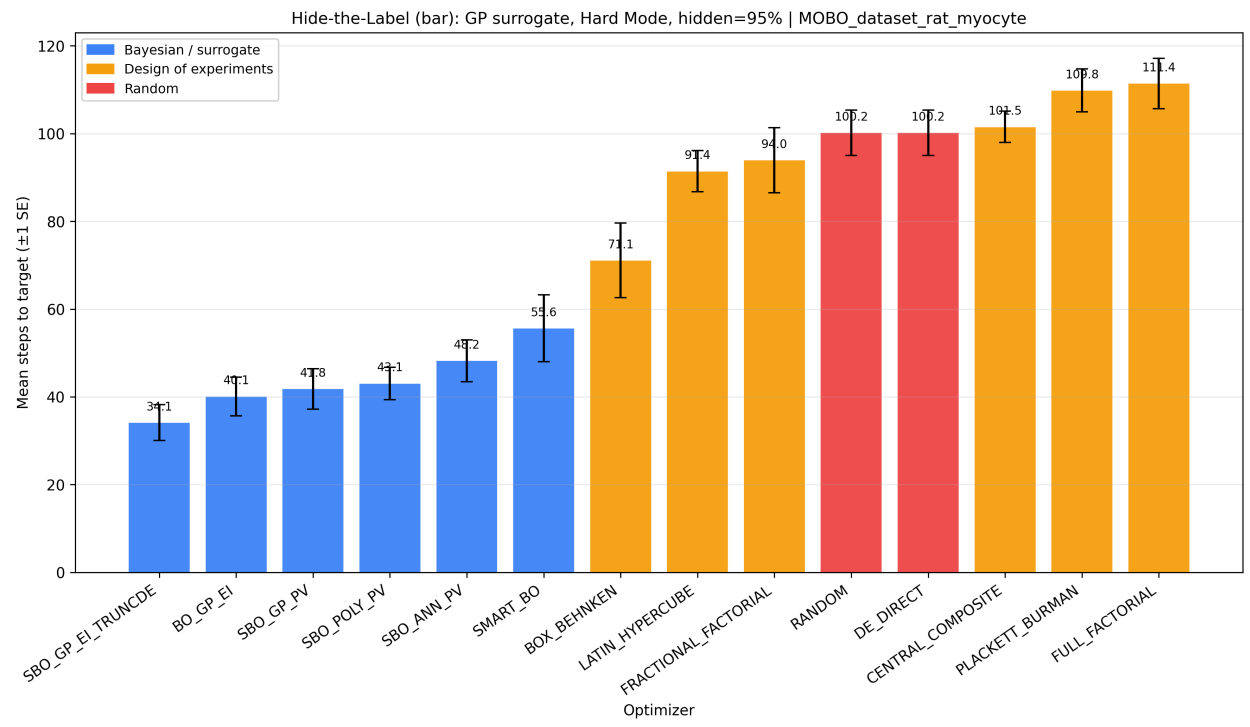

(b) MOBO rat myocyte.

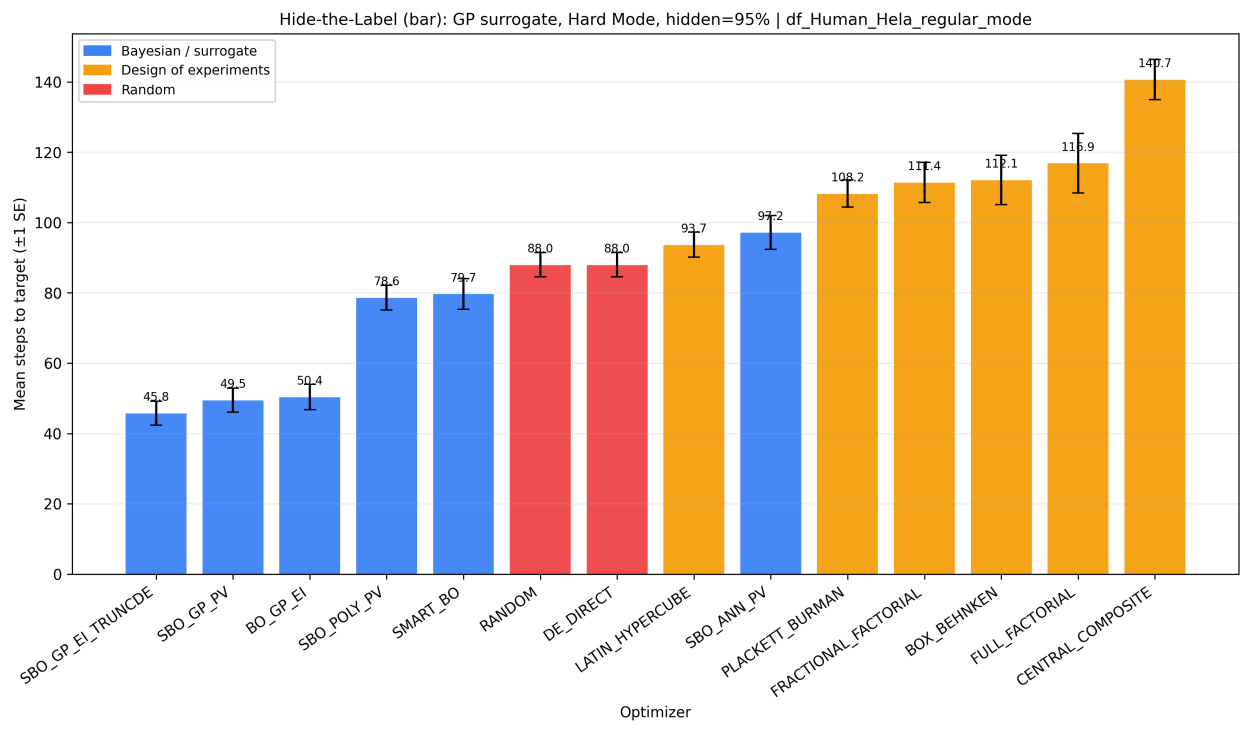

(c) HeLa (regular mode).

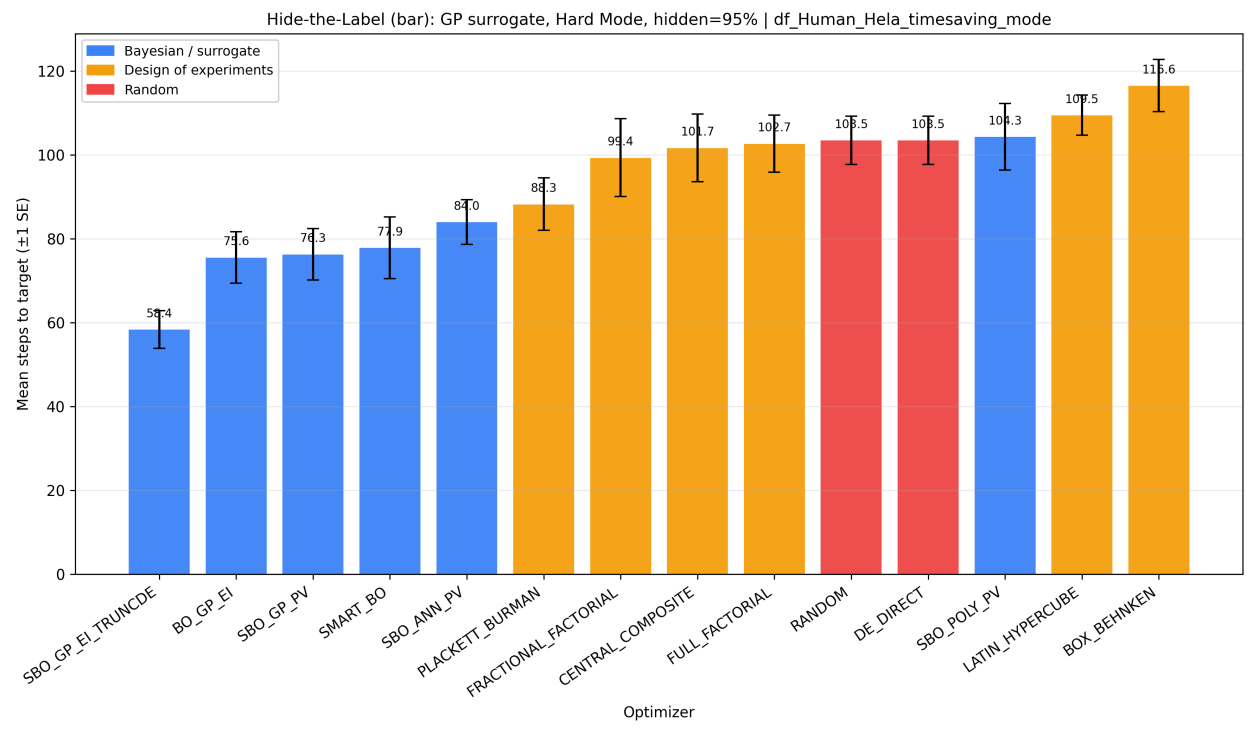

(d) HeLa (time-saving mode).

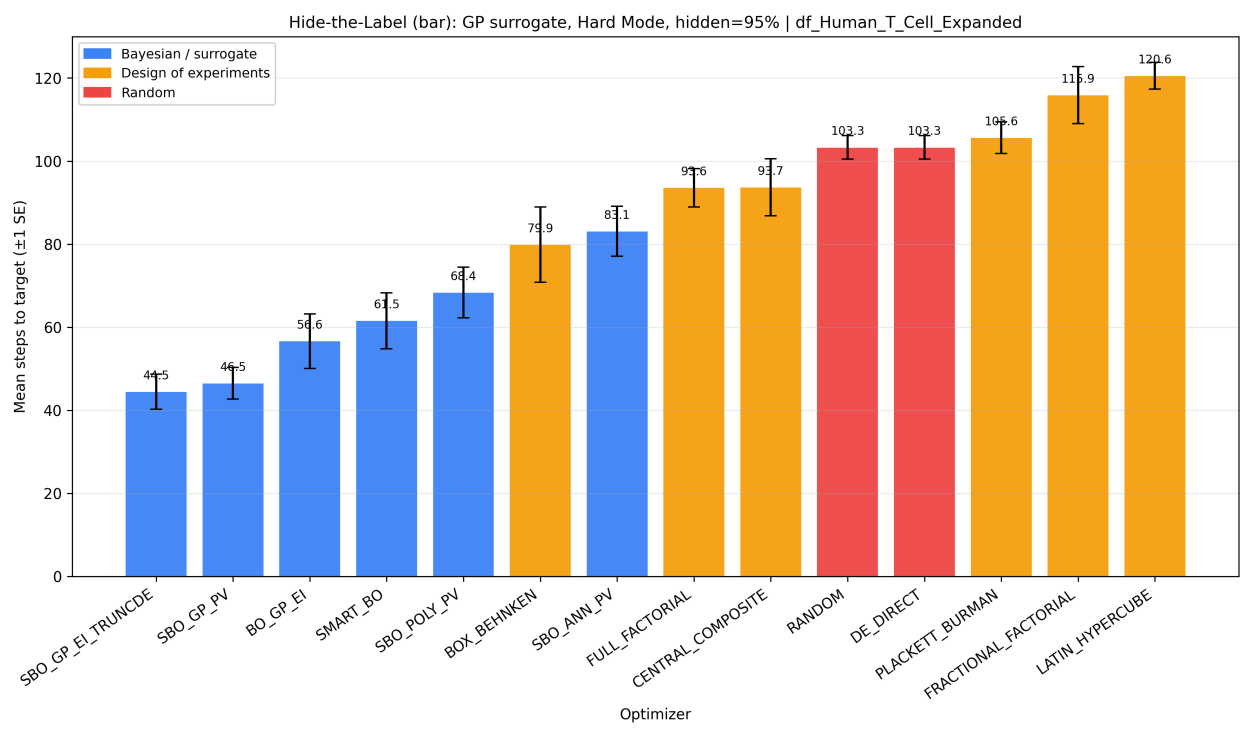

(e) T-Cell.

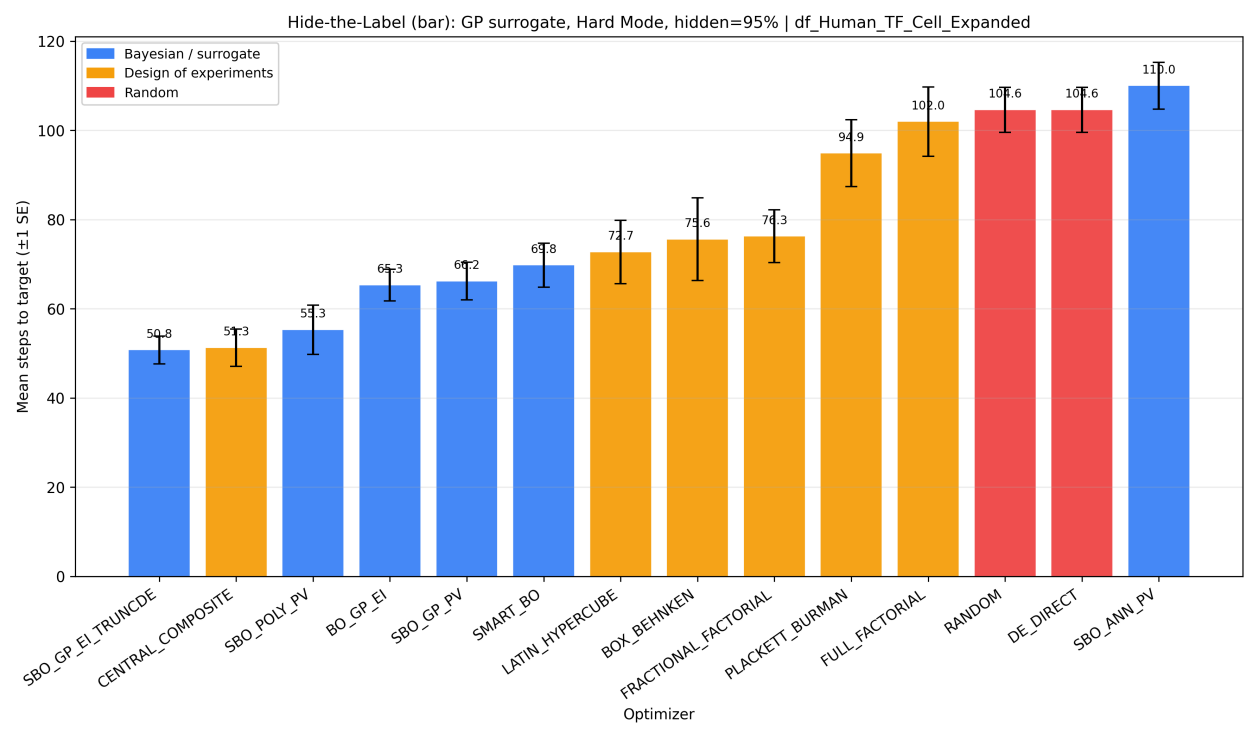

(f) TF-Cell.

Fig. 6. Hide-the-Label (HTL) competition results. **Configuration:** Hard Mode (GP surrogate, heteroskedastic noise & bimodal perturbations), hidden fraction = 95% (5% initially revealed), Batch size = 1. **Datasets:** DBO rat myocyte (a), MOBO rat myocyte (b), HeLa regular mode (c), HeLa time-saving mode (d), T-Cell (e), TF-Cell (f). **Metric:** steps to find target label (lower is better).

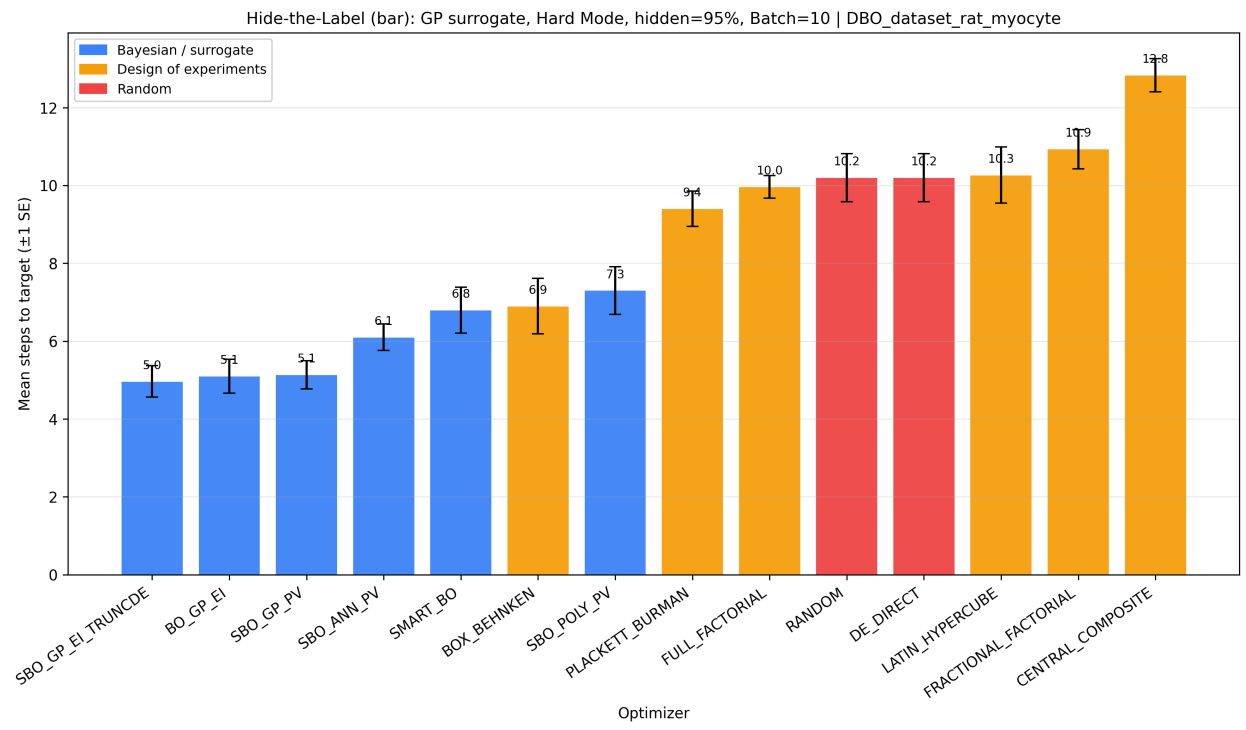

(a) DBO rat myocyte.

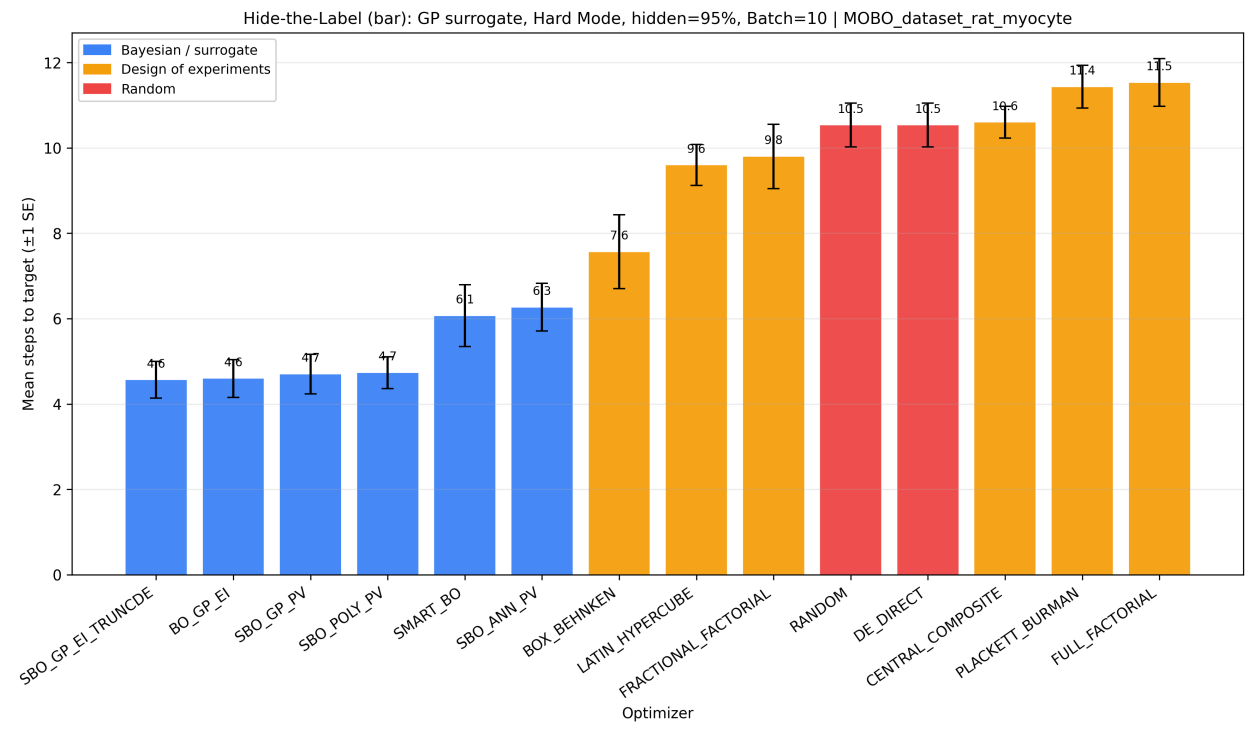

(b) MOBO rat myocyte.

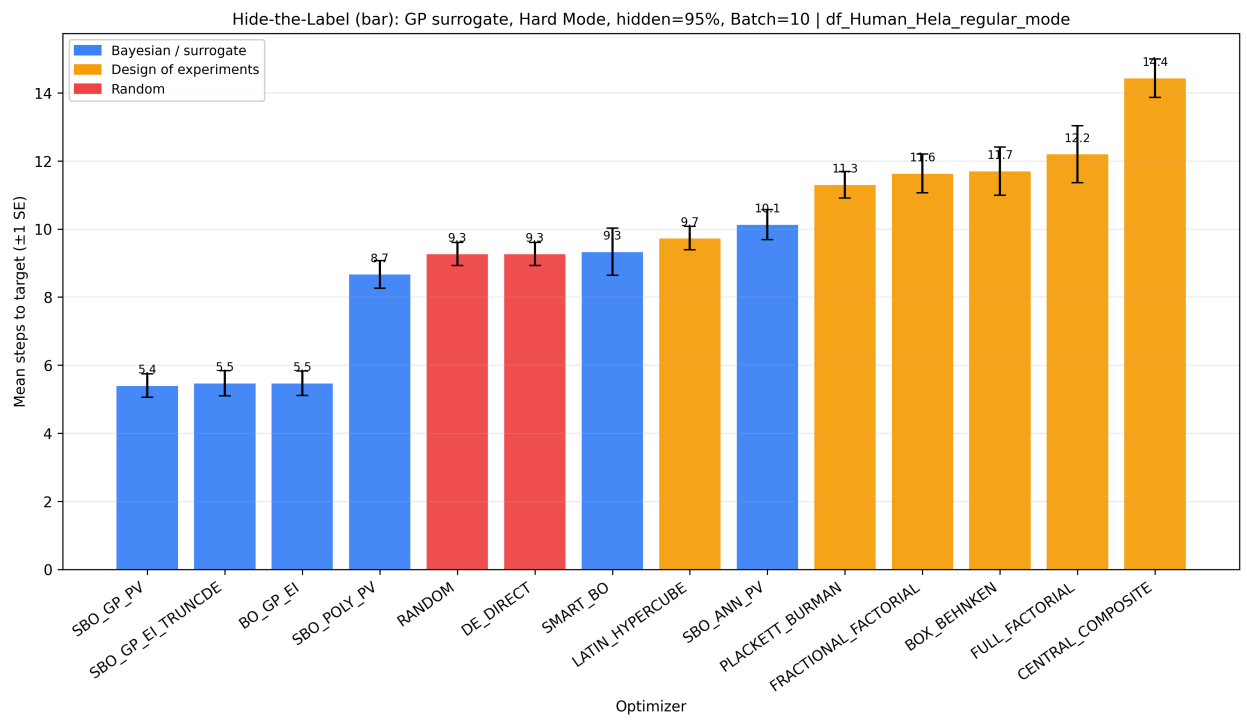

(c) HeLa (regular mode).

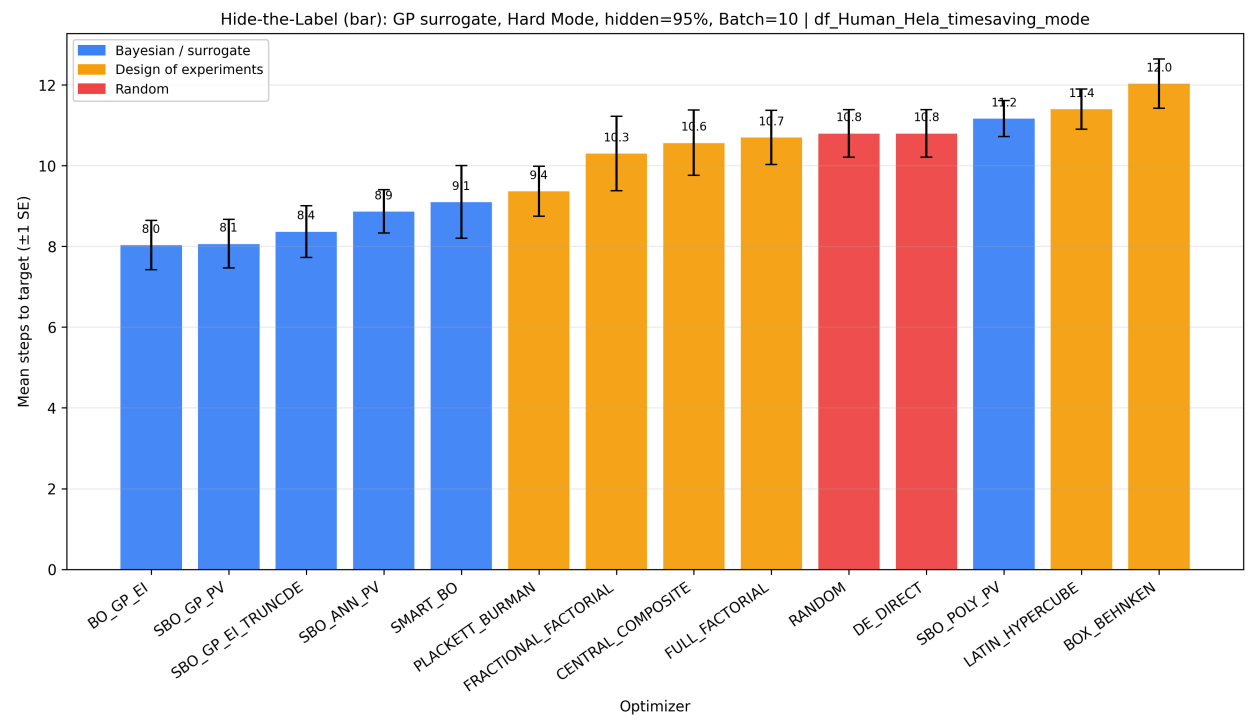

(d) HeLa (time-saving mode).

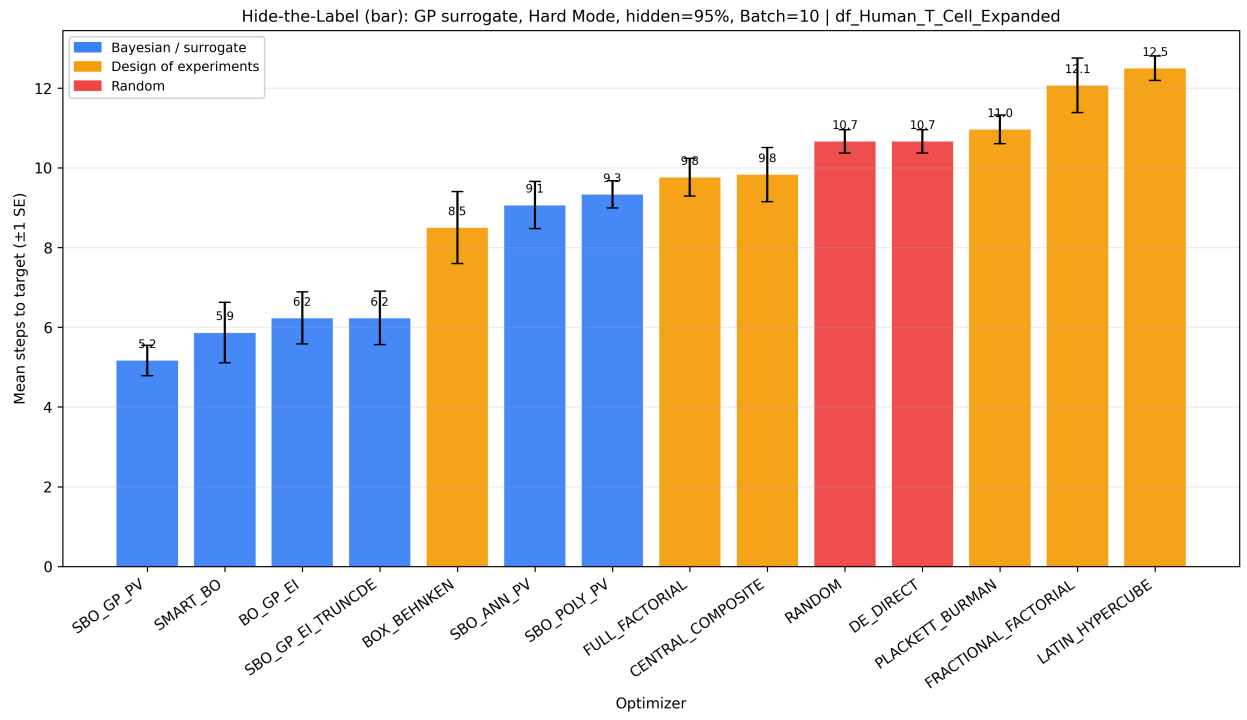

(e) T-Cell.

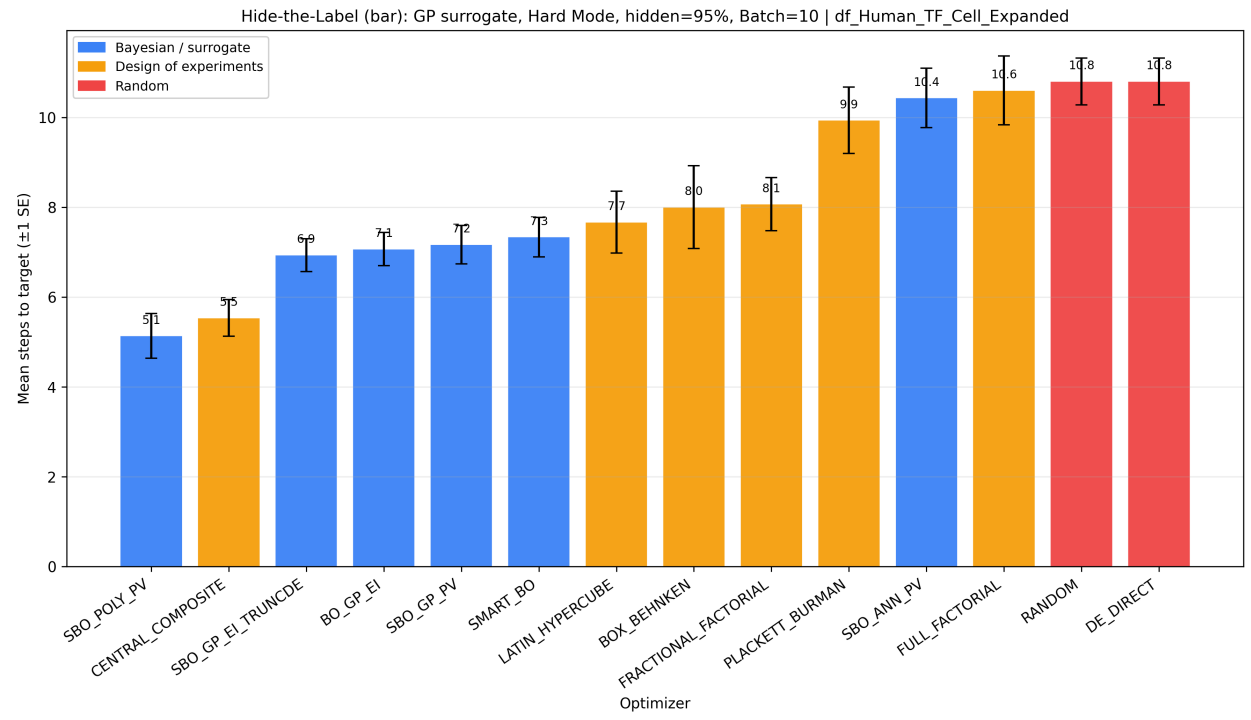

(f) TF-Cell.

Fig. 7. Hide-the-Label (HTL) competition results. **Configuration:** Hard Mode (GP surrogate, heteroskedastic noise & bimodal perturbations), hidden fraction = 95% (5% initially revealed), Batch size = 10. **Datasets:** DBO rat myocyte (a), MOBO rat myocyte (b), HeLa regular mode (c), HeLa time-saving mode (d), T-Cell (e), TF-Cell (f). **Metric:** steps to find target label (lower is better).

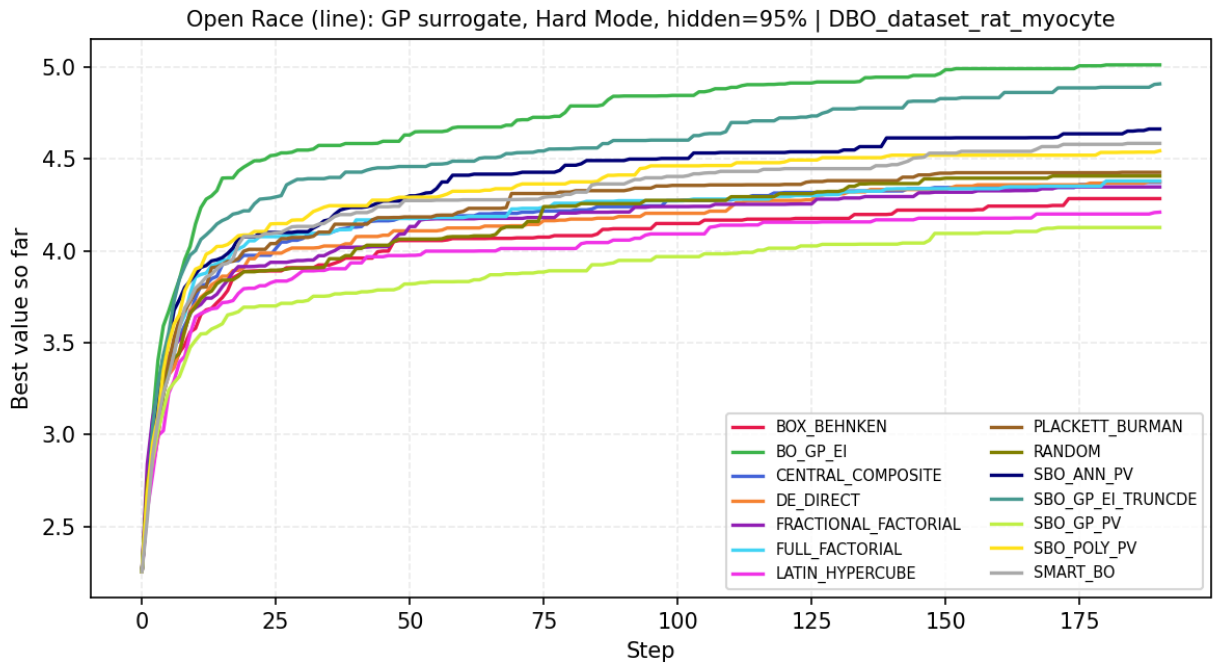

(a) DBO rat myocyte.

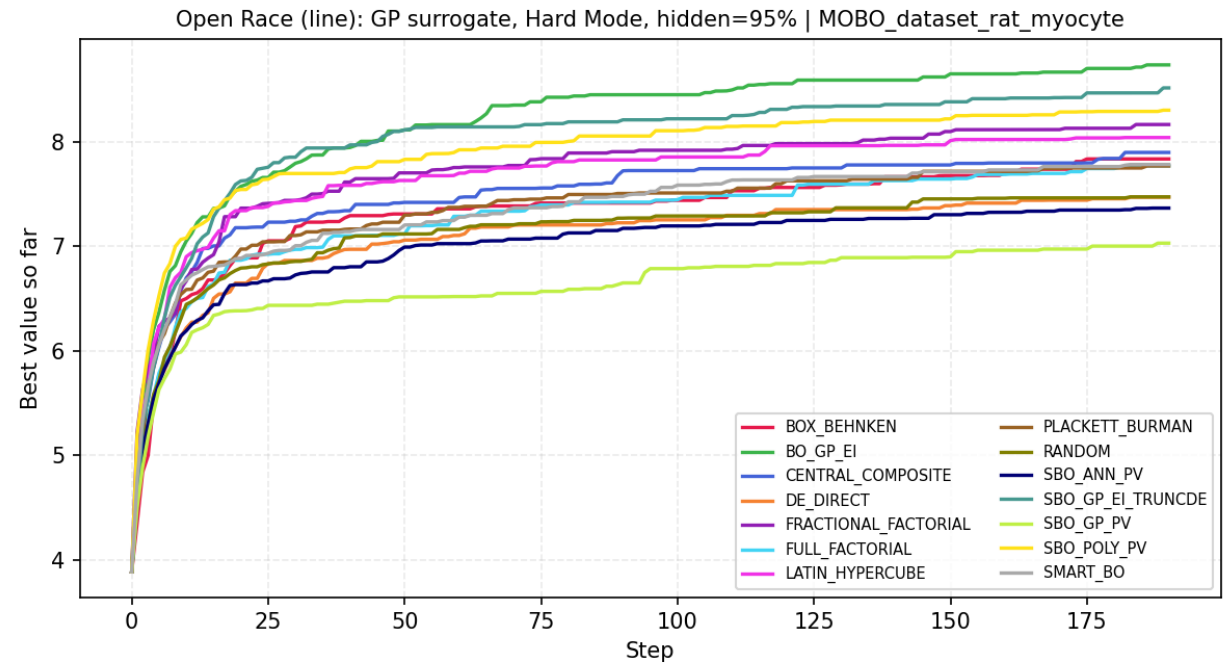

(b) MOBO rat myocyte.

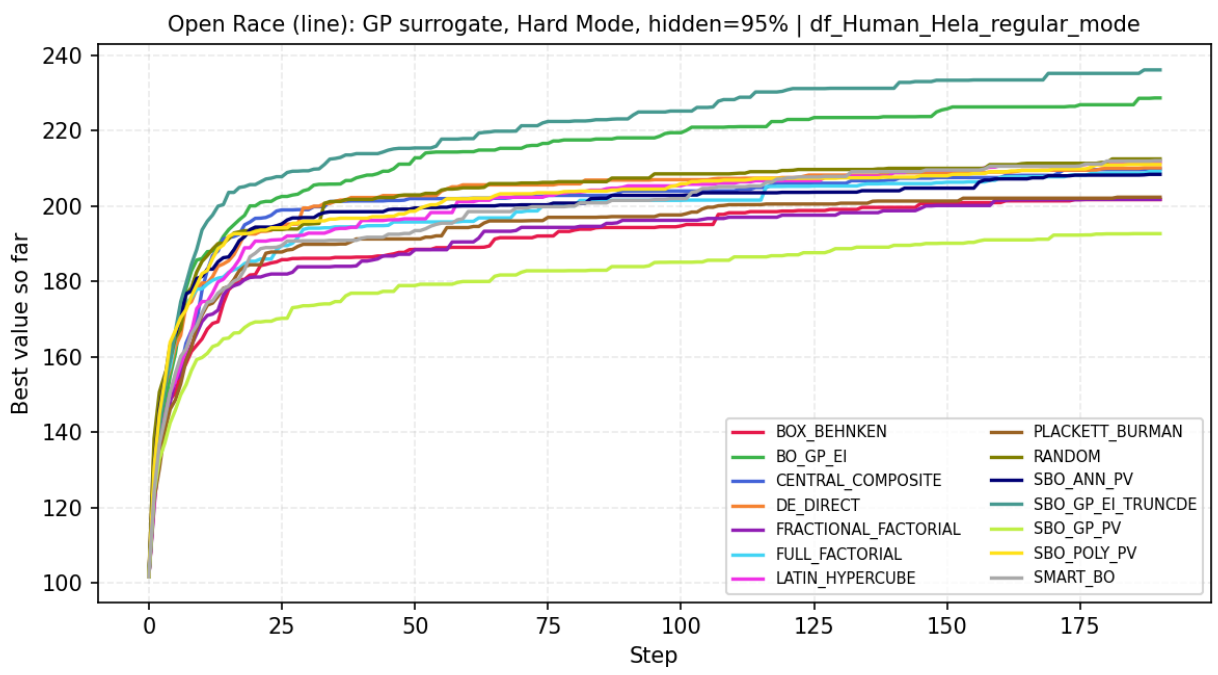

(c) HeLa (regular mode).

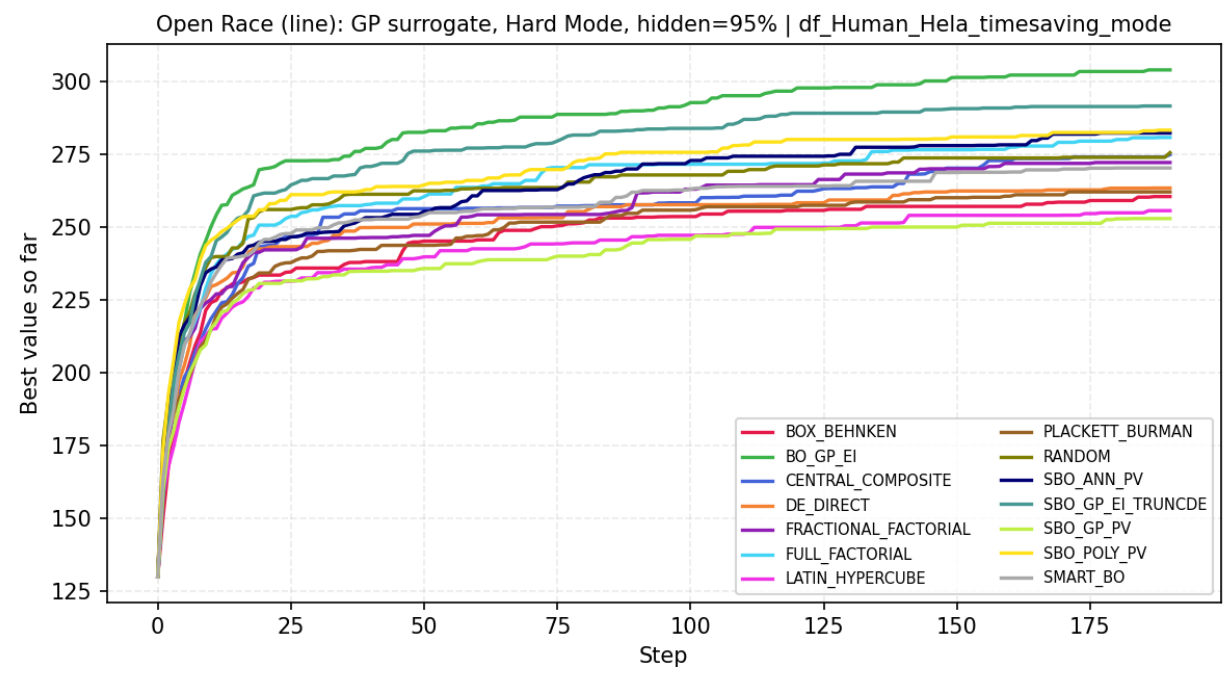

(d) HeLa (time-saving mode).

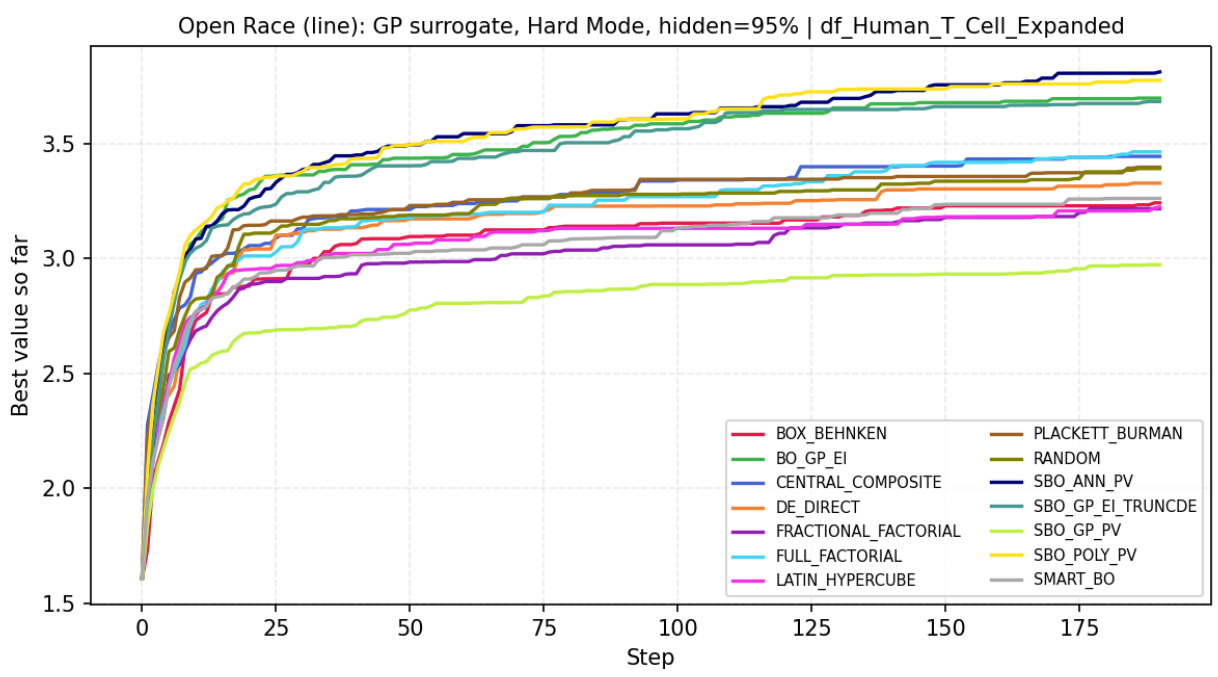

(e) T-Cell.

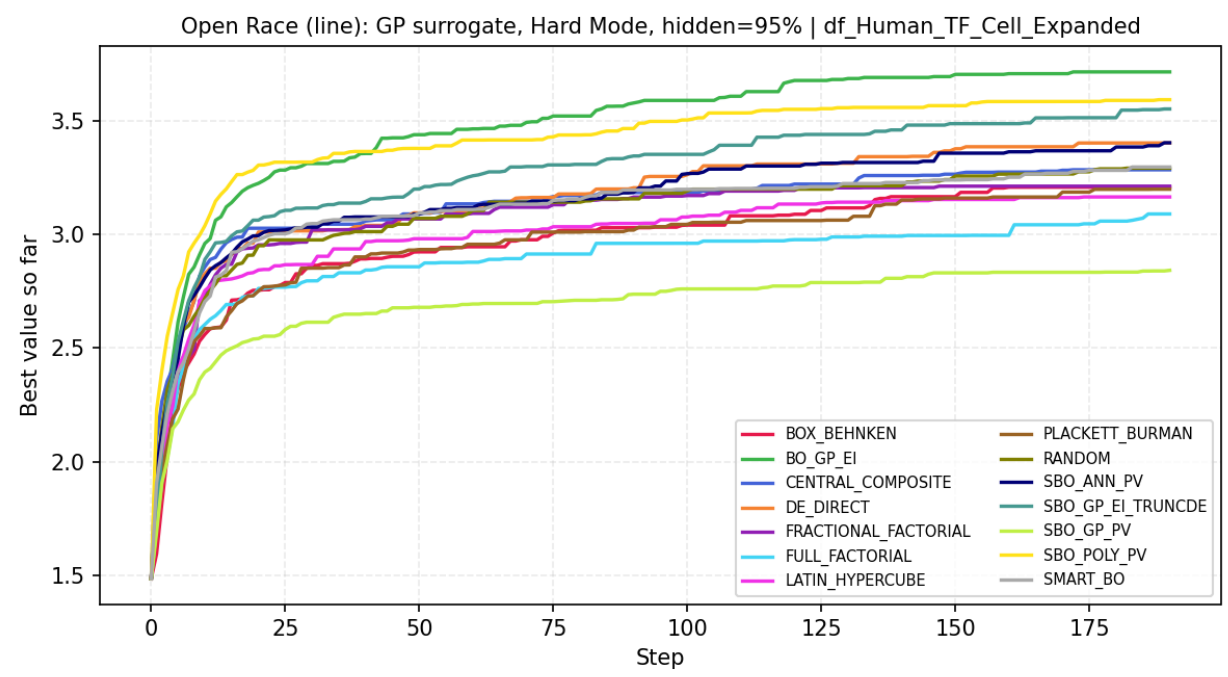

(f) TF-Cell.

Fig. 8. Open Race (OR) competition results. **Configuration:** Hard Mode (GP surrogate, heteroskedastic noise & bimodal perturbations), hidden fraction = 95% (5% initially revealed). **Datasets:** DBO rat myocyte (a), MOBO rat myocyte (b), HeLa regular mode (c), HeLa time-saving mode (d), T-Cell (e), TF-Cell (f). **Metric:** best value found so far over cumulative evaluation steps (higher is better).

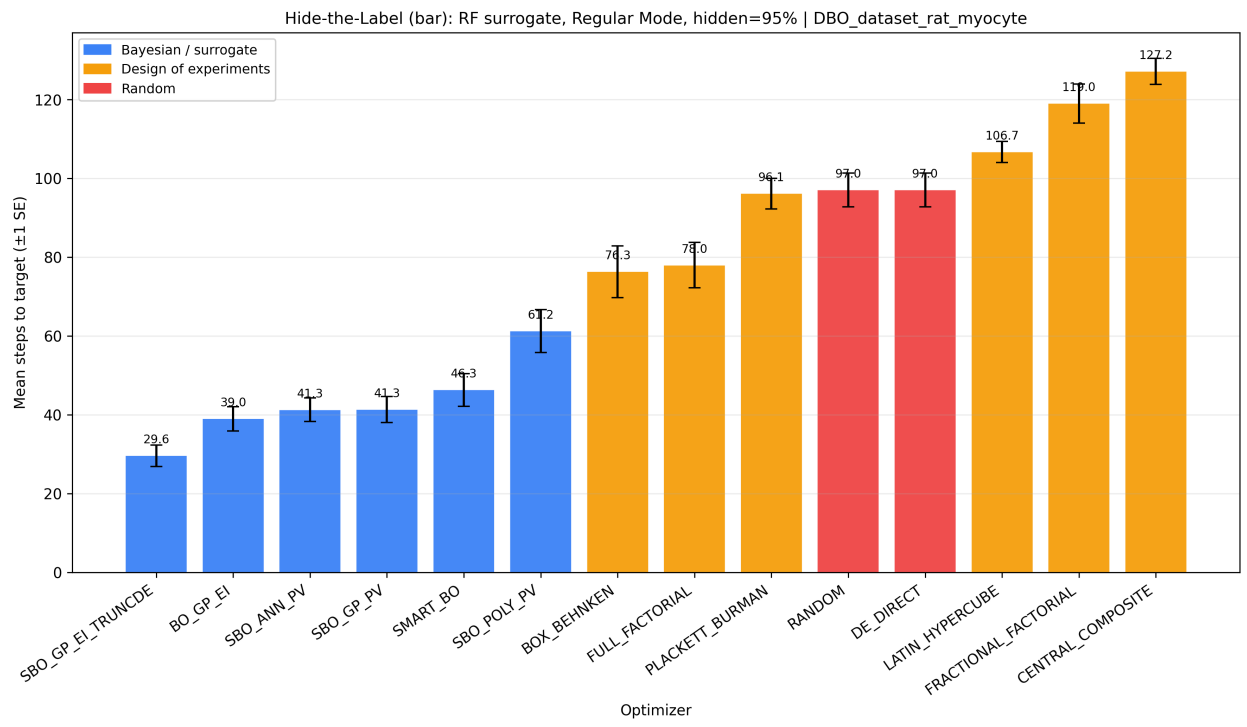

(a) DBO rat myocyte.

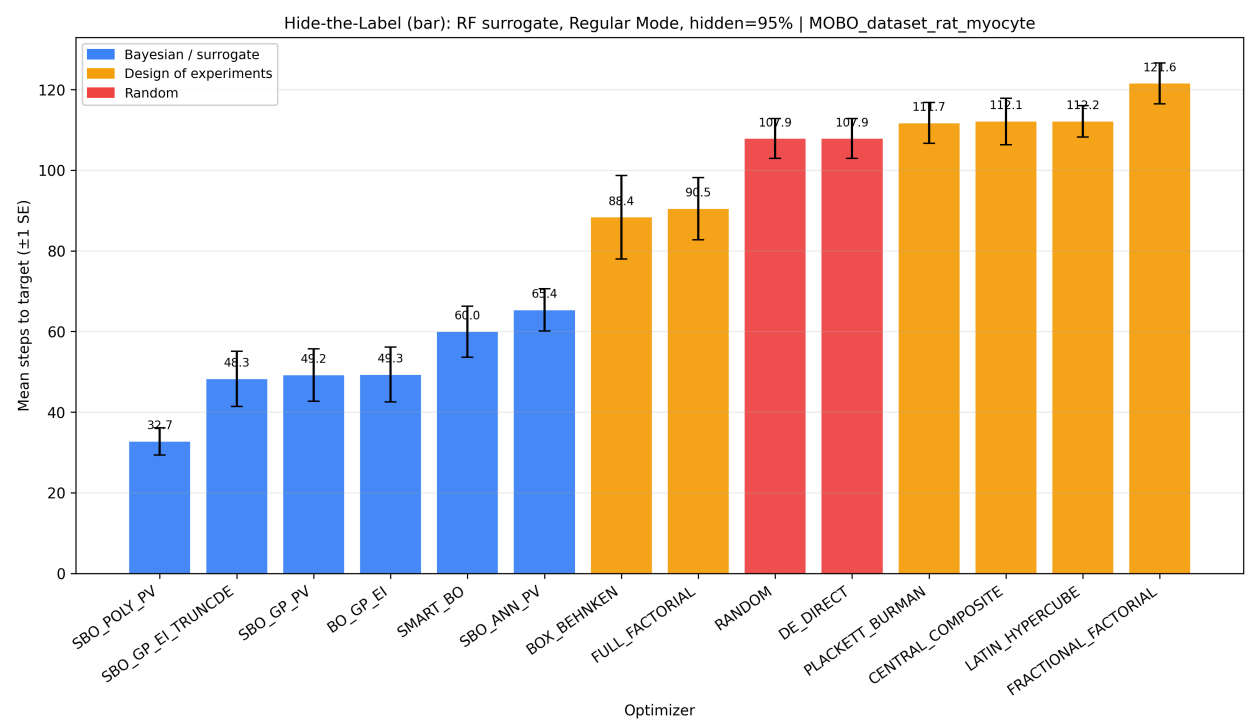

(b) MOBO rat myocyte.

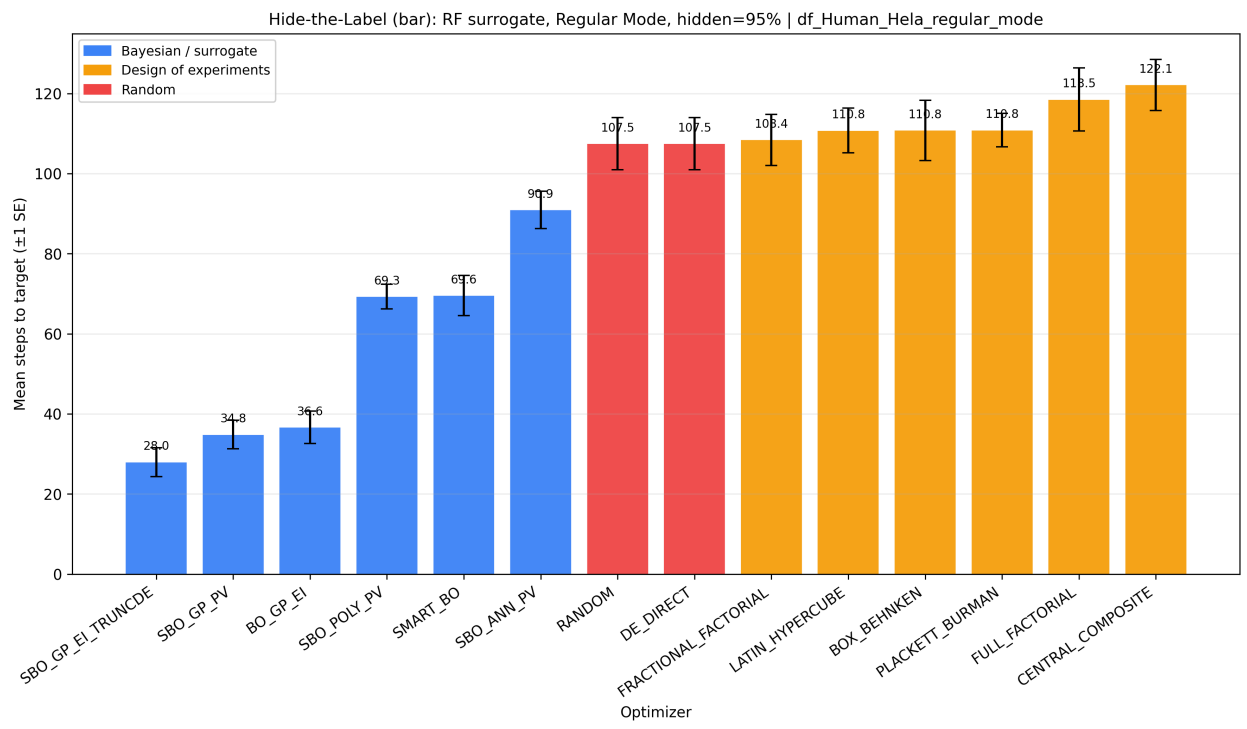

(c) HeLa (regular mode).

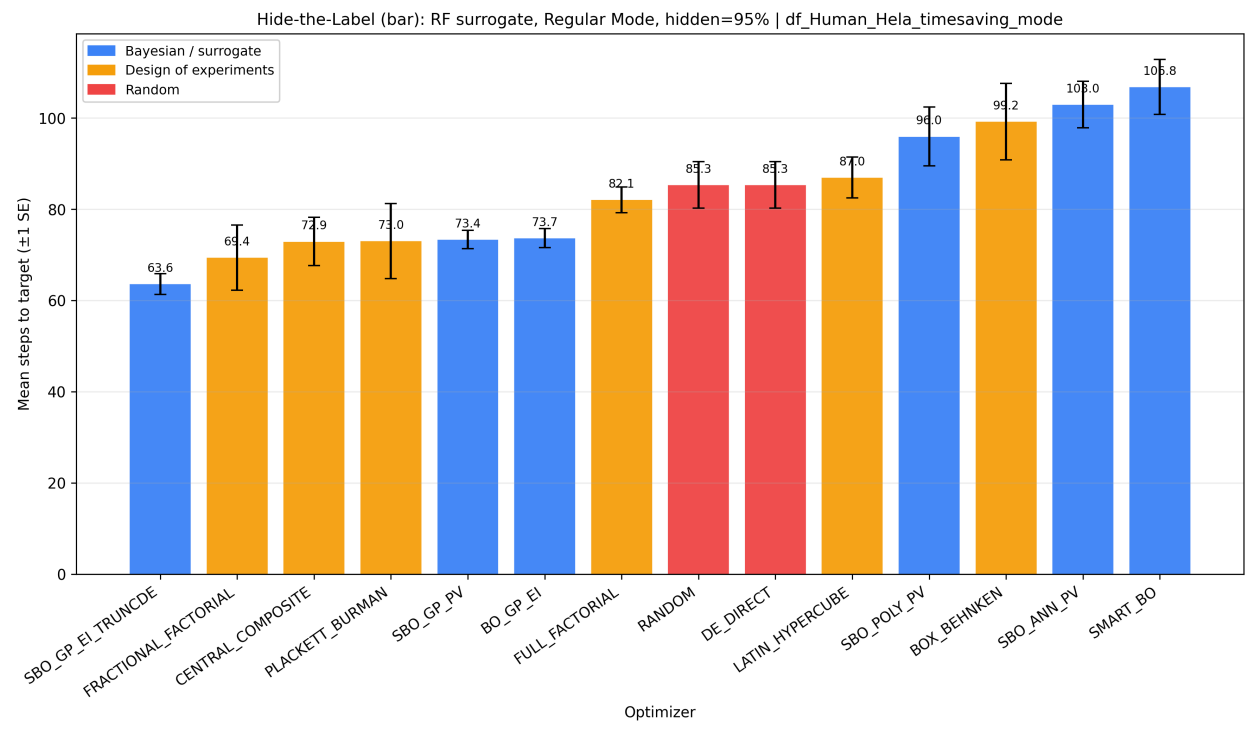

(d) HeLa (time-saving mode).

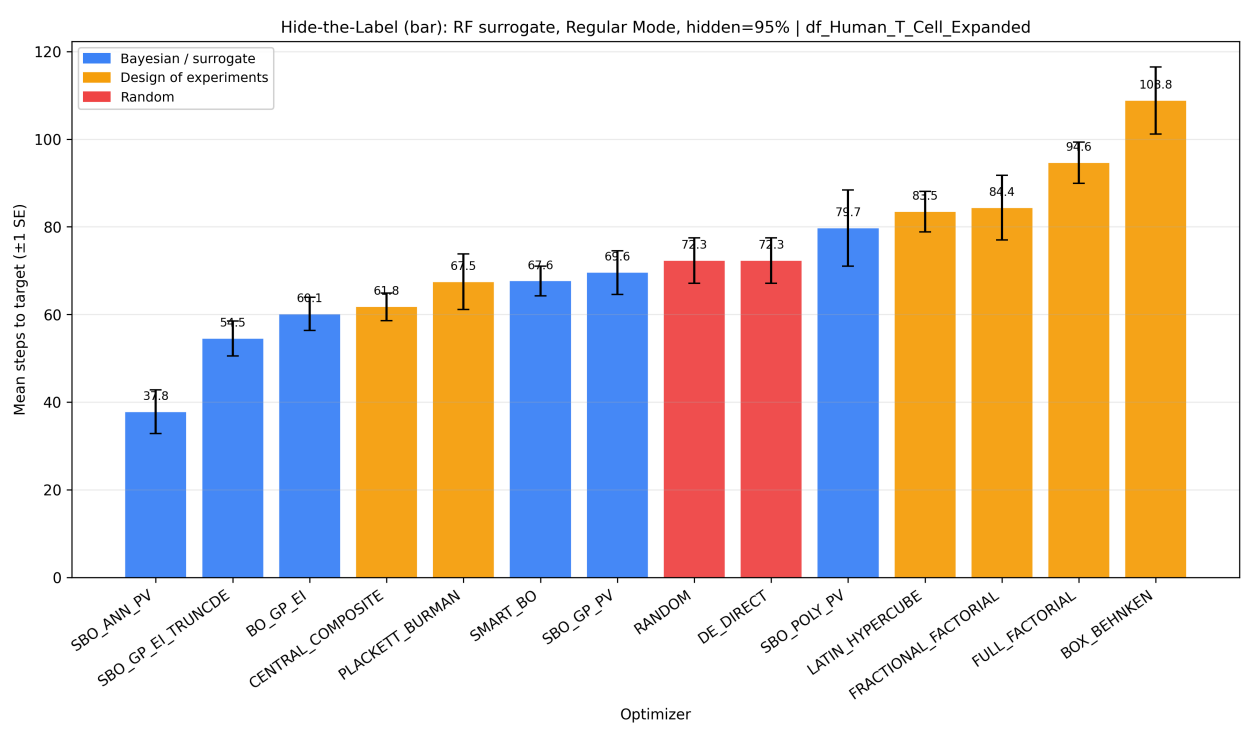

(e) T-Cell.

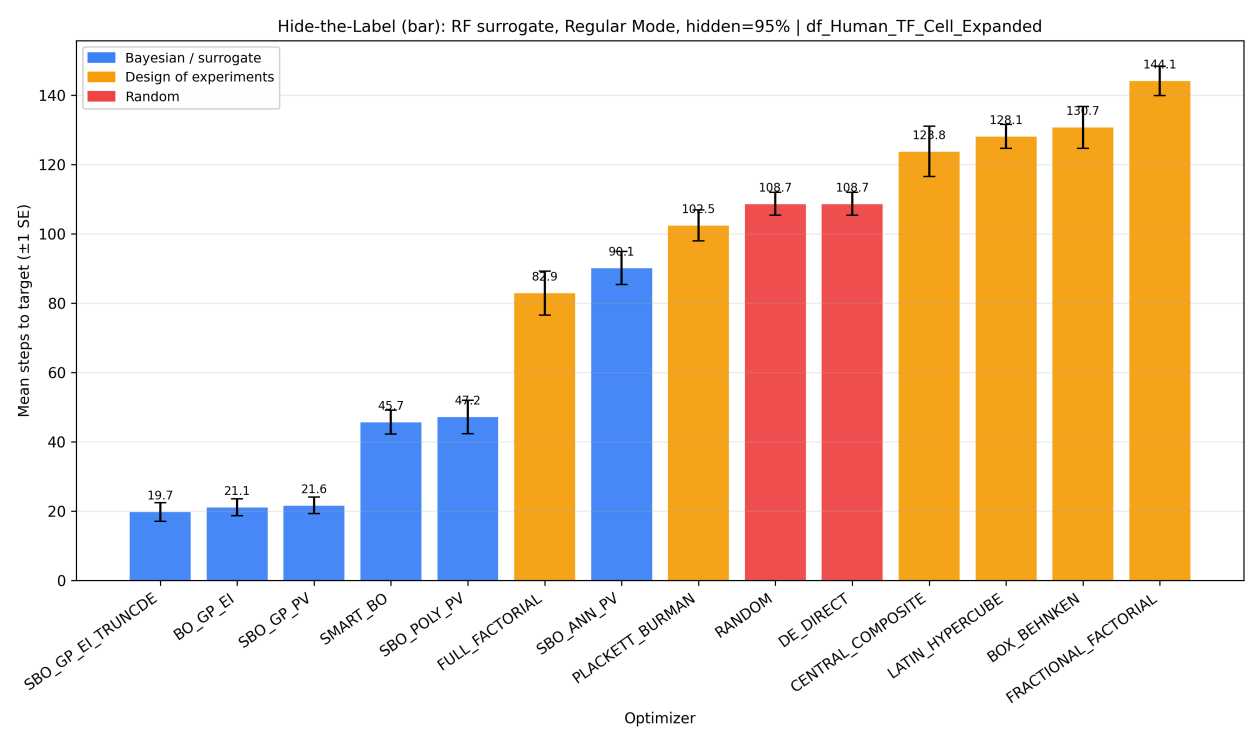

(f) TF-Cell.

Fig. 9. Hide-the-Label (HTL) competition results. **Configuration:** Regular Mode (RF surrogate, low noise), hidden fraction = 95% (5% initially revealed), Batch size = 1. **Datasets:** DBO rat myocyte (a), MOBO rat myocyte (b), HeLa regular mode (c), HeLa time-saving mode (d), T-Cell (e), TF-Cell (f). **Metric:** steps to find target label (lower is better).

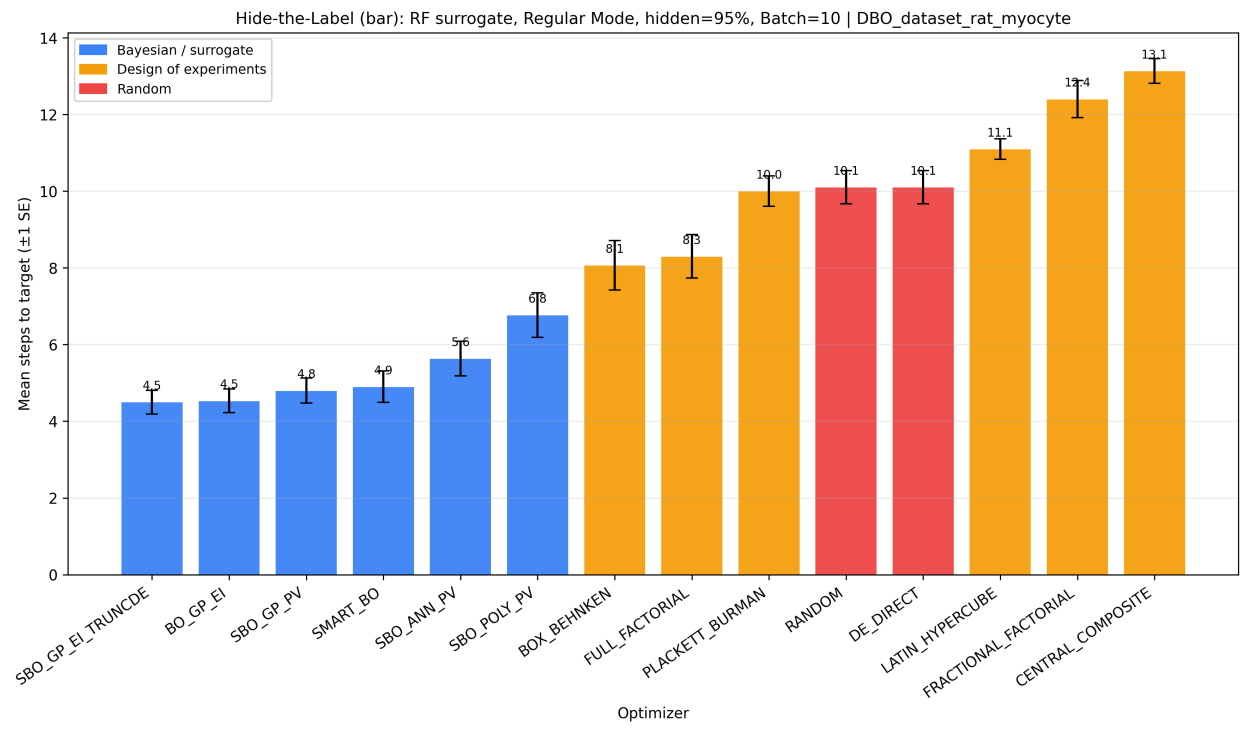

(a) DBO rat myocyte.

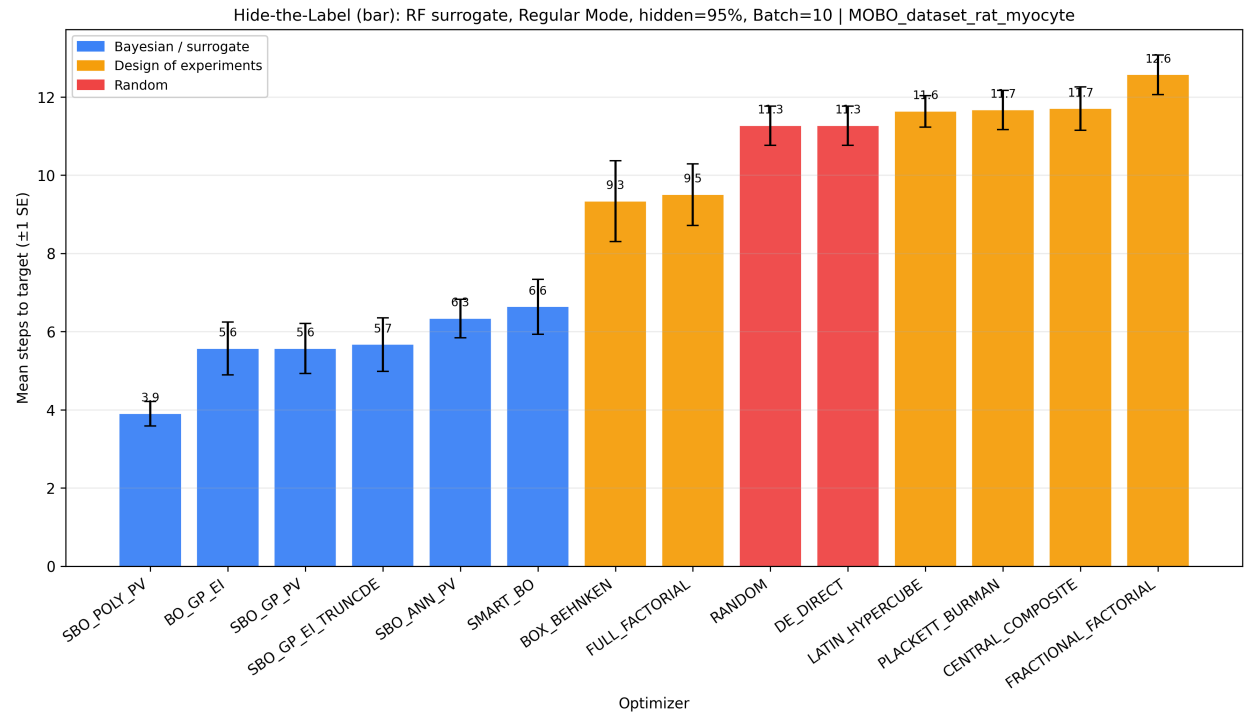

(b) MOBO rat myocyte.

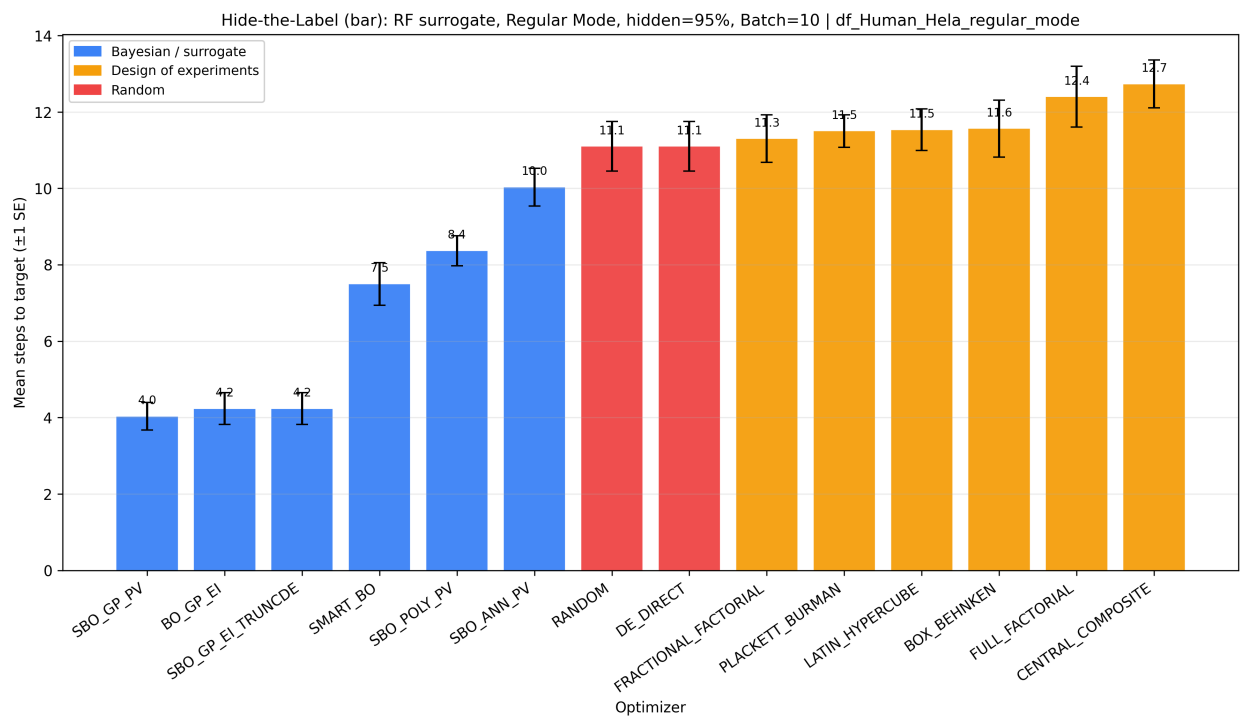

(c) HeLa (regular mode).

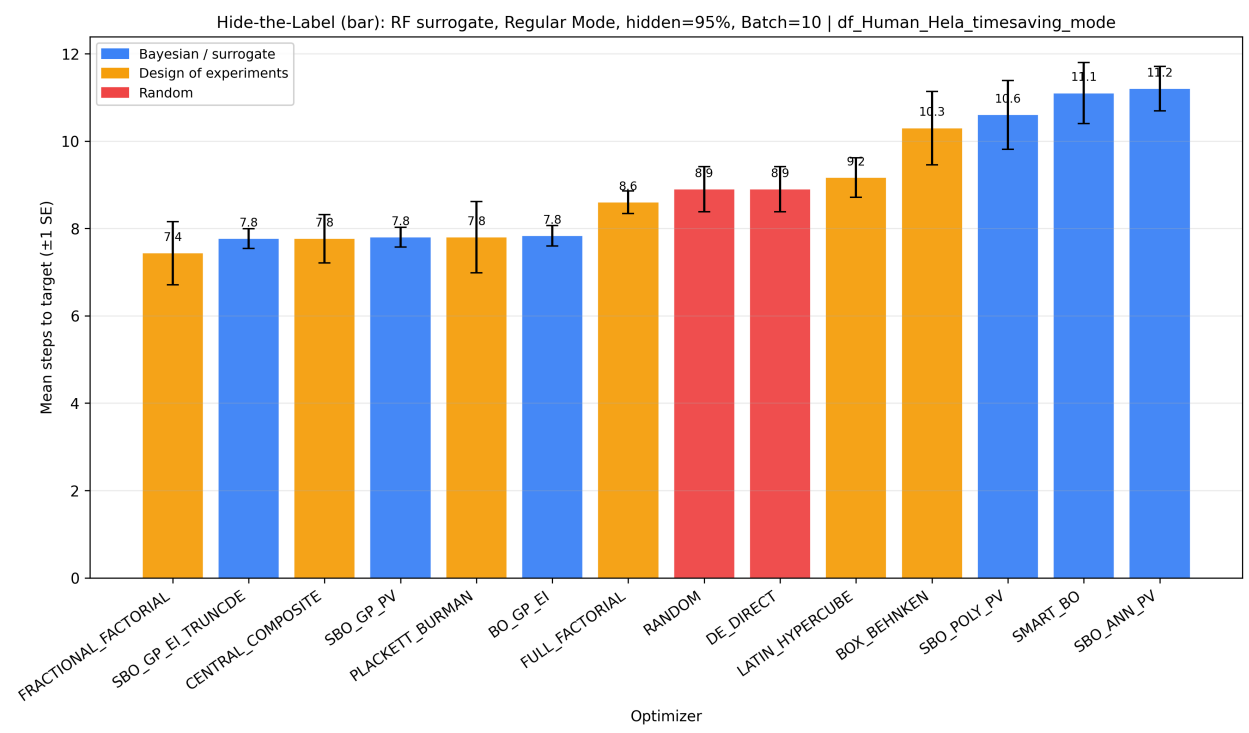

(d) HeLa (time-saving mode).

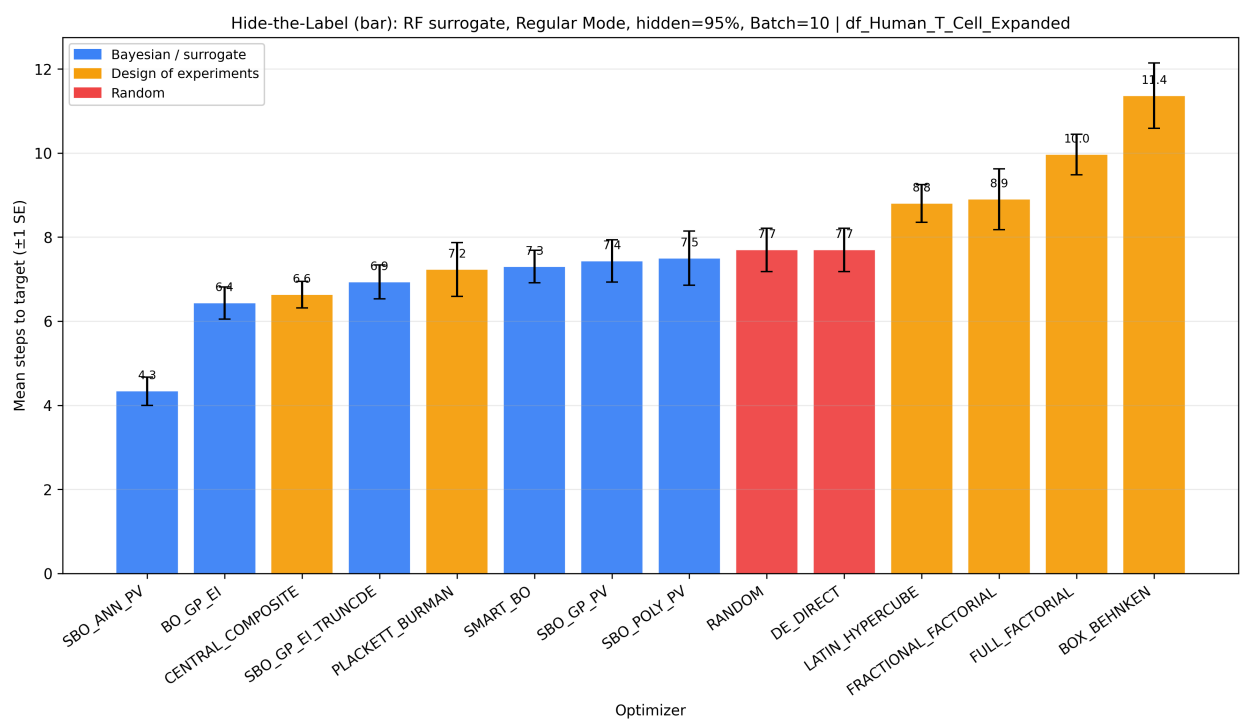

(e) T-Cell.

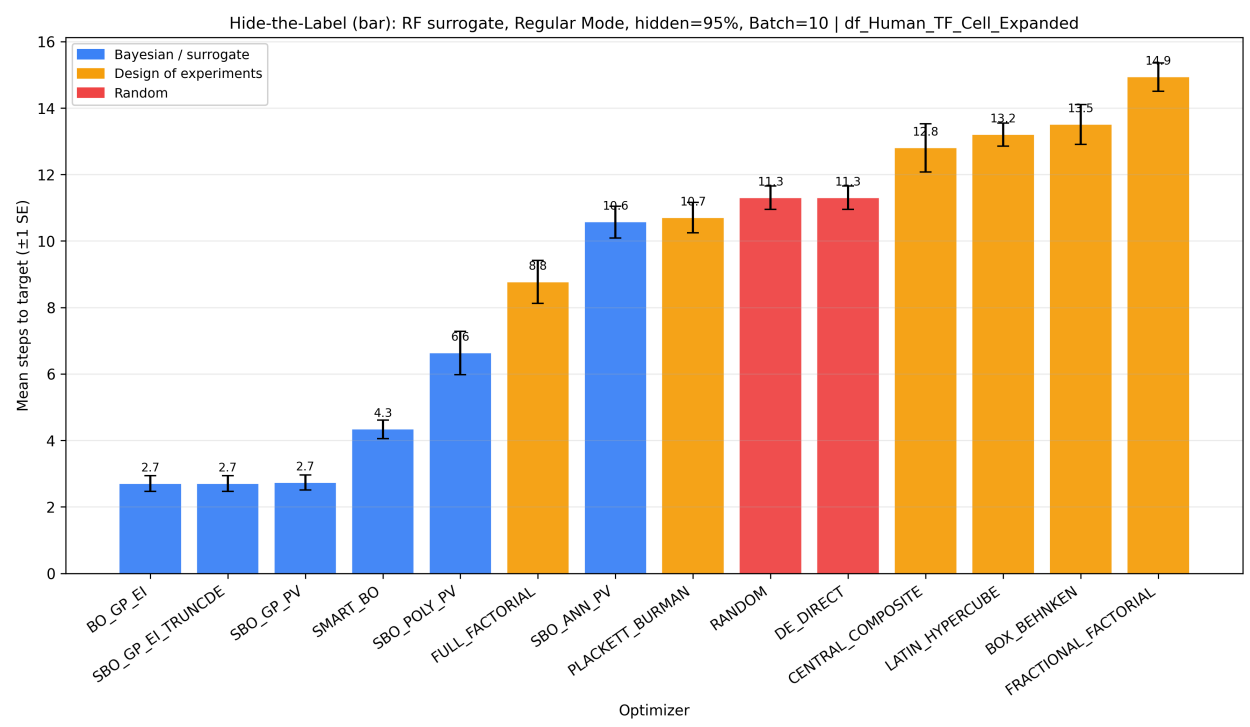

(f) TF-Cell.

Fig. 10. Hide-the-Label (HTL) competition results. **Configuration:** Regular Mode (RF surrogate, low noise), hidden fraction = 95% (5% initially revealed), Batch size = 10. **Datasets:** DBO rat myocyte (a), MOBO rat myocyte (b), HeLa regular mode (c), HeLa time-saving mode (d), T-Cell (e), TF-Cell (f). **Metric:** steps to find target label (lower is better).

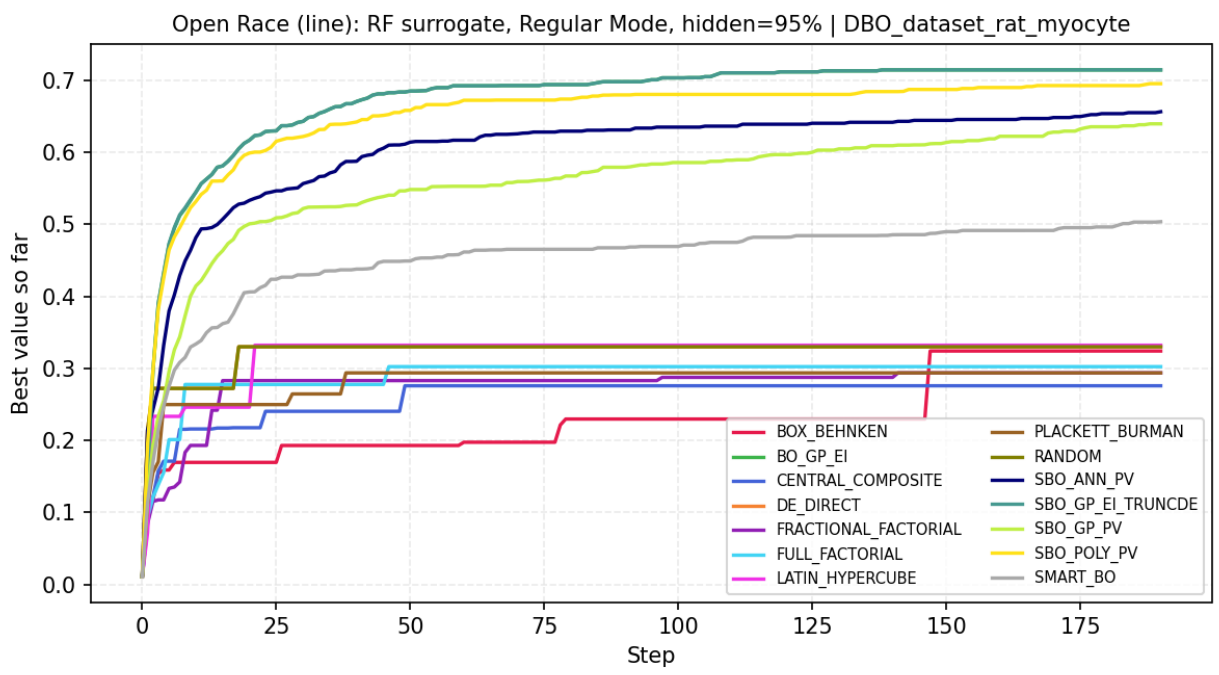

(a) DBO rat myocyte.

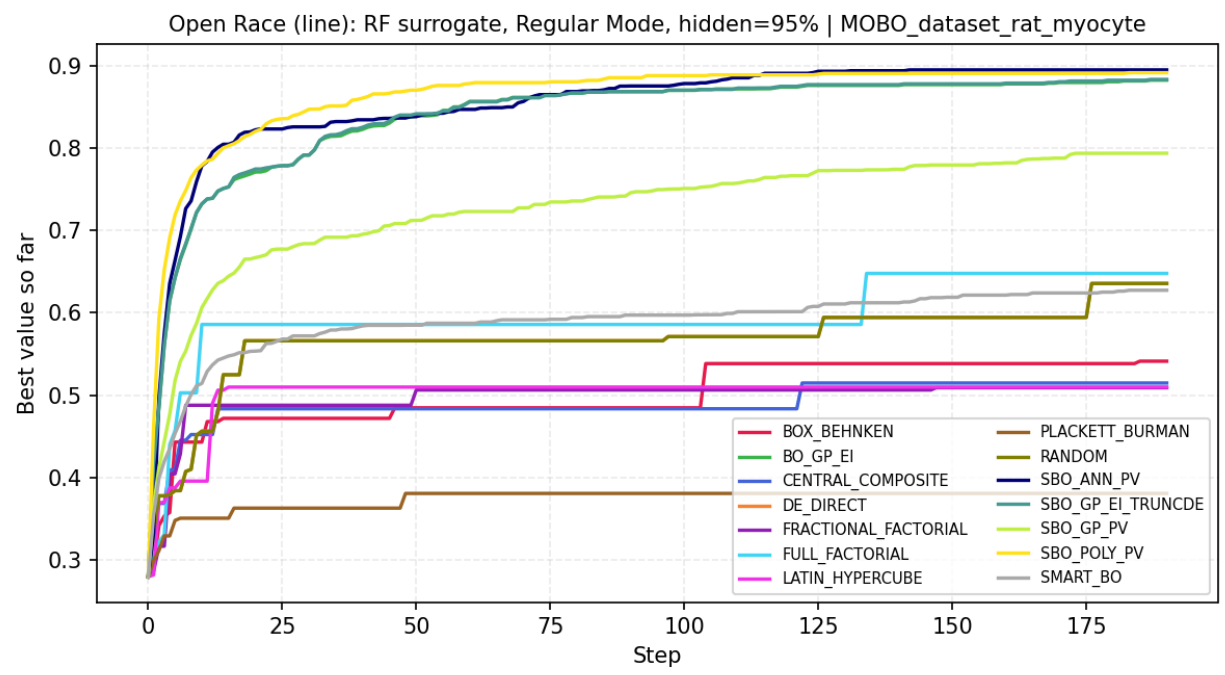

(b) MOBO rat myocyte.

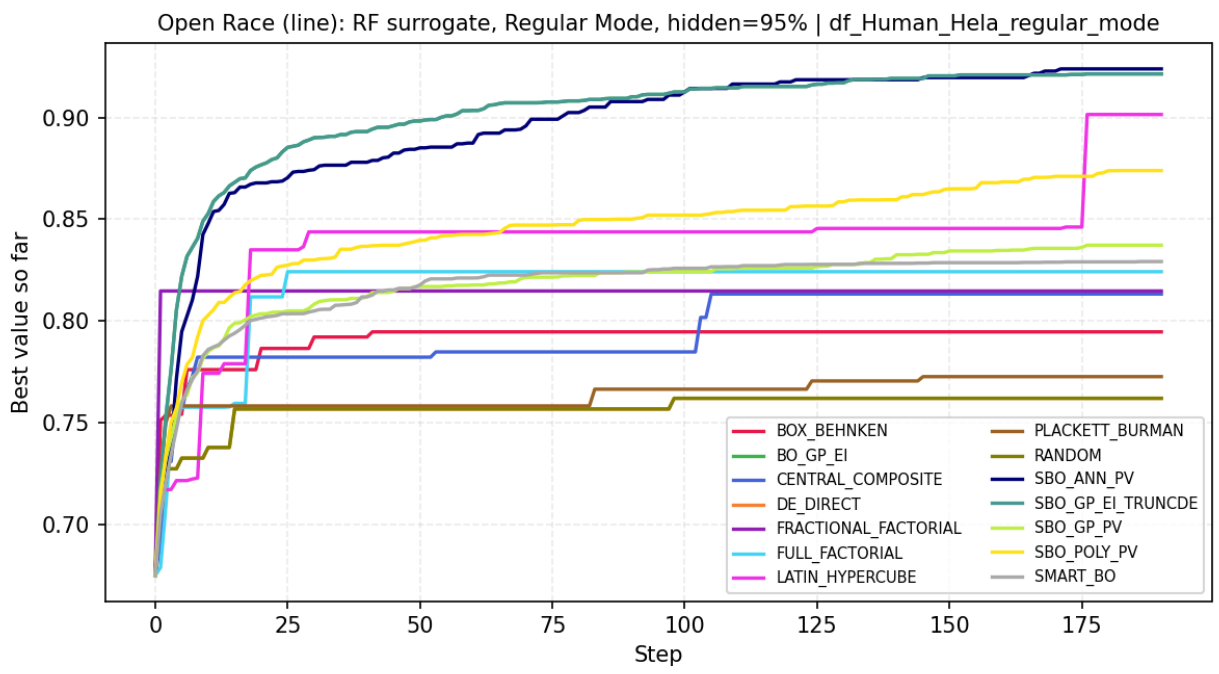

(c) HeLa (regular mode).

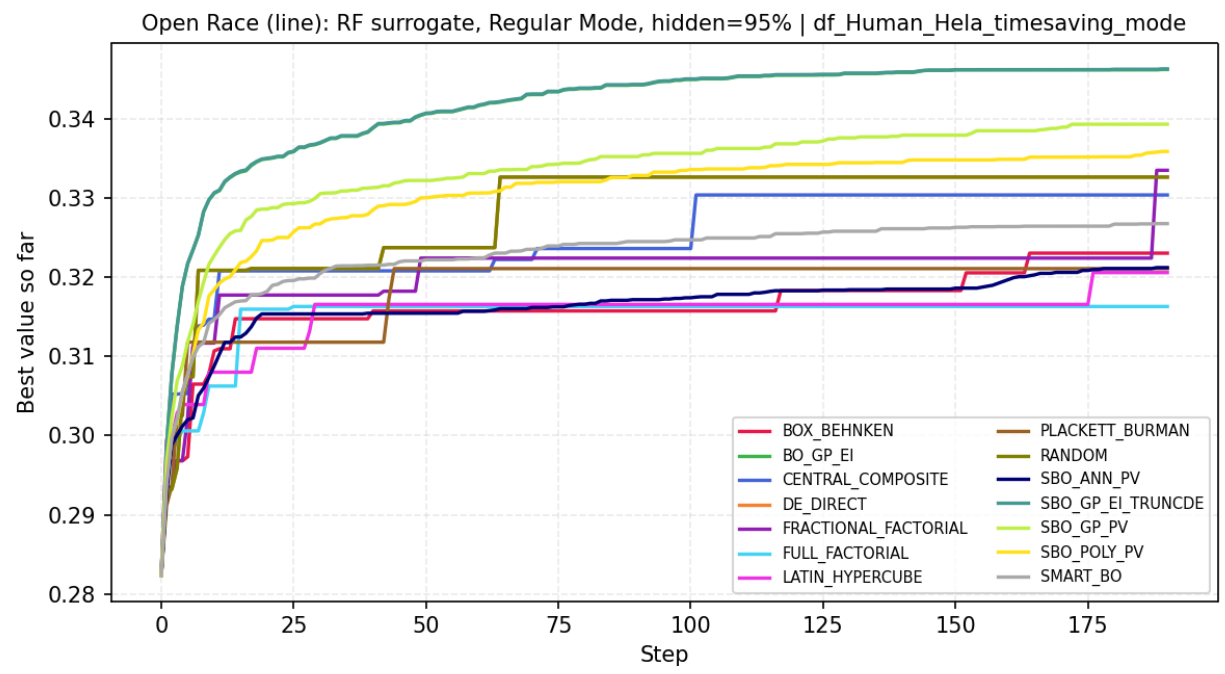

(d) HeLa (time-saving mode).

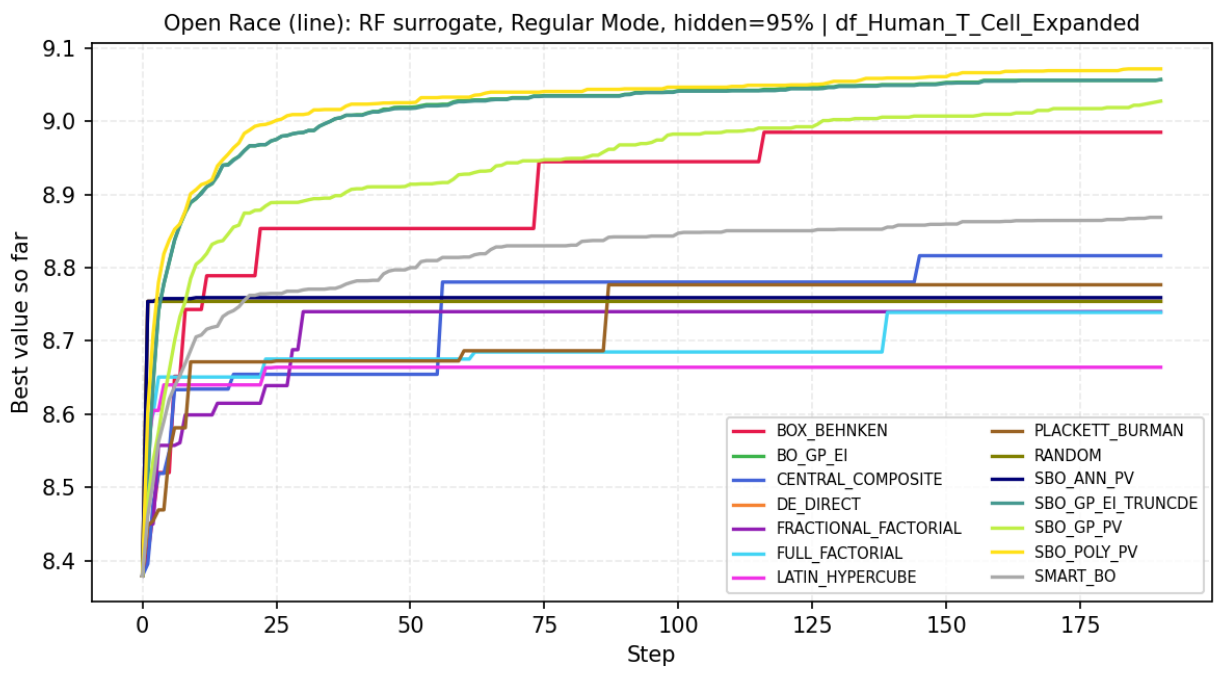

(e) T-Cell.

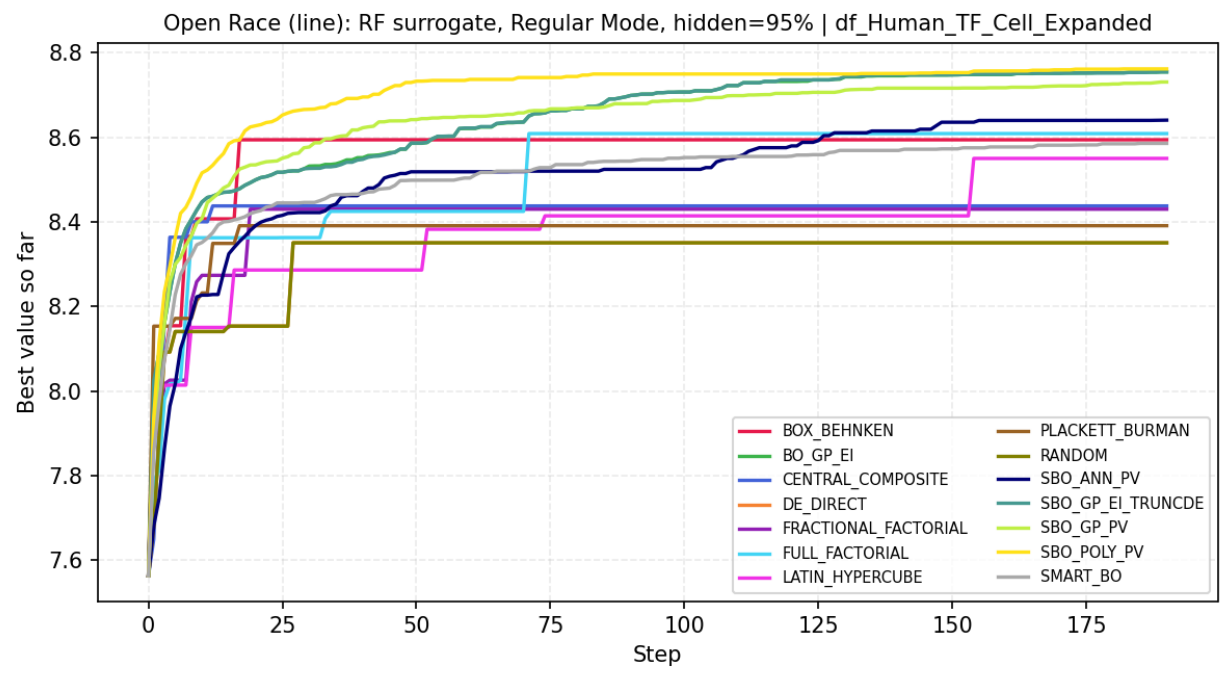

(f) TF-Cell.

Fig. 11. Open Race (OR) competition results. **Configuration:** Regular Mode (RF surrogate, low noise), hidden fraction = 95% (5% initially revealed). **Datasets:** DBO rat myocyte (a), MOBO rat myocyte (b), HeLa regular mode (c), HeLa time-saving mode (d), T-Cell (e), TF-Cell (f). **Metric:** best value found so far over cumulative evaluation steps (higher is better).

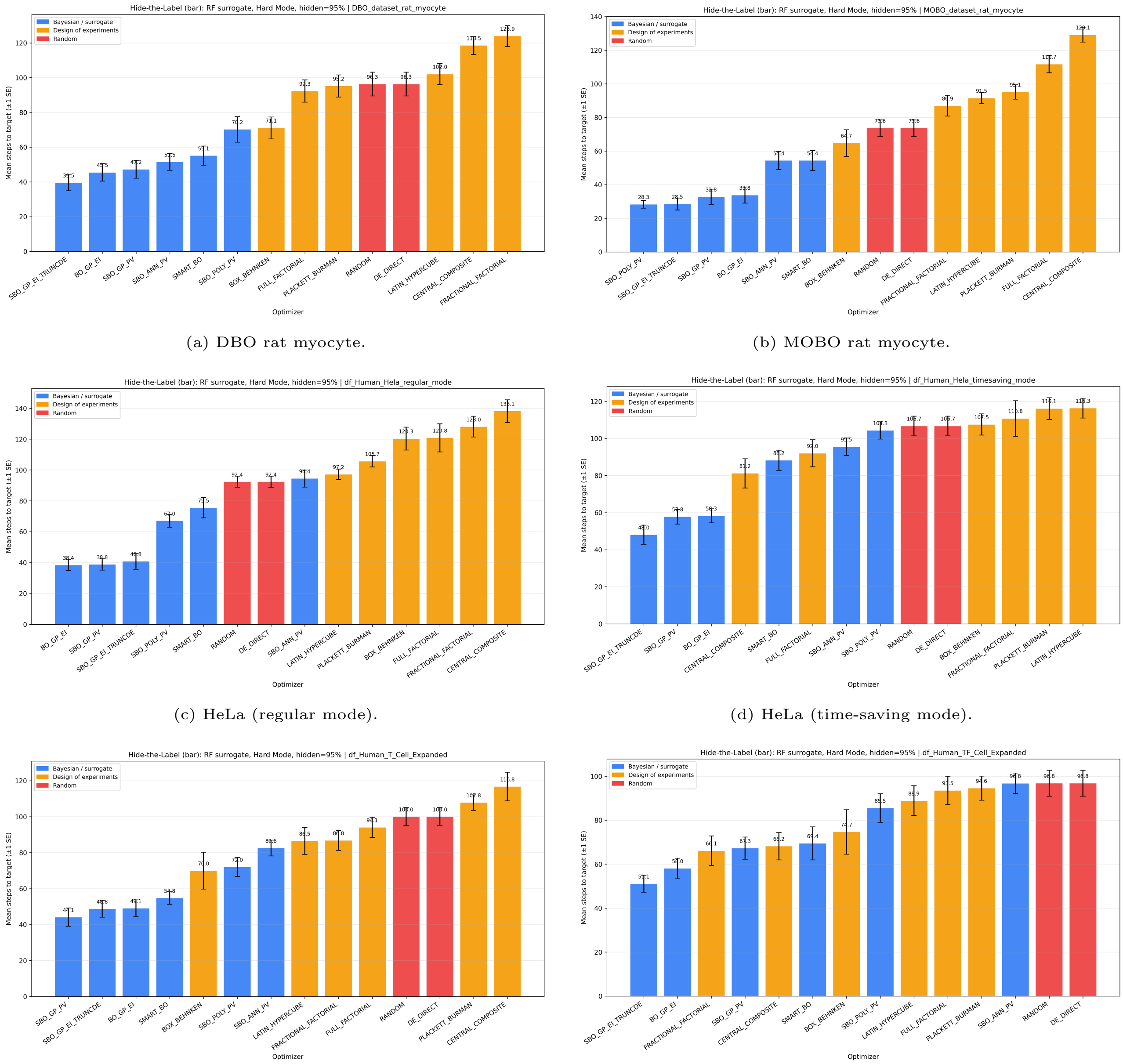

(a) DBO rat myocyte.

(b) MOBO rat myocyte.

(c) HeLa (regular mode).

(d) HeLa (time-saving mode).

(e) T-Cell.

(f) TF-Cell.

Fig. 12. Hide-the-Label (HTL) competition results. **Configuration:** Hard Mode (RF surrogate, heteroskedastic noise & bimodal perturbations), hidden fraction = 95% (5% initially revealed), Batch size = 1. **Datasets:** DBO rat myocyte (a), MOBO rat myocyte (b), HeLa regular mode (c), HeLa time-saving mode (d), T-Cell (e), TF-Cell (f). **Metric:** steps to find target label (lower is better).

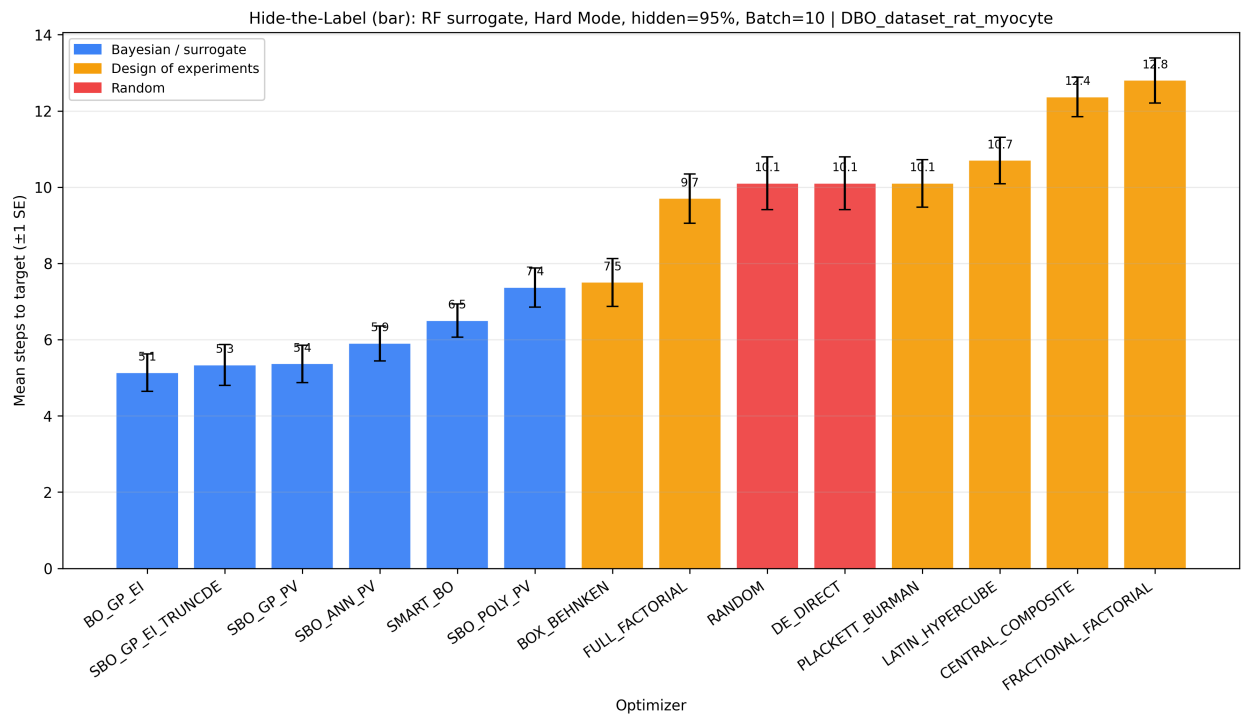

(a) DBO rat myocyte.

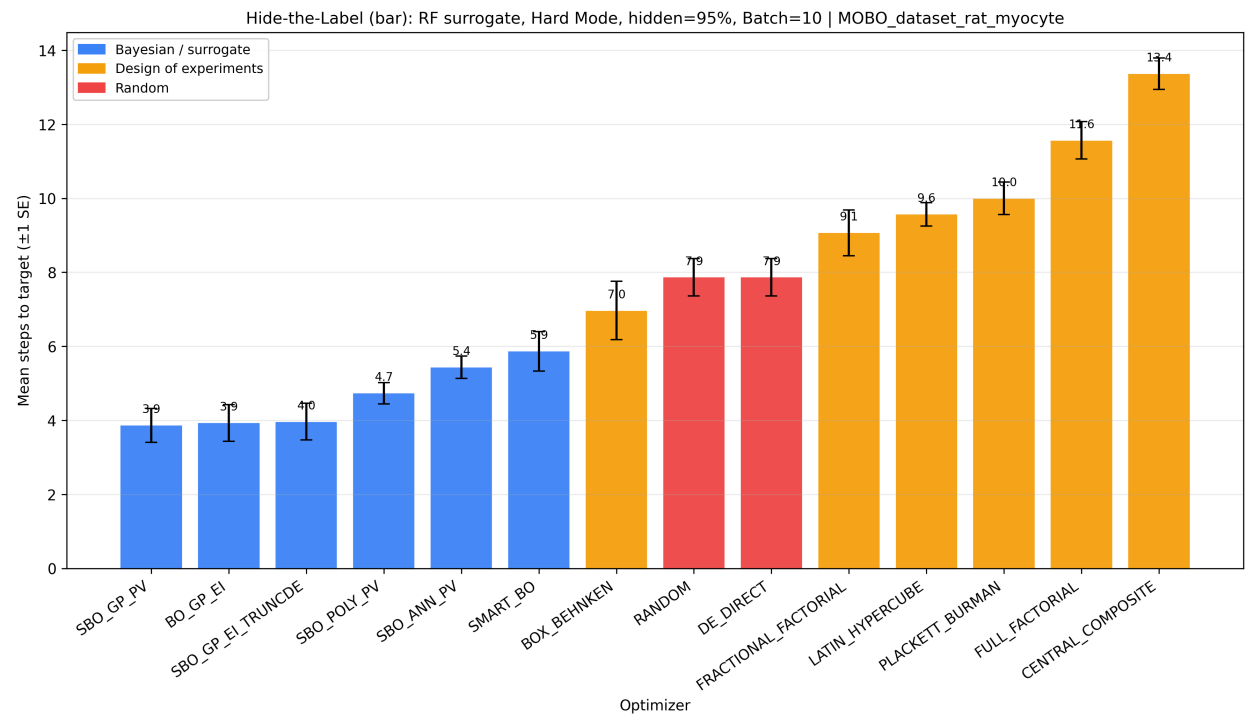

(b) MOBO rat myocyte.

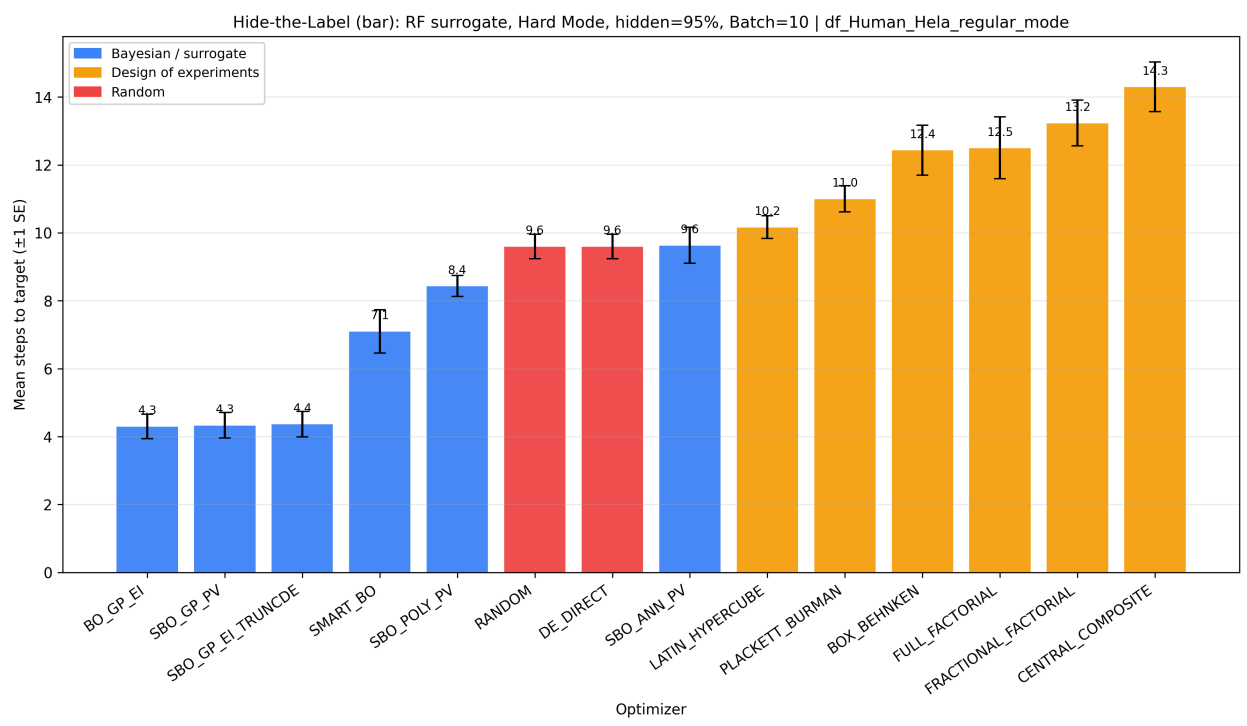

(c) HeLa (regular mode).

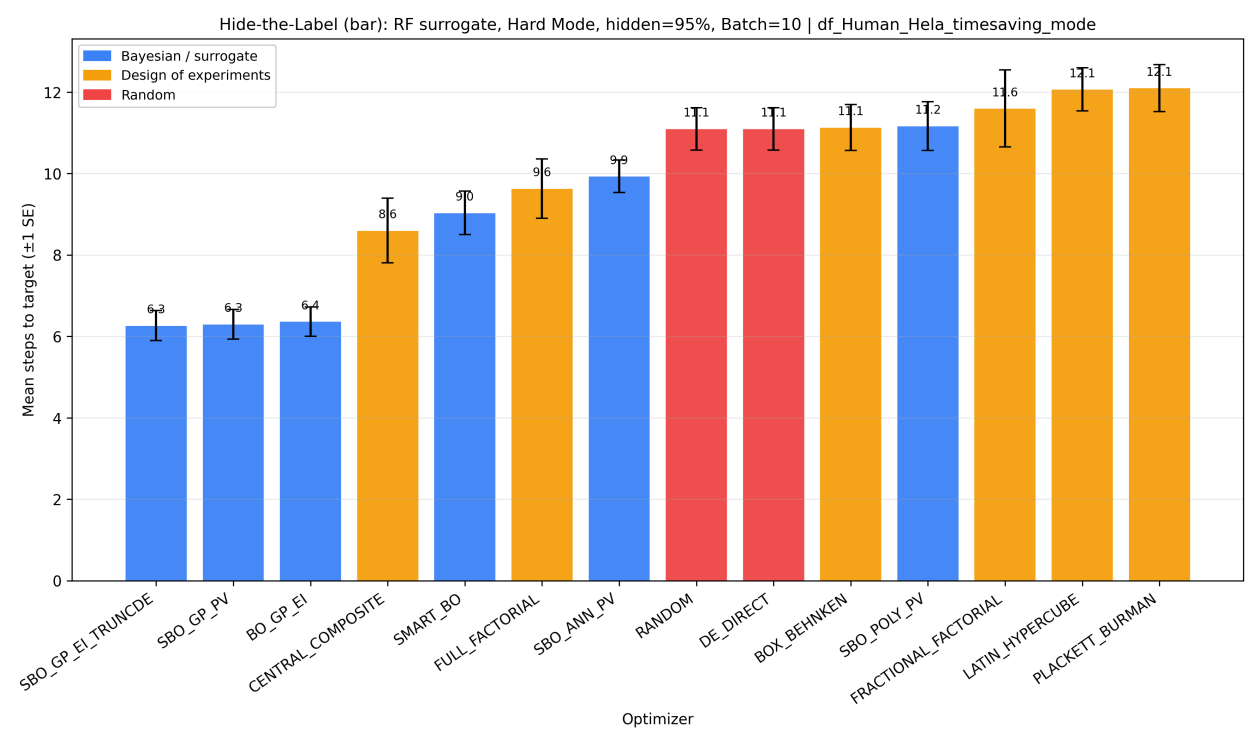

(d) HeLa (time-saving mode).

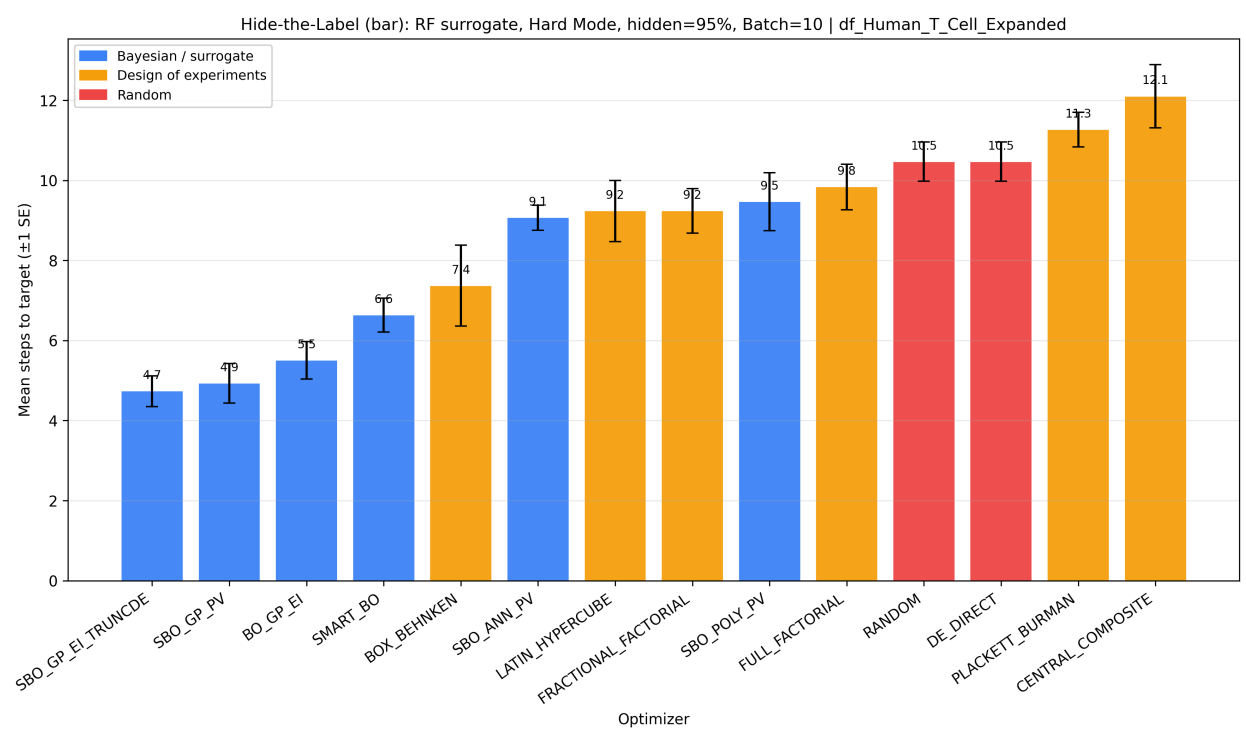

(e) T-Cell.

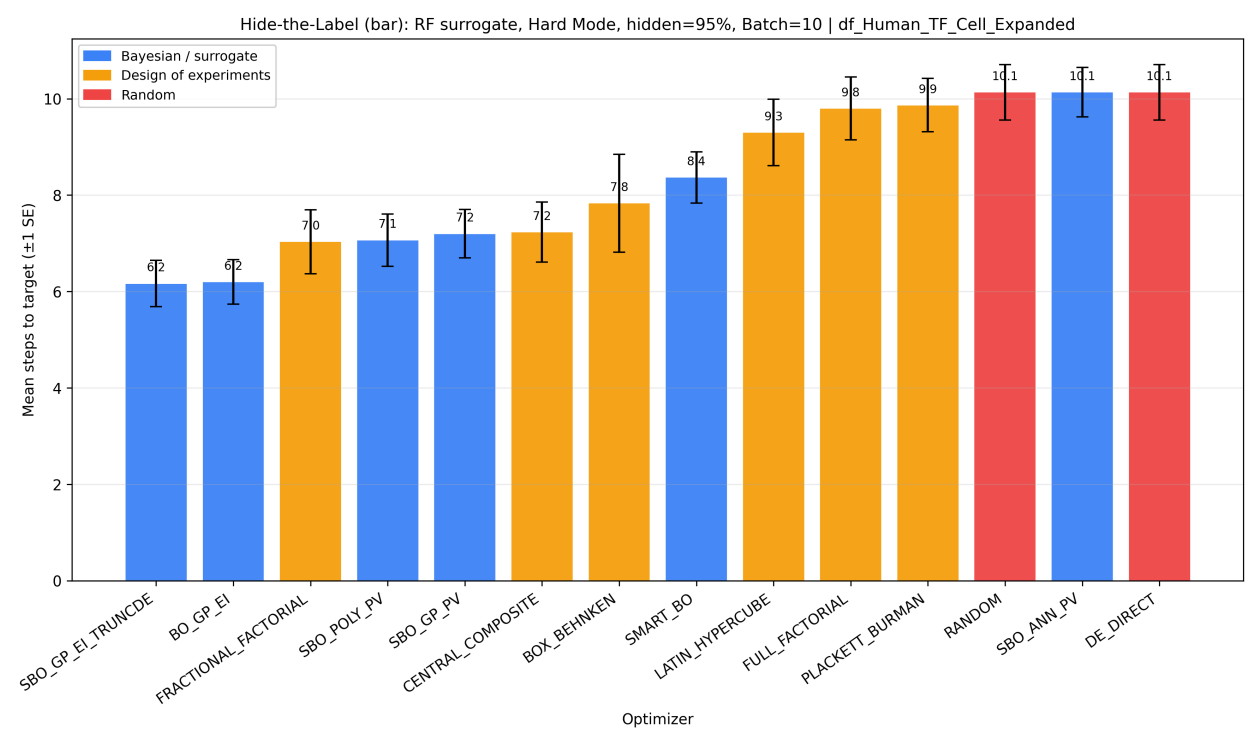

(f) TF-Cell.

Fig. 13. Hide-the-Label (HTL) competition results. **Configuration:** Hard Mode (RF surrogate, heteroskedastic noise & bimodal perturbations), hidden fraction = 95% (5% initially revealed), Batch size = 10. **Datasets:** DBO rat myocyte (a), MOBO rat myocyte (b), HeLa regular mode (c), HeLa time-saving mode (d), T-Cell (e), TF-Cell (f). **Metric:** steps to find target label (lower is better).

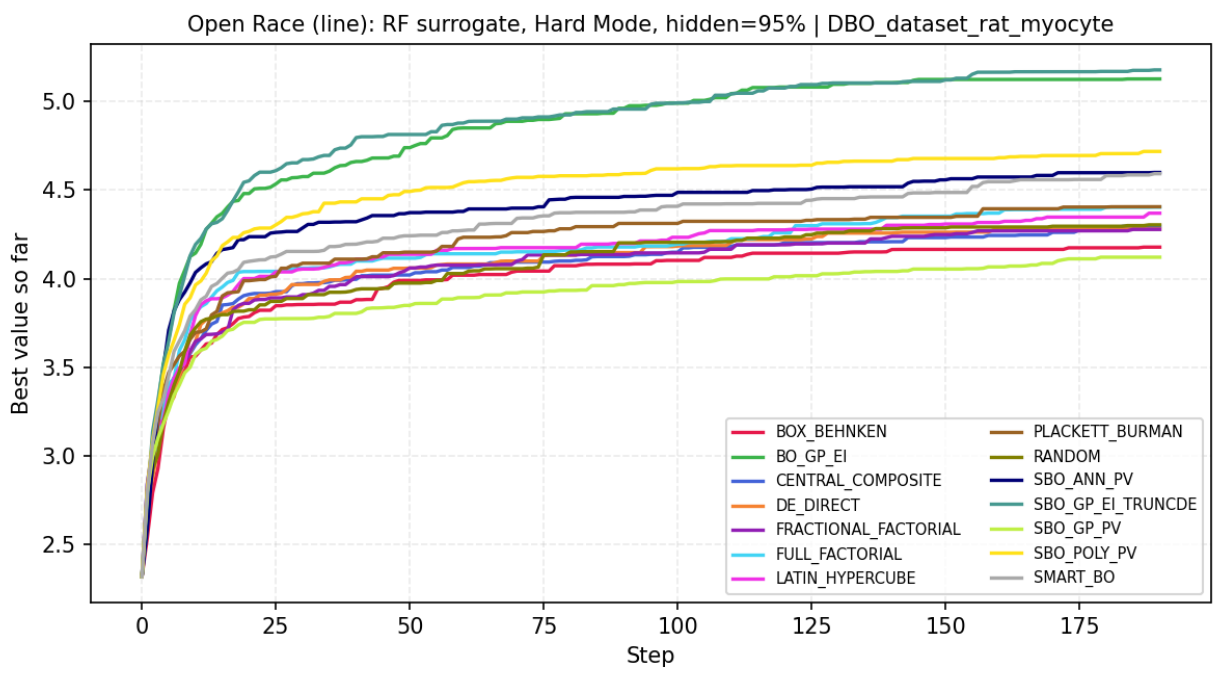

(a) DBO rat myocyte.

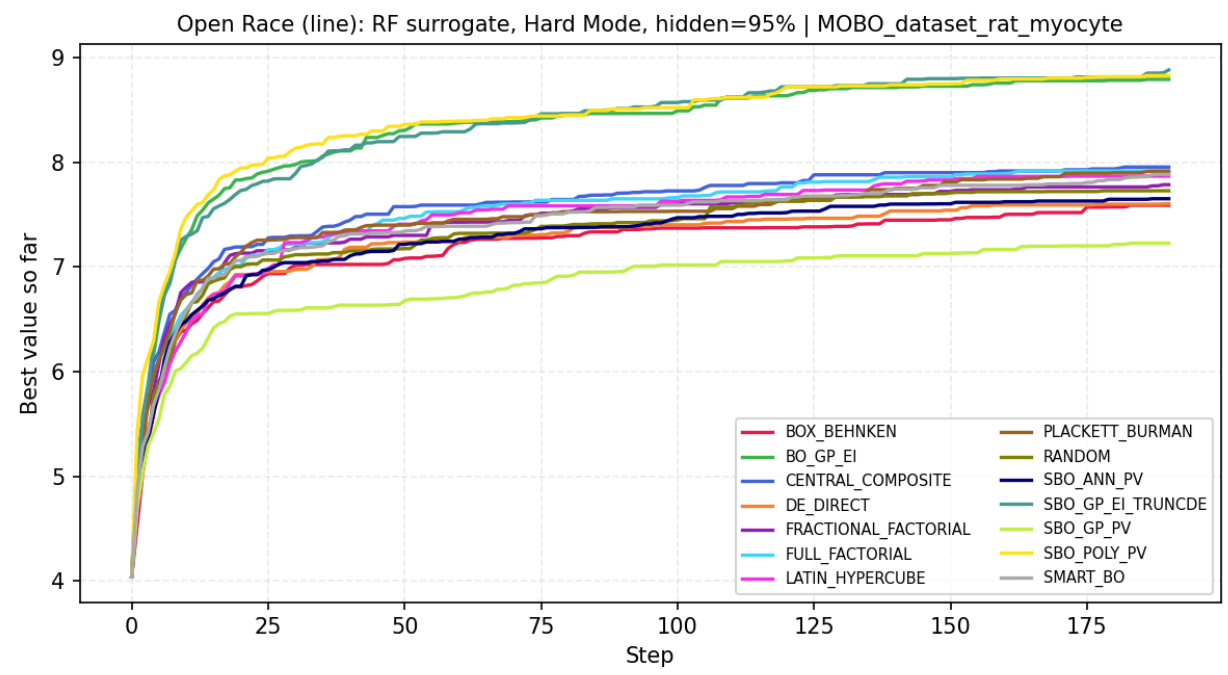

(b) MOBO rat myocyte.

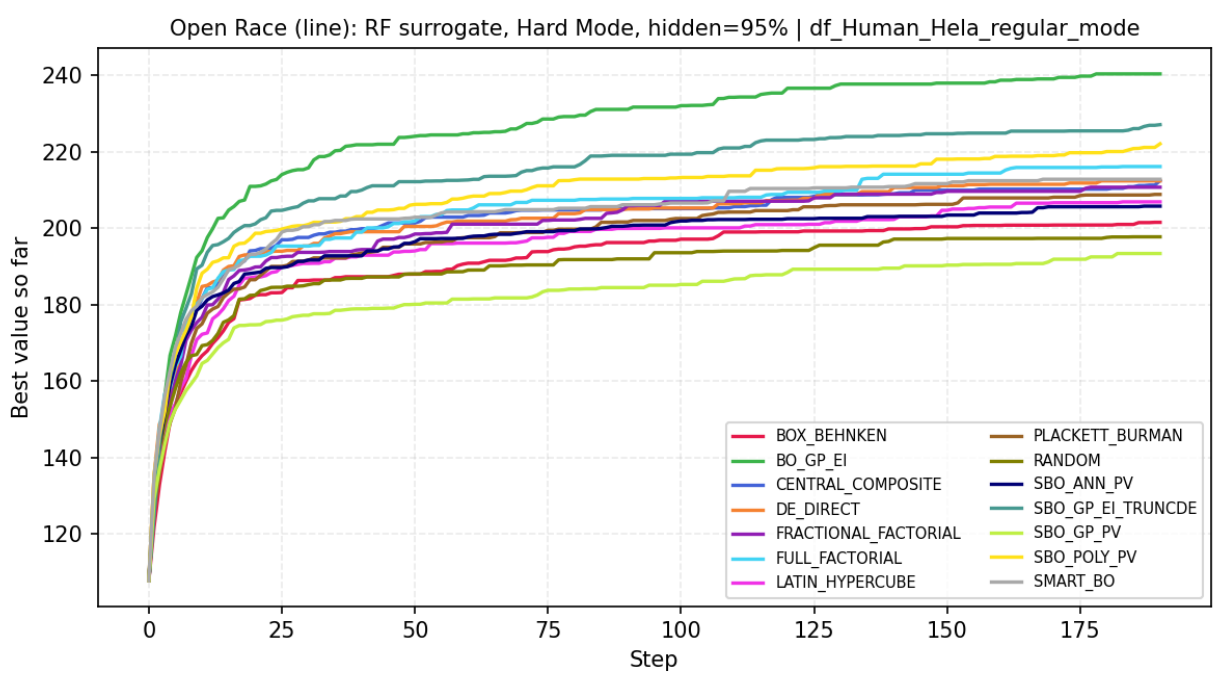

(c) HeLa (regular mode).

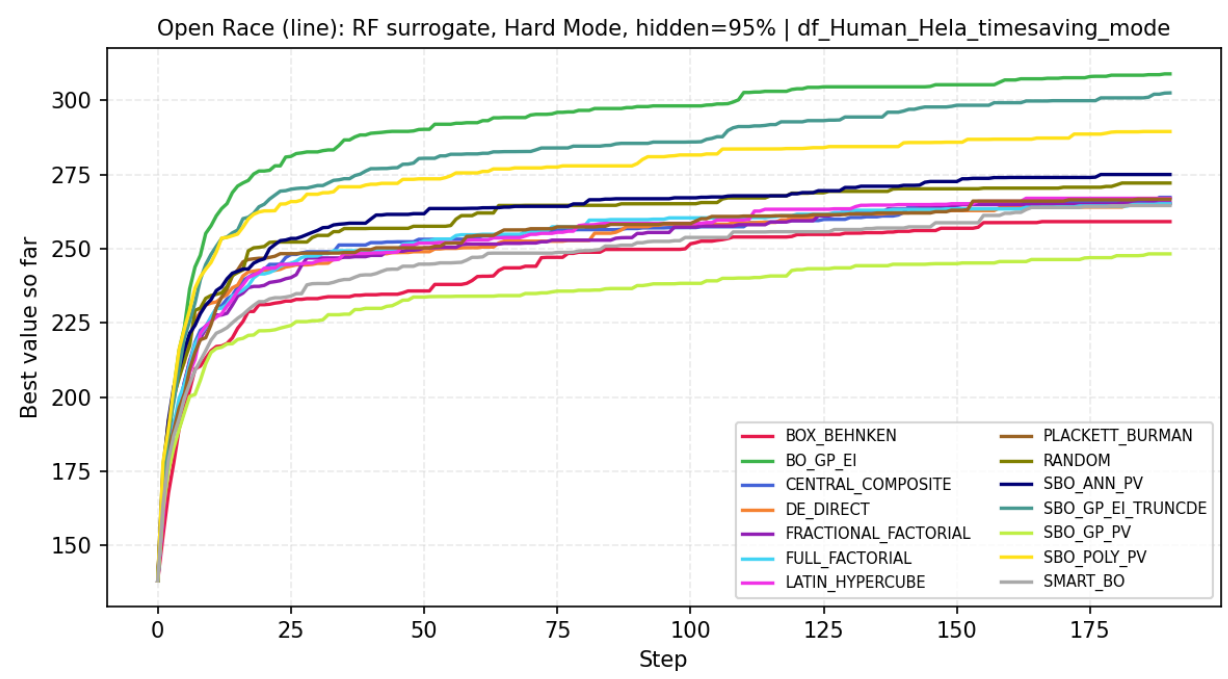

(d) HeLa (time-saving mode).

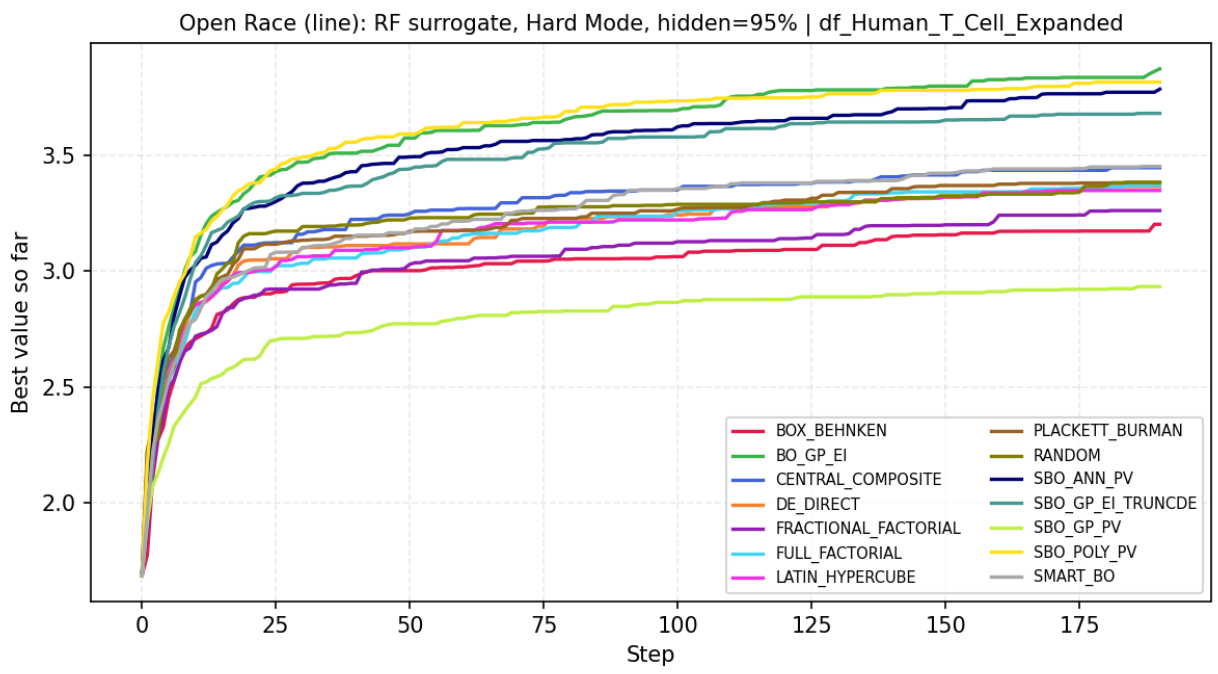

(e) T-Cell.

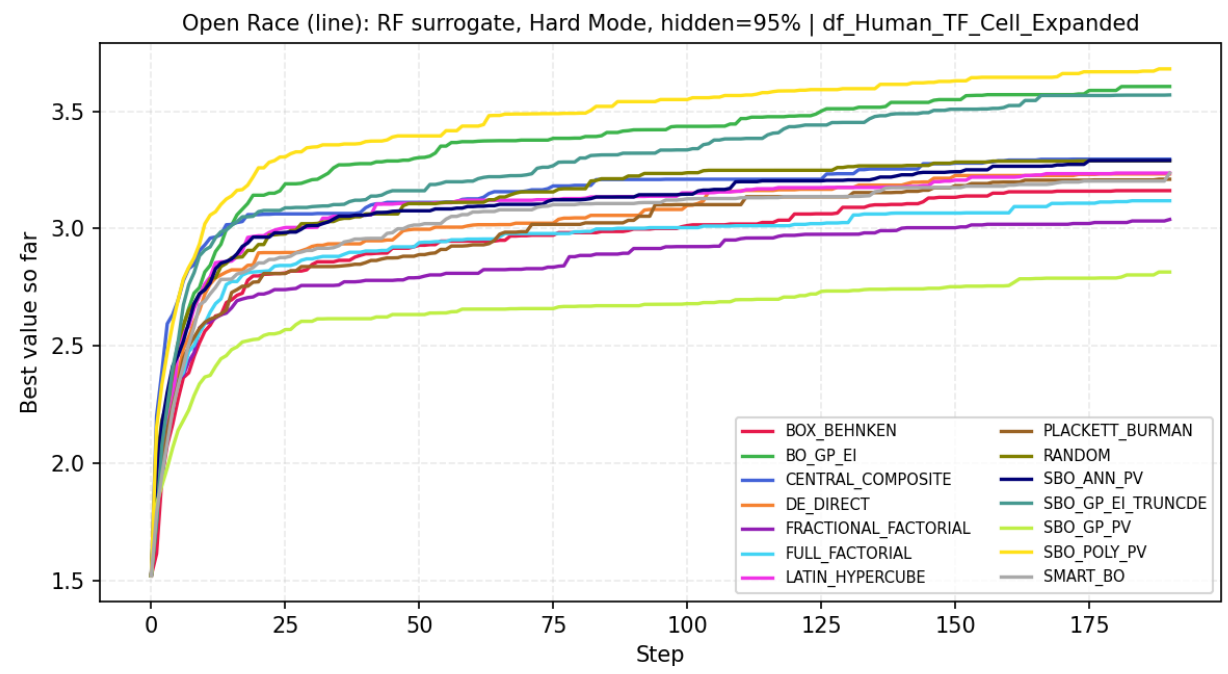

(f) TF-Cell.

Fig. 14. Open Race (OR) competition results. **Configuration:** Hard Mode (RF surrogate, heteroskedastic noise & bimodal perturbations), hidden fraction = 95% (5% initially revealed). **Datasets:** DBO rat myocyte (a), MOBO rat myocyte (b), HeLa regular mode (c), HeLa time-saving mode (d), T-Cell (e), TF-Cell (f). **Metric:** best value found so far over cumulative evaluation steps (higher is better).

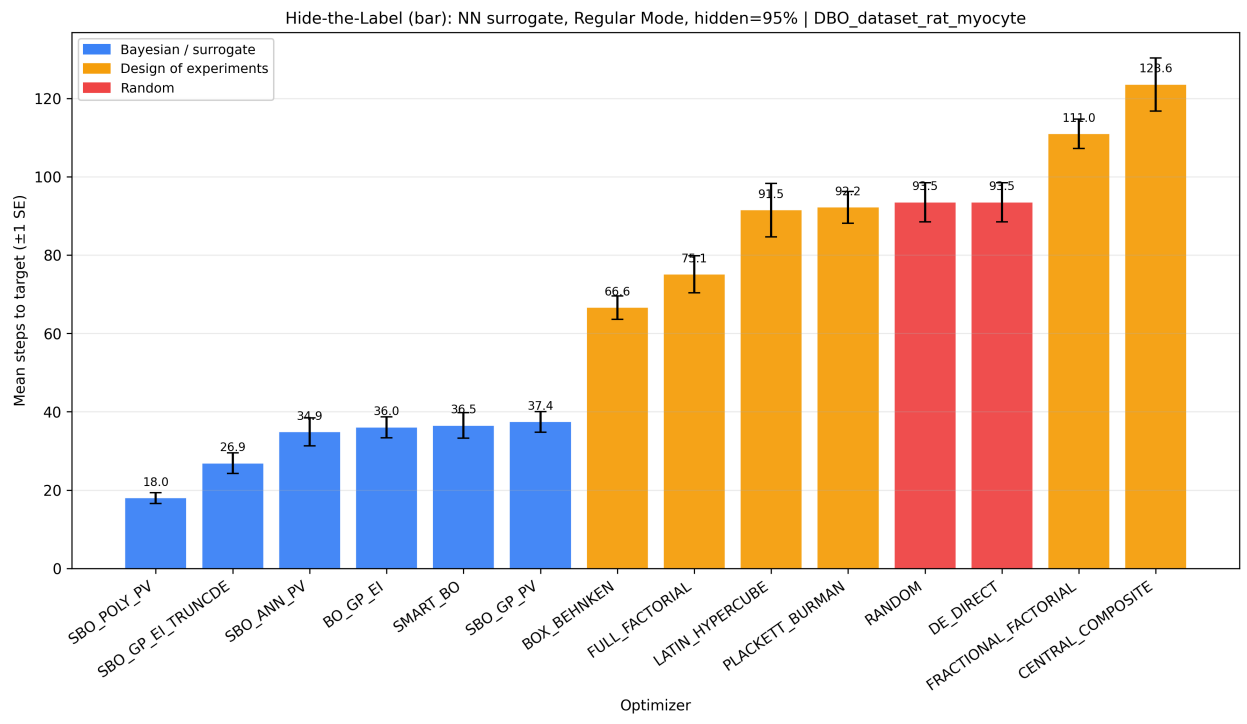

(a) DBO rat myocyte.

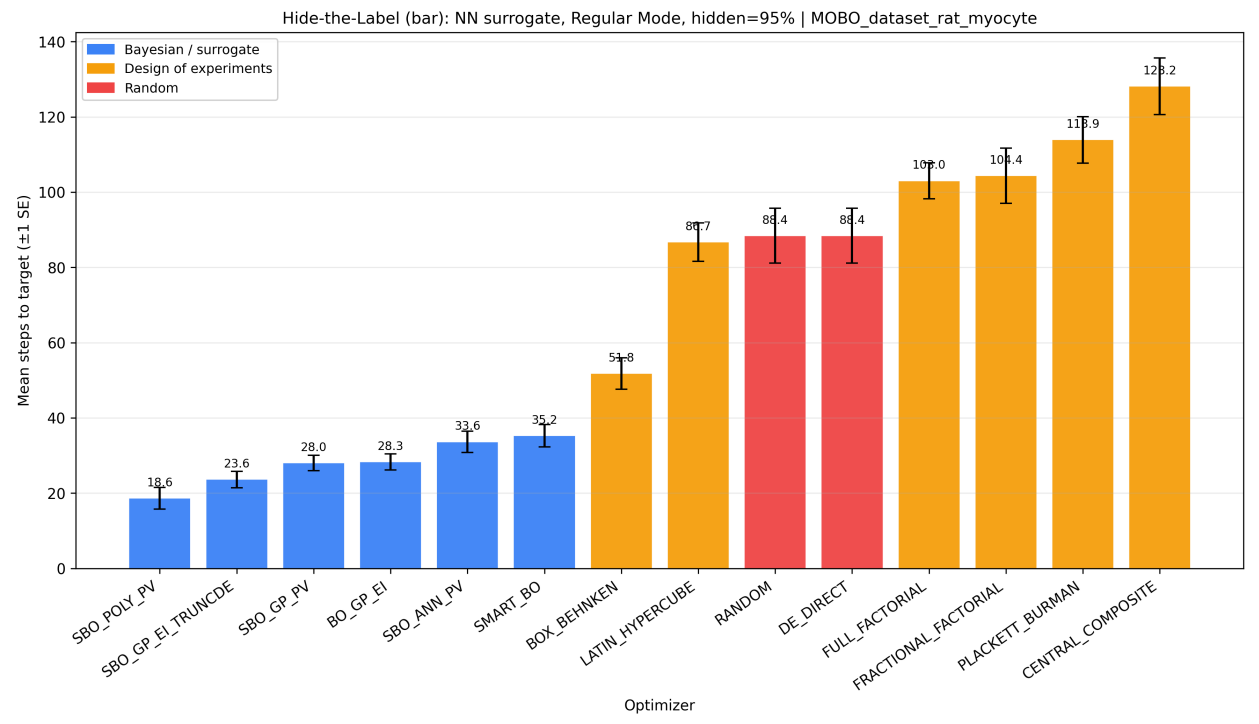

(b) MOBO rat myocyte.

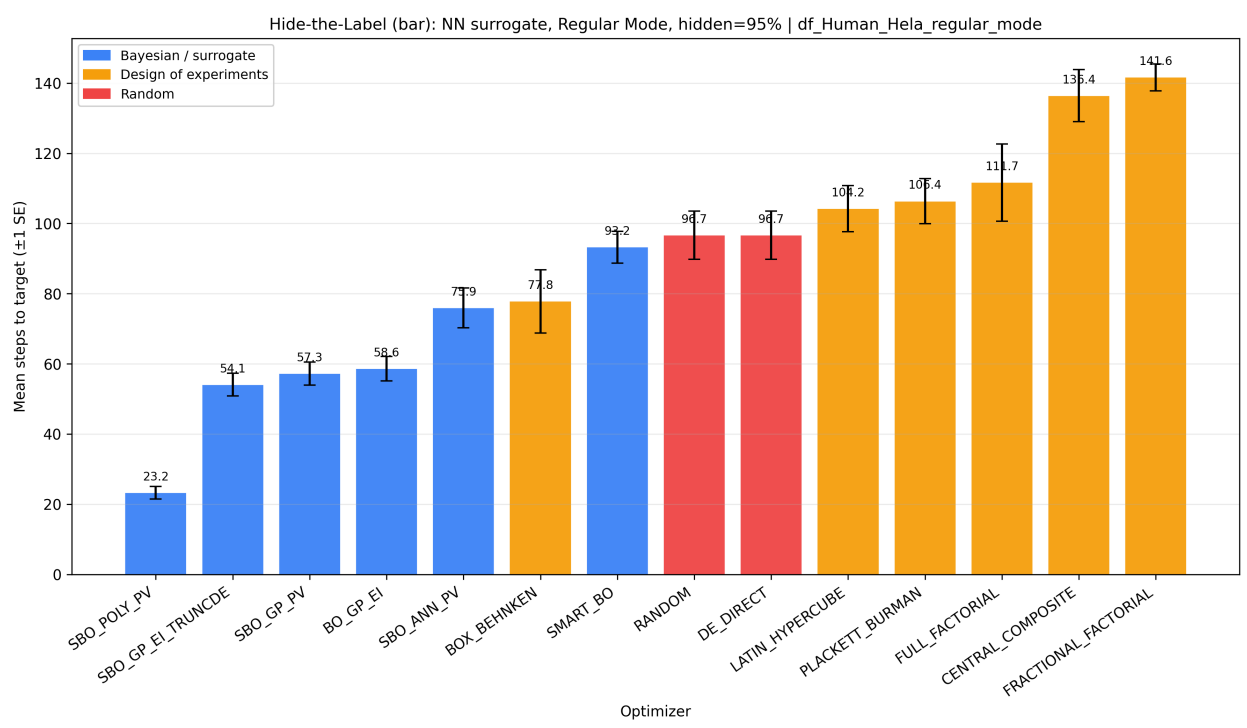

(c) HeLa (regular mode).

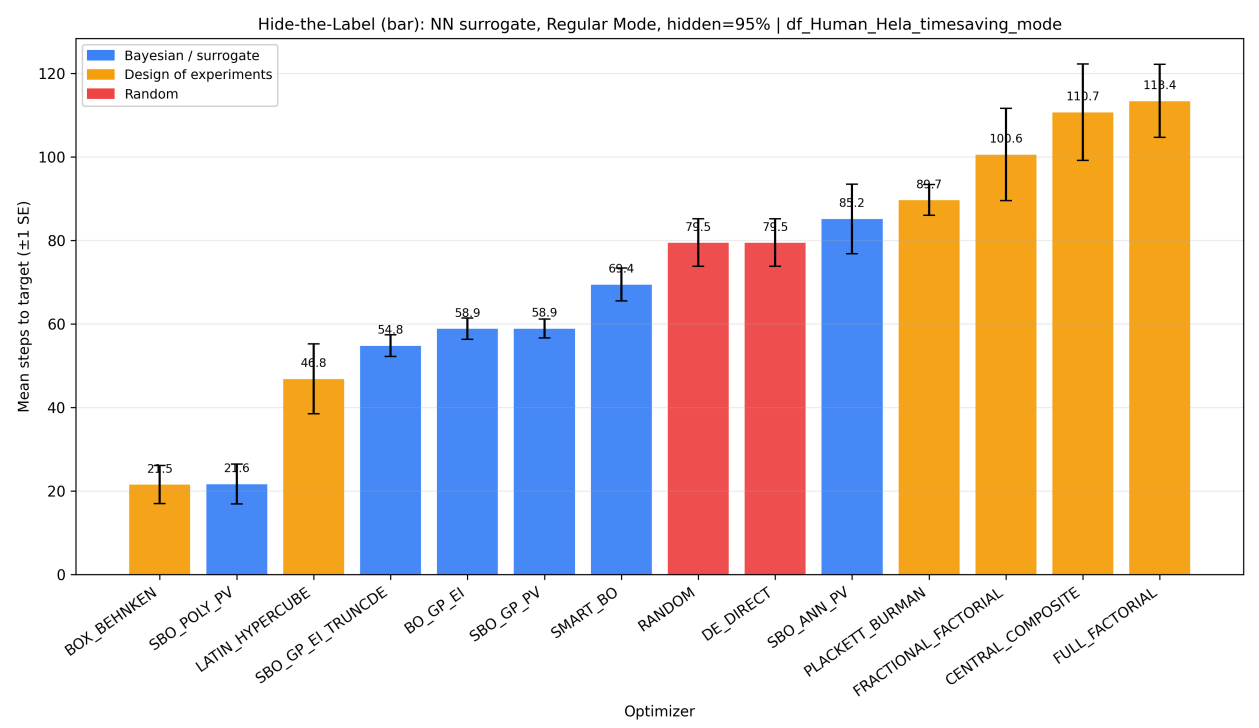

(d) HeLa (time-saving mode).

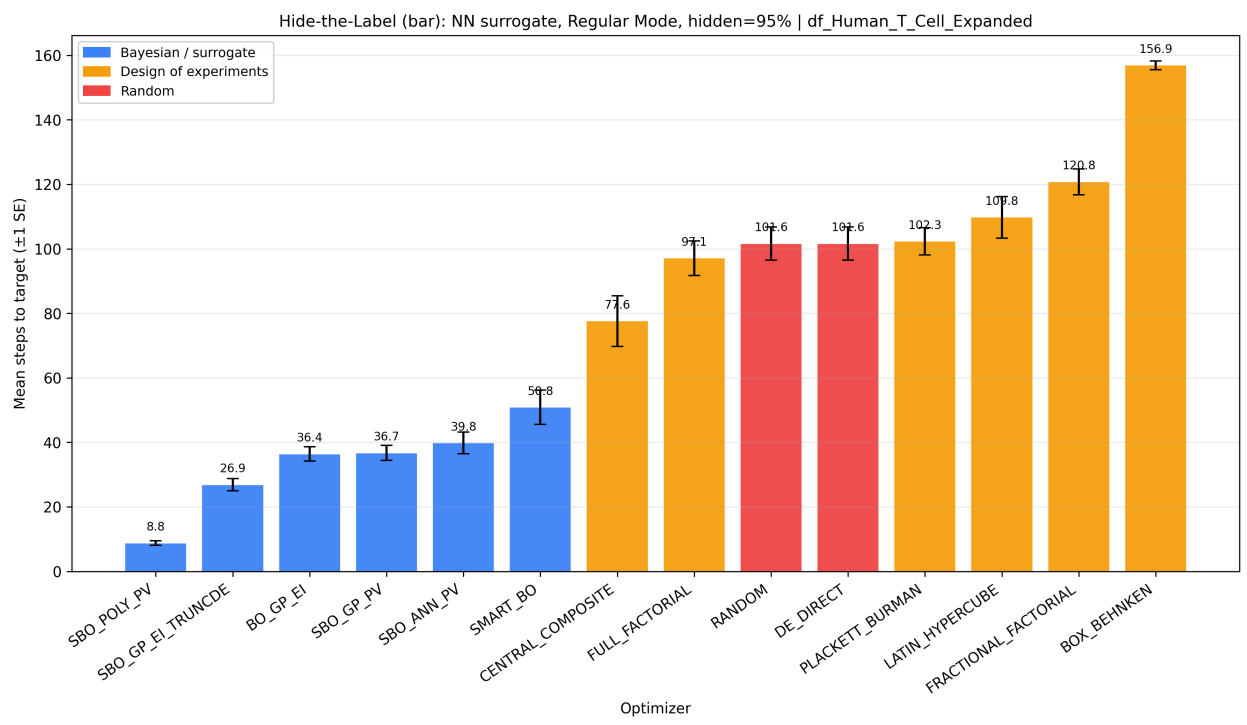

(e) T-Cell.

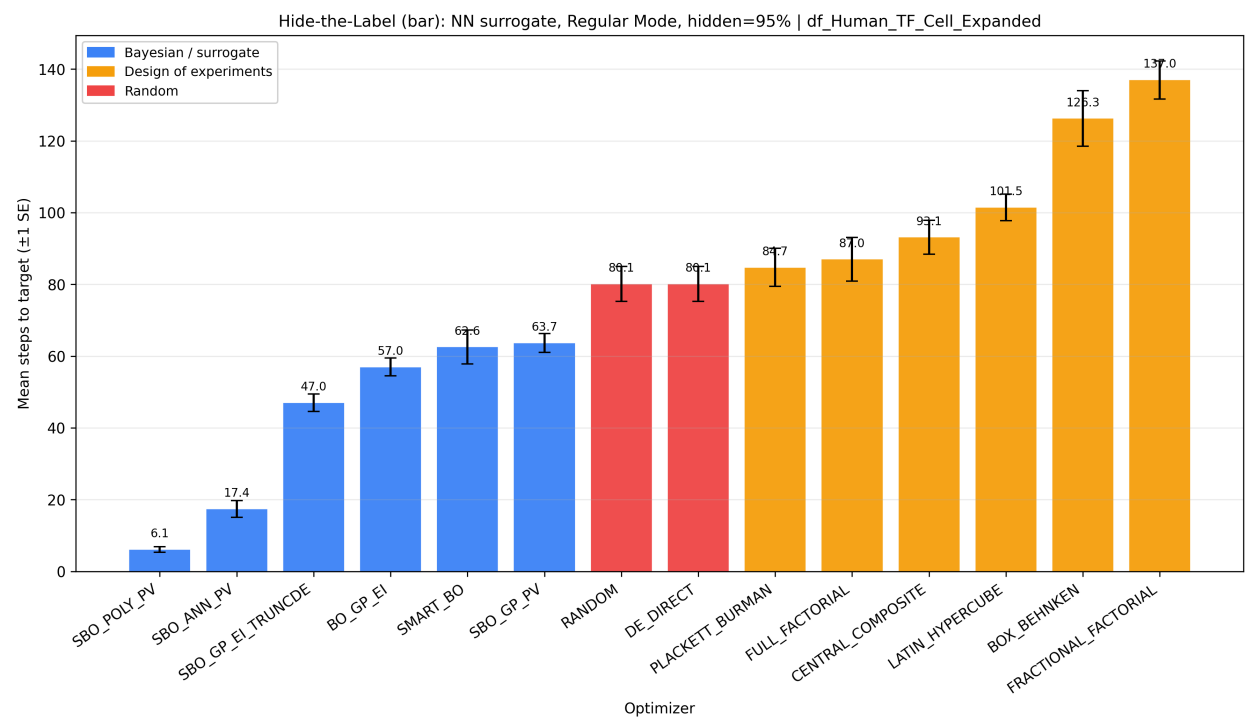

(f) TF-Cell.

Fig. 15. Hide-the-Label (HTL) competition results. **Configuration:** Regular Mode (NN surrogate, low noise), hidden fraction = 95% (5% initially revealed), Batch size = 1. **Datasets:** DBO rat myocyte (a), MOBO rat myocyte (b), HeLa regular mode (c), HeLa time-saving mode (d), T-Cell (e), TF-Cell (f). **Metric:** steps to find target label (lower is better).

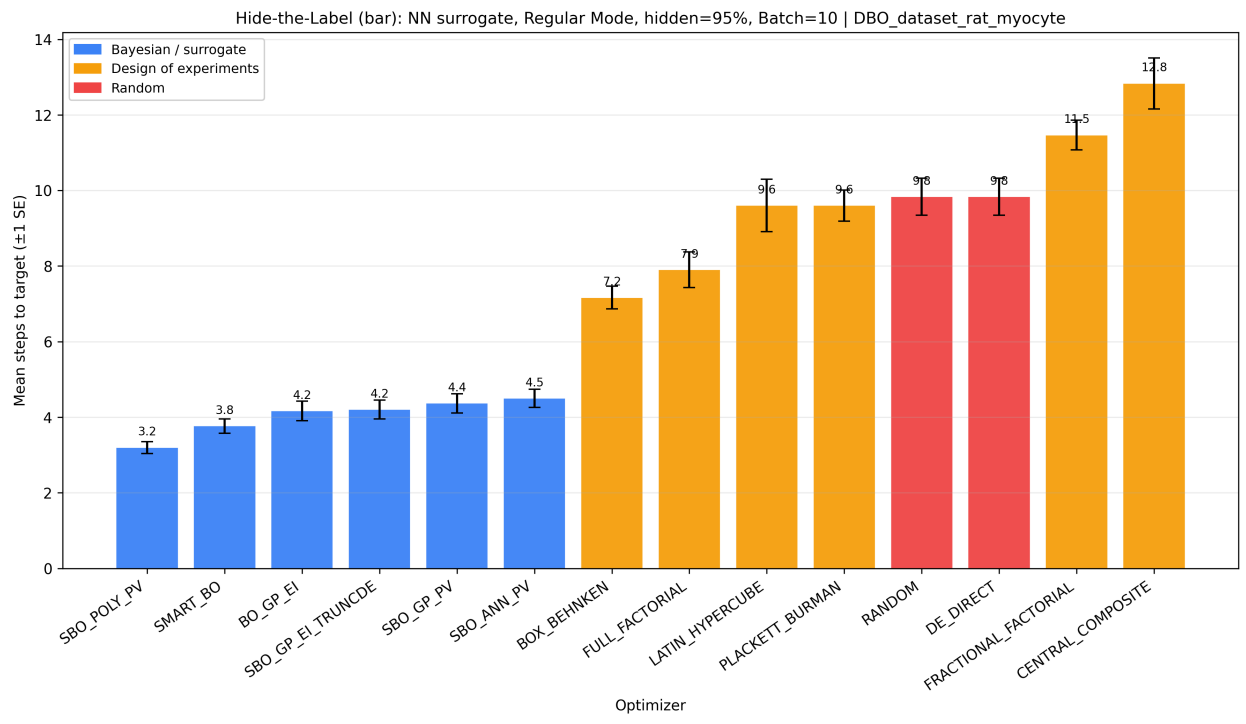

(a) DBO rat myocyte.

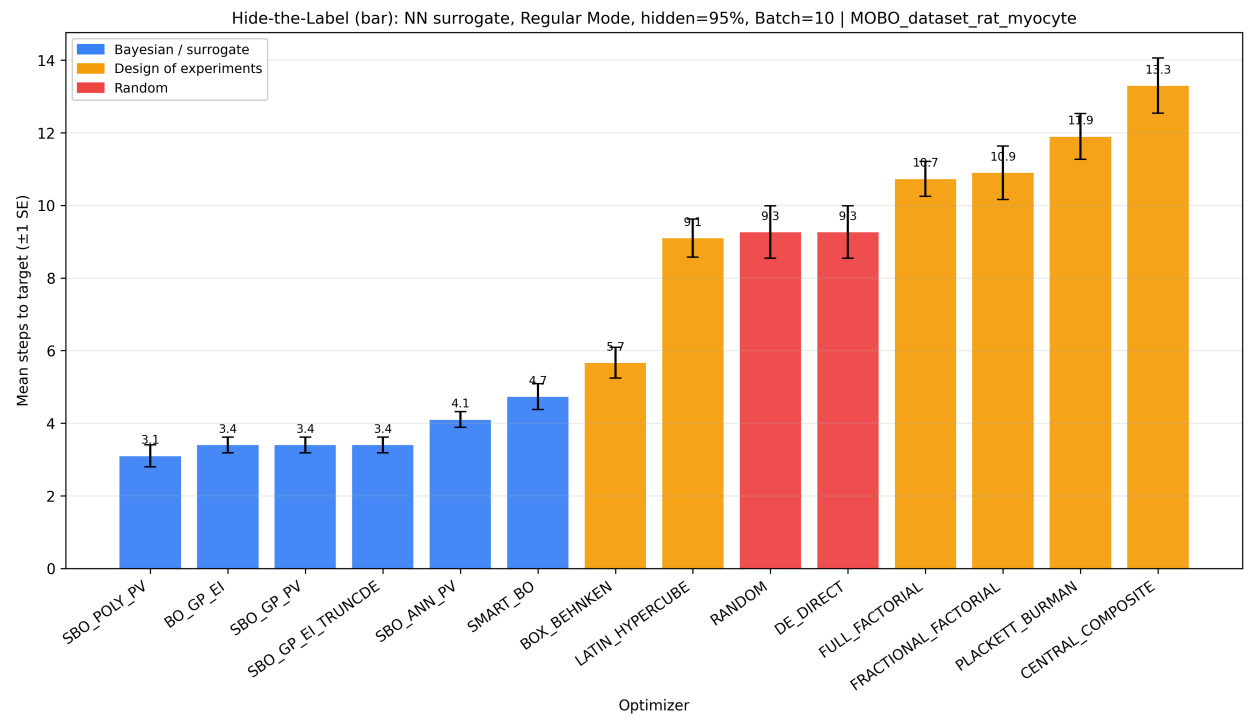

(b) MOBO rat myocyte.

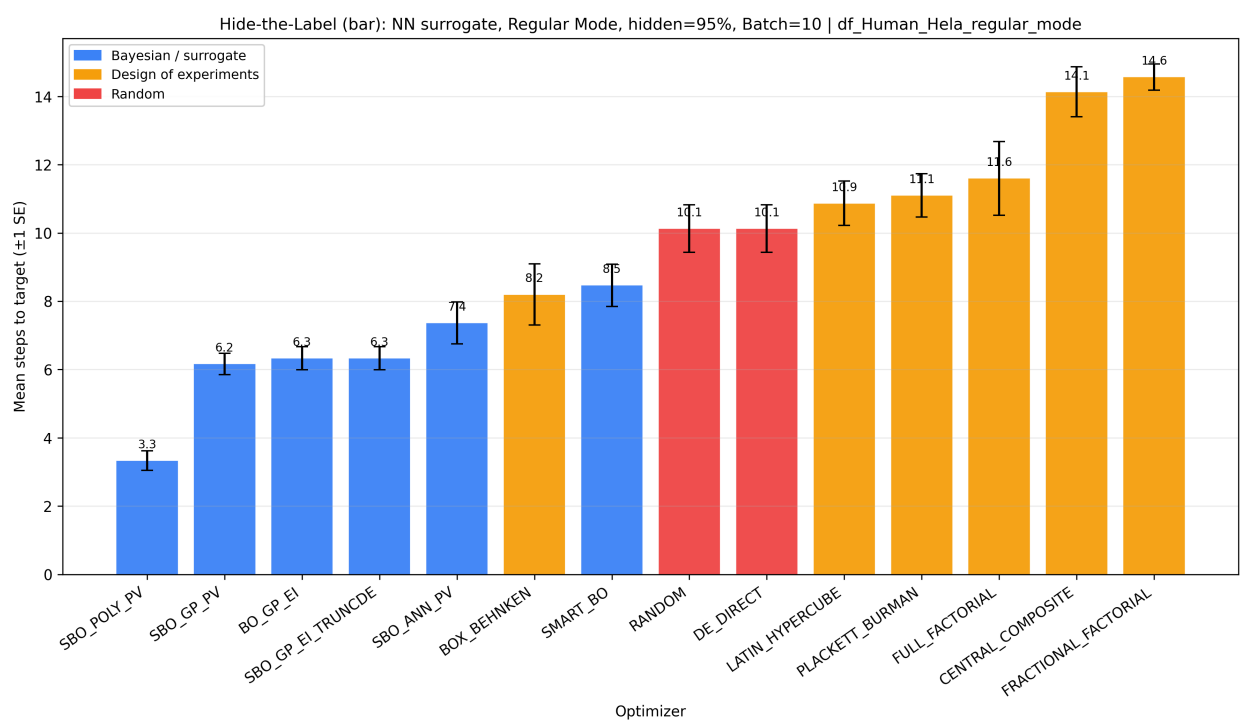

(c) HeLa (regular mode).

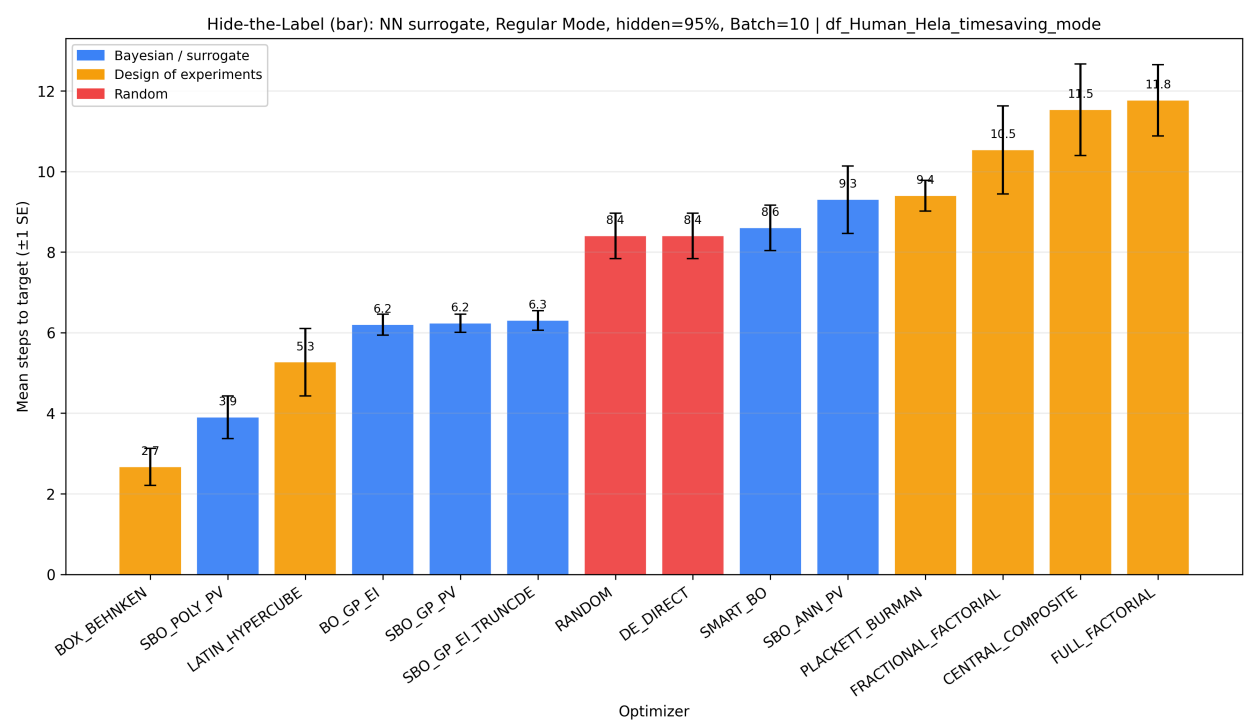

(d) HeLa (time-saving mode).

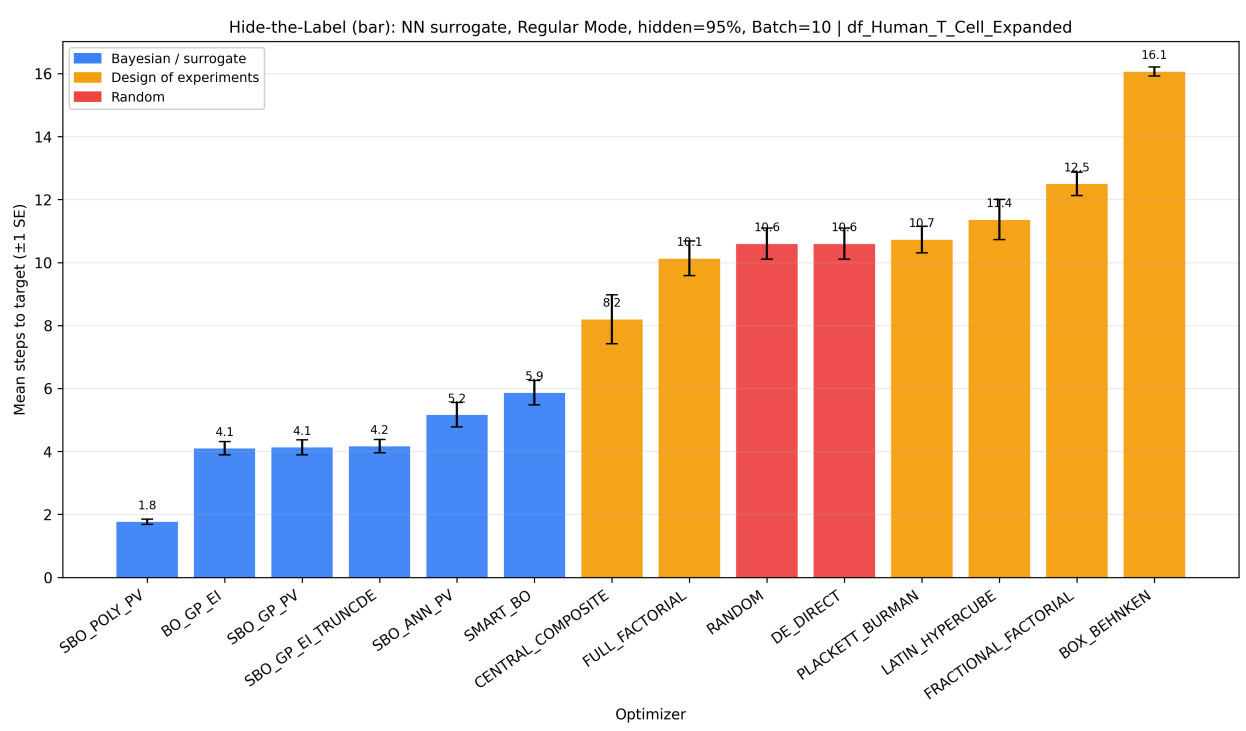

(e) T-Cell.

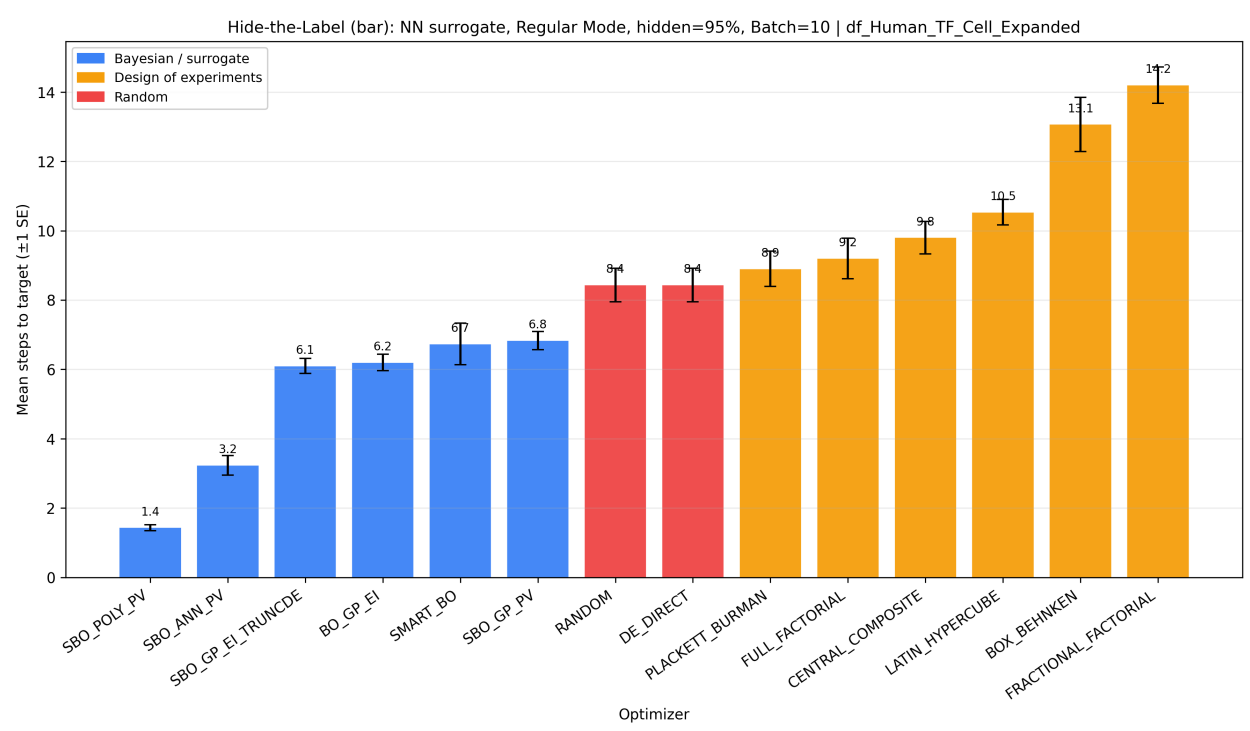

(f) TF-Cell.

Fig. 16. Hide-the-Label (HTL) competition results. **Configuration:** Regular Mode (NN surrogate, low noise), hidden fraction = 95% (5% initially revealed), Batch size = 10. **Datasets:** DBO rat myocyte (a), MOBO rat myocyte (b), HeLa regular mode (c), HeLa time-saving mode (d), T-Cell (e), TF-Cell (f). **Metric:** steps to find target label (lower is better).

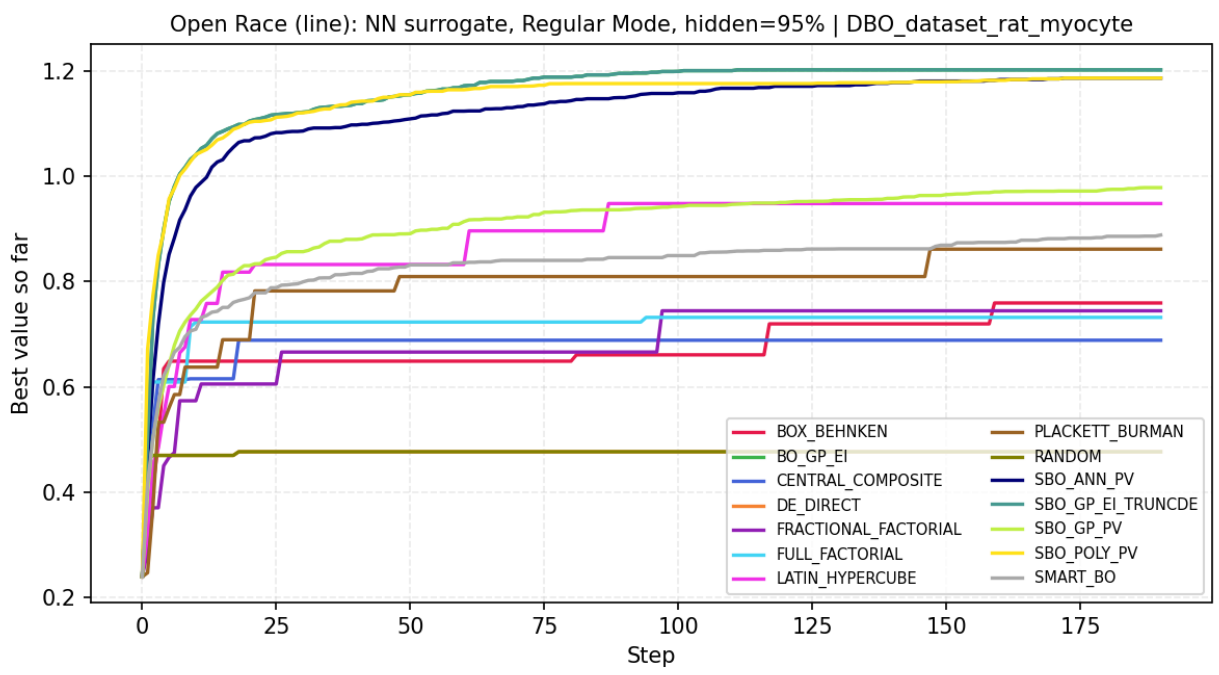

(a) DBO rat myocyte.

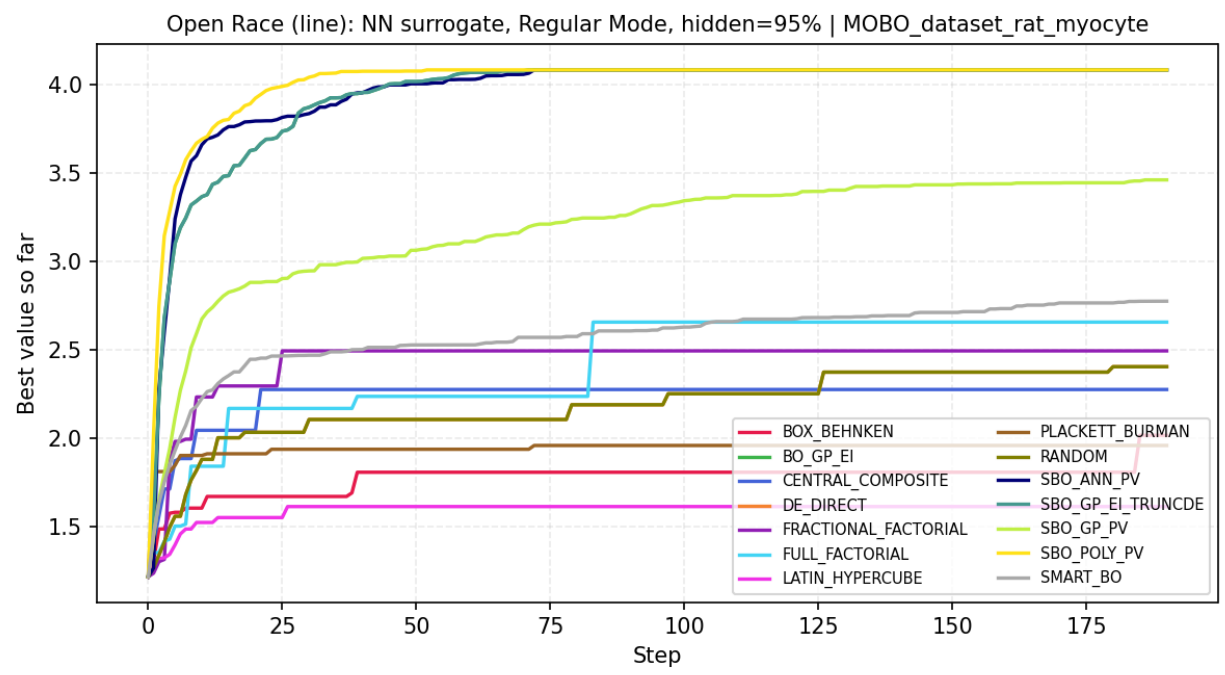

(b) MOBO rat myocyte.

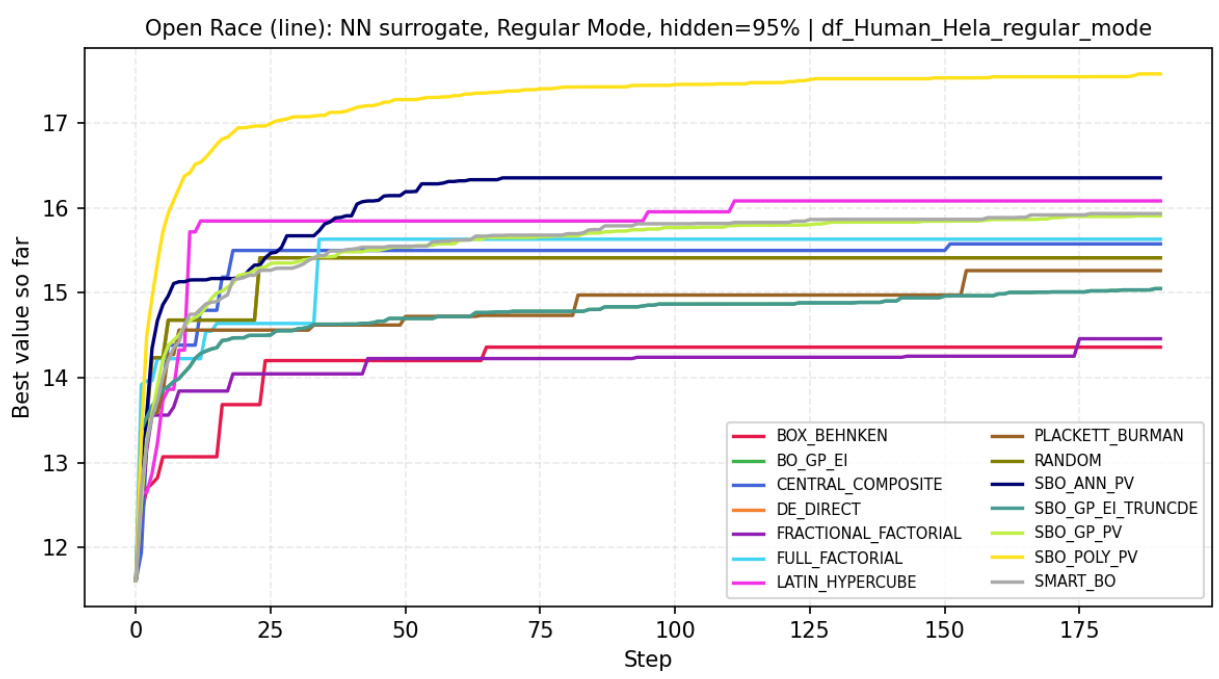

(c) HeLa (regular mode).

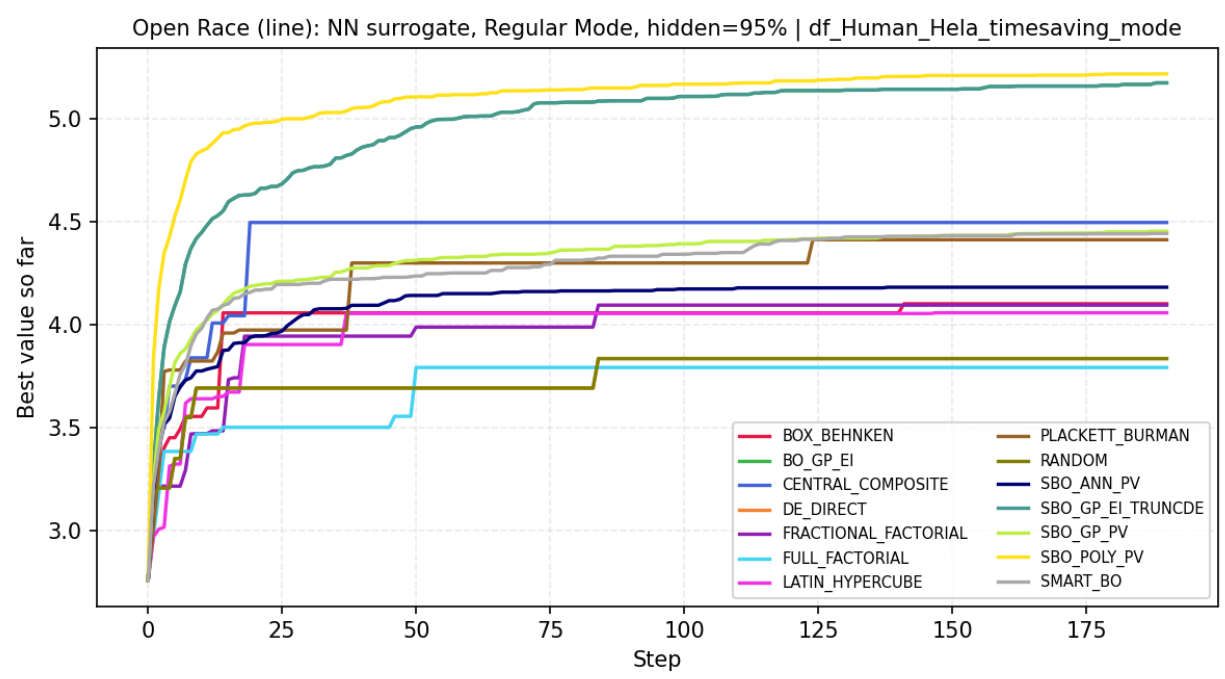

(d) HeLa (time-saving mode).

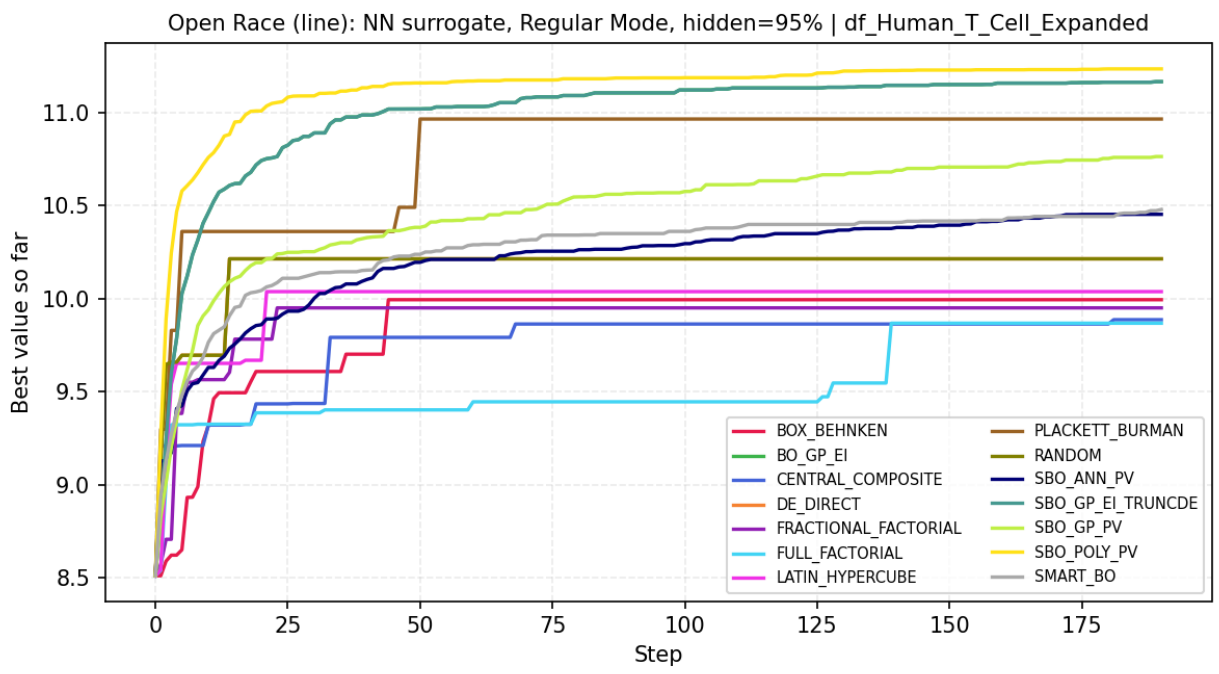

(e) T-Cell.

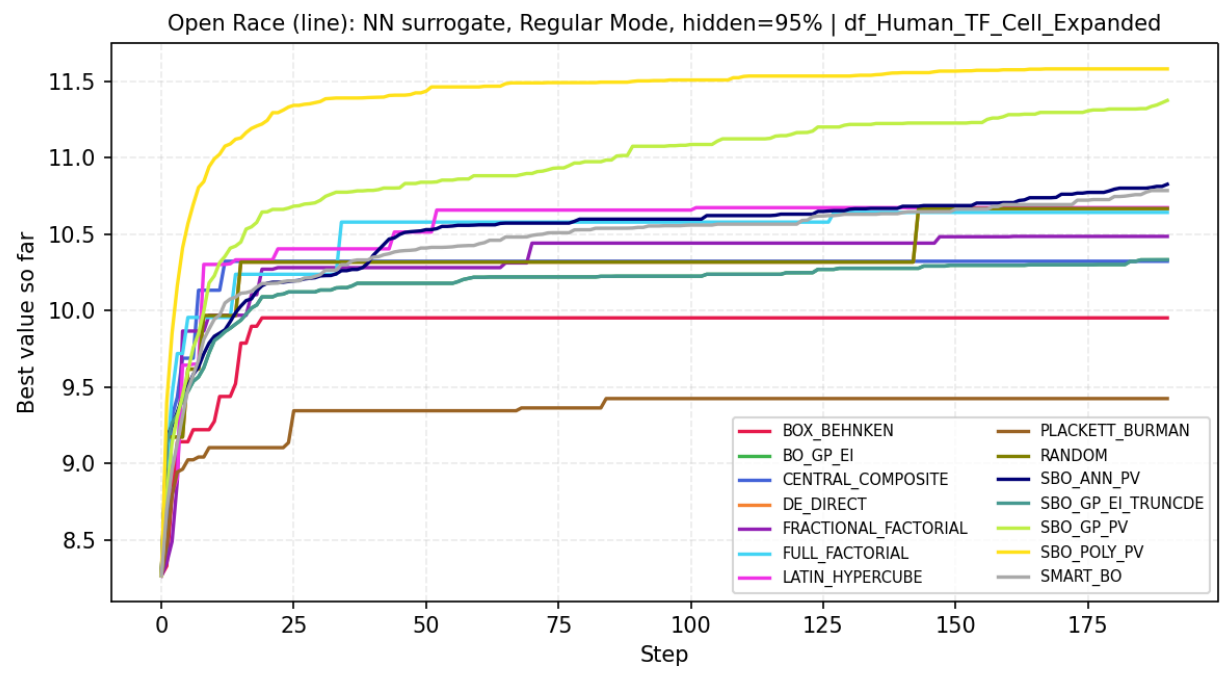

(f) TF-Cell.

Fig. 17. Open Race (OR) competition results. **Configuration:** Regular Mode (NN surrogate, low noise), hidden fraction = 95% (5% initially revealed). **Datasets:** DBO rat myocyte (a), MOBO rat myocyte (b), HeLa regular mode (c), HeLa time-saving mode (d), T-Cell (e), TF-Cell (f). **Metric:** best value found so far over cumulative evaluation steps (higher is better).

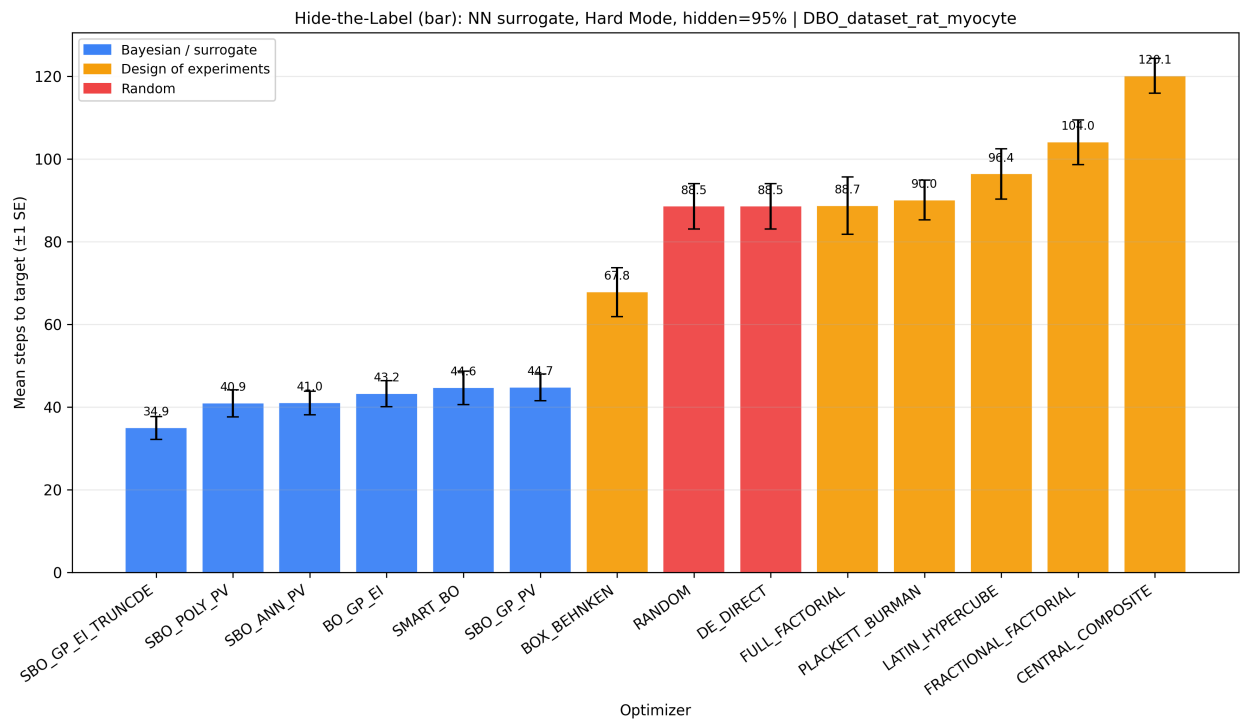

(a) DBO rat myocyte.

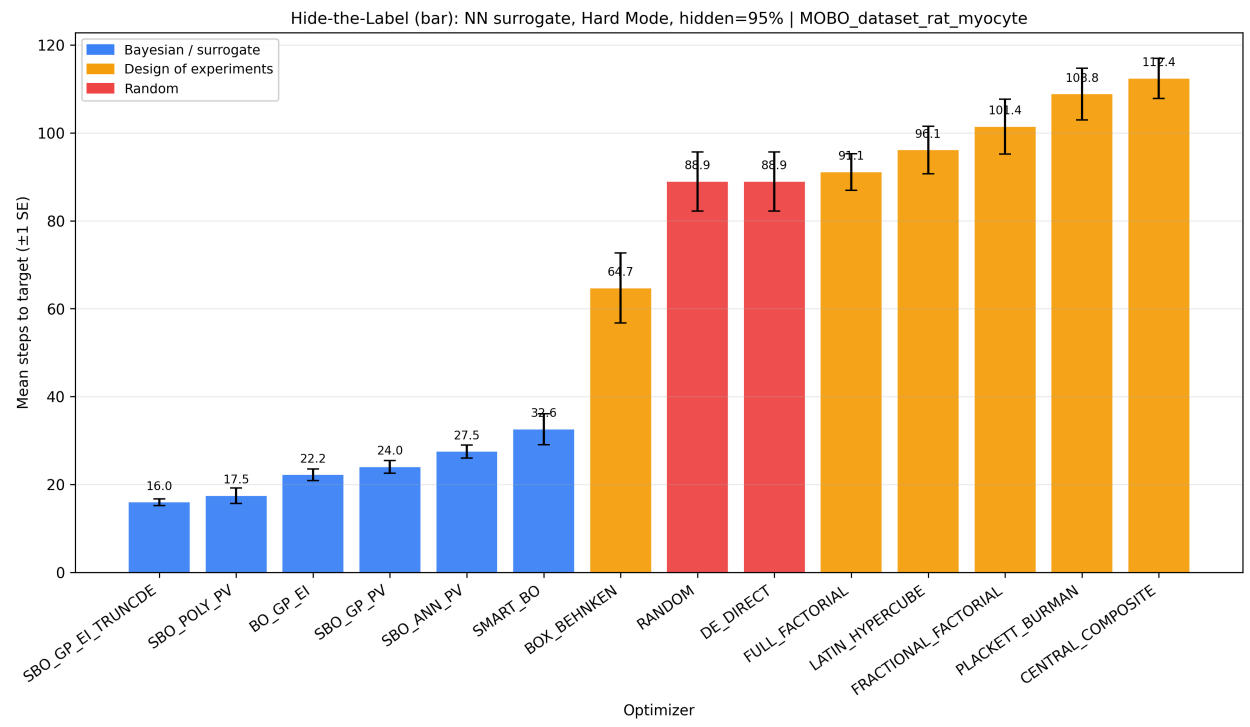

(b) MOBO rat myocyte.

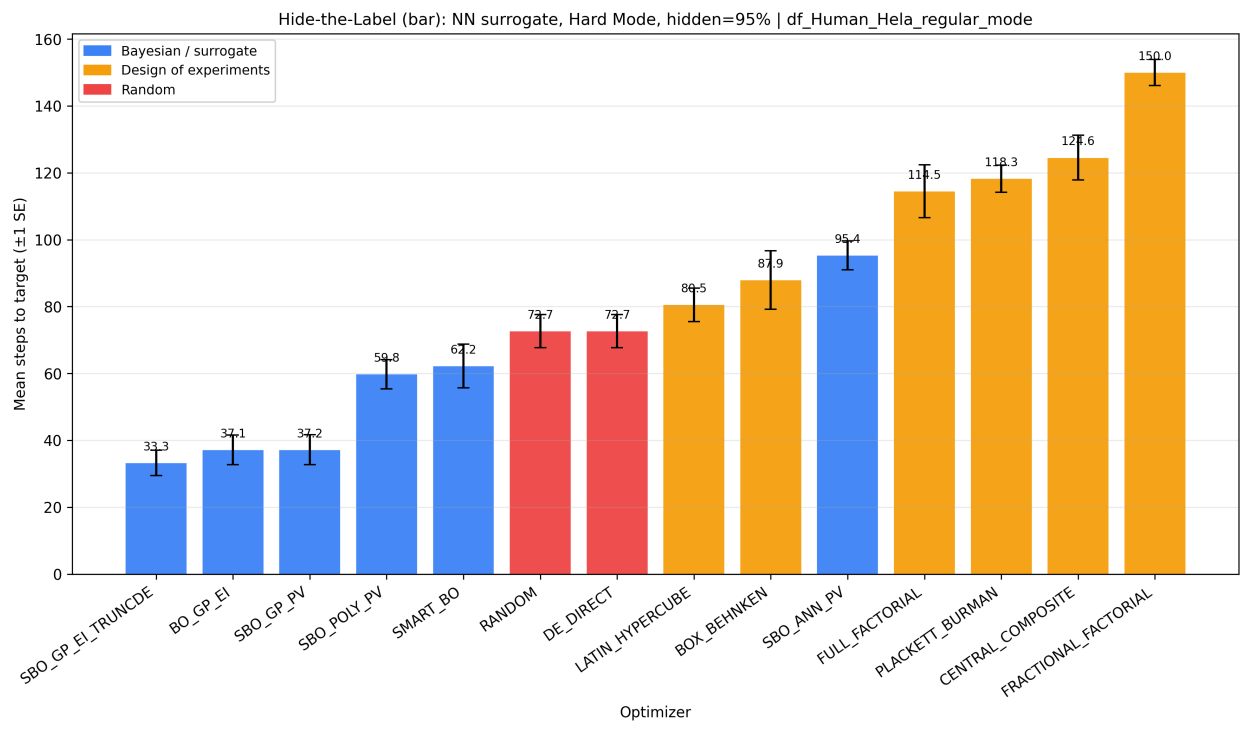

(c) HeLa (regular mode).

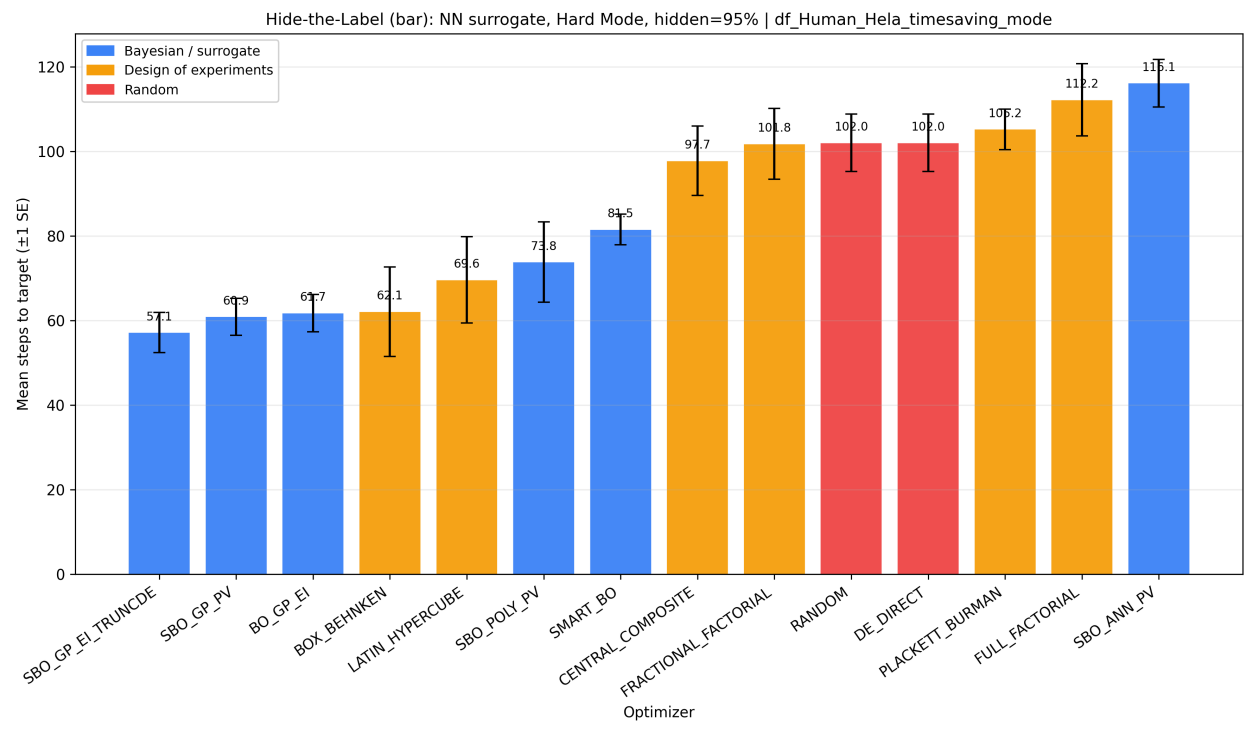

(d) HeLa (time-saving mode).

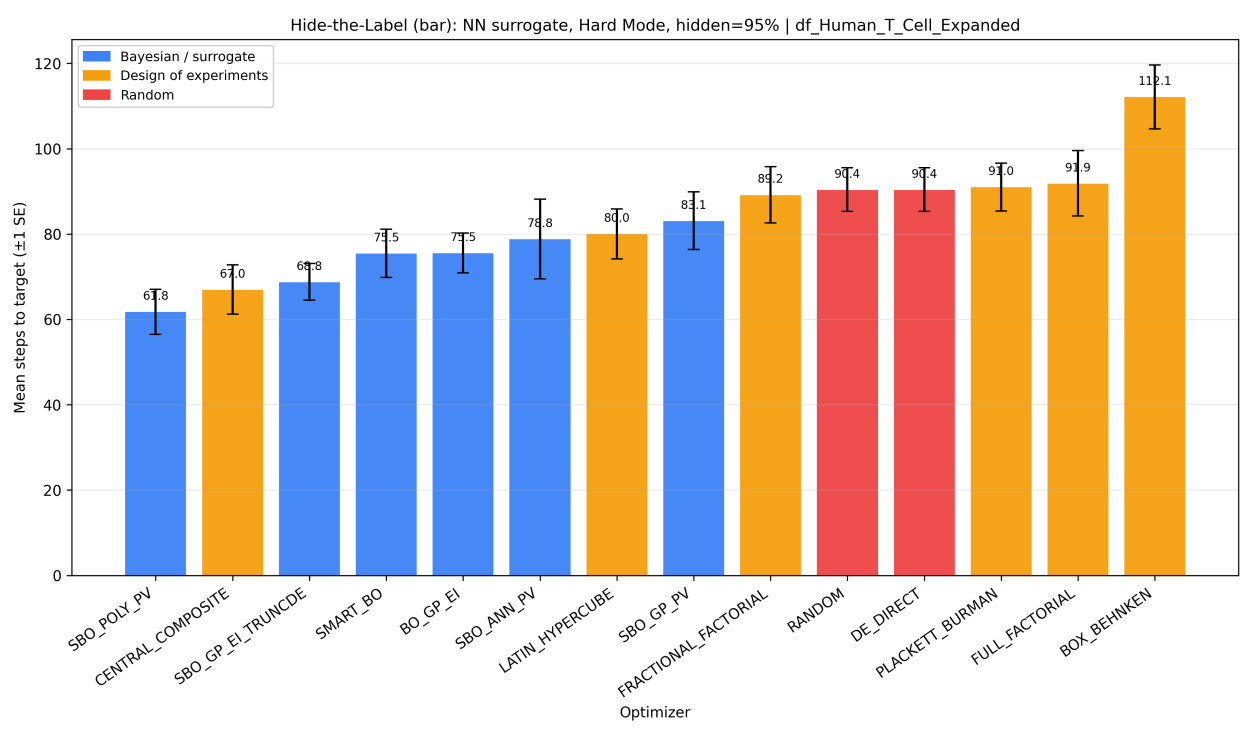

(e) T-Cell.

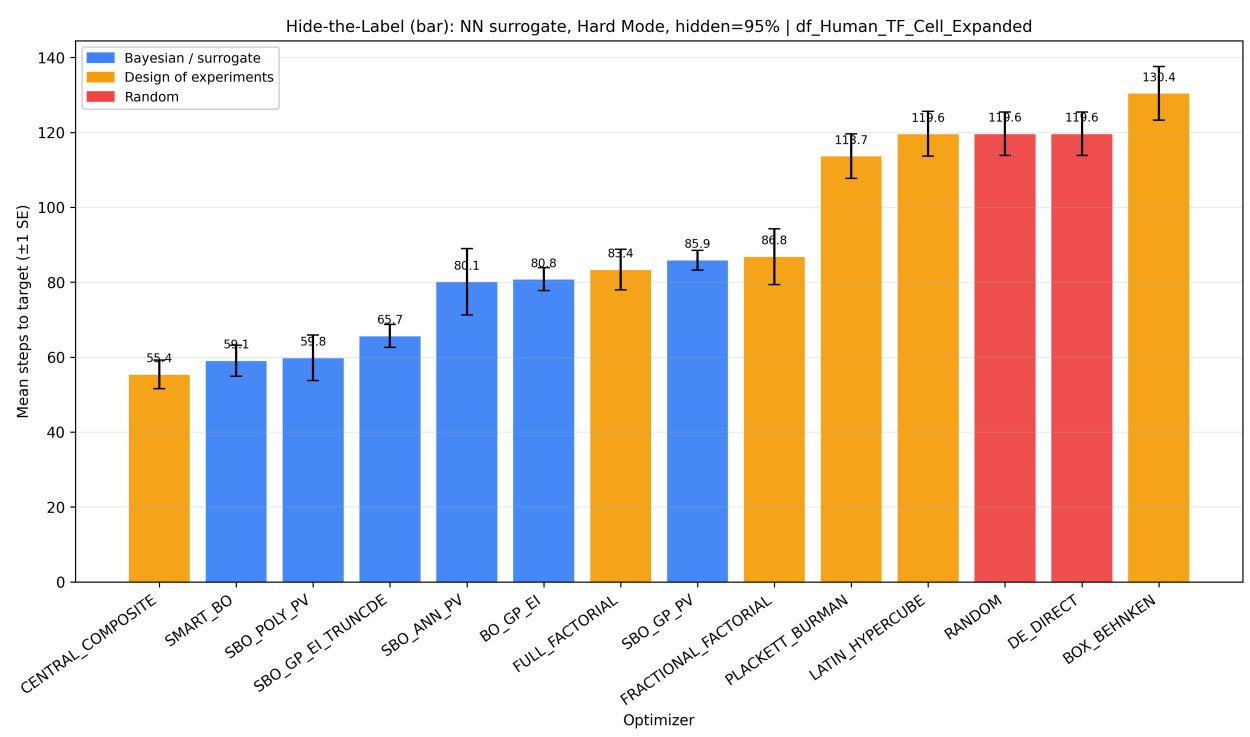

(f) TF-Cell.

Fig. 18. Hide-the-Label (HTL) competition results. **Configuration:** Hard Mode (NN surrogate, heteroskedastic noise & bimodal perturbations), hidden fraction = 95% (5% initially revealed), Batch size = 1. **Datasets:** DBO rat myocyte (a), MOBO rat myocyte (b), HeLa regular mode (c), HeLa time-saving mode (d), T-Cell (e), TF-Cell (f). **Metric:** steps to find target label (lower is better).

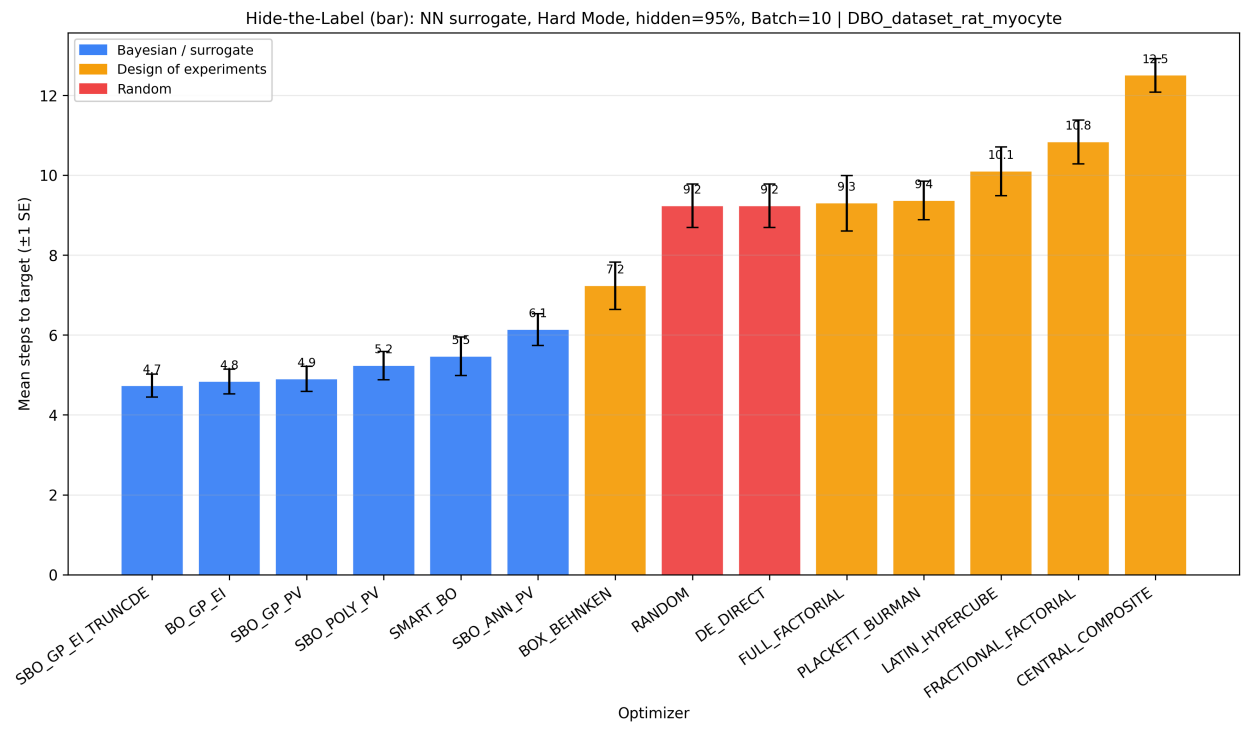

(a) DBO rat myocyte.

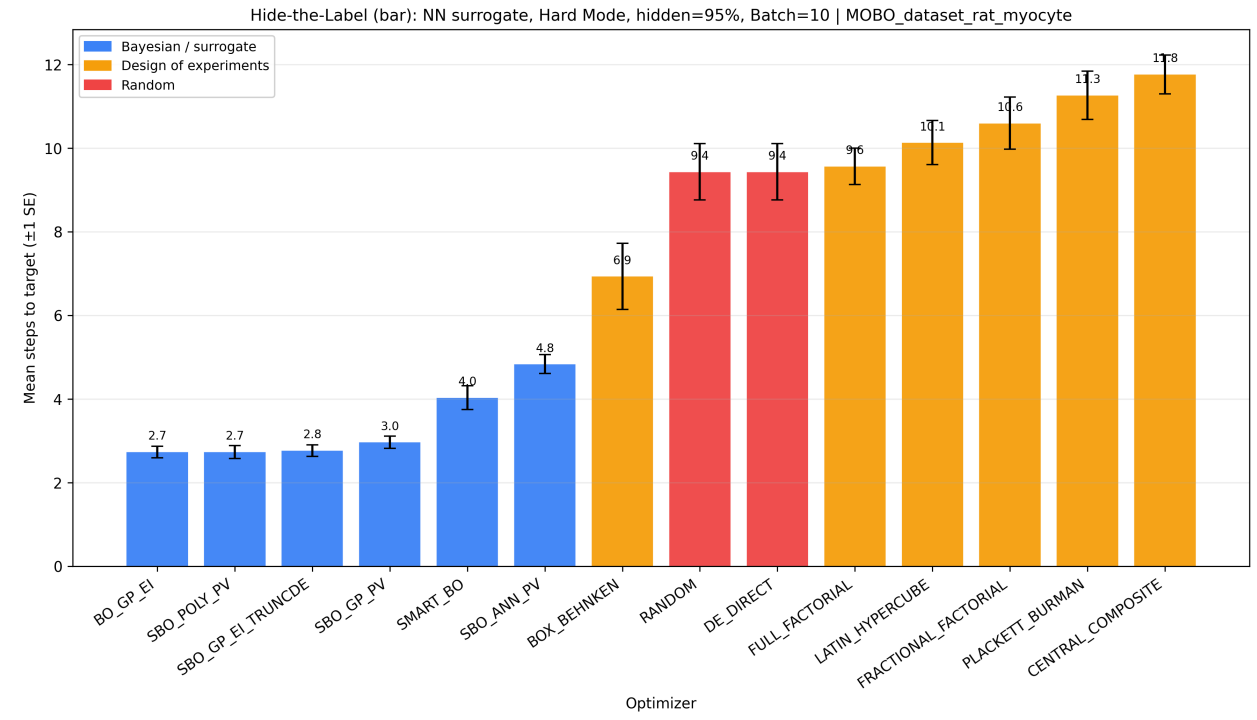

(b) MOBO rat myocyte.

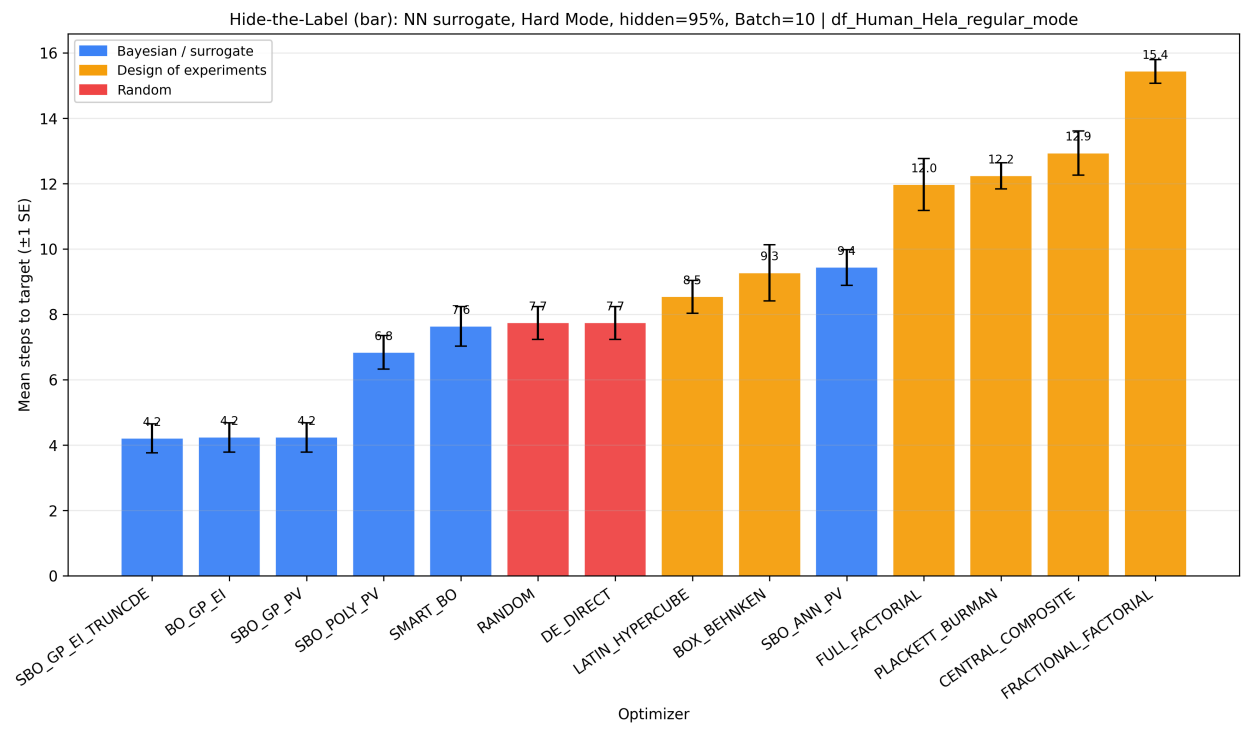

(c) HeLa (regular mode).

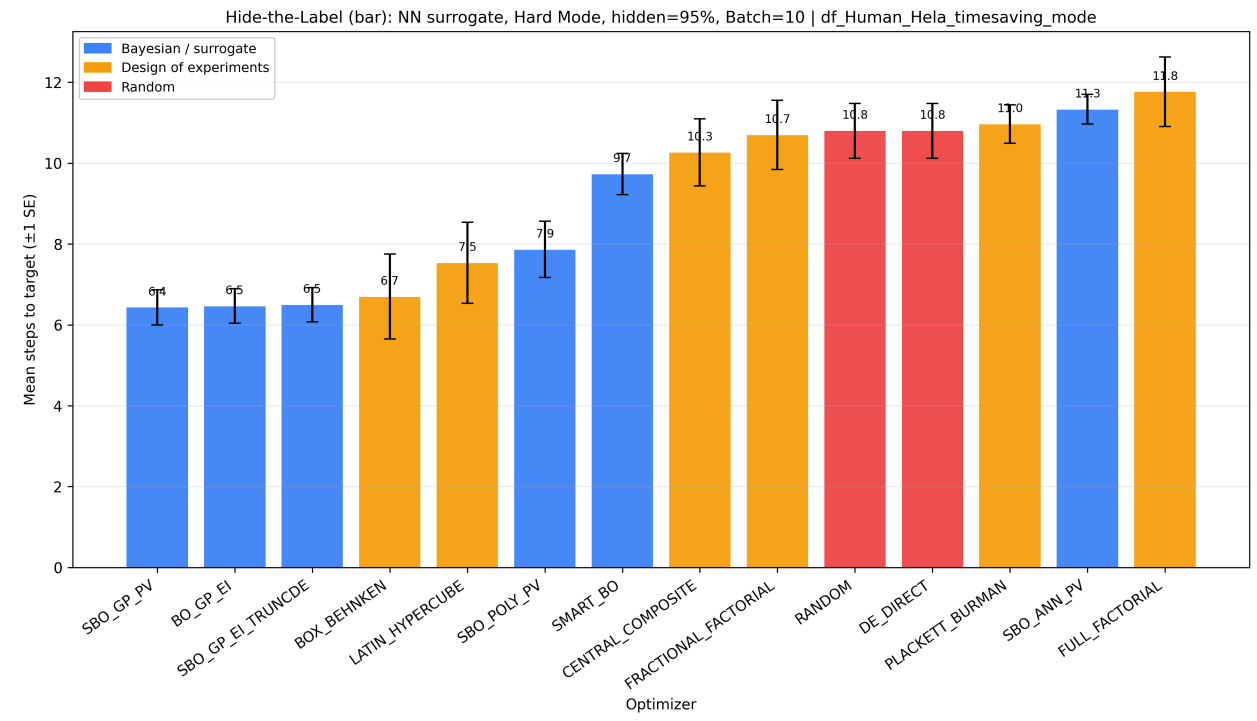

(d) HeLa (time-saving mode).

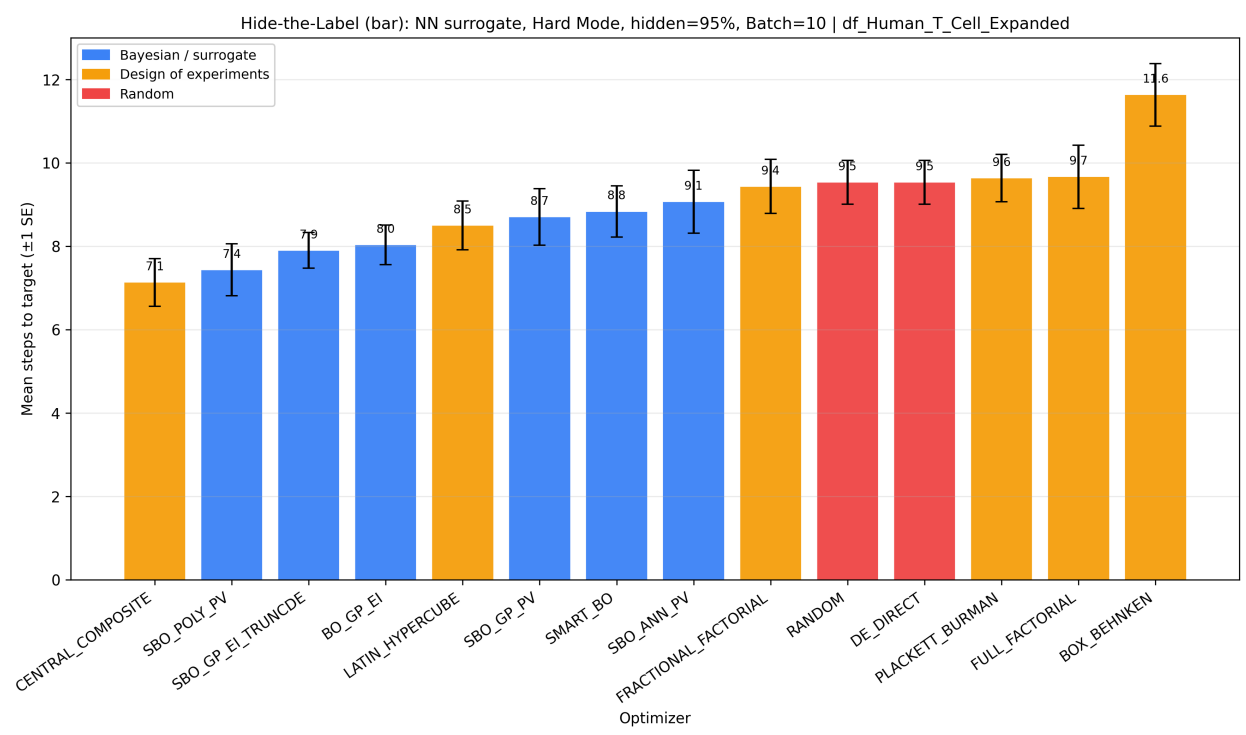

(e) T-Cell.

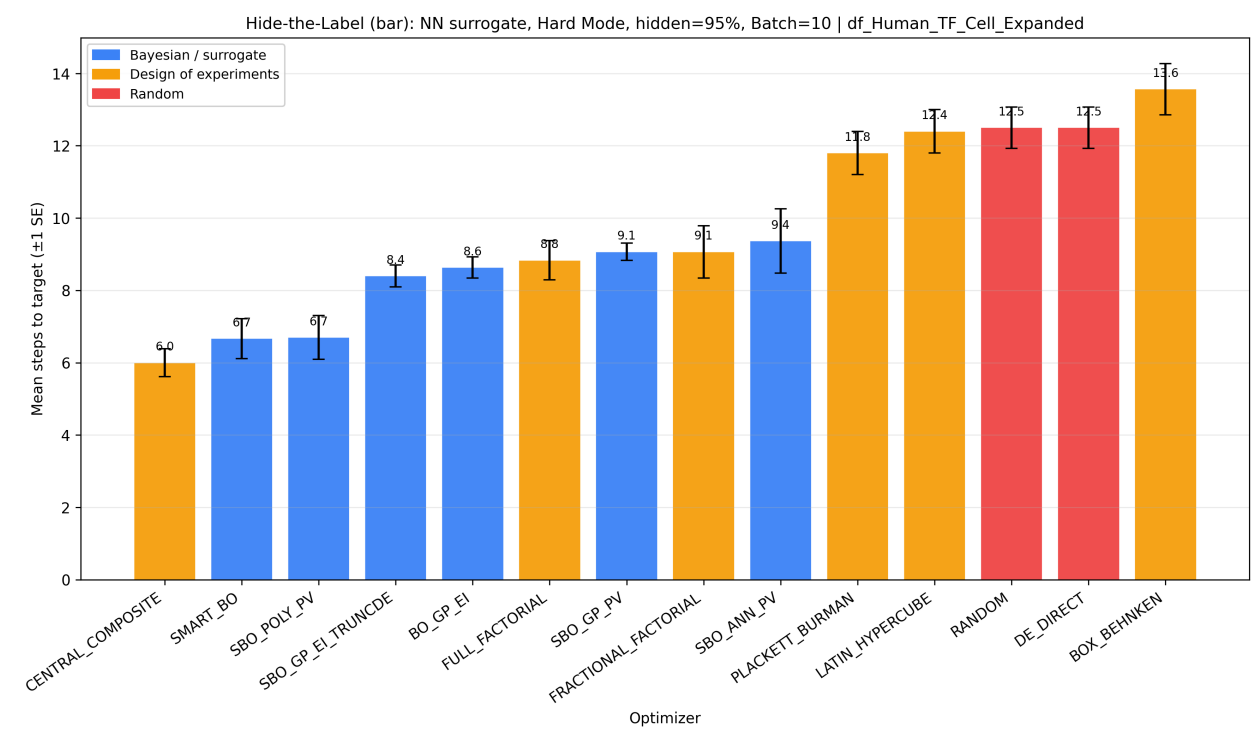

(f) TF-Cell.

Fig. 19. Hide-the-Label (HTL) competition results. **Configuration:** Hard Mode (NN surrogate, heteroskedastic noise & bimodal perturbations), hidden fraction = 95% (5% initially revealed), Batch size = 10. **Datasets:** DBO rat myocyte (a), MOBO rat myocyte (b), HeLa regular mode (c), HeLa time-saving mode (d), T-Cell (e), TF-Cell (f). **Metric:** steps to find target label (lower is better).

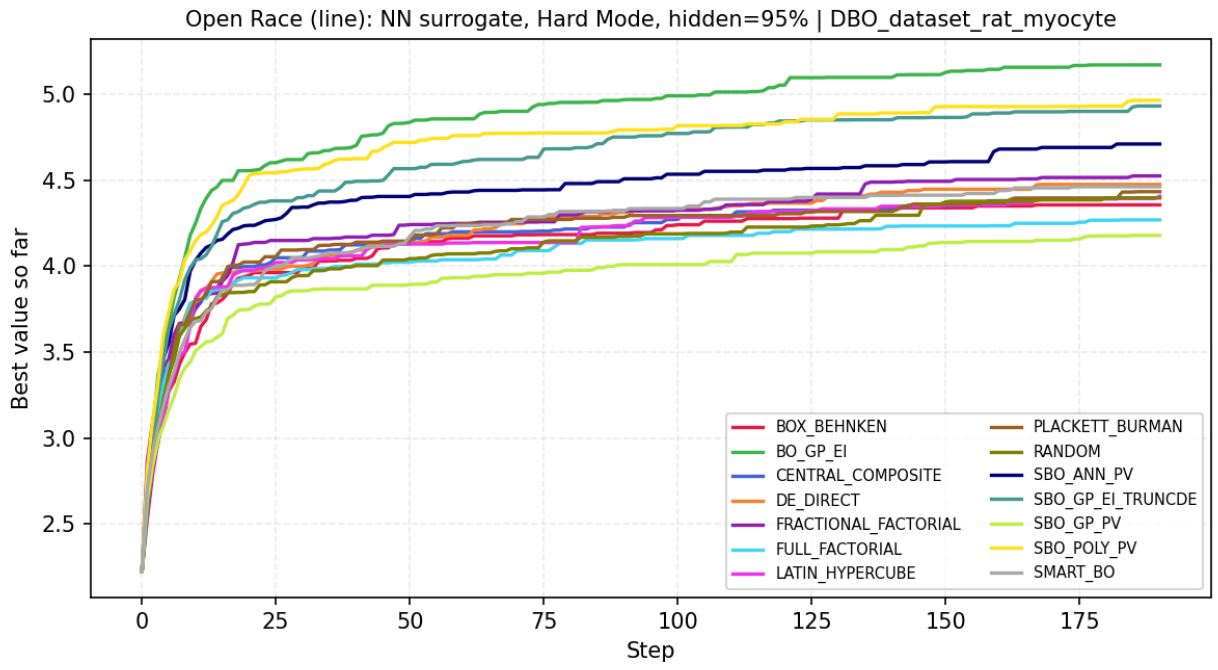

(a) DBO rat myocyte.

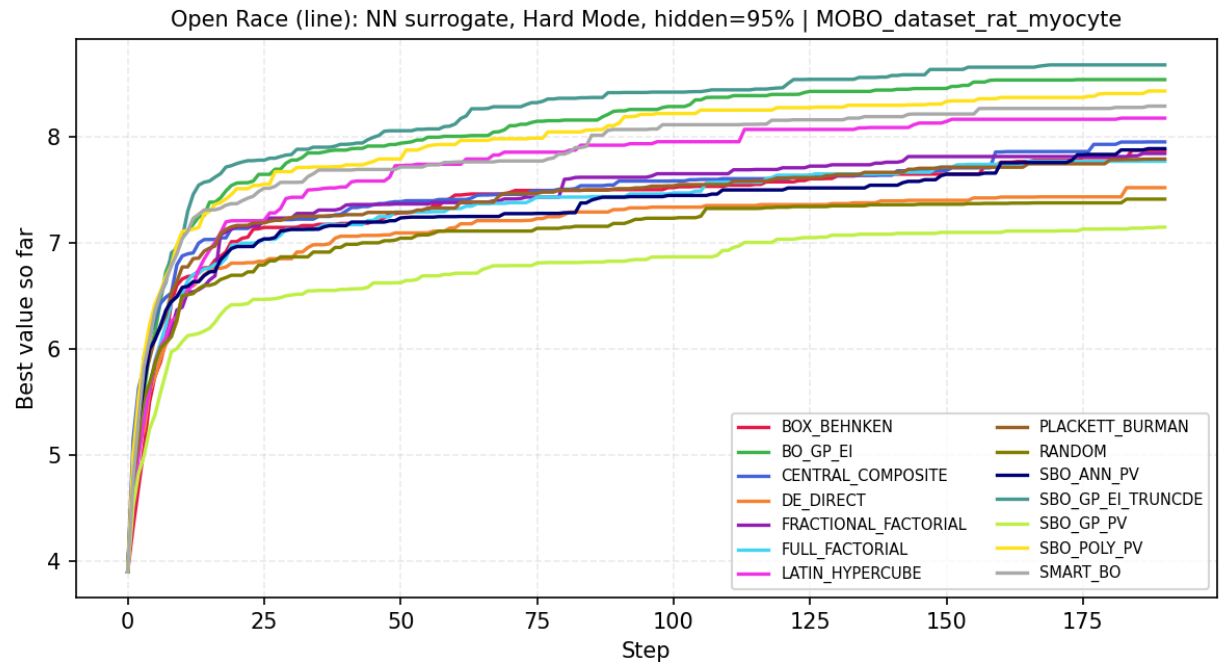

(b) MOBO rat myocyte.

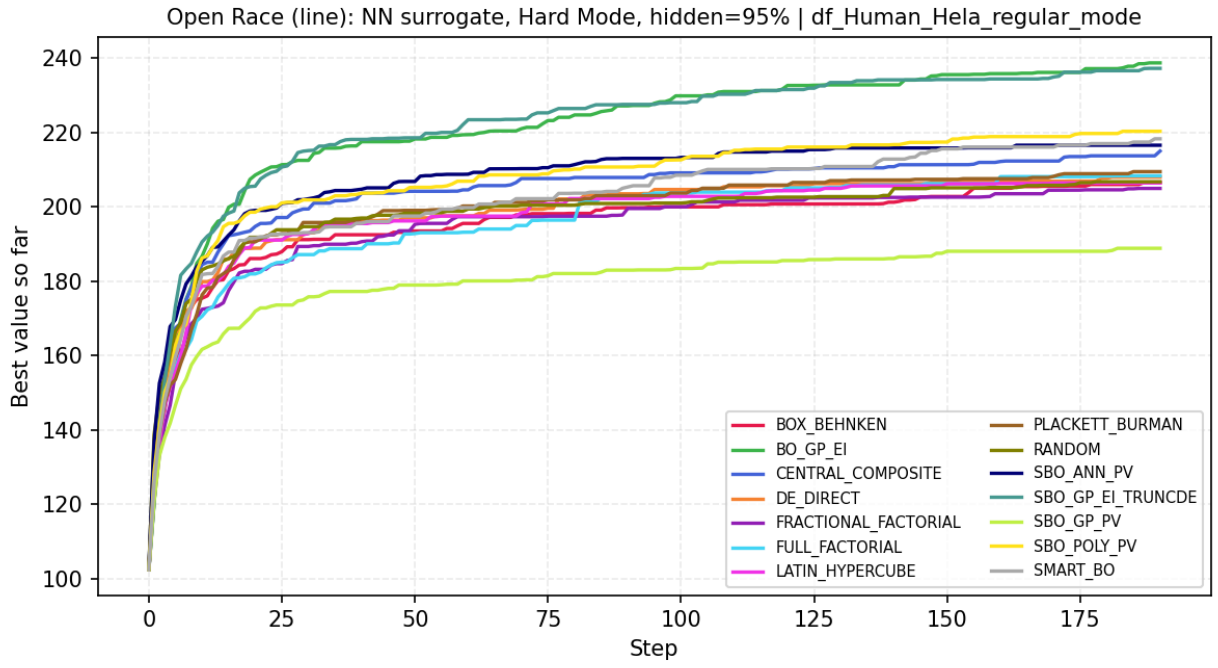

(c) HeLa (regular mode).

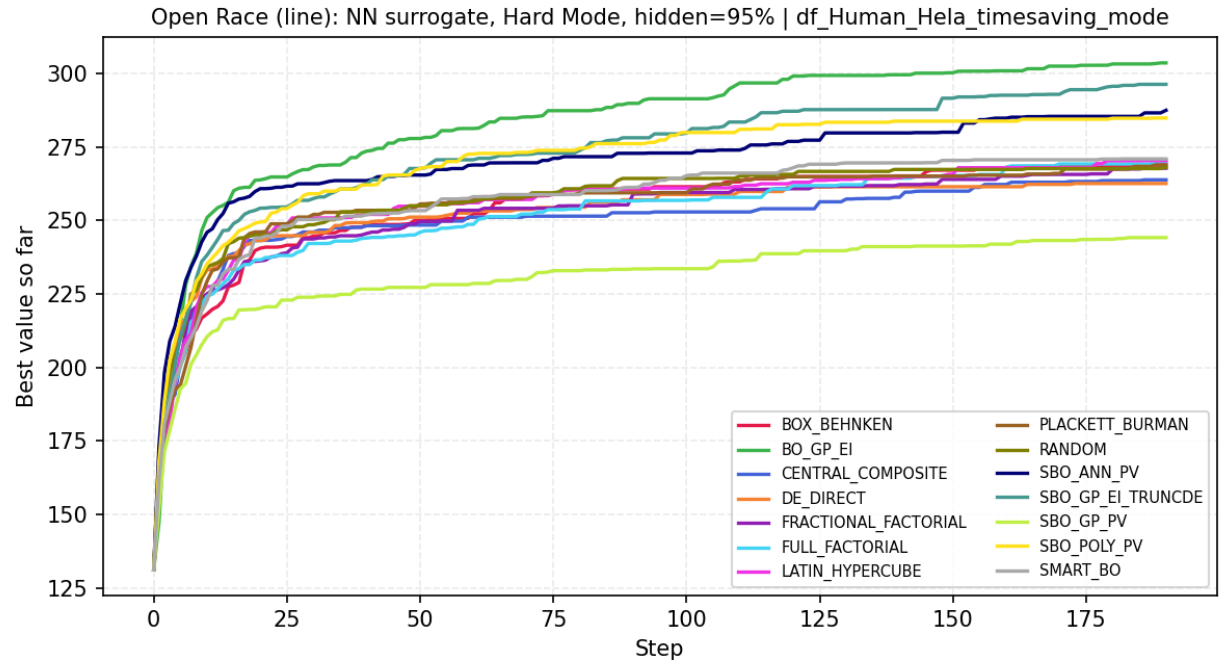

(d) HeLa (time-saving mode).

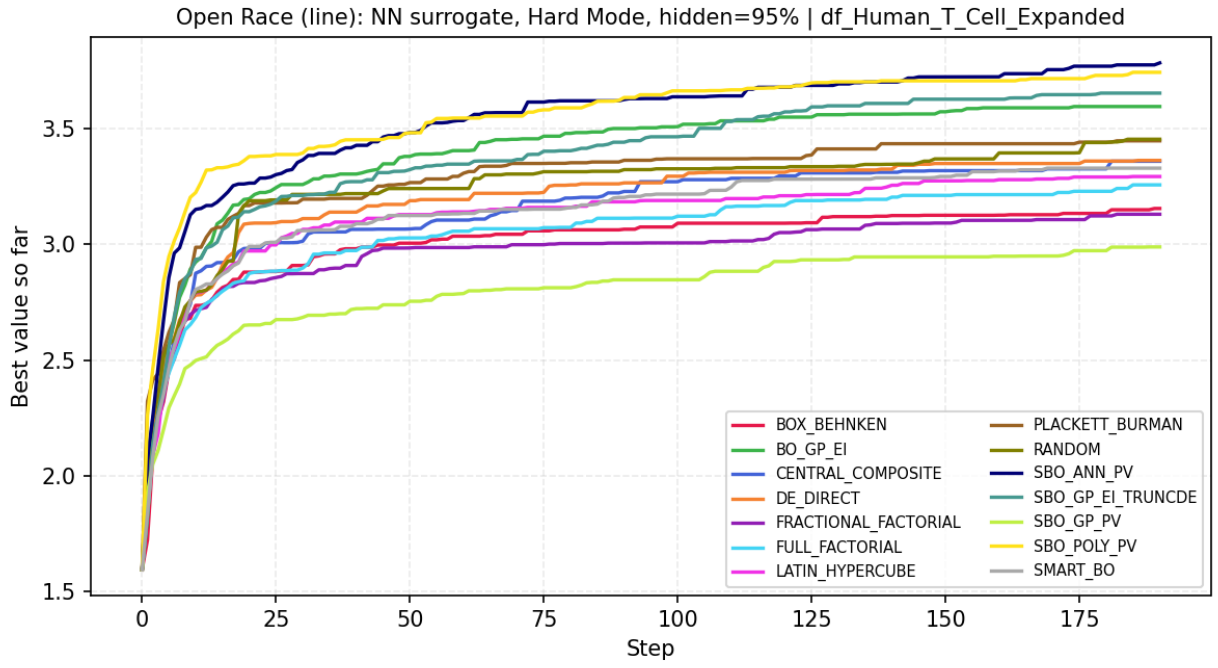

(e) T-Cell.

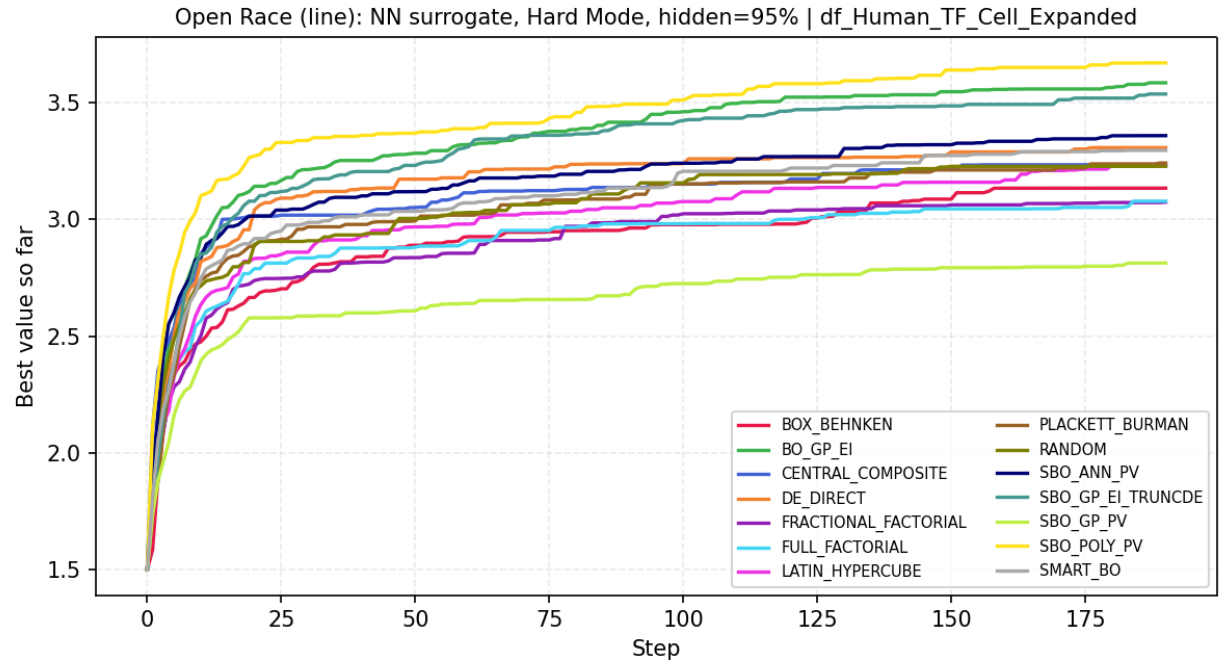

(f) TF-Cell.

Fig. 20. Open Race (OR) competition results. **Configuration:** Hard Mode (NN surrogate, heteroskedastic noise & bimodal perturbations), hidden fraction = 95% (5% initially revealed). **Datasets:** DBO rat myocyte (a), MOBO rat myocyte (b), HeLa regular mode (c), HeLa time-saving mode (d), T-Cell (e), TF-Cell (f). **Metric:** best value found so far over cumulative evaluation steps (higher is better).

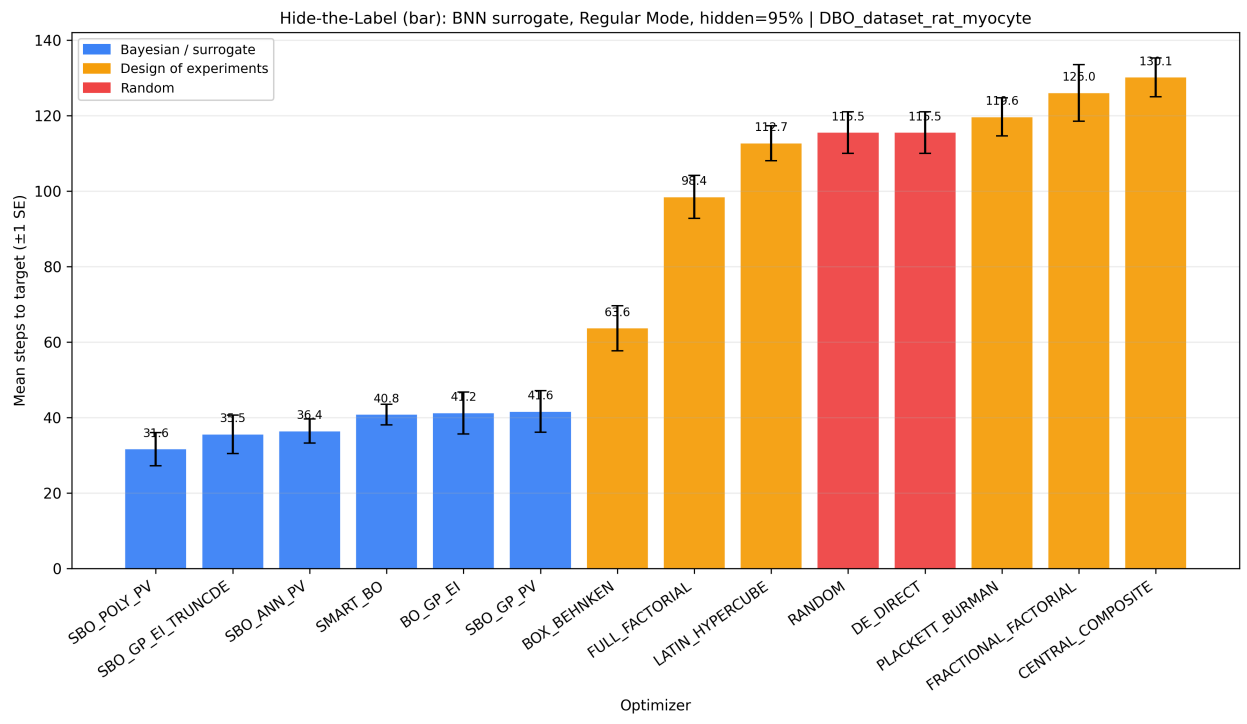

(a) DBO rat myocyte.

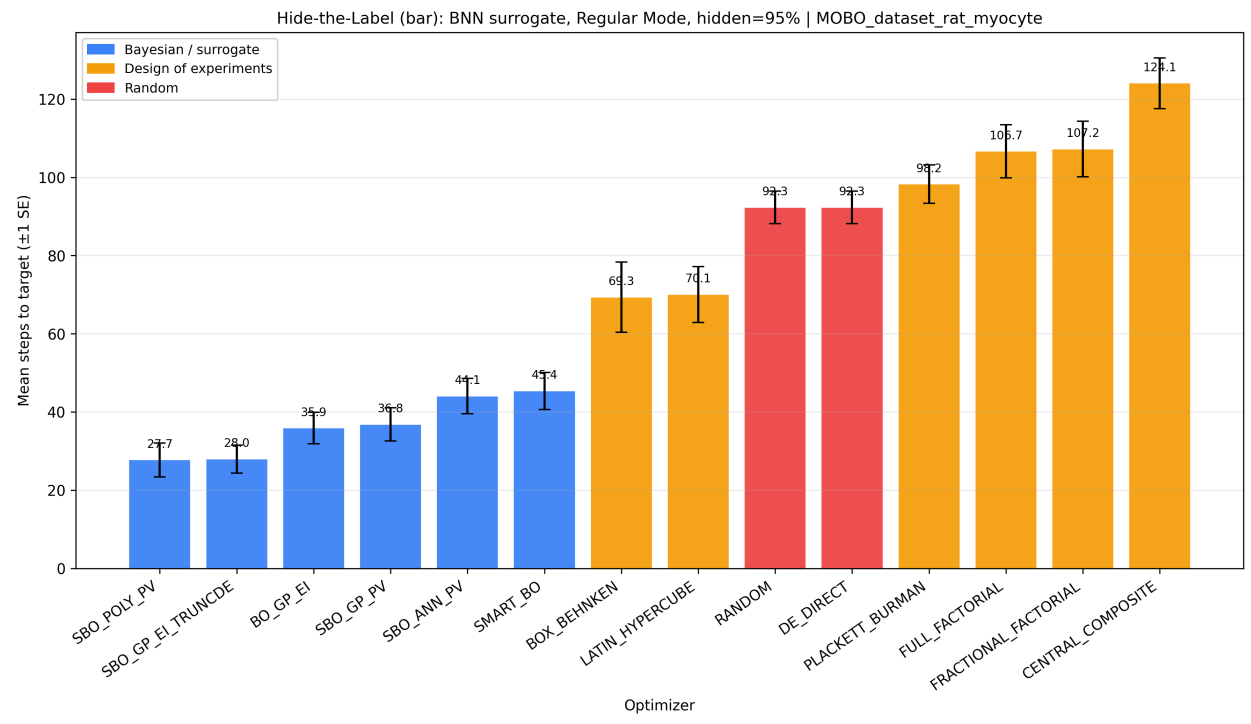

(b) MOBO rat myocyte.

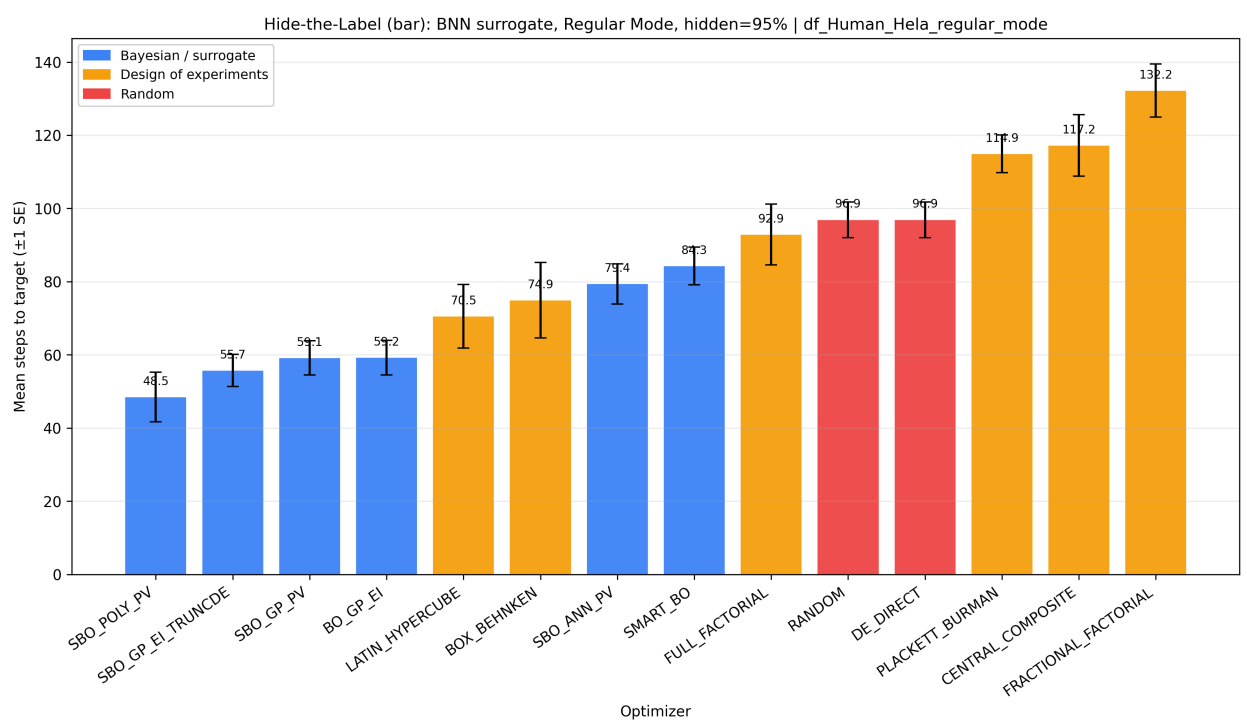

(c) HeLa (regular mode).

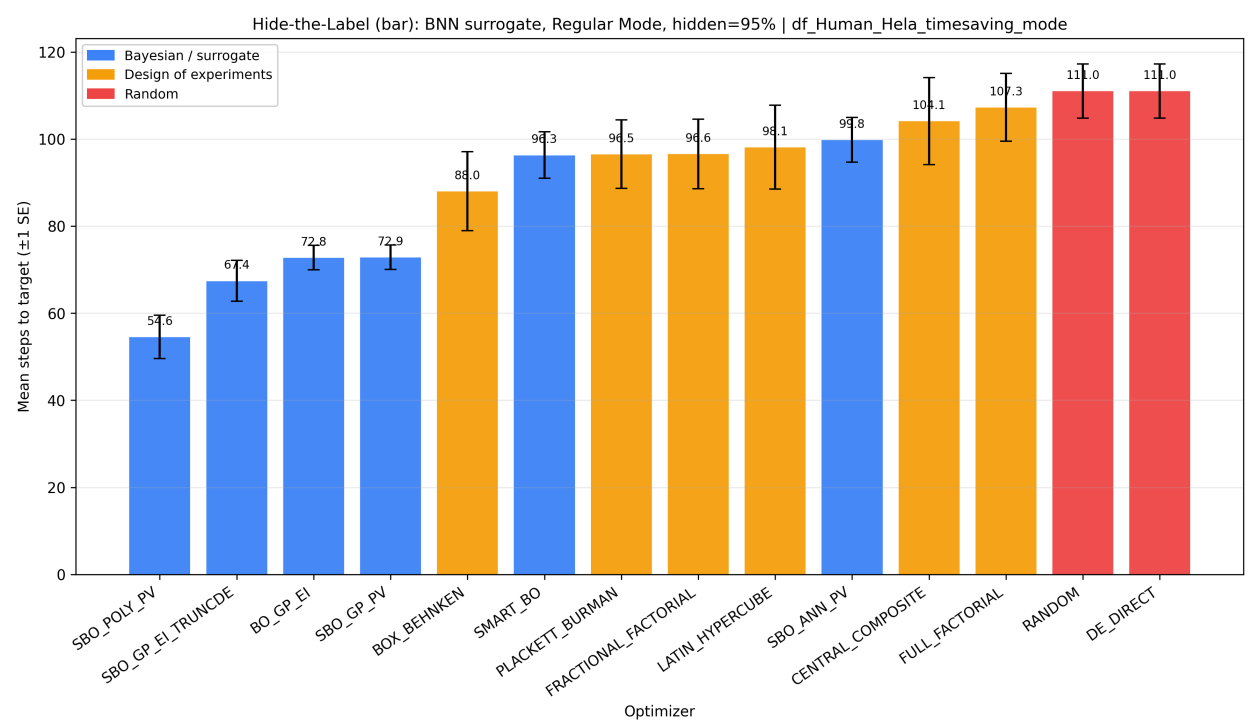

(d) HeLa (time-saving mode).

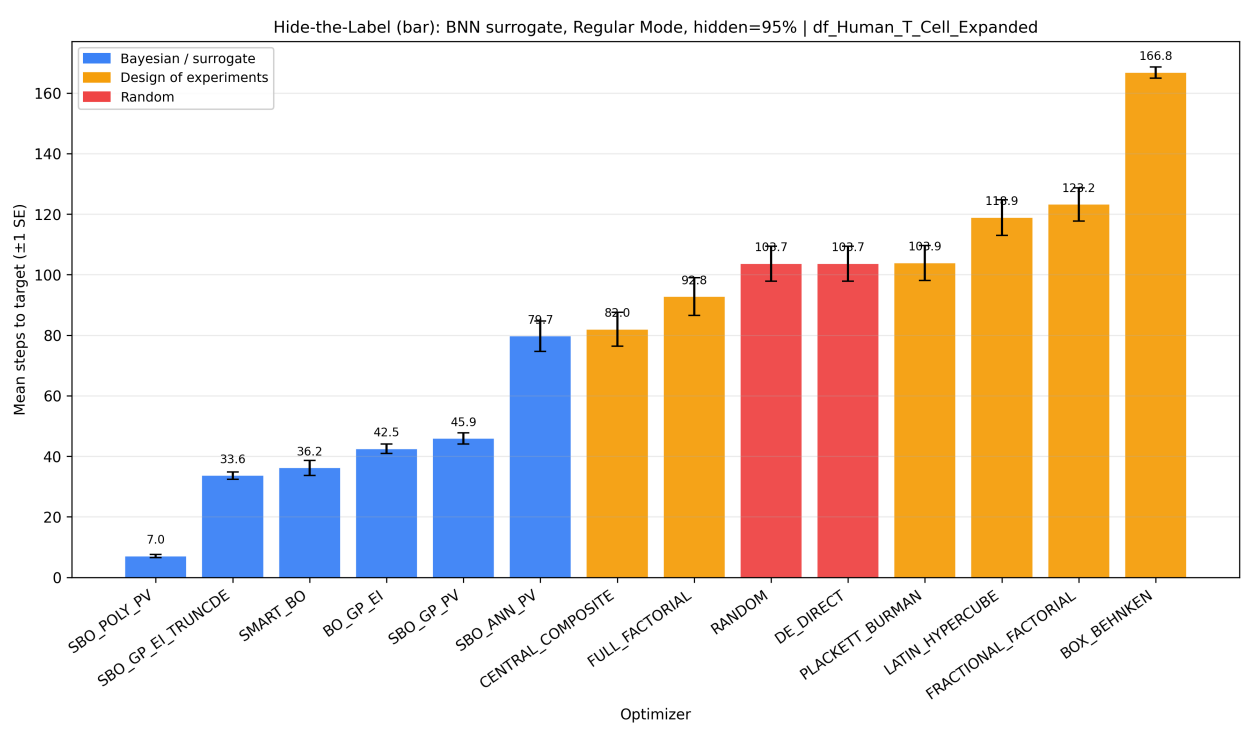

(e) T-Cell.

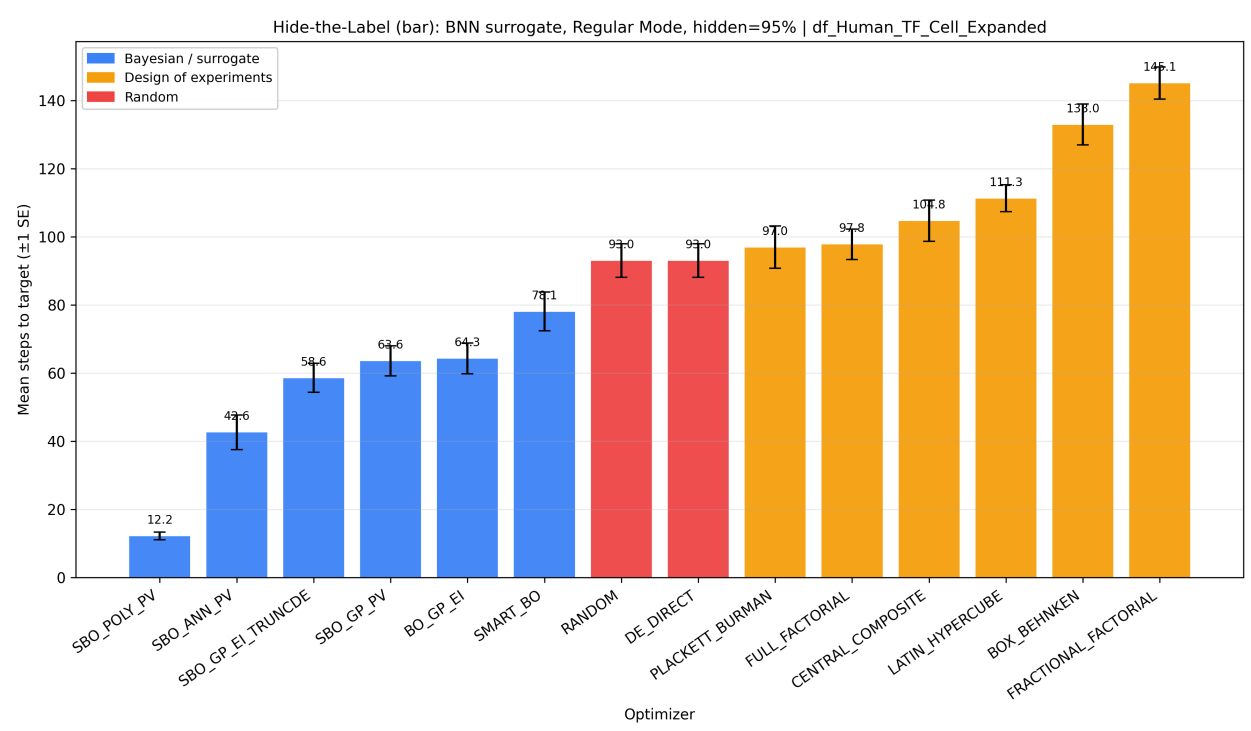

(f) TF-Cell.

Fig. 21. Hide-the-Label (HTL) competition results. **Configuration:** Regular Mode (BNN surrogate, low noise), hidden fraction = 95% (5% initially revealed), Batch size = 1. **Datasets:** DBO rat myocyte (a), MOBO rat myocyte (b), HeLa regular mode (c), HeLa time-saving mode (d), T-Cell (e), TF-Cell (f). **Metric:** steps to find target label (lower is better).

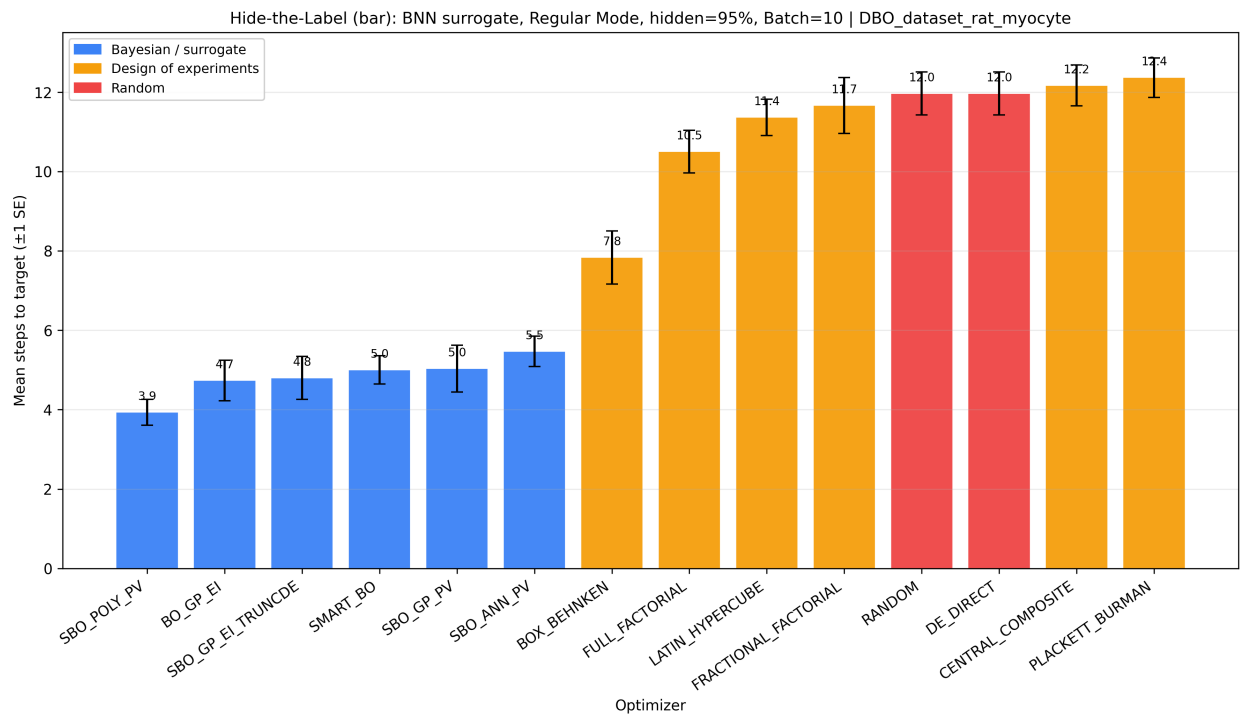

(a) DBO rat myocyte.

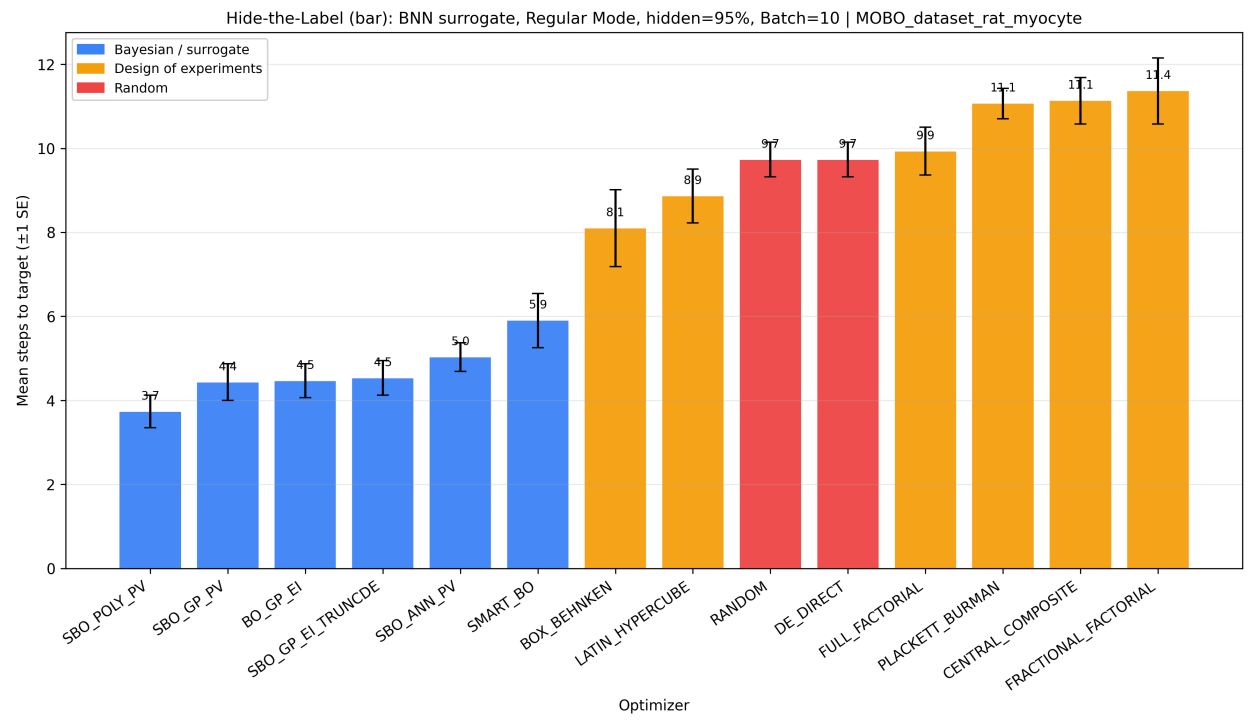

(b) MOBO rat myocyte.

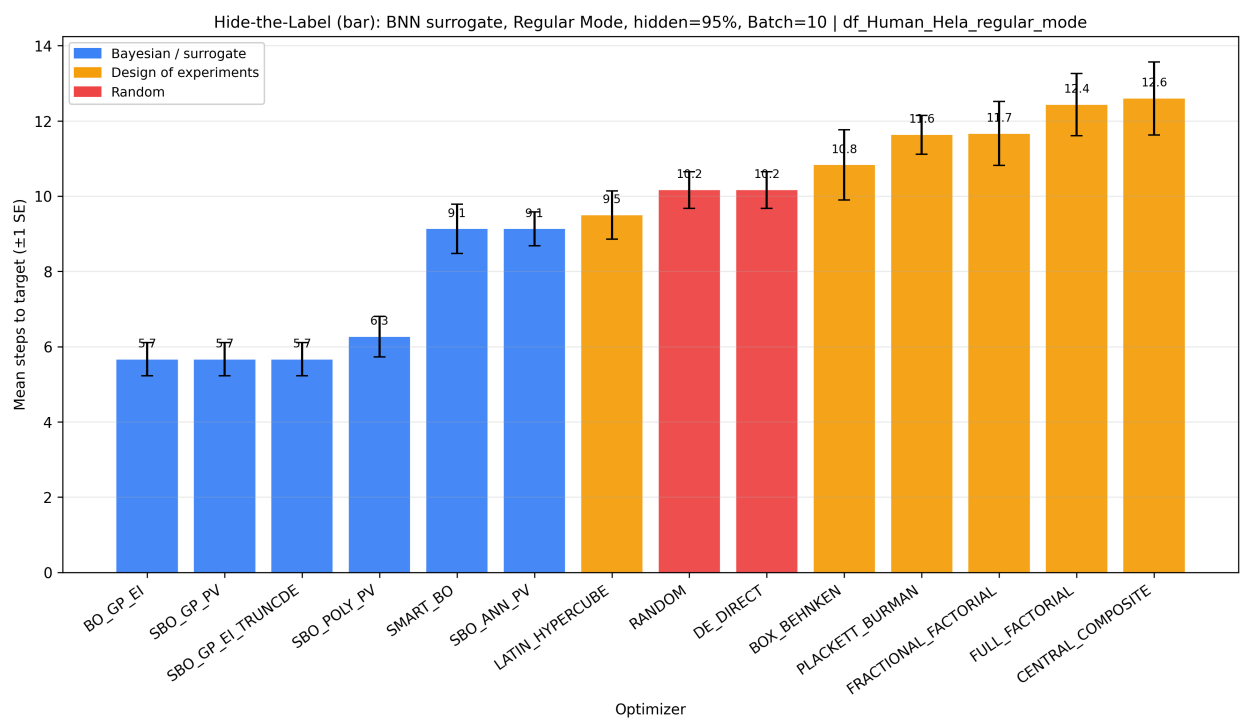

(c) HeLa (regular mode).

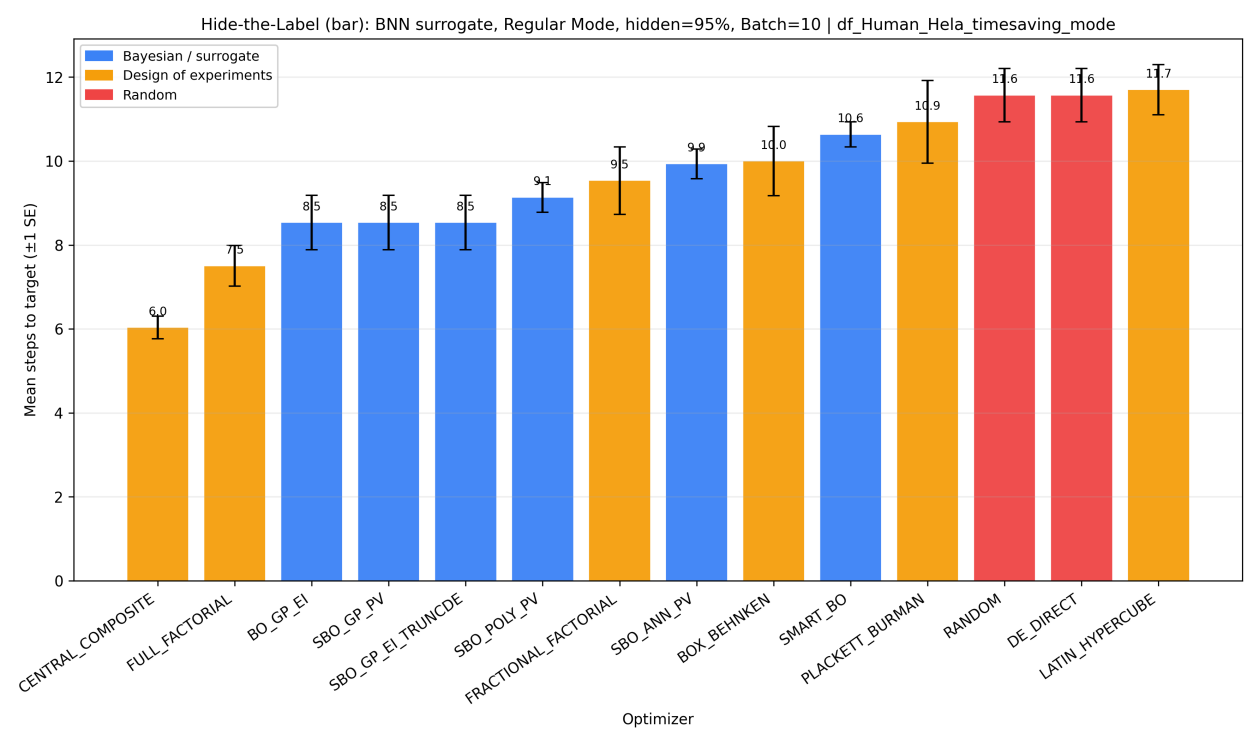

(d) HeLa (time-saving mode).

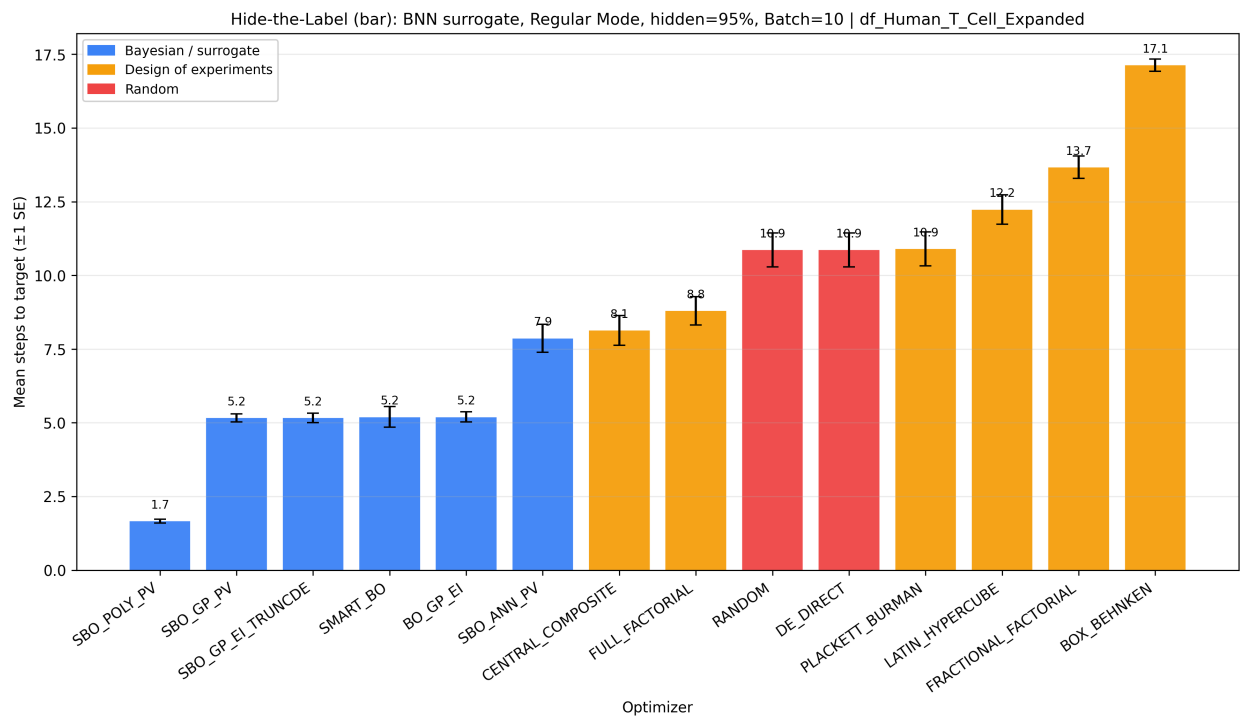

(e) T-Cell.

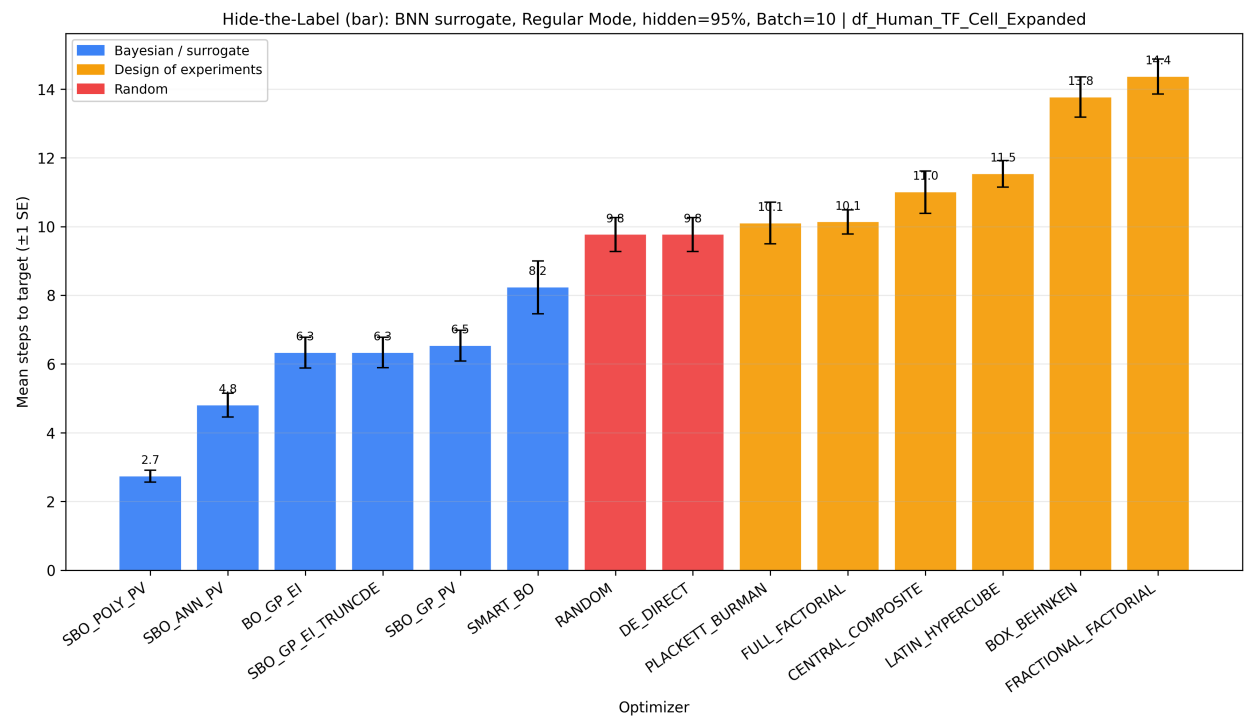

(f) TF-Cell.

Fig. 22. Hide-the-Label (HTL) competition results. **Configuration:** Regular Mode (BNN surrogate, low noise), hidden fraction = 95% (5% initially revealed), Batch size = 10. **Datasets:** DBO rat myocyte (a), MOBO rat myocyte (b), HeLa regular mode (c), HeLa time-saving mode (d), T-Cell (e), TF-Cell (f). **Metric:** steps to find target label (lower is better).

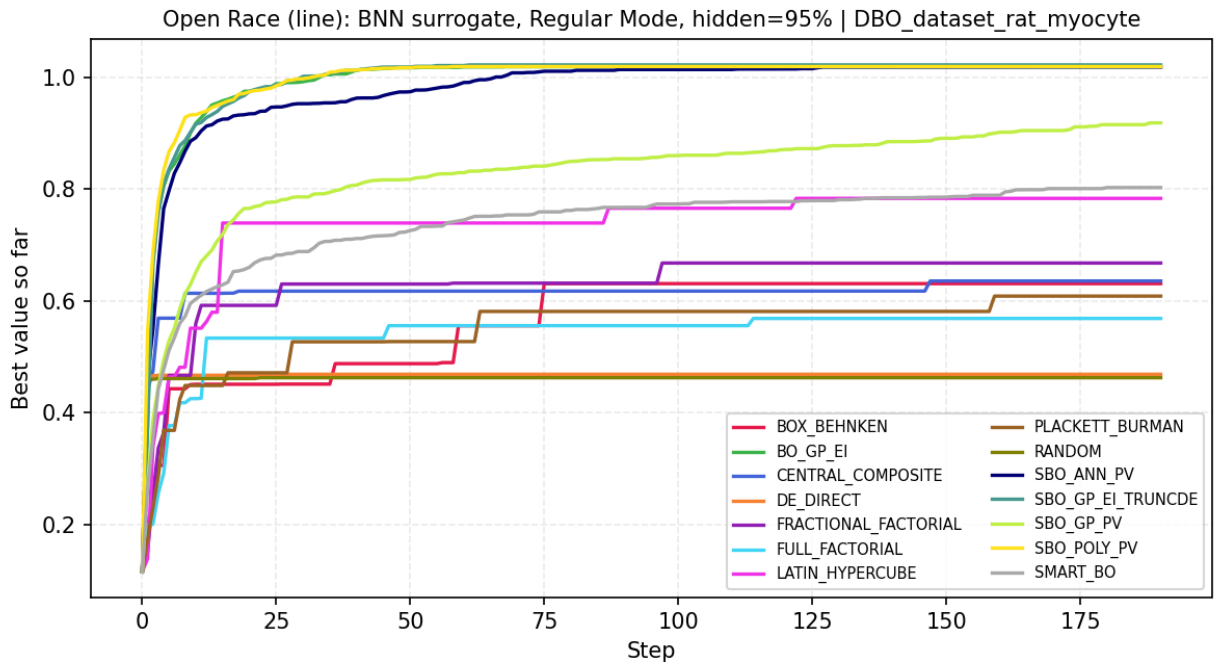

(a) DBO rat myocyte.

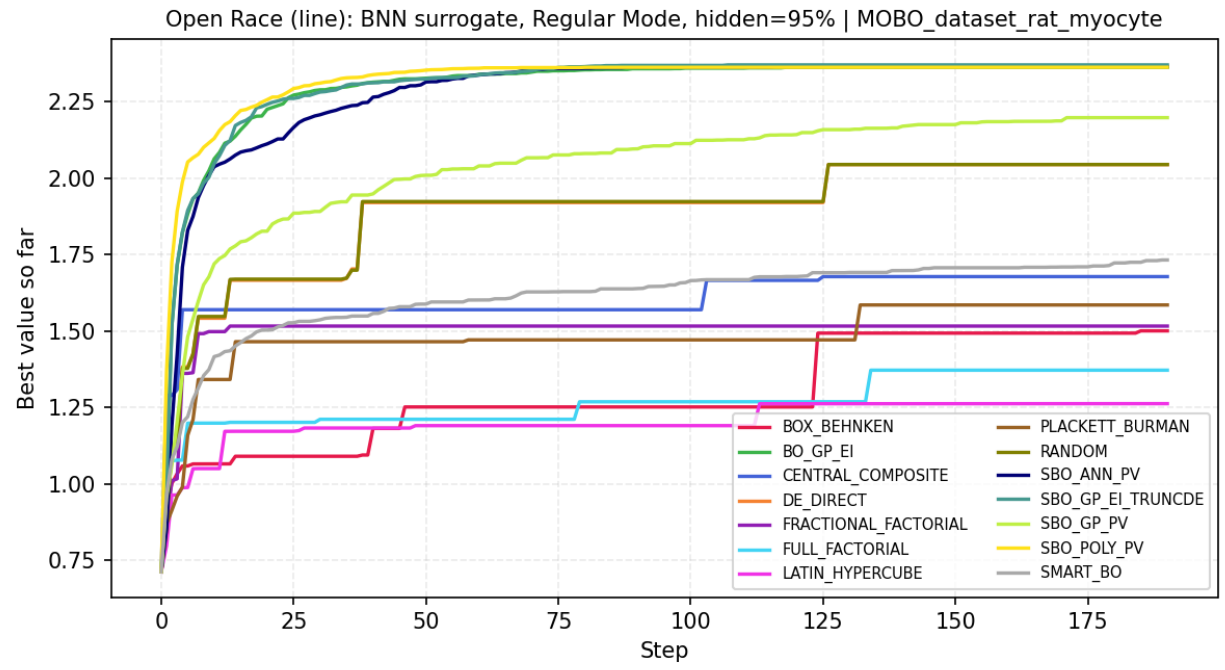

(b) MOBO rat myocyte.

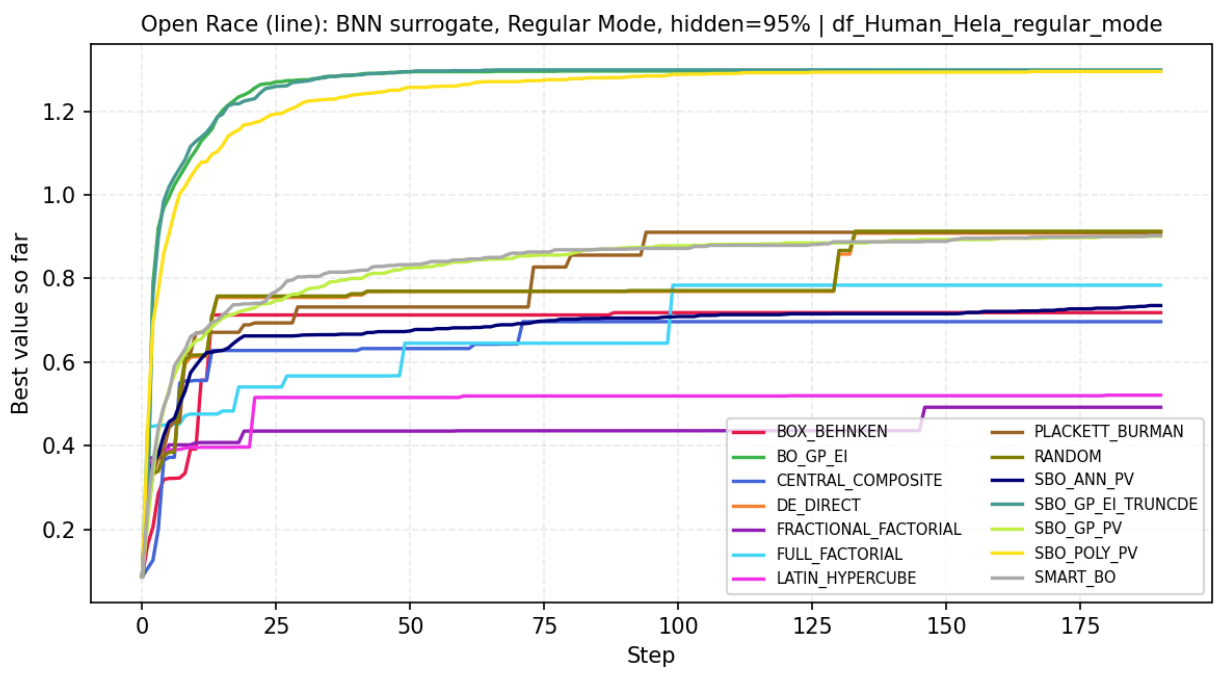

(c) HeLa (regular mode).

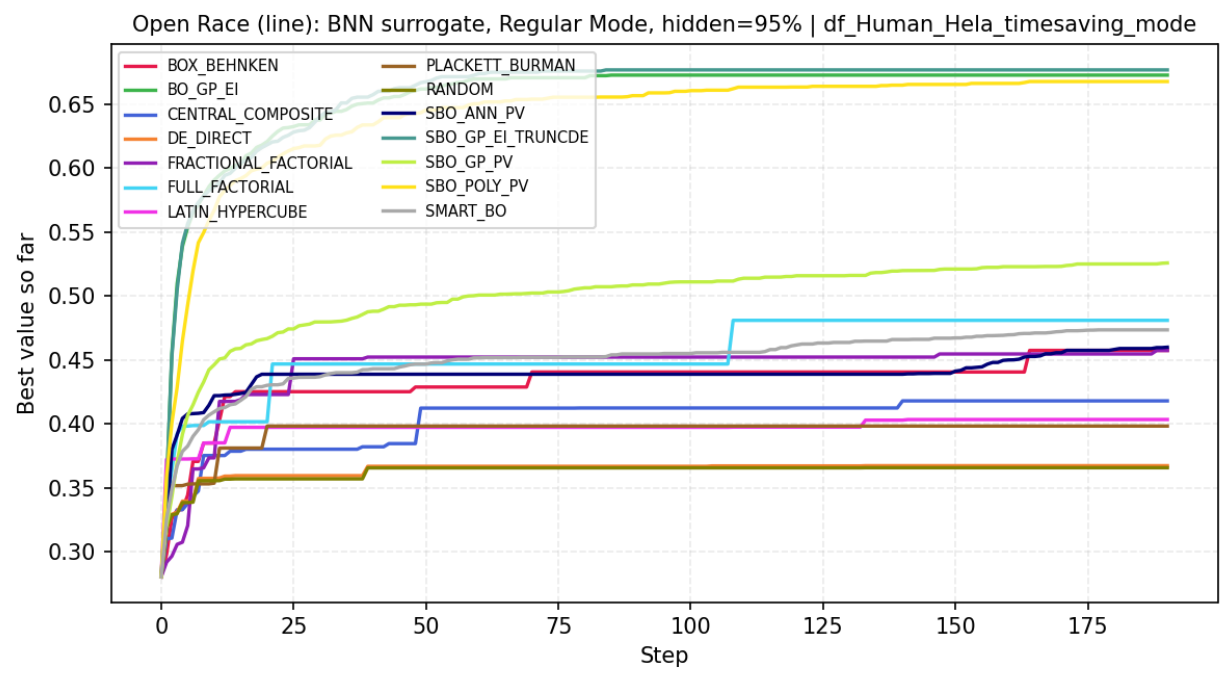

(d) HeLa (time-saving mode).

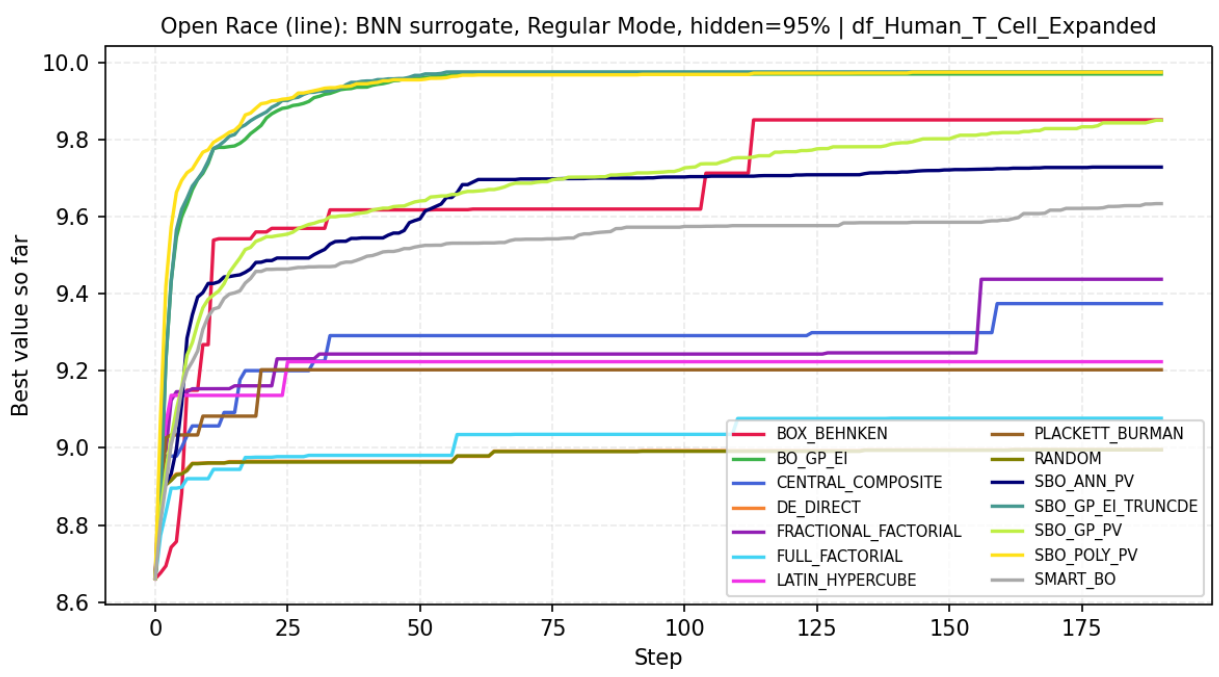

(e) T-Cell.

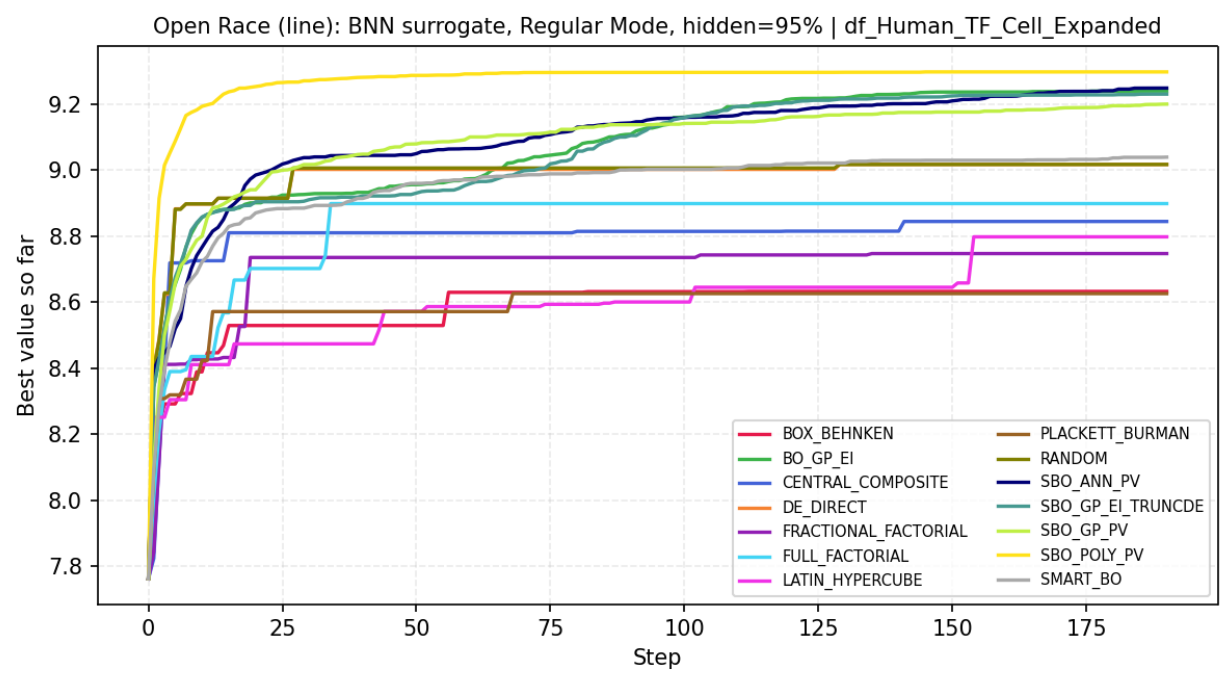

(f) TF-Cell.

Fig. 23. Open Race (OR) competition results. **Configuration:** Regular Mode (BNN surrogate, low noise), hidden fraction = 95% (5% initially revealed). **Datasets:** DBO rat myocyte (a), MOBO rat myocyte (b), HeLa regular mode (c), HeLa time-saving mode (d), T-Cell (e), TF-Cell (f). **Metric:** best value found so far over cumulative evaluation steps (higher is better).

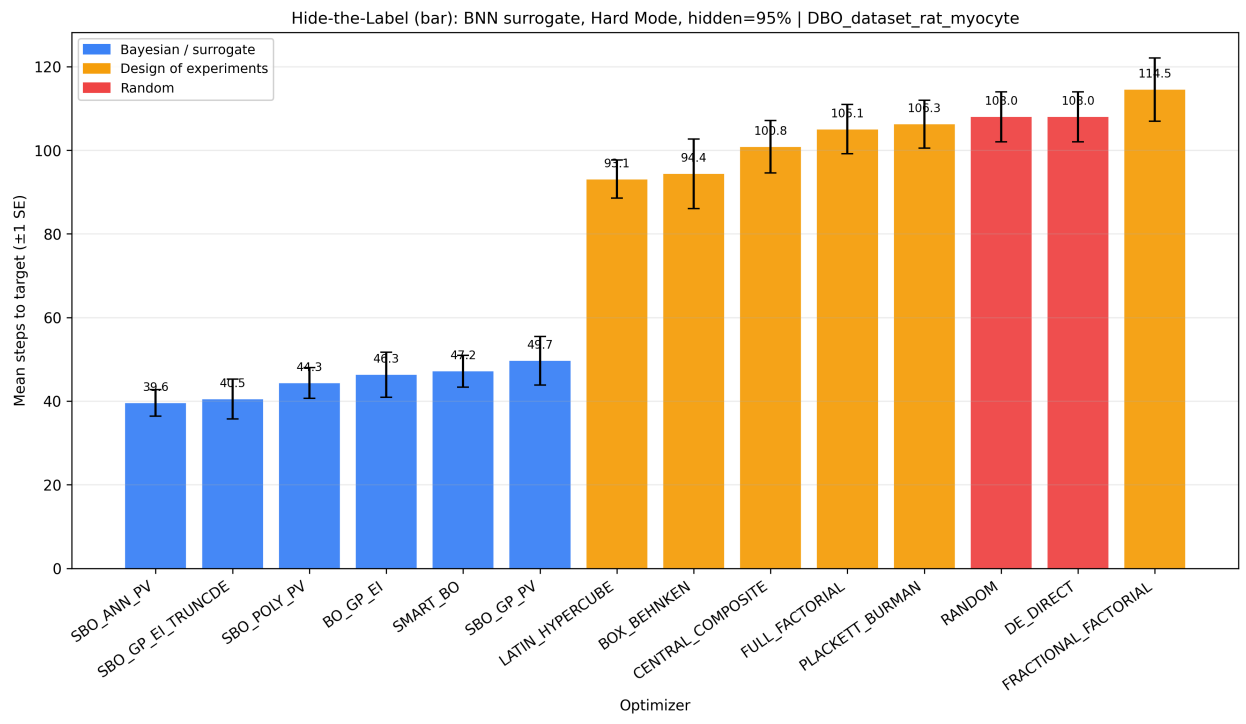

(a) DBO rat myocyte.

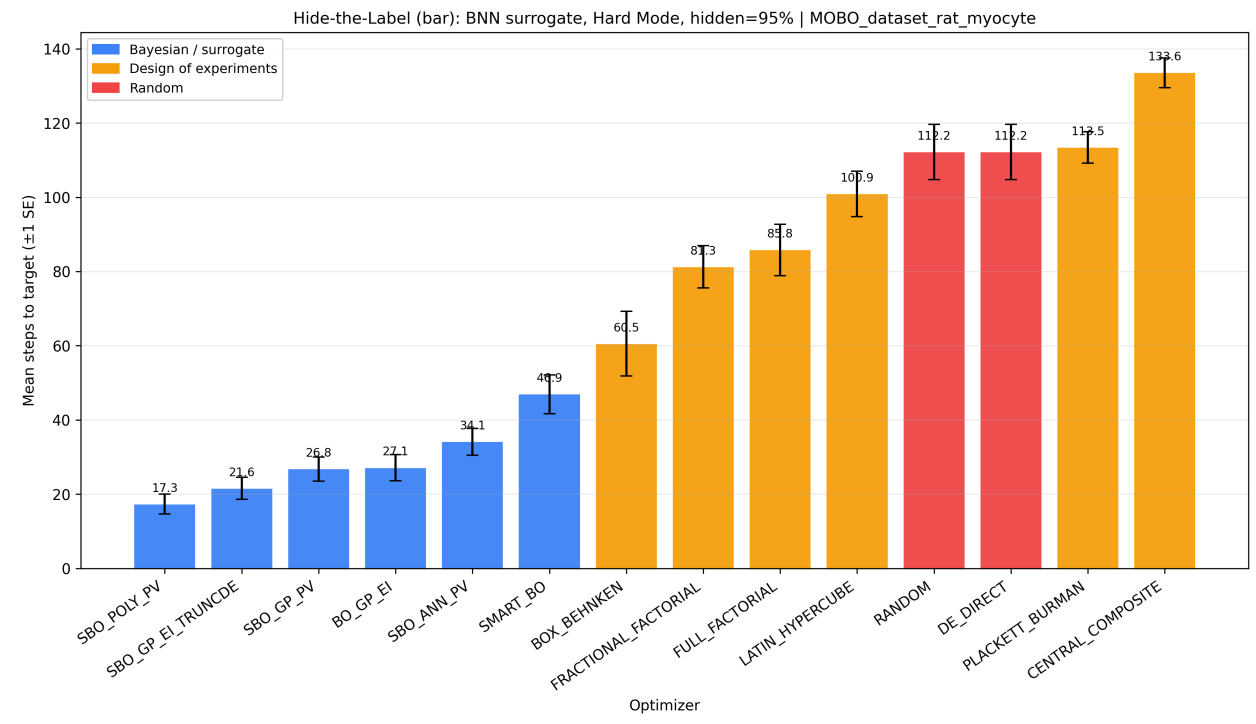

(b) MOBO rat myocyte.

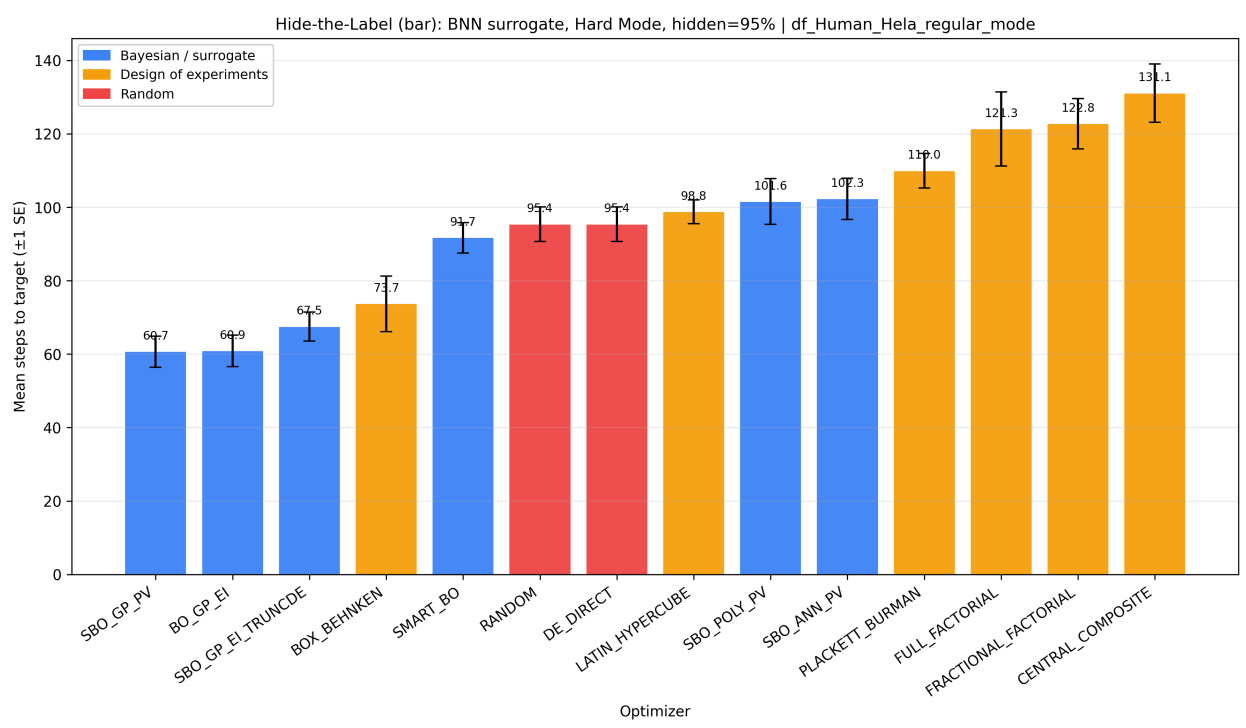

(c) HeLa (regular mode).

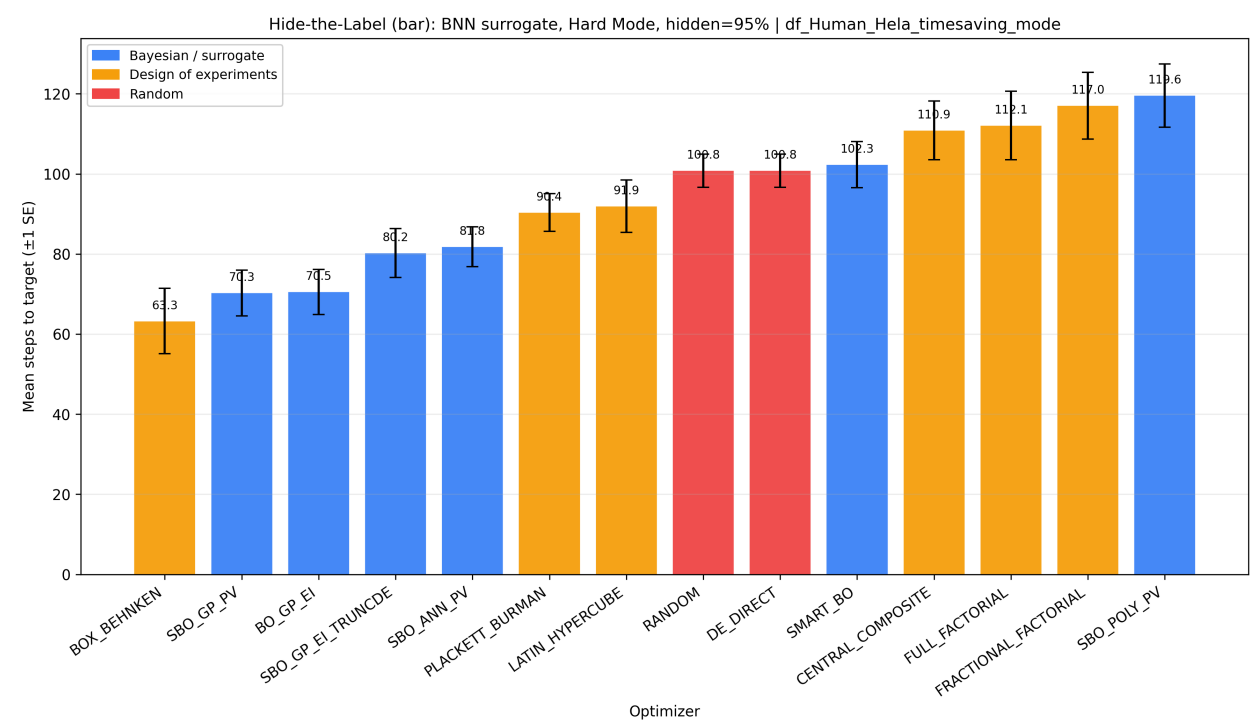

(d) HeLa (time-saving mode).

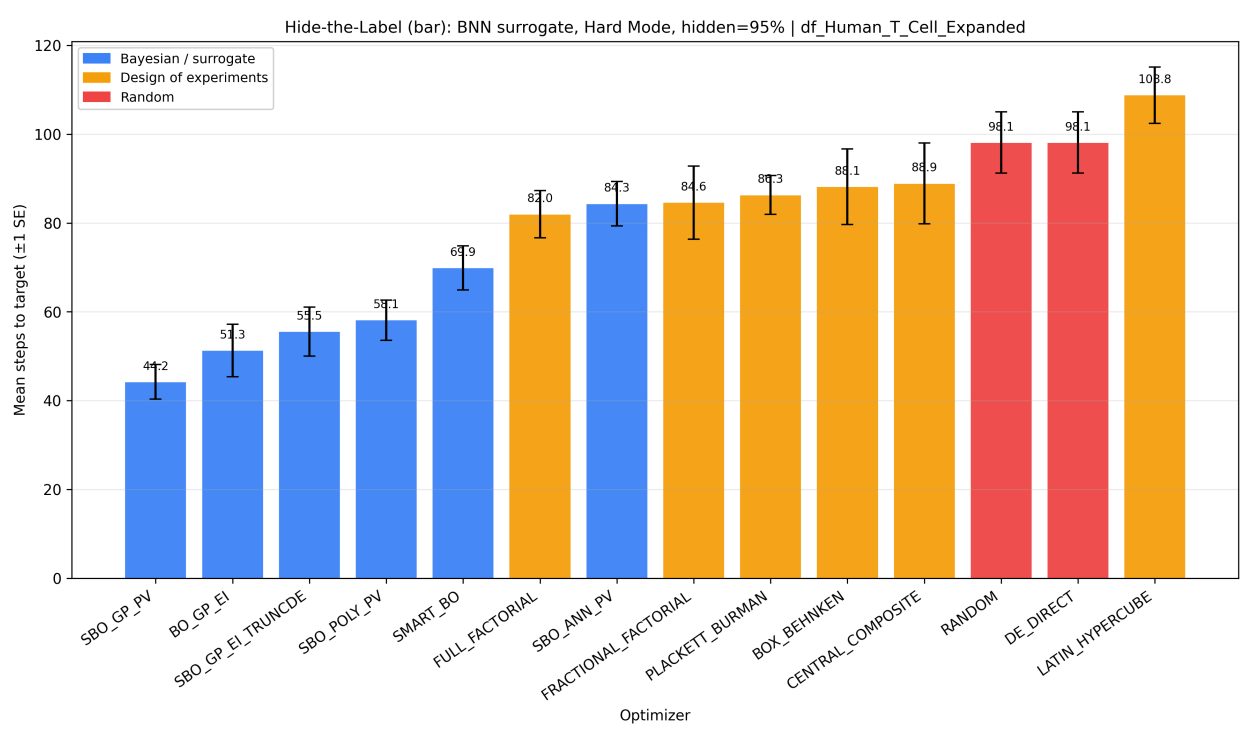

(e) T-Cell.

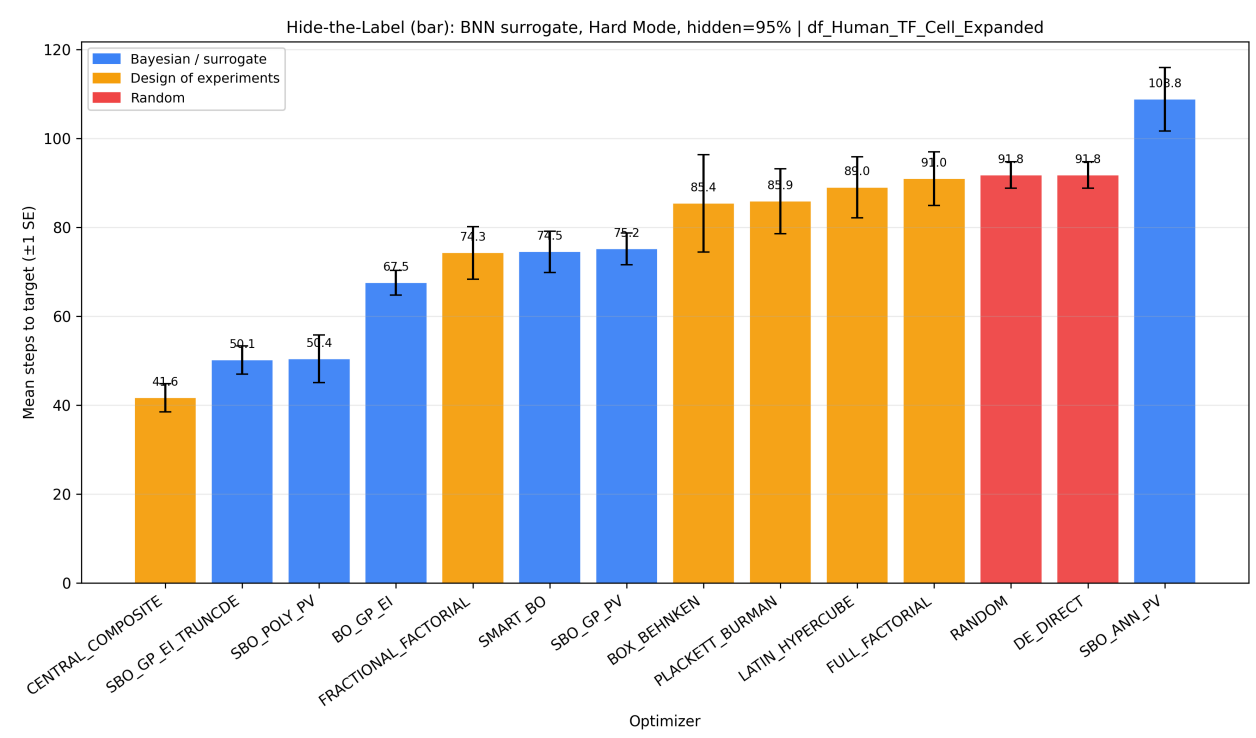

(f) TF-Cell.

Fig. 24. Hide-the-Label (HTL) competition results. **Configuration:** Hard Mode (BNN surrogate, heteroskedastic noise & bimodal perturbations), hidden fraction = 95% (5% initially revealed), Batch size = 1. **Datasets:** DBO rat myocyte (a), MOBO rat myocyte (b), HeLa regular mode (c), HeLa time-saving mode (d), T-Cell (e), TF-Cell (f). **Metric:** steps to find target label (lower is better).

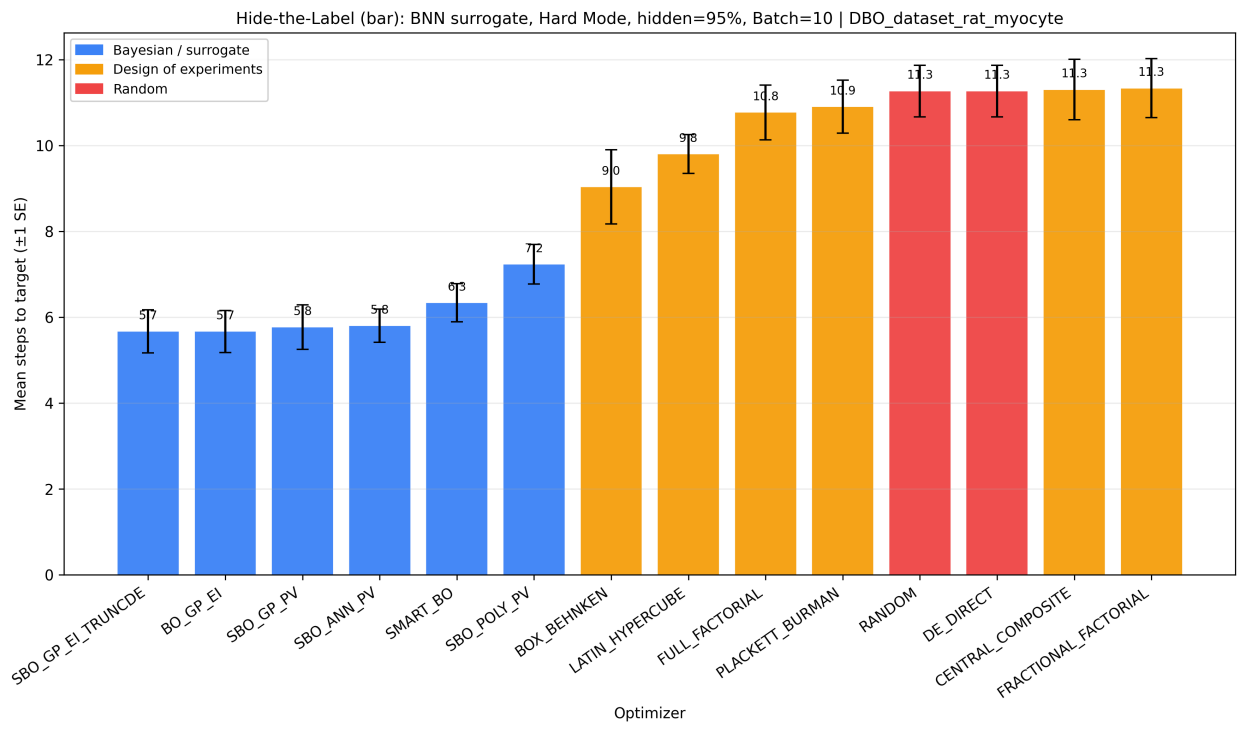

(a) DBO rat myocyte.

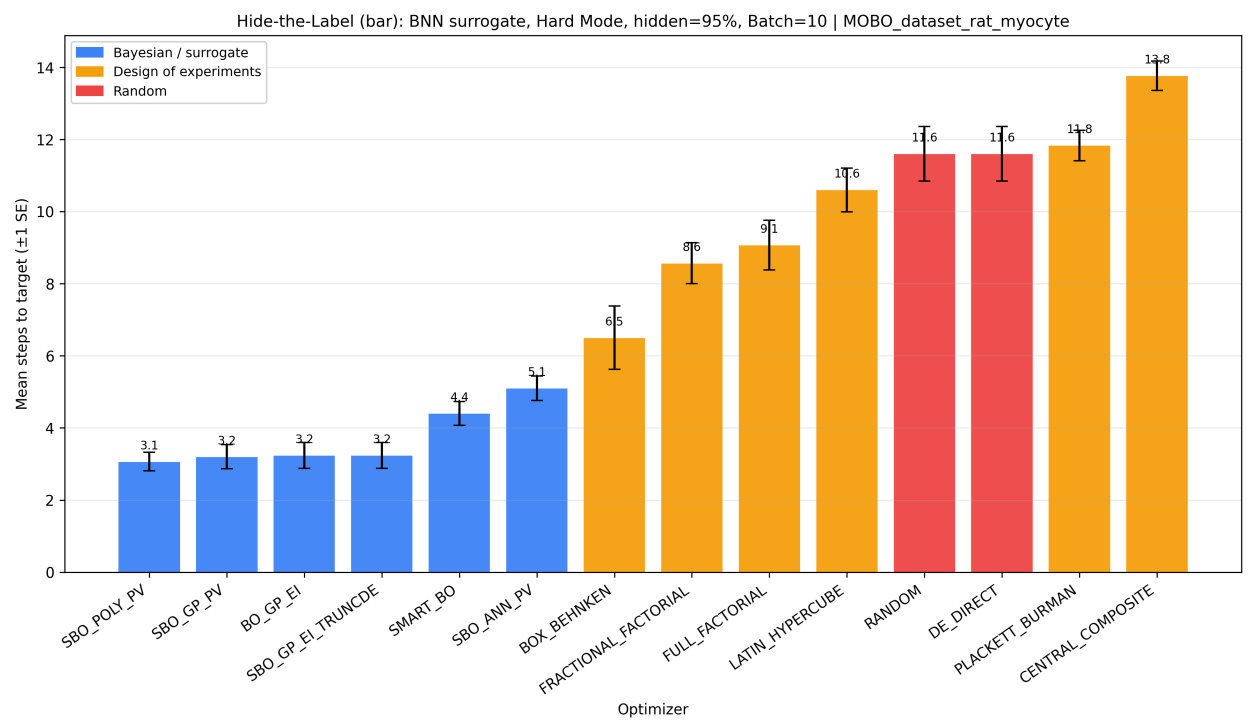

(b) MOBO rat myocyte.

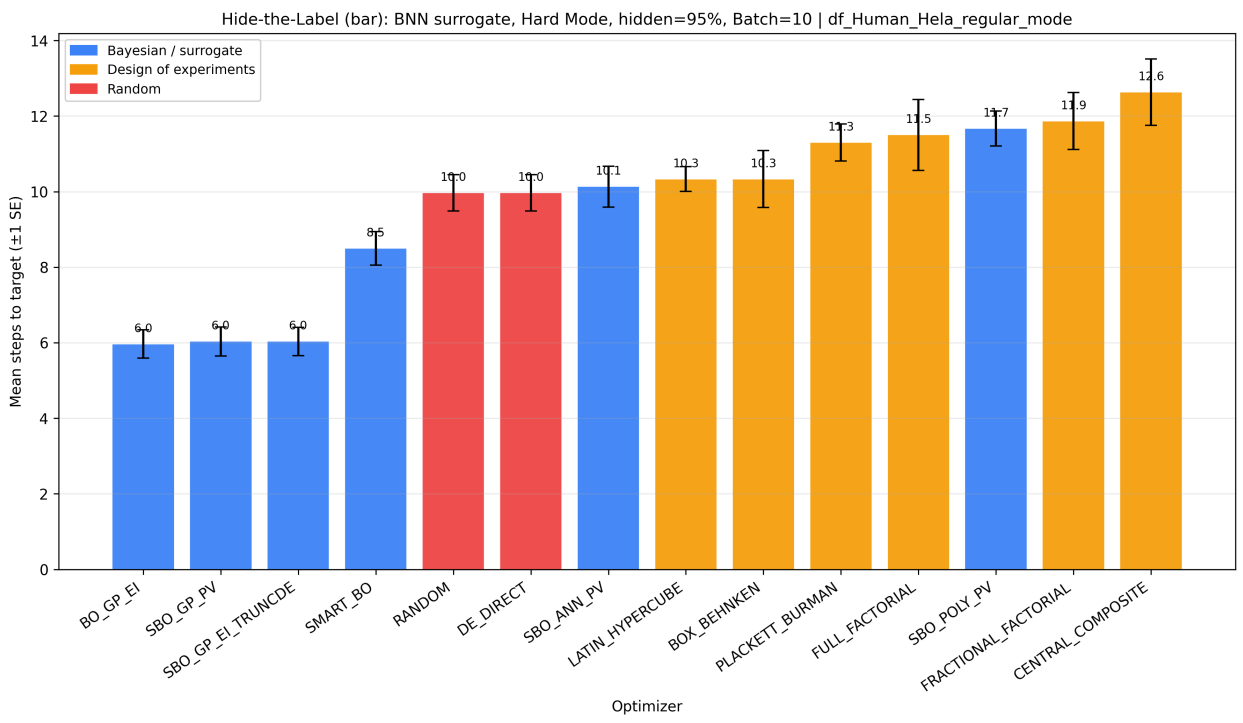

(c) HeLa (regular mode).

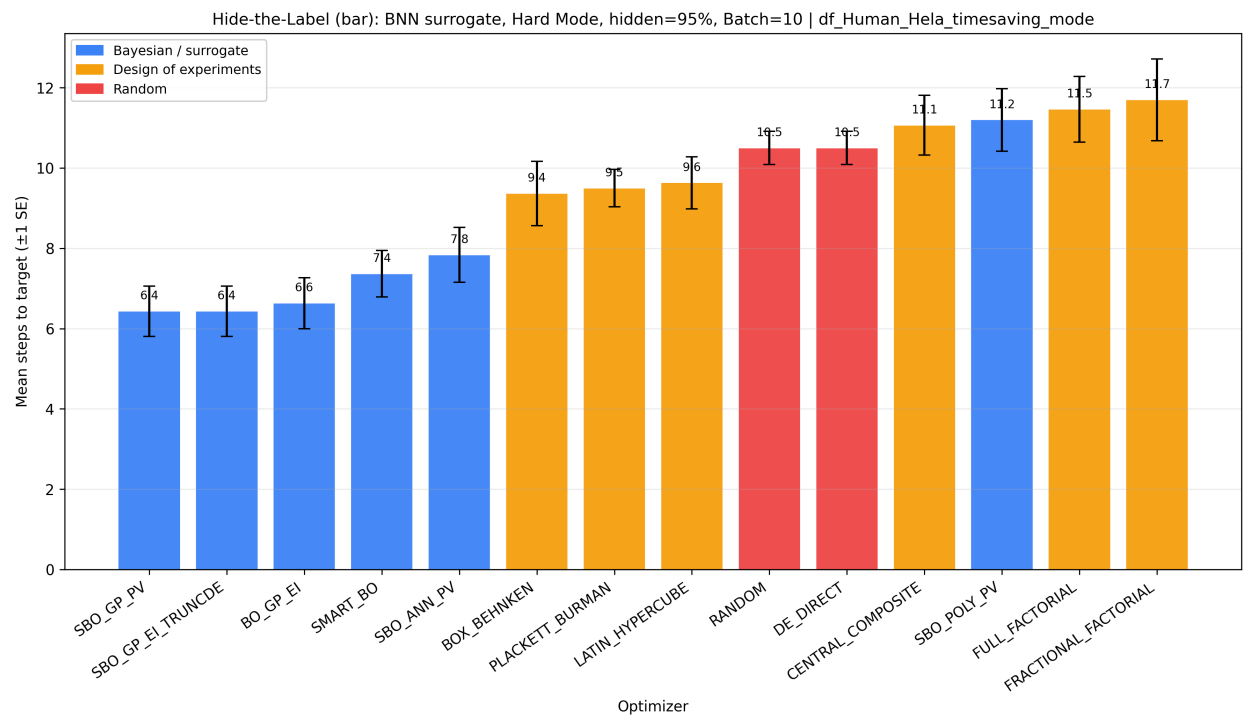

(d) HeLa (time-saving mode).

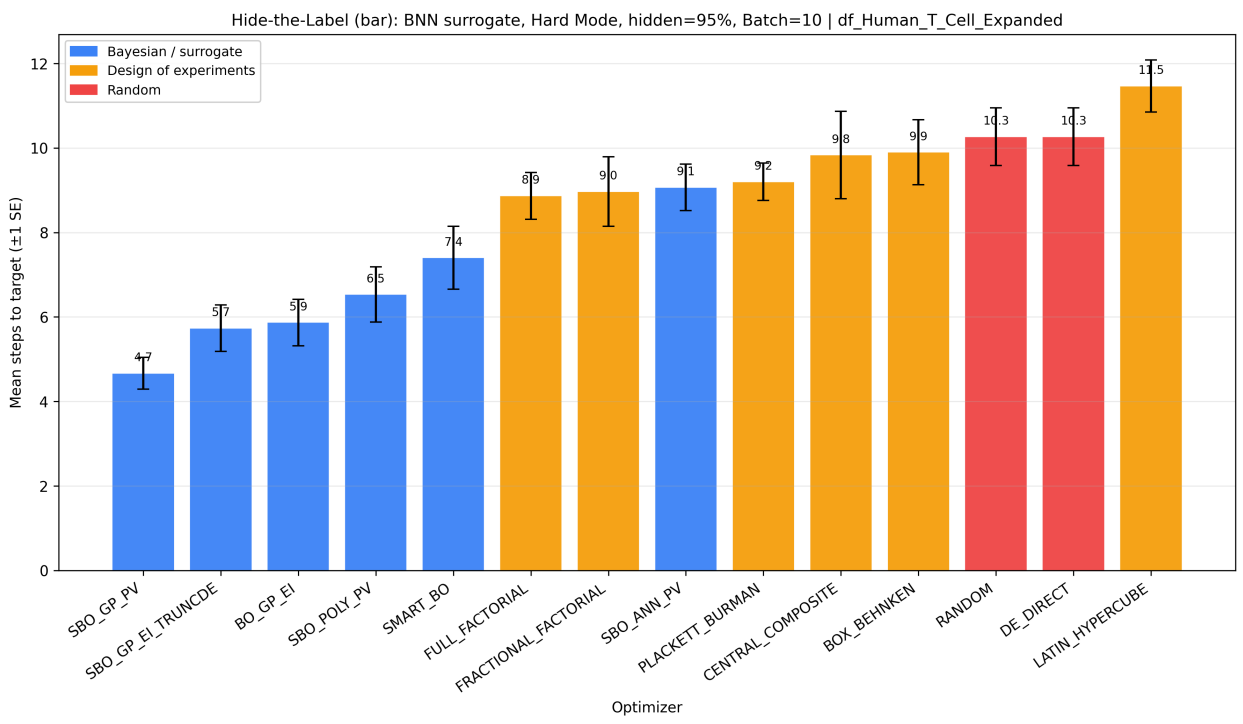

(e) T-Cell.

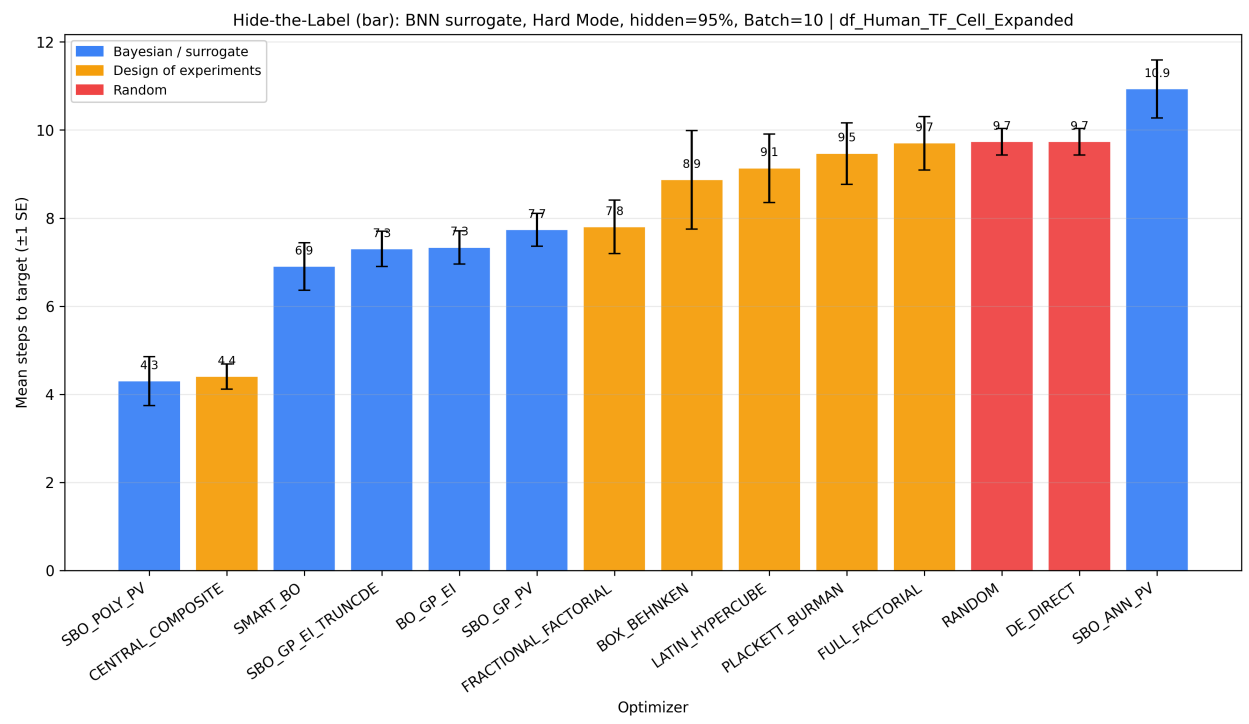

(f) TF-Cell.

Fig. 25. Hide-the-Label (HTL) competition results. **Configuration:** Hard Mode (BNN surrogate, heteroskedastic noise & bimodal perturbations), hidden fraction = 95% (5% initially revealed), Batch size = 10. **Datasets:** DBO rat myocyte (a), MOBO rat myocyte (b), HeLa regular mode (c), HeLa time-saving mode (d), T-Cell (e), TF-Cell (f). **Metric:** steps to find target label (lower is better).

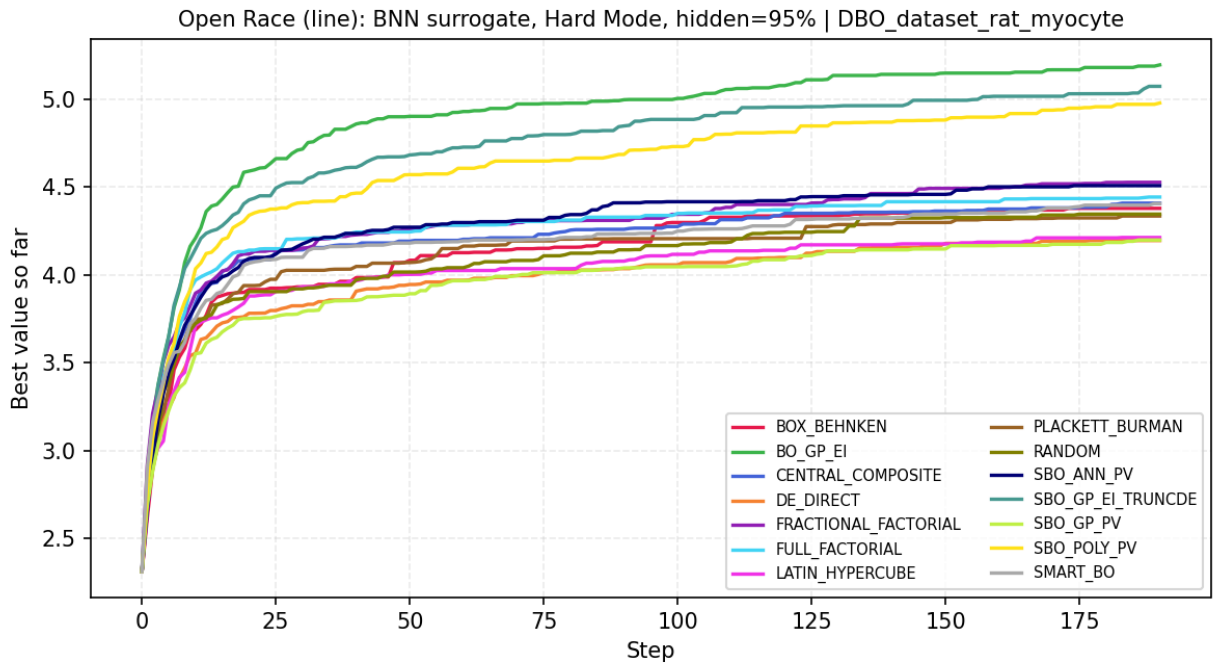

(a) DBO rat myocyte.

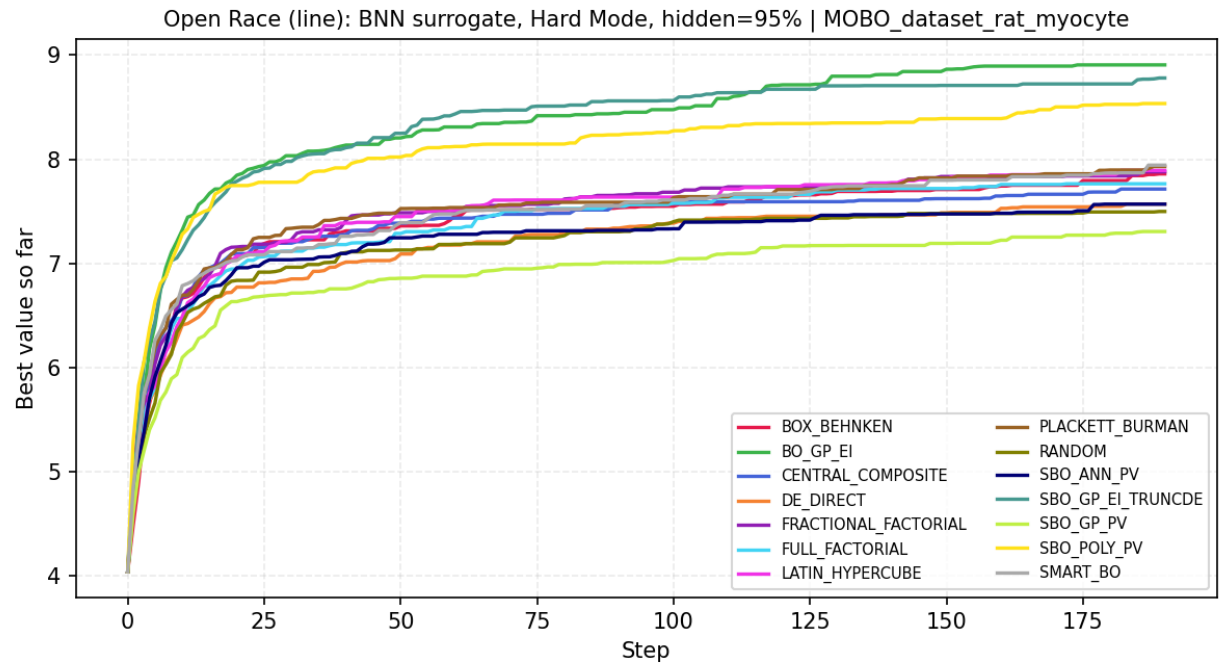

(b) MOBO rat myocyte.

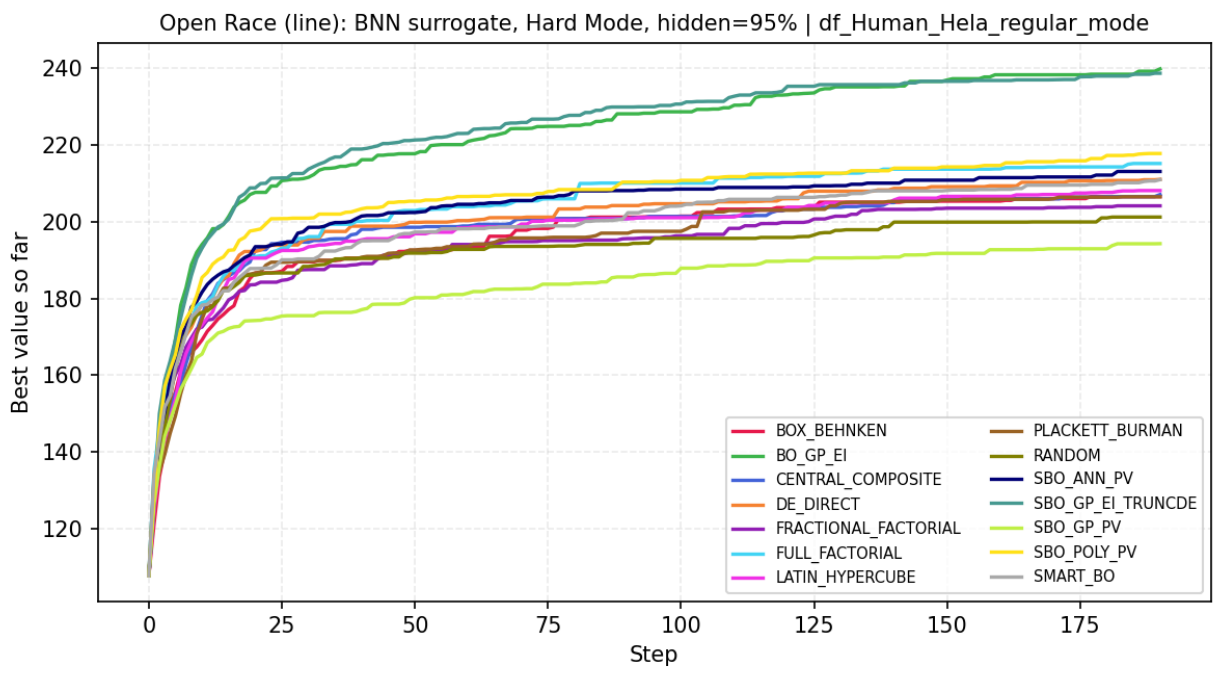

(c) HeLa (regular mode).

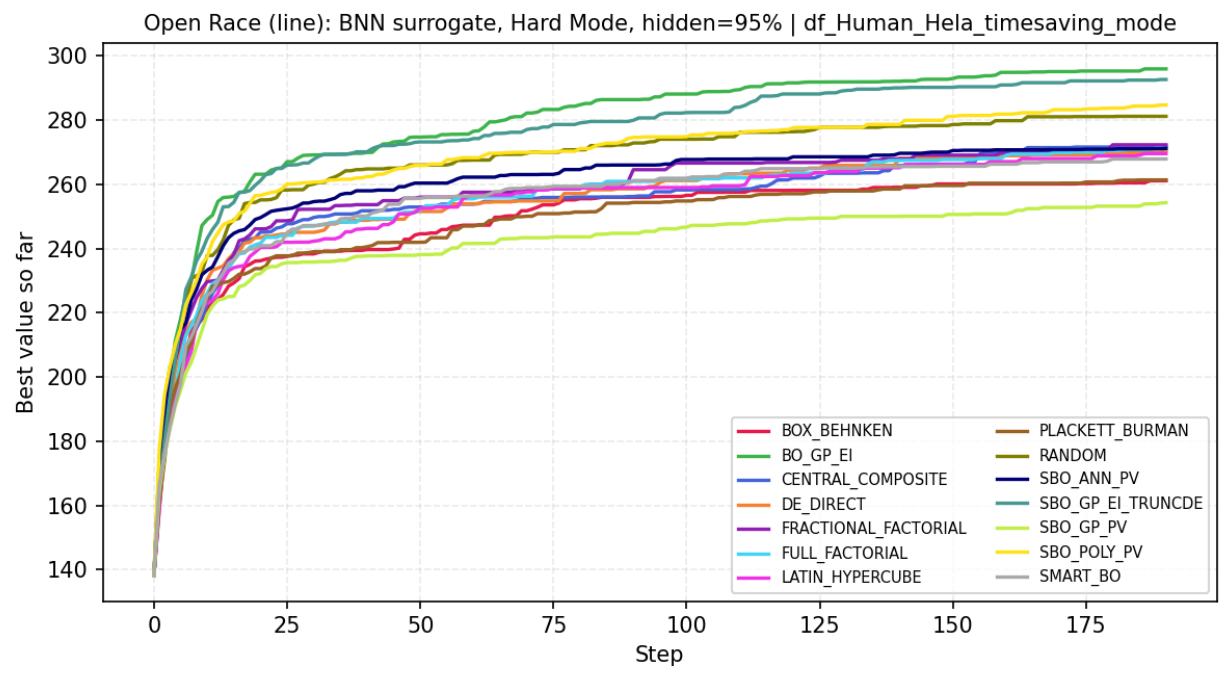

(d) HeLa (time-saving mode).

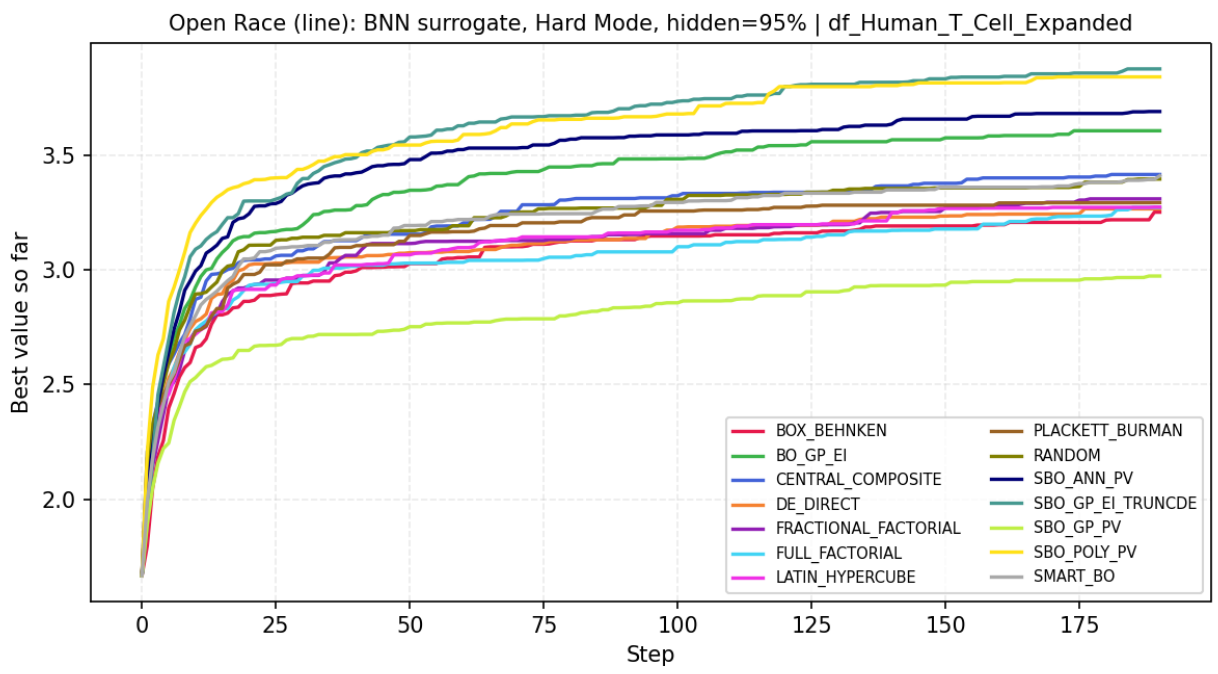

(e) T-Cell.

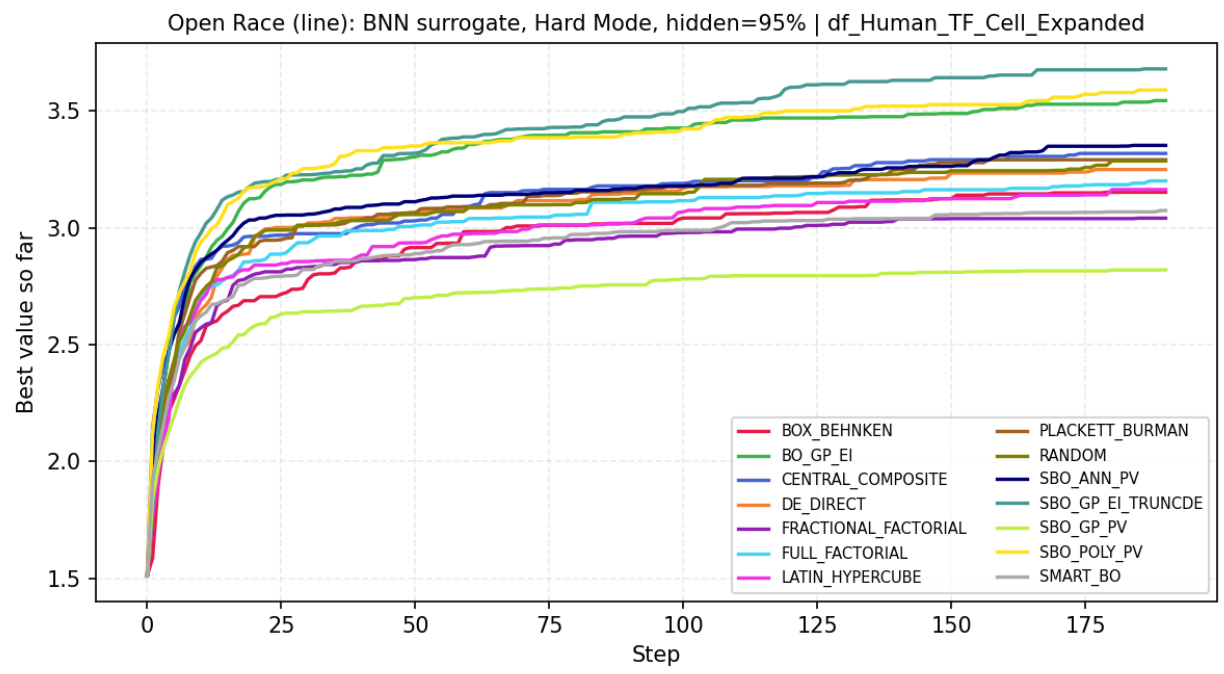

(f) TF-Cell.

Fig. 26. Open Race (OR) competition results. **Configuration:** Hard Mode (BNN surrogate, heteroskedastic noise & bimodal perturbations), hidden fraction = 95% (5% initially revealed). **Datasets:** DBO rat myocyte (a), MOBO rat myocyte (b), HeLa regular mode (c), HeLa time-saving mode (d), T-Cell (e), TF-Cell (f). **Metric:** best value found so far over cumulative evaluation steps (higher is better).
